# An ATP-sensitive phosphoketolase regulates carbon fixation in cyanobacteria

**DOI:** 10.1038/s42255-023-00831-w

**Published:** 2023-06-22

**Authors:** Kuan-Jen Lu, Chiung-Wen Chang, Chun-Hsiung Wang, Frederic Y-H Chen, Irene Y. Huang, Pin-Hsuan Huang, Cheng-Han Yang, Hsiang-Yi Wu, Wen-Jin Wu, Kai-Cheng Hsu, Meng-Chiao Ho, Ming-Daw Tsai, James C. Liao

**Affiliations:** 1 Institute of Biological Chemistry, Academia Sinica, Taipei, Taiwan; 2 Graduate Institute of Cancer Biology and Drug Discovery, Taipei Medical University, Taipei, Taiwan; 3 Institute of Biochemical Sciences, National Taiwan University, Taipei, Taiwan

**Keywords:** Metabolism, Cryoelectron microscopy, Photosynthesis

## Abstract

Regulation of CO_2_ fixation in cyanobacteria is important both for the organism and global carbon balance. Here we show that phosphoketolase in *Synechococcus* *elongatus* PCC7942 (*Se*XPK) possesses a distinct ATP-sensing mechanism, where a drop in ATP level allows *Se*XPK to divert precursors of the RuBisCO substrate away from the Calvin–Benson–Bassham cycle. Deleting the *Se*XPK gene increased CO_2_ fixation particularly during light–dark transitions. In high-density cultures, the *Δxpk* strain showed a 60% increase in carbon fixation and unexpectedly resulted in sucrose secretion without any pathway engineering. Using cryo-EM analysis, we discovered that these functions were enabled by a unique allosteric regulatory site involving two subunits jointly binding two ATP, which constantly suppresses the activity of *Se*XPK until the ATP level drops. This magnesium-independent ATP allosteric site is present in many species across all three domains of life, where it may also play important regulatory functions.

## Abstract (web summary)

Marine cyanobacteria contribute to global carbon balance by fixing CO_2_ and the shift between CO_2_ fixation and ATP production requires fine-tuning its metabolic fluxes to light–dark cycles. These cycles can be very short in marine environments due to sea currents and fast adaptation is key to avoid futile cycles. In this study, Lu et al. provide a mechanistic insight into how this process is tightly regulated.

## Main

**Fig. 1:**
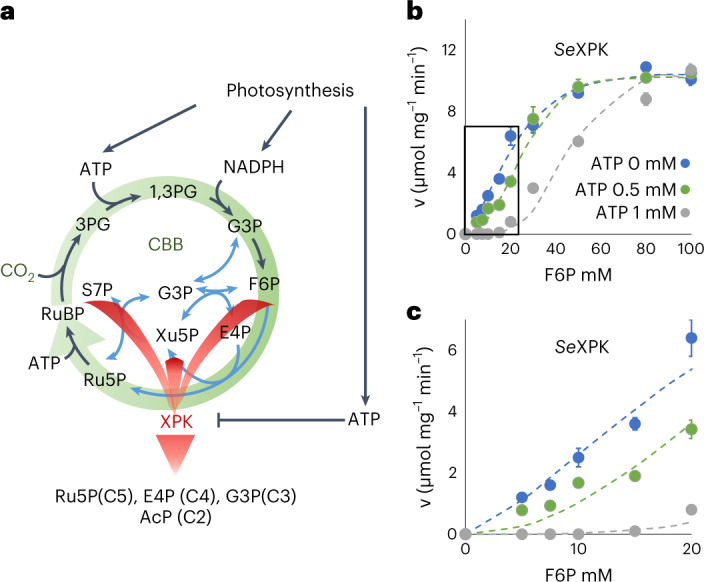
Effects of ATP on the activity of *Se*XPK. **a**, Proposed roles of *Se*XPK in response to ATP levels. When ATP levels are high during photosynthesis, *Se*XPK is inactive. When ATP levels are low, *Se*XPK would break down CBB cycle intermediates, including Xu5P, F6P and S7P, to stop carbon fixation. **b**, Plots of the initial activity of *Se*XPK against different concentrations of F6P in the presence of 0, 0.5 or 1 mM ATP and saturating 30 mM phosphate (*n* = 3 individual reactions, mean ± s.e.m.). **c**, Enlarged plot from **b**, in the region of F6P below 20 mM. Source data

Terrestrial phototrophic organisms draw down about 120 Gt of carbon from atmosphere annually primarily through the Calvin–Benson–Bassham (CBB) cycle^1^. Similar figures have been estimated for marine organisms. This flux is about ten-times higher than that emitted by anthropogenic release of CO_2_^2,3^ Thus, an increase in the biological carbon fixation by even a few percent would greatly influence the carbon balance on earth. Cyanobacteria utilize solar energy to split water and produce NADPH and ATP via photosystems and the electron transport chain to support carbon fixation via the CBB cycle^4^. The CBB cycle accounts for the vast majority of carbon fixation on earth^5–7^, in which RuBisCO is the major enzyme catalyzing conversion of CO_2_ and ribulose-1,5-bisphosphate (RuBP) to two molecules of 3-phosphoglycerate (3PG). Subsequent steps require ATP and NADPH to reduce 3PG to glyceraldehyde-3-phosphate (G3P), which can be metabolized to fructose-6-phosphate (F6P) as precursors for glycogen synthesis or to regenerate RuBP to continue the CBB cycle (Fig. 1a). The regulation of CBB cycle involves multiple factors and mechanisms, including light, redox potential and circadian regulation^8–11^. As a marine organism, cyanobacteria frequently experience underwater light fluctuation and encounter environmental stress. The cell has to shut down CO_2_ fixation rapidly in low-ATP conditions to avoid futile cycling resulting from simultaneous CO_2_ fixation and respiration. Although all ATP-dependent reactions proceed slower at low ATP levels, it is desirable to stop the CBB cycle preferentially and immediately to prevent non-productive resource draining; however, such a mechanism has not been discovered. On the other hand, if this mechanism can be exploited, carbon fixation in cyanobacteria could be increased.

Phosphoketolase (XPK) belongs to the transketolase family, a member of thiamine pyrophosphate (TPP)-dependent enzyme superfamily^12,13^. It converts F6P, xylulose-5-phosphate (Xu5P) or sedoheptulose-7-phosphate (S7P), which are intermediates in RuBP regeneration, to produce acetyl phosphate (AcP) and C3, C4 or C5 sugar phosphates (Fig. 1a). XPK is also a key enzyme in the synthetic non-oxidative glycolysis^14,15^ and has been exploited for engineering metabolism^16,17^; however, its physiological role in cyanobacteria is not fully understood. A previous report^18^ suggested that XPK in the cyanobacterium *Synechocystis* sp. is responsible for utilization of xylose or glucose under heterotrophic conditions. We have shown^19^ that the cyanobacterium *Synechococcus* *elongatus* PCC7942 XPK (*Se*XPK) is inhibited by ATP and participates in ATP production through the XPK–acetate kinase (ACK) pathway for long-term survival under anaerobic, dark and osmotic stress conditions. While deleting *Se*XPK did not affect growth under constant light or a 12 h/12 h light/dark (12-h L/D) cycle under aerobic conditions^19^, overexpression of *Se*XPK caused lethality. The role of XPK in carbon metabolisms and its ATP inhibitory mechanism are still puzzling in cyanobacteria under phototrophic growth conditions. In the CBB pathway, allosteric AMP inhibition of fructose-1,6/sedohaptulose-1.7-bisphosphatase has been reported in *Synechocystis* 6803 (ref. ^20^). In glycolysis, pyruvate kinase (Pyk) from *Synechococcus* PCC 6301 is inhibited by ATP^21^. Moreover, a CO_2_ fixing enzyme PEP carboxylase (PEPC) in *Synechocystis* is regulated by Pll sensor-transducer, which is allosterically modulated by adenyl nucleotides^22^. In the absence of Pll, PEPC is inhibited by ATP. The above studies elucidated the importance of allosteric enzyme regulations by adenyl nucleotides in metabolic pathways.

Considering that *Se*XPK is inhibited by ATP^19^, we propose that XPK may play a heretofore unrecognized role in dynamic metabolic regulation by diverting the RuBisCO substrate precursors away from the CBB cycle when ATP is low (Fig. 1a) to avoid futile cycling. If so, its function would be most pronounced under low-ATP conditions, such as during light fluctuations, and the molecular mechanism of ATP sensing by XPK would provide a useful insight. Furthermore, if XPK serves as a metabolic ‘brake’ for the CBB cycle, it could be deleted or inhibited to increase CO_2_ fixation, thereby increasing the net carbon draw down by cyanobacteria and even producing useful compounds.

## Results

### *Se*XPK functions as a CBB brake upon ATP drop

**Fig. 2:**
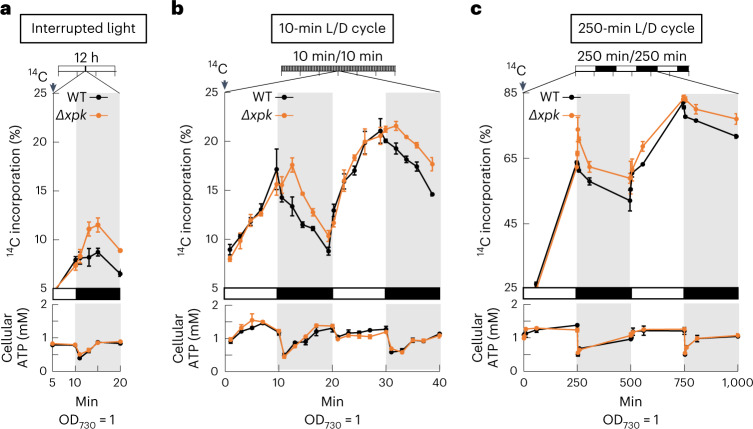
Delayed cessation of CO_2_ incorporation by *Δxpk* after light-to-dark switch. **a**, Three WT and the three *Δxpk* cultures were grown in BG11 medium containing 50 mM NaHCO_3_ under 12-h L/D cycles until the OD_730_ reached 1 (top). Then, during hours 5–6 in the light period, both cultures were refreshed with BG11 then supplied with 2 mM NaH^14^CO_3_, as time 0, under 50 μmol m^−2^ s^−1^ light. The light was switched off 10 min later. The ^14^C incorporation was analyzed for samples collected at 5, 10, 11, 13, 15 and 20 min. The ^14^C incorporation % (*y* axis) was calculated by ^14^C_cell_ / (^14^C_cell_ + ^14^C_medium_) (*n* = 3 biological repeats, mean ± s.e.m.). Under the same experimental conditions as above, WT and *Δxpk* cultures were supplied with 2 mM unlabeled NaHCO_3_ (bottom). Equal volumes of culture samples from each time point were collected and the ATP concentration was determined by a luminescence-based assay, as described in Methods (*n* = 3 biological repeats, mean ± s.e.m.). **b**, WT and *Δxpk* cultures were grown under 10-min L/D cycles then supplied with 10 mM NaH^14^CO_3_ at the end of the night, as time 0. **c**, Same as **b**, except for growing under 250-min L/D cycles. Source data

**Extended Data Fig. 1:**
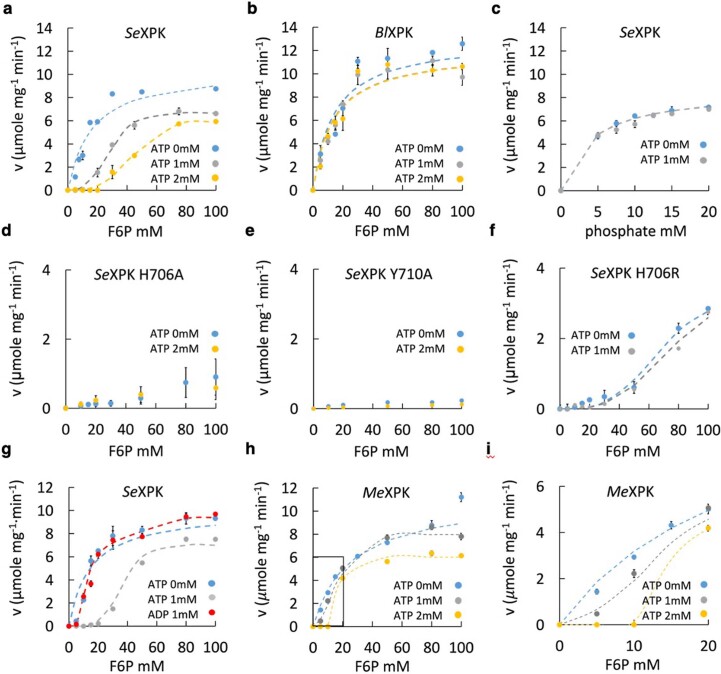
Steady-state kinetic analysis of XPK. **a**. ***Se*XPK** (V_max_ 10.8 ± 0.1 µmole mg^−1^ min^−1^; K_m_ 19 ± 1.5 mM) is inhibited by 1 or 2 mM of ATP, in the presence of 30 mM phosphate. **b**. Under the same condition, *B. longum* XPK (*Bl*XPK) displays no inhibition by ATP. **c**. *Se*XPK is not inhibited by phosphate substrate in the presence of saturating F6P (150 mM). **d**. H706A mutant of *Se*XPK lost most activity. **e**. Y710A mutant of *Se*XPK lost most activity. **f**. H706R mutant of *Se*XPK shows only minor effect by ATP. **g**. ADP does not affect *Se*XPK activity. The inhibition curve with ATP is included for comparison. **h**. *Methylomonas sp*. XPK (*Me*XPK) is also inhibited by ATP, though to a lesser extent than *Se*XPK. An enlarged view of the inset is shown in **i**. Data represent n = 3 individual reactions, mean ± SEM. Each plot represents one of the three experimental repeats. Source data

We first performed steady-state kinetic analysis, which suggested that *Se*XPK is inhibited in vitro by ATP through an allosteric mode (Fig. 1b,c), similar to *Cryptococcus* *neoformans* XPK reported previously^23^. On the other hand, *Bifidobacterium* *longum* XPK showed no inhibition by ATP (Extended Data Fig. 1 and Supplementary Table 1). Based on the kinetic data, the activity of *Se*XPK should be constantly inhibited when the intracellular ATP concentration is about 1 mM or greater; however, immediately after transition from light to darkness, ATP drops to 0.4 mM (Fig. 2), which should relieve the inhibition of *Se*XPK.

To investigate the role of XPK under the low-ATP conditions, we measured the carbon incorporation by the wild-type (WT) and XPK deletion (*Δxpk*) strains during the transition from light to dark. We grew these strains under 12-h L/D cycles until the optical density at 730 nm (OD_730_) reached 1. In the middle of the light period, NaH^14^CO_3_ was added to the cultures and carbon incorporation and intracellular ATP concentration were measured before and after switching to darkness. Results show that the carbon incorporation was similar between the WT and *Δxpk* strains in the presence of light (Fig. 2a); however, upon abrupt switching from light to dark, the carbon incorporation by the WT strain began to decrease, coinciding with the ATP drop. The net carbon incorporation continued to decrease, presumably due to carbon release in the dark period, whereas the intracellular ATP concentration recovered due to respiration. In contrast, the *Δxpk* strain continued incorporating CO_2_ for about 5 min in darkness, suggesting that the cell in the absence of *Se*XPK was not able to stop CBB activity immediately upon abrupt switching from light to dark.

To further validate this result, we evaluated carbon incorporation of the WT and *Δxpk* strains in two continuous L/D cycles at different frequencies, 10 min/10 min or 250 min/250 min L/D periods (Fig. 2b,c). In both cases, the carbon incorporation by the WT and the *Δxpk* strains were equal during the light phase. When entering the dark phase, the carbon incorporation was immediately reduced in the WT strain, but continued to increase in the *Δxpk* strain for several minutes, again coinciding with the ATP drop (Fig. 2b–d). The continued carbon uptake by the *Δxpk* strain in dark indicates the lack of CBB brake provided by XPK. On the other hand, the ATP level was not affected by the XPK deletion, suggesting that XPK’s primary role is the regulation of CBB cycle activity in response to the ATP level, but not the regulation of ATP level itself. Although XPK produces AcP, which leads to acetate and ATP production, the ATP production efficiency by respiration is much higher and dwarfs the contribution of XPK.

The CBB regulation by XPK is distinct from the well-known post-translational oscillator KaiABC, which controls various processes, including CBB cycle genes^8,24^. The regulation by XPK also complements a thioredoxin-modulated protein CP12, which forms an inactive ternary complex with glyceraldehyde-3-phosphate dehydrogenase (GAPDH) and phosphoribulokinase (PRK) to reduce carbon fixation in dark or oxidative conditions^11^. Deletion of CP12 in *S.* *elongatus* PCC7942 retards growth and reduces O_2_ consumption through respiration at night^25^. In contrast, *Se*XPK reduces carbon fixation by sensing a sudden drop of the ATP level (Fig. 2), but its deletion does not affect growth under normal conditions^19^ or influence O_2_ evolution in light and consumption in dark (Supplementary Table 2).

### *Se*XPK removes RuBisCO substrate precursors when ATP is low

**Fig. 3:**
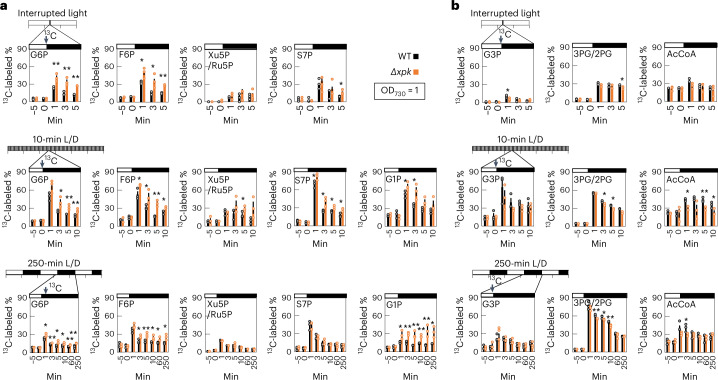
The increases of ^13^C-labeled RuBisCO substrate precursors in *Δxpk* after transition to darkness. Three WT and the three *Δxpk* cell cultures were grown under constant light (top) or different L/D conditions (middle and bottom) until OD_730_ reached 1. Equal amount of NaH^13^CO_3_ was added to the WT and *Δxpk* cultures in the middle of the light as 0 min (arrow) immediately before the light was turned off (black bar). **a**, XPK substrates (F6P, Xu5P and S7P) are RuBisCO substrate precursors. Sugar phosphates (G6P, G1P and Ru5P) are derived from F6P and Xu5P. **b**, XPK products and their derived compounds. G3P is a catalyzed product by XPK. 3PG, 2PG and AcCoA are intermediates and products in glycolysis. The ^13^C-labeled % was analyzed as the % of ^13^C / ^12^C. The ^13^C-labeled and unlabeled ^12^C-metabolites were analyzed by multiple reaction monitoring using LC–MS/MS, as described in Methods. The sum of the peak area from the ^13^C-labeled metabolite (^13^C) was divided by the total peak area consisting of the area from the unlabeled ^12^C metabolite (^12^C) and the sum area from labeled ^13^C. *n* = 3 biological repeats, mean ± s.e.m. Gray (WT) and orange (*Δxpk*) dots represented individual data points. Asterisk indicated significant difference between the WT and the *Δxpk* using one-sided statistical *t*-test via one-way analysis of variance (ANOVA). *P < 0.05 and **P < 0.01. Source data

To further investigate the function of *Se*XPK, we measured the levels of ^13^C-incorporated metabolites in the WT and *Δxpk* strains upon the light to dark switch for the three conditions in Fig. 2. NaH^13^CO_3_ was added immediately before light to dark switching and the levels of ^13^C-incorporated metabolites were measured. Results showed that ^13^C-labeled glucose-6-phosphate (G6P) and fructose-6-phosphate (F6P) were higher in the *Δxpk* strain relative to the WT in all three L/D conditions (Fig. 3a, with original data in Supplementary Fig. 1a). The ^13^C-labeled xylulose-5-phosphate (Xu5P)/ribulose-5-phosphate (Ru5P) and sedoheptulose-7-phosphate (S7P) levels were higher in the *Δxpk* strain under the interrupted light and 10-min L/D conditions. The levels of glucose-1-phosphate (G1P) were also higher for 10-min and 250-min L/D cycles, compared to the WT. In contrast, ^13^C-labeled glyceraldehyde-3-phosphate (G3P), 3-phosphoglycerate (3PG), 2-phosphoglycerate (2PG) and acetyl-CoA (AcCoA) were slightly less or not changed in the *Δxpk* strain compared to WT (Fig. 3b and Supplementary Fig. 1b). These data suggest that *Se*XPK in WT drained these precursors of the RuBisCO substrate to stop CO_2_ fixation after switching to dark. Without such a brake in the *Δxpk* strain, the cell continued to fix carbon using the remaining ATP and NADPH, while starting the tricarboxylic acid cycle, oxidative pentose pathway and respiration to generate ATP and reducing power. Simultaneously fixing CO_2_ and producing CO_2_ can form a futile cycle with a net drain of ATP, which can adversely affect cell physiology in general.

### Deletion of *Se*XPK increases net carbon fixation and enables sucrose production in high-density cultures

**Fig. 4:**
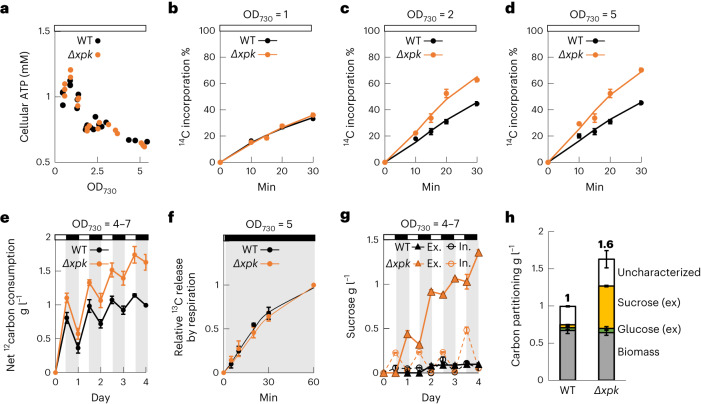
High-density culture of the *Δxpk* strain enhanced net carbon fixation resulting in sucrose secretion at night. **a**, Cellular ATP levels decreased at high cell density. Three WT and the three *Δxpk* cultures were grown under 12-h L/D cycles. Light intensity was 50 μmol m^−2^ s^−1^. The samples for ATP measurements were collected under light periods at different OD_730_. **b**, The carbon incorporation rates of the WT and the *Δxpk* at low cell density (OD_730_ = 1). **c**, The carbon incorporation rates of the WT and the *Δxpk* at D_730_ of 2. **d**, The carbon incorporation rates of the WT and the *Δxpk* at OD_730_ = 5. **e**, Net bicarbonate consumptions from the three WT and the three *Δxpk* cultures were measured within 4 d. 100 mM of NaHCO_3_ was added to the culture at day 0. Equal amounts from each culture were collected at the end of light (white) and at the end of dark (gray) and the remaining bicarbonate was converted to CO_2_ and measured. The net carbon fixation in the WT and in the *Δxpk* was 1 g l^−1^ and 1.6 g l^−1^, respectively after 4 d. **f**, ^13^CO_2_ was released from the WT and the *Δxpk* cultures into medium in dark. The cultures were fed with 100 mM NaH^13^CO_3_ for 2 d in 12-h L/D cycles then refreshed with BG11 before transition to dark. The carbon release at 60 min was set as 1 and the relative value at each time point is presented. **g**, *Δxpk* synthesized sucrose in the light and secreted sucrose in the dark. Open circles represent intracellular sucrose and closed triangles represent secreted sucrose. **h**, The carbon partitioning in biomass (gray), excreted (ex) sucrose (yellow), excreted glucose (green) and other uncharacterized molecules (white) after 4 d. *n* = 3 biological repeats, mean ± s.e.m. (**a**–**h**). Source data

In addition to L/D switching, we looked for other conditions that may reduce intracellular ATP levels. We found that the ATP level decreased when the cell density increased in a shaking culture (Fig. 4a), presumably due to self-shading^26^ and high-frequency light fluctuation. We then measured the ^14^C incorporation by the cells at different cell densities during the light phase in the 12-h L/D cycle. Notably, we found that the *Δxpk* strain incorporated more CO_2_ than WT even during the light phase as the cell density increased from 2 to 5 at OD_730_ (Fig. 4b–d). At OD_730_ = 5, the *Δxpk* strain fixed 63% more carbon than the WT strain (Fig. 4d), based on their slopes within 30 min. Thus, *Se*XPK in the WT strain can reduce carbon fixation by sensing low levels of ATP under self-shaded, light-fluctuating conditions.

Then we assessed the net carbon fixation of long-term, high-density WT and *Δxpk* cultures in a 12-h L/D cycles with the cell density from 4–7 at OD_730_. The *Δxpk* culture again showed increased bicarbonate consumption compared to the WT culture in the light periods. In the dark periods, approximately half of the pre-fixed carbon was released via respiration. At the end of the 4-d culture, the WT and *Δxpk* cultures respectively consumed 1 g l^−1^ and 1.6 g l^−1^ of carbon of bicarbonate and dissolved CO_2_ (Fig. 4e).

To investigate the effect of *Se*XPK deletion on the respiratory carbon release, we grew the WT and *Δxpk* cultures with NaH^13^CO_3_ for 2 d to label the cellular components with ^13^C, then washed away the residual NaH^13^CO_3_ in the medium and measured ^13^CO_2_ release from both cultures in darkness. The result (Fig. 4f) showed that the WT and *Δxpk* cultures released equal amounts of the pre-fixed ^13^C after switching to darkness, suggesting that deletion of *Se*XPK did not affect respiration. Hence, *Se*XPK functions as a CBB brake but is not involved in carbon release during dark respiration.

**Extended Data Fig. 2:**
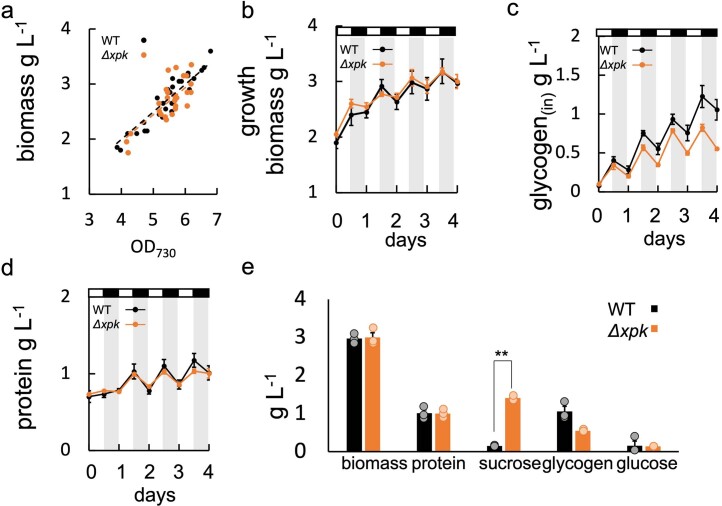
Deletion of *Se*XPK enhanced sucrose production but did not alter biomass. **a, b**. Biomass and the cell optical density (OD_730_) increase approximately equally in three WT and three *Δxpk* cultures in four days. **c**. Glycogen contents from equal volume of the three WT or the three *Δxpk* culture were measured in four days. **d**. protein content of the WT and the *Δxpk* in four days. **e**. The weights of biomass, protein, sucrose, glycogen and glucose were measured from the three WT and the three *Δxpk* cultures after four days. ** indicated the significant difference between the WT and the *Δxpk* in sucrose productions, using one-sided statistical *t*-test via one-way Anova. The *p* value = 3 × 10^−6^. **a**–**e**: n = 3 biological repeats, value = mean ± SEM. Source data

To determine the distribution of the fixed carbons, we measured the amounts of biomass and sugar contents of the WT and *Δxpk* strains during the 4-d culture (Fig. 4g,h and Extended Data Fig. 2). Notably, the increased carbon fixation by the *Δxpk* strain mostly resulted in sucrose secretion, which was synthesized during the light phase and excreted in the dark phase (Fig. 4g). At the end of 4 d, 1.3 g l^−1^ of sucrose was excreted from the *Δxpk* culture, which is tenfold higher than that from the WT culture. The amount of biomass and protein showed no difference between the two cultures (Extended Data Fig. 2b,d), whereas more sucrose but less glycogen was detected in the *Δxpk* culture (Fig. 4h and Extended Data Fig. 2c).

**Extended Data Fig. 3:**
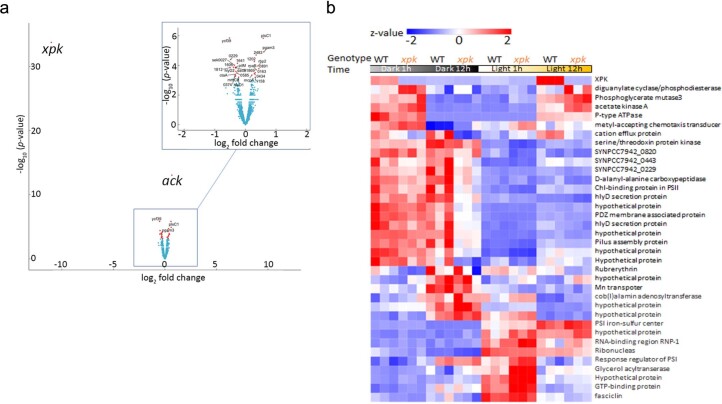
Transcriptional differences between WT and *Δxpk* across 4 time points. WT and *Δxpk* were grown under 12 h L/D cycle till OD_730_ reached 2. The samples were harvested 1 h after dark, at the end of dark, 1 h after light and at the end of light. Equal amount of RNA was extracted from both culture for RNA-seq analysis. **a**. Volcano plot between WT and *Δxpk* (n = 3 biological repeats, mean ± SEM). A few genes showed significant changes (red dots, p < 0.05). Their expressional fold change was less than 2 (x-axis, enlarged plot), except XPK and its downstream ACK. Thus, no global changes due to absence of XPK. The y axis represents FDR adjusted p-values, which are computed after first obtaining a two-sided Wald test p value by fitting expression data to a negative binominal Generalized Linear Model (GLM). **b**. The heat map showed the comparison of the 36 genes that appeared different in expression levels between WT and *Δxpk* (ANOVA-like statistical analysis by CLC) at 4 different time points. XPK was peaked at the end of light as an evening gene. However, the transcriptional differences are small as the z-values are ± 2. Source data

To determine whether the deletion of *Se*XPK caused any global gene expression change, we measured transcriptome profiles from the WT and *Δxpk* cultures with cell density of 2 at OD_730_, under 12-h L/D conditions. Our RNA-seq data showed only minor global transcriptome differences between WT and *Δxpk* (Extended Data Fig. 3a,b). In particular, the expression level of the cation efflux protein was lower in the *Δxpk* strain compared to the WT; however, its function is not yet characterized. Additionally, a few membrane-associated proteins were also downregulated in the *Δxpk* strain. Deletion of *Se*XPK did not alter the expression levels of carbon metabolic genes under the conditions examined. This result implied that *Se*XPK influences carbon fixation primarily at the metabolite level.

### *Se*XPK facilitates survival under high-frequency L/D cycles

**Fig. 5:**
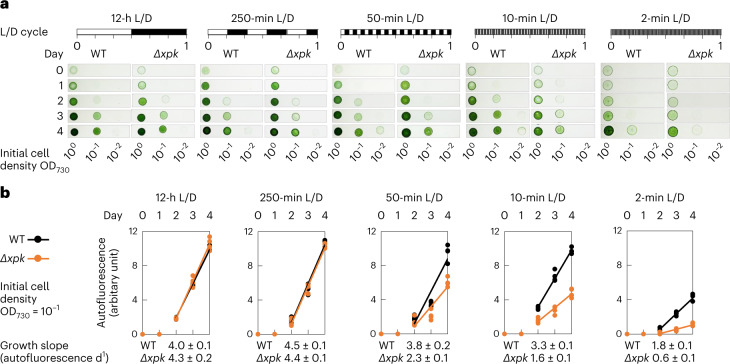
*Se*XPK functions as a regulator in rapid light–dark cycles. **a**, WT and *Δxpk* cells were first grown under continuous light for 2–3 d. Aliquots of both cultures were adjusted with culture medium to equal cell density with OD_730_ = 1. Both were subjected to tenfold serial dilutions (10^−1^, 10^−2^ and 10^−3^) and dotted on the BG11 medium containing agar plates for continuous growth under different frequencies of L/D cycles for 4 d. The 10^−3^ dilution culture dots did not grow and thus are not shown. **b**, Autofluorescence (*y* axis) of the dotted WT (black) and *Δxpk* (orange) cultures was monitored during the culture time (day, *x* axis). The fluorescence intensity for each cell dot from the 10^−1^ dilution was plotted and the growth slopes per day were calculated from the linear phase day 2 to day 4 (*n* = 4 biological repeats, ±s.e.m.). Source data

As the function of *Se*XPK was observed mainly during the L/D transitions, we evaluated whether *Se*XPK would have an impact on growth under different frequencies of L/D cycles. The diurnal (12-h L/D cycle) growth curves of the WT and *Δxpk* strains were very similar (Supplementary Fig. 2), in agreement with previous results^19^. Then we dotted equal amount of WT and *Δxpk* cells on BG11 agar plates and observed their growth at different frequencies of L/D cycle from 1 cycle per day (12-h L/D) to 360 cycles per day (2-min L/D). Imaging and autofluorescence measurements of the growing cell dots revealed that high-frequency L/D cycles reduced the growth rates of both the WT and *Δxpk* strains, but substantially more so for the latter (Fig. 5). These results suggest that XPK facilitates cyanobacteria survival in rapid L/D transitions. In control, no growth differences between the WT and *Δxpk* strains were observed under low-frequency L/D cycles. Thus, *Se*XPK’s function as a CBB cycle brake under low-ATP conditions is also important for cell survival in high-frequency L/D fluctuation.

### Unique ATP-binding motif in XPK

**Fig. 6:**
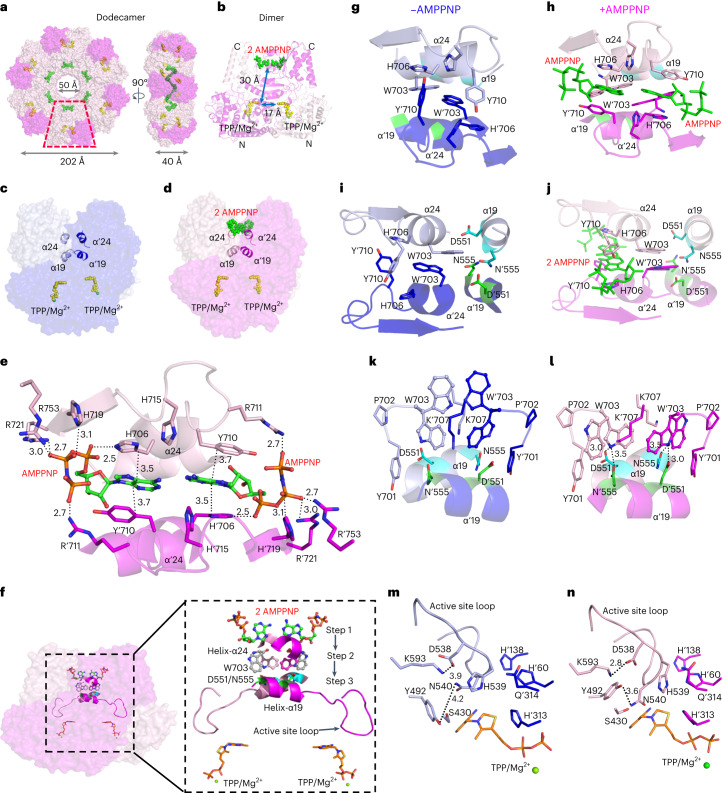
Structure and mechanism for the allosteric regulation of *Se*XPK by ATP. **a**, The dodecamer assembly of *Se*XPK in complex with 12 AMPPNP. **b**, Cross-section view of the XPK dimer unit showing 2 AMPPNP molecules (green spheres) bound to the dimer interface near the C terminus, whereas TPP/Mg^2+^ (yellow/green spheres) is located at the same interface but near the N terminus. **c**,**d**, A different view of the dimer interface for comparison between the free and complexed structures, respectively. **e**, Details of the ATP-binding site, showing that both AMPPNP molecules interact with residues from both subunits (pink and magenta colors). **f**, A bird’s eye view of the mechanism for allosteric inhibition in *Se*XPK. **g**–**n**, Stepwise illustration of the whole process of the allosteric regulation involving local conformational changes. Further explanation with a stereo views is provided in Extended Data Fig. 7. Primed residues designate residues from the reciprocal subunit in the dimer.

**Extended Data Fig. 4:**
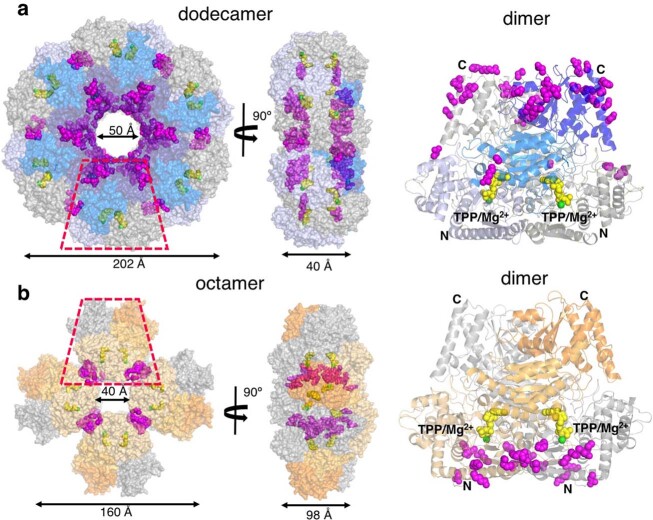
Cryo-EM structures of free forms of *Se*XPK and *B. longum* XPK. **a**. *Se*XPK dodecamer and its dimer unit, with its N-, middle, and C-subdomains colored in light blue, marine and dark blue in one subunit, while the other subunit is colored in gray for differentiation. The distribution of the residues (shown in magenta spheres) involved in the dimer-dimer interface of the dodecamer XPK are primarily located at the C-terminal subdomain. The coenzyme TPP/Mg^2+^ is shown in yellow/green spheres**. b**. *B. longum* XPK octamer and its dimer unit. The dimer is shown in orange and gray for each monomer. The residues involved in the dimer-dimer interface of octamer XPK are highlighted in magenta spheres. They are predominantly located at the N-terminal subdomain, in contrast to the *Se*XPK dodecamer.

**Extended Data Fig. 5:**
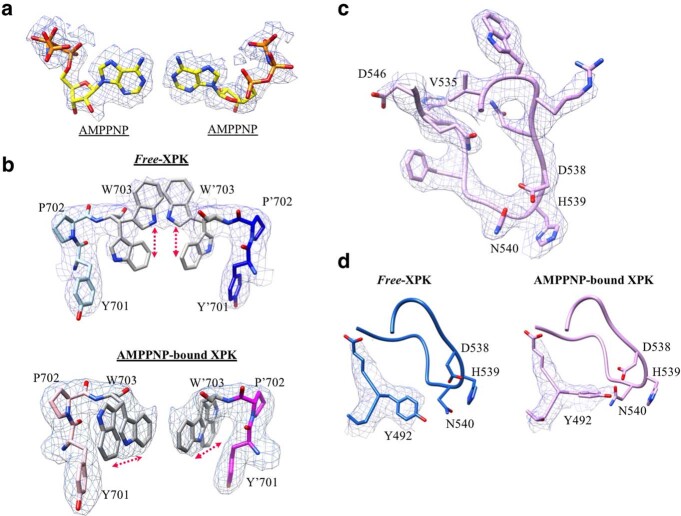
Representative cryo-EM density maps of the AMPPNP, and the key elements involved in the allosteric regulation of *Se*XPK. **a**. The representative electron density for AMPPNP modeling (residue from B subunit is labeled with a prime). A weak density blob was observed adjacent to the –PPNP moiety of AMPPNP, which appears to be too weak to be a magnesium ion. In addition, no carboxylate is in close distance (<3 Å). Therefore, it is tentatively assigned as a water molecule bridging His719 or His706 with the phosphate groups of the AMPPNP. **b**. The modeling of residues YPW in the free- and AMPPNP-bound XPK. In free XPK, the density can merely accomendate one W703 from either A or B subunit. Therefore, the two W703 residues are alternatively positioned downwards and upwards. Conversely, upon AMPPNP binding, the individual W703 can be modeled in two conformers, giving a dynamic swing-like motion. **c**. Cryo-EM density map for modeling the active site loop region (V534-S546) of AMPPNP-bound *Se*XPK. **d**. The electron density for modeling Y492 in free- and AMPPNP-bound XPK.

Given the importance of the physiological role of *Se*XPK, we solved the cryo-EM structures of *Se*XPK, both free (with coenzyme TPP/Mg^2+^ bound) and complexed with the non-hydrolyzable ATP analog, AMPPNP. In contrast to the published XPK structures from *Lactococcus* *lactis* and *B.* *breve/longum*, which exist as dimers in crystals^27–29^, the cryo-EM structure of *Se*XPK is a dodecamer composed of six dimeric units, forming a circular donut-shaped array both for the AMPPNP-bound form (Fig. 6a, Supplementary Fig. 4 and Supplementary Table 3) and in the absence of AMPPNP (Extended Data Fig. 4a, Supplementary Fig. 5 and Supplementary Table 3). The dimer is assembled face-to-face with two AMPPNP molecules residing head-to-head at the dimer interface in-between the C terminus of the subunits, approximately 30 Å away from the catalytic site of the respective subunit (Fig. 6b, with density maps in Extended Data Fig. 5a). Comparison between the free and bound dimers in the same orientation (Fig. 6c,d) shows that the two subunits jointly bind two AMPPNP. The key residues, H706 and Y710 from reciprocal subunits (helix α24 based on Supplementary Fig. 3), sandwich AMPPNP in the dimer interface by forming π–π stacking interactions with the adenine ring of AMPPNP. The α- and β-phosphate groups of AMPPNP are stabilized by H706, H719, R721 and R753 and the γ-phosphate group by R711 from the reciprocal subunit (Fig. 6e). Furthermore, unlike most other ATP-binding sites, there is no Asp or Glu within 5 Å of AMPPNP and no detectable divalent metal ion binding to the PPNP moiety. Instead, the negative charges of PPNP are mainly stabilized by the positive charges of the adjacent Arg and His residues (Fig. 6e).

Even though H706 and Y710 on two different subunits are the key residues for ATP binding, they are also key residues for maintaining the integrity of the dimer interface (Fig. 6e). Thus, H706A and Y710A lost most of their catalytic activities to nearly the limit of detection (Extended Data Fig. 1d,e). As the corresponding residue of H706 is Arg in the non-ATP-inhibited XPK from *B.* *adolescentis*^19^ and *B.* *longum* (Supplementary Fig. 3), we tested and confirmed that the XPK-H706R mutant was active but not inhibited by ATP (Extended Data Fig. 1f). Another notable residue involved in the binding of ATP is R711, which forms a salt bridge with the γ-phosphate of ATP and explains the lack of inhibition by ADP (Extended Data Fig. 1g). The results demonstrate the power of cryo-EM in enzymology^30^.

**Extended Data Fig. 6:**
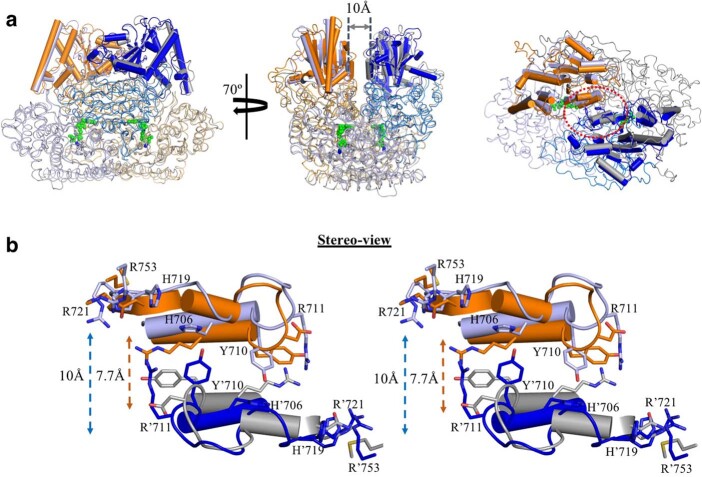
Structural comparison of the C-terminal domain interface in *S. elongatus* and *B. longum* XPKs in the free form. **a**. Left: structural superimposition of the dimer form between *S. elongatus* (dark/light blue) and *B. longum* (orange/gray) with RMSD 1.046 Å from 1291 Cα atoms of the respective dimer shows the C-terminal domain (in cartoon cylindrical presentation) interface of the subunits, mainly constituted of two helices from individual subunits. Middle: the approximate distance between the helices is 10 Å in *S. elongatus*, wider than 7.7 Å measured in *B. longum* XPK. Right: the view of the interface from above (circled with red dash lines). **b**. Stereo view showing the different residues and distances at the ATP-binding interface in the two XPKs. In addition to the narrower space between the helices compared to the *S. elongatus*, *B. longum* XPK has an Arg replacing H706 of *Se*XPK, Asn replacing R721, and Met replacing R753. Therefore, *B. longum* is unfavorable for ATP binding.

For comparison, we also solved the cryo-EM structure of *B.* *longum* XPK and found it to be an octamer, resembling the recently reported structure with calcium instead of magnesium ions^31^ (Extended Data Fig. 4b, Supplementary Fig. 6 and Supplementary Table 3). Of note, while the dimer–dimer interface in the *Se*XPK dodecamer involves residues near the C terminus, the *B.* *longum* XPK octamer involves residues near the N terminus. Comparison between the C-terminal interfaces of free *Se*XPK and free *B.* *longum* XPK structures provides a structural basis for the lack of ATP inhibition in *B.* *longum* XPK (Extended Data Fig. 6).

As the donut-shape or four-leaf clover shape-like particles or two-dimensional (2D) class average images were not in perfect symmetry (Supplementary Figs. 4b, 5b and 6b), we further examined whether the assigned symmetries were pseudosymmetry. As described in Methods and Supplementary Fig. 7, some relative random motions were observed between different dimers in both *Se*XPK dodecamer and *B.* *longum* octamer assemblies, suggesting the likelihood of pseudosymmetry in these structures.

Structure-based searches of the Protein Data Bank (PDB)^32^ were performed to determine whether there is any similar ATP-binding mode in 5,933 protein structures complexed with ATP, ADP, AMP or analogs. The search criteria are defined in Extended Data Fig. 8a. The PDB structures meeting the requirement of the atomic distances are listed in Source Data File 1 (226 structures). Most of these structures, however, have both aromatic residues from the same polypeptide chain. Only three structures consist of two aromatic residues from different subunits of a dimer positioned nearby the adenine ring (Extended Data Fig. 8b–d). For all of these, only one aromatic residue forms ring stacking with the adenine ring of the nucleotide and none has an additional Arg nearby. Therefore, no structure matches the full criteria, supporting the uniqueness of the ATP-binding mode.

**Extended Data Fig. 7:**
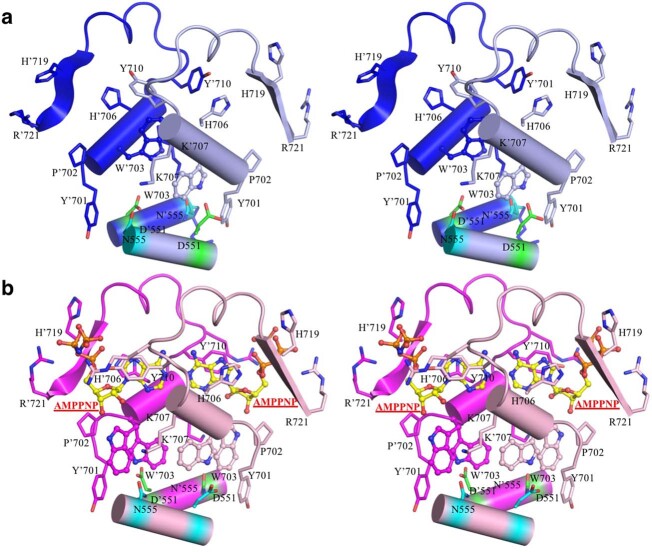
Stereo view of the expanded views of the ATP allosteric site of *Se*XPK. **a**. The free state, providing the combined views of Fig.3g,i,k. **b**. Bound with AMPPNP, providing combined views of Fig. 3h,j,l. Comparison between the free and bound structures here and in the main text provides detailed information on the effect of ATP binding, for all 8 residues in the conserved motif P702, W703, H706, Y’710, R’711, H719, R721, R753 as well as the key helices α24 and α19. Since the main text focuses more on the interaction with α24, here we provide detailed explanation for the interaction with α24, which also involves dynamic features of W703 (density maps in Extended Data Fig. 5b). P702 resides at a turn connecting the helix α24 and the loop where W703 and Y701 respectively reside. Therefore, it provides certain degree of flexibility to the local region. In the free XPK, the W703 from one subunit can reside at two positions, swinging upwards and downwards, but alternates with the other W703. When the W703 side chain swings upwards, it partially occupies the ATP binging space. In the AMPPNP-bound structure, both W703 residues swing downwards and show subtle side chain movement (side-to-side), which is stabilized by the cation-pi interaction with K707 and an H-bond between C=O of Y701 and NH of W703. Such interaction is also likely facilitated by P702. The three residues form a beta-turn like configuration to keep W703 side chain pointing downwards and interact with D551 or N555 from helix α19. In addition, the dynamic feature of W703, assisted by its neighboring residue P702, could function as a regulatory switch in response to ATP binding.

**Extended Data Fig. 8:**
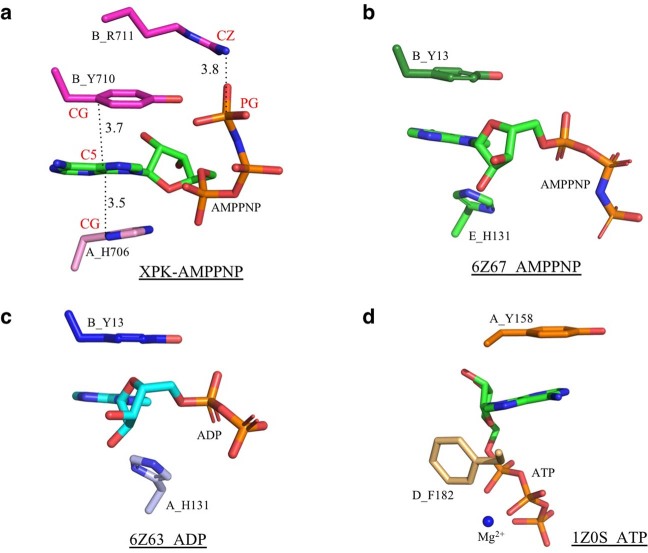
Structure-based PDB searches. **a**. The PDB search criteria: (1) The distances (Å) between the atom C5 of the adenine ring and the atom CG of the Y710/H706 side chain. The tyrosine and histidine are from different subunits. (2) The distance between the phosphorous atom PG of the gamma-phosphate group and the atom CZ of a neighboring arginine. These distances were relaxed to ≤5 Å in the search. The two subunits of a dimer are designated as A and B. **b–d**. Nucleotide binding modes of the three closest search hits. The cases (**b**, **c**) are the same protein crystalized with ADP and AMPPNP^28^. In all three cases^28,29^, the nucleotide binds to the dimer interface, and interacts with the aromatic residues from different protein chains of the dimer. However, only one aromatic residue forms base stacking with the adenine ring. Details of the search data are shown in Source Data File 1. Source data

The root-mean-square deviation between the structures with and without AMPPNP-bound is very small: (0.42 Å from 7,897 Cα atoms of the dodecamer and 0.36 Å from 1,256 Cα atoms of the dimer). Nonetheless, ATP binding to the allosteric site could cause conformational change of active site residues through a pathway of local conformational changes involving three structurally observable steps, as summarized in Fig. 6f and detailed in Fig. 6g–n and Extended Data Fig. 5b–d, with further explanation by stereo views in Extended Data Fig. 7 showing important roles of P702–W703 in these conformational steps. In step 1, ATP binding affects side-chain conformations of residues in helix α24 as explained above (Fig. 6g–j; in two different views). In step 2, the W703 side chain from both subunits wobble in two alternative positions and form an H-bond interaction with D551 from the same subunit or N555 from the other subunit; both are highly conserved residues from helix α19. Also, W703 forms a cation-pi interaction with K707 (Fig. 6i–l). In step 3, these interactions lead to closer contact of the active site loop with the adjacent conserved catalytic residues such as Y492–N540 and K593–D538 interactions (Fig. 6m,n), which could impede substrate binding.

### Presence of the ATP-regulatory motif in all three domains of life

**Fig. 7:**
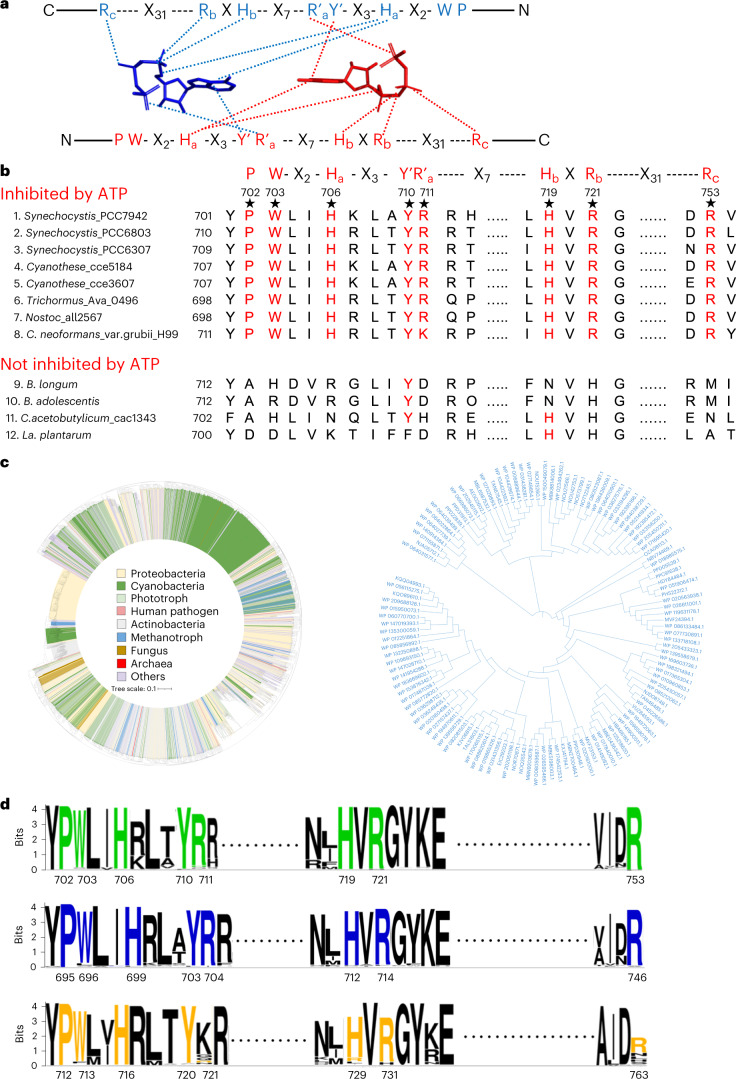
Proposed ATP-regulatory motif and its occurrence in diverse microorganisms. **a**, Schematic illustration of how two conserved motifs (red and blue) jointly interact with two ATP molecules (red and blue, respectively). Primed residues cross-interact with the reciprocal ATP molecule. **b**, The conservation of the structurally characterized ATP-regulatory motif (labeled in red) among phosphoketolases that have been biochemically characterized to be inhibited (1–8) or uninhibited (9–12) by ATP^19,23,33^. **c**, Construction of a neighbor-joining tree for the 3,190 ATP-regulatory motif-containing protein sequences (with all eight motif residues identical, except that R_a_ is allowed to be K also for fungus due to reported data in row 8). The NCBI accession nos. of all sequences are in Source Data File 2. The subset of methanotroph is shown here and some other subsets are shown in Supplementary Fig. 9. **d**, Comparison of sequence logos from randomly chosen 1,377 cyanobacterial sequences (NCBI taxid, 1117), 176 methanotroph sequences (NCBI taxid, 135618) and 1,194 fungus sequences (NCBI taxid, 33154) for the eight conserved residues in the ATP-regulatory motif along with their surrounding residues. The motif residues are shown in green, blue and orange colors, respectively. Residue numbering is based on *Se*XPK, *Methylomonas* sp. DH-1 and *C.* *neoformans* var. grubii H99, respectively. The logos were constructed by use of the WebLogo 3 web server^70^. Source data.

The above structural and functional analyses identified eight important residues (P702, W703, H706, Y710, R711, H719, R721 and R753) for the allosteric inhibition of *Se*XPK by ATP (Fig. 6e and Extended Data Fig. 7). These residues are conserved in other XPKs reported to be inhibited by ATP^19,23^, leading to a conserved motif PWX_2_H_a_X_3_Y’R’_a_X_7_H_b_XR_b_X_31_R_c_, where a,b,c designate histidine or arginine at different positions and primed residues cross-interact with the reciprocal ATP molecule as illustrated in Fig. 7a. This representation is equivalent to Fig. 6e, where each ATP molecule interacts with residues from both unprimed and primed subunits. In support, the XPK variants not inhibited by ATP from this work and previous reports^19,33^ are not conserved at these positions (Fig. 7b, rows 9–12).

**Extended Data Fig. 9:**
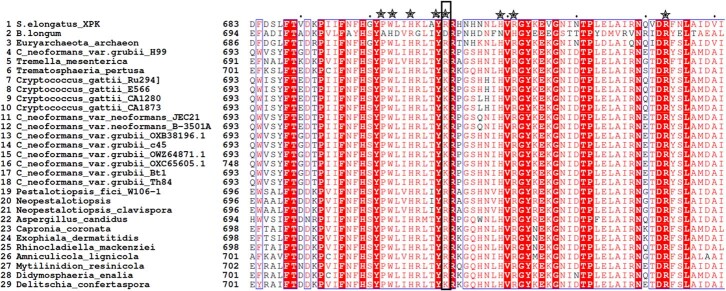
Sequence alignment of phosphoketolase from the non-bacteria groups. These include Fungi species (no. 4-no. 29) and one Archaea (no. 3) identified in our NCBI protein sequence search. The residue locations of the ATP-regulatory motif (**PW**X_2_**H**_**a**_X_3_**Y’R’**_**a**_X_7_**H**_**b**_X**R**_**b**_X_31_**R**_**c**_) in the sequence are marked with stars. The bracket shows that K is as prevalent as R when included in the search for fungi species, as also included in Source Data File 2. The *C. neoformans_var.grubii_H99* XPK (with a K in the R’_a_ position) was shown to be allosterically inhibited by ATP^23^. The accession code of the individual sequence: **1**. WP_011244323.1; **2**. CBK70695.1; **3**. TMA00651.1; **4**. XP_012048352.1; **5**. XP_007002465.1; **6**. KIR51125.1; **7**. KIY30858.1; **8**. KIR44508.1; **9**. KIR57564.1; **10**. XP_5657776.1; **11**. XP_772632.1; **12**. OXB38196.1; **13**. OWZ52886.1; **14**. OWZ64871.1; **15**. OXC65605.1; **16**. OWT40626.1; **17**. OXG51936.1; **18**. XP_007841480.1; **19**. XP_007841480.1; **20**. KAF7515066; **21**. XP_024673765.1**; 22**. XP_007722306.1; **23**. XP_009159227.1; **24**. XP_013267806.1; **25**. KAF2007887.1; **26**. XP_033675732.1**; 27**. XP_033574715.1; **28**. KAF2267372.1; **29**. KAF2205034.1. Source data

Next, we investigated the prevalence of this motif by NCBI BLAST in a non-redundant protein database^34^. From the 4,930 phosphoketolase sequences with >60% sequence identity to the *Se*XPK, 3,175 sequences showed all eight residues identical (Fig. 7c and Source Data File 2). A subset of these sequences with ≥70% identity is shown in Supplementary Fig. 8. These phosphoketolase sequences predominantly belong to cyanobacteria, proteobacteria and actinobacteria phyla. As expected, they include a number of aquatic cyanobacteria, including marine species such as *Synechococcus*, *Oscillatoria*, *Anabaena*, *Okeania* and *Acaryochloris*. Among the proteobacteria phylum, the major groups are phototrophic and methanotrophic bacteria, including *Methylomonas* sp., known for bioengineering applications^35,36^. The remaining sequences came from a wide variety of species, including sulfur-oxidizing autotrophic bacteria, acidobacteria, chemolithoautotrophic bacteria; human pathogens *Spirochaetes*, *Parachlamydiaceae* and *Cryptococcus* (fungi); plant pathogen *Pestalotiopsis* (fungi); and *Euryarchaeota archaeon* (Extended Data Fig. 9 and Source Data File 2). The actual number of species could be larger if conserved residues in addition to identical residues are included, such as replacing the R_a_ residue with a lysine (Extended Data Fig. 9).

To compare the relative abundance of the sequences containing the ATP-regulatory motif between different species, we then constructed sequence logos from sets of sequences without pre-selection for cyanobacteria, methanotroph and fungus, for the eight motif residues and their surrounding ones (Fig. 7d). The results suggest that the regulatory motif is highly abundant in cyanobacteria and methanotroph and substantially less so in fungi. Further structural and functional analyses of other systems are highly warranted.

Notably, the ATP-binding motif also exists in methanotrophs and methylotrophs, which do not have the CBB cycle but use the ribulose monophosphate (RuMP) cycle^37^ to assimilate C1 compounds, including methane, methanol and formaldehyde. To test this motif prediction, we cloned a methylotrophic *Methylomonas* sp. XPK in *E.* *coli* and overexpressed and purified the protein. Indeed, its allosteric inhibition by ATP was validated based on steady-state kinetics (Extended Data Fig. 1h,i). As RuMP and CBB cycles share many common enzymes and metabolites, including the XPK substrates Xu5P, F6P and S7P, it is conceivable that XPK also serves as a metabolic brake in response to the ATP level to regulate the RuMP cycle. Indeed, as methane and methanol in the environment are scarce and their supply may be intermittent, an XPK control of its assimilation based on its energy level may be of importance.

## Discussion

**Fig. 8:**
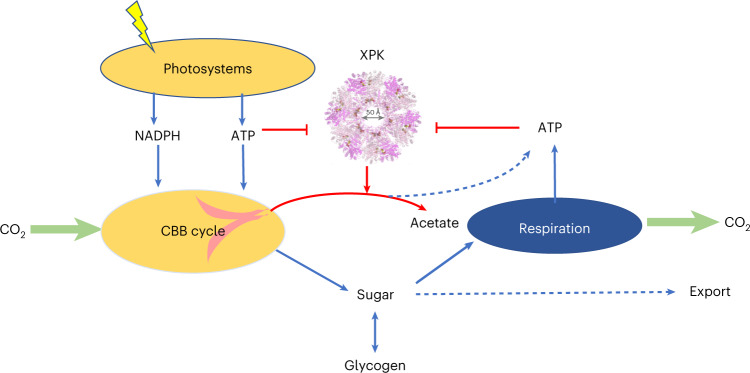
XPK serves as a CBB cycle brake under low ATP conditions. Under normal ATP conditions (>1 mM), which occur at sufficient light or during dark periods when respiration is fully functional and CBB stops due to the lack of reducing power, XPK is inhibited and serves no function; however, during sudden environmental changes (for example self-shading and high-frequency light fluctuation), the ATP level drops and XPK is relieved from inhibition. XPK then diverts the CBB intermediate to stop CO_2_ fixation and produces AcP, which is then converted to acetate and ATP.

**Extended Data Fig. 10:**
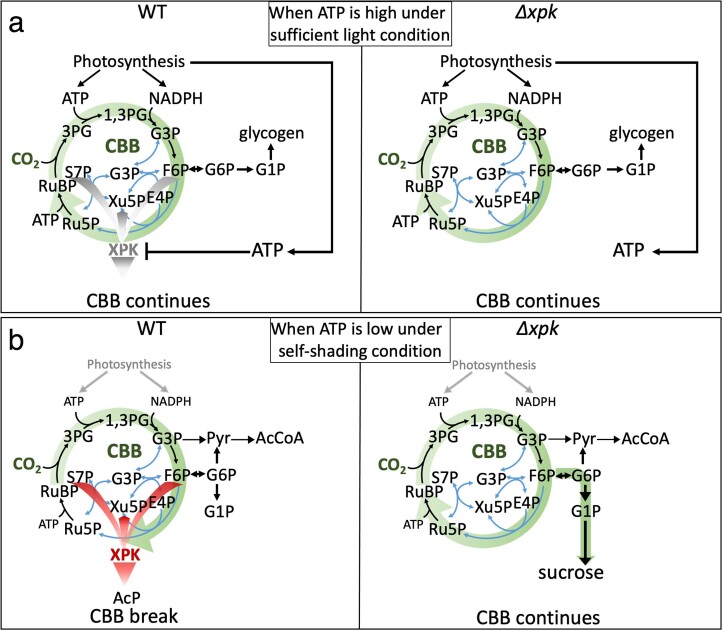
Scheme of WT and *Δxpk* under high ATP or low ATP condition. **a**. When light-driving ATP is sufficient, *Se*XPK is inhibited in WT and carbon fixation via CBB is continued. Deletion of SeXPK has no effect. The fixed carbons are stored as glycogen. **b**. When ATP level is low under self-shaded condition, XPK in WT metabolizes sugar phosphates to interrupt carbon fixation. In *Δxpk*, CO_2_ is continuously incorporated via CBB to form hexose-phosphates, followed by sucrose synthesis and excretion.

XPK is an enzyme present in many microorganisms, primarily involved in substrate-level phosphorylation to produce AcP. Cyanobacterial XPK, however, evolved to acquire a unique mechanism to bind to ATP at the interface between two subunits through its N-terminal residues. The binding leads to a conformational change of active site residues to inhibit its activity. With this ATP-sensing activity, *Se*XPK stops carbon fixation when ATP levels are low, by diverting CBB cycle intermediates to AcP, which may proceed to produce acetate and ATP (Fig. 8 and Extended Data Fig. 10). Typically, low ATP levels occur at the transition between L/D periods. Alternatively, during high-frequency light fluctuation caused by self-shading or ocean vertical currents, ATP levels could also drop. Under these conditions, if both CBB cycle and respiration are functional, the simultaneous operation of both leads to a futile cycle with a net effect of draining ATP. *Se*XPK evolved to avoid this wasteful activity and represents a distinct mechanism to control carbon fixation through CBB. Thus, *Se*XPK facilitates cell survival under high-frequency light fluctuations.

The regulatory role of *Se*XPK is not essential under constant light and 12-h L/D cycle conditions. Thus, deletion of *Se*XPK does not affect growth; however, in a high-cell-density culture with self-shading, deletion of *Se*XPK increased net carbon fixation, due to the lack of the CBB cycle brake. Notably, the increased carbon fixation flux primarily results in sucrose secretion. Thus, *Se*XPK interrupts carbon fixation and prevents sucrose excretion in response to low ATP; however, the link between *Se*XPK deletion and sucrose secretion remains unclear.

Unlike introducing synthetic pathways or overexpressing CBB pathway genes that increase carbon fixation, deletion of XPK achieves these goals without additional gene modifications^38–40^. In contrast to most studies on enhancing photosynthetic carbon assimilation or reducing respiratory mechanisms, it is rare that deletion of a single gene elevates net carbon fixation toward sugar production^41,42^. The deletion of *Se*XPK can be combined with other metabolic engineering strategies to increase the production of sucrose or other products. Another example has been discovered in *Synechocystis* *Δeda* mutant whose 2-keto-3-deoxygluconate-6-phosphate (KDPG) aldolase is lacked in the Entner–Doudoroff (ED) pathway. KDPG cannot be converted to pyruvate and glyceraldehyde-3-phosphate but enhances CBB flux and glycogen formation^43^. Sucrose production in cyanobacteria is used as osmoprotective compound to balance the osmotic potential between cell interior and exterior^44^. Previously, by adding salt to a sucrose-secreting strain expressing *cscB*, a heterologous sucrose/proton symporter, in *S.* *elongatus* PCC7942, sucrose production was dramatically increased up to 0.9 g l^−1^ a day^45^. Additionally, overexpressing sucrose phosphate synthase (SPS) in *S.* *elongatus* UTEX2973 produced 1.1 g l^−1^ sucrose a day without salt stimulation^46^. The *xpk* deletion enables sucrose synthesis, probably due to its increase in the substrate levels for SPS (Fig. 3 and Extended Data Fig. 2). G1P is the precursor for UDP/ADP glucose formation, then SPS converts UDP/ADP glucose and F6P to sucrose phosphate. Subsequently, sucrose is formed via sucrose phosphate phosphatase. Of note, the *Δxpk* strain synthesized more sucrose but accumulated less glycogen in *Δxpk* (Fig. 4 and Extended Data Fig. 2c). Unlike sucrose synthesis, which can proceed with UTP, glycogen synthesis requires ATP to form the glucan chain. In the WT strain, XPK functions as a CBB brake under low ATP levels. When ATP is again available, carbon fixation resumes immediately. In the *Δxpk* strain, carbon fixation via CBB continues even under low ATP levels and produces excess hexophosphates, which are then converted to sucrose and secreted. As CBB continues in the *Δxpk* strain, glycogen breaks down to G1P, followed by phosphoglucoisomerase (PGI) and ED pathway to replenish CBB intermediates^47^. This may explain the lower glycogen amount in the *Δxpk* strain (Extended Data Fig. 2c). While glycogen and sucrose can both store energy in the cell, storage of sucrose increases osmotic stress. As a result, sucrose is secreted from the *Δxpk* strain. Hence, the role of XPK is to prevent two wasteful processes under low ATP conditions: futile cycling between carbon fixation and respiration and sucrose secretion.

The secretion of sucrose by *Δxpk* in the dark without osmotic stress broadens the conditions from our previous results^19^. Cyanobacteria have capability for exporting compounds such as saccharides, acetate, lactate and toxic metabolites^48^. According to the membrane transporter database TransportDB2.0 (ref. ^49^), *S.* *elongatus PCC7942* contains ten major facilitator superfamily protein transporters and some of them may participate in multidrug and sugar efflux. These may be responsible for sucrose secretion; however, further investigation is required to understand the mechanism involved in sugar secretion.

Overall, these findings suggest that the ATP allosteric regulation of XPK, with a newly identified binding site that is widely present in nature, provides a sensitive metabolic brake to C1 carbon assimilation cycles in response to ATP shortage due to sudden environmental changes and is particularly important for marine cyanobacteria as the cells are constantly subject to intermittent supply of light. As the CBB cycle shares many steps with the RuMP cycle in methylotrophs, it is conceivable that the same ATP-sensing mechanism is used to control methane and methanol assimilation in these organisms (Extended Data Fig. 1h,i). How this mechanism can be exploited to increase C1 assimilation presents an interesting topic for further studies.

## Methods

### Recombinant protein expression and purification

*S.* *elongatus* PCC7942 XPK (Synpcc7942_2080) was constructed under a pDC vector harboring a *lac* promoter and its N terminus was in-framed with a 6His-tag for nickel column purification. H706R, H710A and Y710A *S.* *elongatus* mutants were generated via QuickChange II Site-Directed Mutagenesis kit (Agilent). *B.* *longum* XPK (BIL_11880) and *Methylomonas* sp. DH-1 XPK (AMY39_18755) were also constructed by using the above plasmid. *Se*XPK, its mutant variants and *B.* *longum* XPK were individually transformed in *E.* *coli* DH5αDE3.1 strain. Recombinant XPK proteins were expressed by adding 1 mM IPTG in 5 l LB culture supplied with 250 µg ml^−1^ spectinomycin and incubated in 30 °C, 150 r.p.m., for 16–20 h. The purification procedure followed that of Suzuki et al.^50^ with some modifications. The cell pellets were resuspended in buffer A, containing 20 mM HEPES–NaOH (pH 7.2), 0.2 mM thiamine pyrophosphate (TPP), 1 mM MgCl_2_ and cOmplete protease inhibitor cocktail tablets (Roche). The suspended cells were disrupted with a homogenizer (Avestin EmulsiFlex-C3), then centrifuged at 20,000 r.p.m., 4 °C for 30 min to remove cell debris. The supernatant was applied to an open column stuffed with cOmplete His-Tag purification resin (Roche). The resin was equilibrated with buffer A before loaded with protein sample. The sample-loaded resin was subjected to washing steps with buffer A supplemented with 10 mM imidazole and increased concentration of NaCl (from 0.1 M, 0.4 M to 1 M NaCl). Before protein elution, the resin was further washed with buffer A to reduce the salt content and was subsequently washed with buffer A supplemented with 0.25 M imidazole to elute proteins. The eluted protein sample was concentrated and loaded into a 5-ml Q Sepharose column (GE Healthcare), pre-equilibrated with buffer A. The protein sample was eluted with a NaCl gradient in buffer A supplemented with 1 M NaCl. The collected fraction sample was applied to a HiLoad 16/600 SuperdexTM 200 pg column (GE Healthcare) pre-equilibrated with a buffer containing 20 mM HEPES–NaOH (pH 7.2), 0.1 M NaCl, 0.2 mM TPP and 1 mM MgCl_2_. The eluted protein was examined with SDS–PAGE for purity as well its identity with mass spectrometry.

### Kinetic assay

XPK enzymatic velocity was determined by measuring the formation of AcP using a colorimetric method^14^. The 50-µl enzymatic reaction solutions contained 30 mM sodium phosphate (pH 6.4), 1 mM TPP, 1 mM MgCl_2_ and different concentrations of fructose-6-phosphate (F6P) or sodium phosphate (Pi). The reaction began by adding 8 µg of purified XPK. A total of 40 µl was taken from each reaction at serial time points (30 s to 2 h) and the reaction was stopped by adding 60 µl 2 M hydroxylamine (pH 6.5) in a 96-well plate. After incubating for 10 min at room temperature, the coloring reagents, including 40 µl 15% trichloroacetic acid, 40 µl 4 M HCl and 40 µl 3% FeCl_3_ in 0.1 M HCl were added sequentially. Absorbance at 505 nm was then recorded on a plate reader. A standard curve using commercial lithium potassium AcP was used to relate absorbance to concentration, which showed a linear relationship between 0.3125 to 100 mM in the conditions described. The OD_505_ values were converted to the product AcP in µmol and used to establish kinetic curves (Fig. 1 and Extended Data Fig. 1) and calculate the initial velocity (µmol AcP mg XPK^−1^ min^−1^ or simply µmol mg^−1^ min^−1^). *V*_max_ and *K*_m_ values were determined by varying the concentration of one substrate with another substrate held constant at a saturating level (30 mM Pi or 150 mM F6P) (Extended Data Fig. 1 and Supplementary Table 1).

### Cyanobacteria strains and growth condition

*Synechococcus* *elongatus* PCC7942 WT and the *Δxpk* strain^19^ were respectively grown in 50 ml BG11 medium^51^, pH 8, supplemented with 50 mM NaHCO_3_, under a 12-h L/D cycle. The incubation conditions were 30 °C, light intensity 50 µmol m^−2^ s^−1^ and shaking speed at 150 r.p.m. Gentamycin (10 µg ml^−1^) was added to the *Δxpk* culture to ensure the growth of the *xpk* knockout genotype. The cell growth was monitored at an optical density of 730 nm (OD_730_). The average dry-cell weight of 384 mg l^−1^ was calibrated for the culture with an OD_730_ of 1 used for experimental calculation.

### ^14^C incorporation experiment

For the ^14^C incorporation experiments (Fig. 2a), WT and *Δxpk* cultures (triplicates for individual culture) were grown in the BG11 medium containing 50 mM NaHCO_3_, under a 12-h L/D cycle (light intensity, 50 µmol m^−2^ s^−1^; 30 °C; shaking speed, 150 r.p.m.). Then, after 5–6 h during the light period, the cultures were respectively refreshed with BG11. Then, 0.8 ml from individual culture (OD_730_ = 1) was transferred to a 24-well Petri dish under light. The ^14^C-labeling was initiated (time 0) by adding 0.2 ml 10 mM NaH^14^CO_3_ (containing 113 µCi of total ^14^C) to the cultures. After 10 min, the light was switched off. Then, the cultures were collected at the time points (under darkness): 5, 10, 11, 13, 15 and 20 min and further subjected to the ^14^C incorporation analyses^51^. Then, 40 µl of each labeled culture was transferred to Costar Spin-X centrifuge tube filters, spun at full speed for 10 s to separate cells from the medium. The cell pellet was immediately resuspended with 200 µl 0.1 M NaOH, then transferred to a scintillation vial. The cell-free medium (~40 µl) was supplemented with 160 µl BG11 and transferred to another vial. The Ultima Gold scintillation cocktail (5 ml) was then added to each vial for liquid scintillation counting analyses. The ^14^C incorporation % (*y* axis) was calculated by ^14^C_cell _/ (^14^C_cell_ + ^14^C_medium_) × 100 (*n* = 3, mean ± s.e.m.). For the experiments shown in Fig. 2b,c, the WT and *Δxpk* cultures (triplicate batches) were grown under a 10-min L/D cycle or 250-min L/D cycle. The cultures were refreshed with BG11 and adjusted to the cell density (OD_730_ = 1) in the dark. Then, 0.8 ml starting culture (OD_730_ = 1) was added with 0.2 ml 50 mM NaH^14^CO_3_ upon switching to light (set as time 0 min). The following samples were collected at different time points from two continuous L/D cycles. To analyze the ^14^C incorporation at the high cell density, (Fig. 4b–d), the WT and *Δxpk* cultures (triplicate batches) were grown under a 12-h L/D cycle until the OD_730_ reached to 1 or 2. The cultures were refreshed with BG11 and adjusted OD_730_ to 1, 2 or 5. After 5–6 h illumination, an 0.8-ml culture (OD_730_ = 1, 2 or 5) was added with 0.2 ml 10 mM NaH^14^CO_3_ (set as time 0 min).

### ATP measurements

The following procedure was based on Rust et al.^52^ with modifications. A total of 0.4 ml WT and *Δxpk* cultures (triplicate batches for both) were transferred to 1.5-ml tubes containing 0.14 ml 3 M HClO_4_ and 77 mM EDTA. The mixture was then frozen with liquid nitrogen to stop the reactions and lyse cells. Next, the samples were defrosted and neutralized with 0.26 ml solution, containing 1 M KOH, 0.5 M Tris and 0.5 M KCl, to adjust the pH to 7. After 14,000*g* centrifugation at 4 °C for 30 min, the supernatant was collected for ATP measurements. Then, 38 µl supernatant was diluted with 62 µl buffer containing 100 mM HEPES, pH 7.4, 25 mM KCl and 50 mM MgSO_4_. To plot the ATP standard curve, 1 nM to 1 µM ATP solutions were prepared in parallel. The 100 µl sample and ATP solutions were transferred to an opaque 96-well plate and 100 µl luciferase-based BacTiter-Glo assay solution was added to each sample well. The luminescence signals from each reaction and standard were measured and then the ATP amount was quantified as showed in Figs. 2 and 4a.

### The measurement of O_2_ evolution and consumption

The amount of O_2_ evolution and consumption in cells was analyzed using a Clark-type oxygen electrode along with a water-jacket cell^53^. The cell cultures (grown under light intensity of 50 μmol m^2^ s^−1^) containing 10 μg *Chla* were mixed with 2 mM potassium ferricyanide and 2,6-dichloro-p-benzoquinone (an artificial electron acceptor) in a 1-ml reaction. The reaction mixture was then placed in a stirred water-jacket cell under dark. The O_2_ consumption of the cell culture was quantified by measuring the decreased amount of O_2_ (μmol *Chla* mg^−1^ h^−1^). To quantify the amount of O_2_ evolution from water split during photosynthesis, the cell culture was supplied with saturated light (Supplementary Table 2).

### Quantification of the ^13^C-labeled sugar phosphates and acetyl-CoA

The WT and *Δxpk* cultures (50 ml, three batches for each) were first grown under continuous light and then refreshed with BG11 medium before ^13^C-labeling. At time 0, the cultures were added with 2 mM NaH^13^CO_3_ and simultaneously, the light was switched off. Samples (2 ml, OD_730_ adjusted to 1) were collected from individual cultures at different time points before (−5, 0 min) and after dark (1, 3, 5 min and so on). The collected cultures were then subjected to 14,000*g* centrifuge for 10 s. The pelleted cells were immediately frozen with liquid nitrogen. The following metabolite extraction procedure was based on Dempo et al.^54^ and Lunn et al.^55^ with modifications. The frozen cell pellets were first mixed with a 200-µl solution (methanol:chloroform:water in a 5:2:2 ratio (v/v)) and then subjected to freeze–vortex–thaw steps for three rounds to disrupt the cell wall. Next, samples were placed in −30 °C for 2 h and subsequently mixed with 150 µl cold water before being centrifuged at 400*g* for 15 min to isolate aqueous phase from protein and nonpolar phase. This cold-water extraction step was repeated once. Then, the aqueous phase was dried by speedVac at 30 °C before adding 100–200 µl water to resuspend the samples. To remove macromolecules, the sample solutions were applied to 10-kDa Ultracell centrifugation tubes. After 10 min centrifugation at 14,000*g*, the flowthrough solution containing phosphosugars and soluble metabolites was isolated for following liquid chromatography-tandem mass spectrometry (LC–MS/MS) analyses. Each sample (10 µl) was loaded to triple quadrupole Shimadzu LC–MS 8045 with YMC-Triart C18 ExRS (metal-free column) (150 mm × 2.1 mm, 1.9 µm, product no. TAR08SP9-15Q1PTP) at a 0.40 ml min^−1^ flow rate. Separation was performed as described by Campos et al.^56^, starting with 100% solvent A. Solvent B was increased linearly to 7.5% over 10 min after the starting point. From 10–13 min, solvent B was increased to 52.6% linearly and was maintained until 15 min. From 15–25 min, 98% solvent B was used to remove the remnant. From 25–30 min, solvent B was decreased to 0% for column equilibration. The column temperature was maintained at 50 °C. LC–MS/MS was set in the negative mode with spectra acquired over a mass range of 50–1,000 m/z. The acquisition parameters were as follows: interface temperature, 250 °C; desolvation line temperature, 200 °C; heat block temperature, 400 °C; desolvation gas, nitrogen; nebulizing gas flow rate, 3.0 l min^−1^; drying gas, nitrogen; drying gas flow rate, 5 l min^−1^. All metabolites and their labeling patterns were measured by multiple reaction monitoring in the negative mode. Each peak area of ^12^C- and ^13^C-incoroporated metabolite was quantified. The ^13^C-incorporated % of individual metabolite was calculated as (^13^C-compound / ^12^C-compound) and shown in Fig. 3 and Supplementary Fig. 1. Statistical significance difference of the ^13^C-incorporated % between the WT and *Δxpk* was analyzed by one-way ANOVA (*n* = 3 biological repeats, mean ± s.e.m.).

### Sugar and biomass composition measurements

To obtain high-cell-density cultures, both WT and *Δxpk* cultures were grown until OD_730_ reached 2–3 and then were refreshed with BG11 medium before adjusting the OD_730_ to 4. An equal volume of individual culture was supplemented with 100 mM NaHCO_3_ and inoculated in a Corning 75-cm^2^ cell culture flask for 4 d under shaking at 70 r.p.m. Two aliquots of 1-ml culture from WT and *Δxpk* were collected and placed in Costar Spin-X centrifuge tube filters and spun at 15,000*g* for 1 min, to separate cells and medium. The medium sample (2 ml) was collected for extracellular sugar content analyses (Fig. 4g). Equal amounts of the cell pellet were used for glycogen and intracellular sucrose measurements (Extended Data Fig. 2 and Fig. 4g). The metabolite extraction method is described above. For extracellular sugar content measurements, 100-μl medium samples were first mixed with known amount of lactate as an internal control and then subjected to 3-kDa Ultracell centrifuge tubes to remove macroparticles. Then, 10 μl of the clear flowthrough was analyzed by a UHPLC Agilent 1290 infinity II system at 50 °C, using Hi-Plex H hydrogen 8 µm, 300 × 6.5 mm column. By isocratic elution with 30 mM sulfuric acid at 0.4 ml min^−1^ flow rate, the glucose and sucrose contents were separated and detected by the refractive index detector. The amount of lactic acid was detected by the diode array detector at UV wavelength 210 nm. For intracellular sucrose and glucose measurements, the sample extracts were quantified by monitoring the NADH production using a colorimetric method. The concentrations of the standard NADH samples were prepared from 0.125 mM to 1 mM. The extracts containing sucrose and glucose were digested to glucose and fructose by mixing with 200 mM sodium acetate, pH 4.8, containing 6 U invertase (from baker’s yeast, Sigma-Aldrich), at 55 °C for 1 h. The cell pellet for glycogen measurement was mixed with 200 μl KOH 30% (v/v) then boiled for 30 min. Acetic acid was added to the sample for adjusting the pH to 7 at room temperature. The sample was then mixed with the enzymatic digesting reagent, containing 50 mM sodium acetate, pH 4.8, 6 U ml^−1^ amyloglucosidase and 50 U ml^−1^ α-amylase. The reactions were held at 37 °C for 2 h to catalyze glycogen to glucose and then stopped by heating to 99 °C for 10 min. Then, 50 μl the digested samples were transferred to a 96-well plate and mixed with 100 μl solution containing 100 mM HEPES, pH 7.5, 1 mM MgCl_2_, 1 mM ATP, 1 mM NAD and 0.6 U hexokinase (from baker’s yeast, Sigma-Aldrich) to convert glucose to G6P. The absorption reading of NADH at OD_340_ was used as a blank. Subsequently, 0.9 μl G6P dehydrogenase (from *Leuconostoc* *mesenteroides*, Roche) was added to each reaction that converted G6P and NAD to phosphogluconate and NADH. The OD_340_ reading was recorded per minute until the values reached a plateau. The method above was also used to quantify intracellular glucose content without invertase reactions, where the sucrose content was calculated by subtracting the glucose content. For biomass measurements (Extended Data Fig. 2a,b), 2-ml cultures were transferred to pre-weighted Costar Spin-X centrifuge tubes. After centrifugation and freeze-drying overnight, the dry-cell weight of the cultures was quantified. The carbon content of the biomass was calculated using the formula CH_1.81_N_0.17_O_0.20_P_0.013_S_0.009_ (ref. ^57^)_._ The total protein content (Extended Data Fig. 2d) of the freeze-dried samples was quantified using a Pierce BCA protein assay kit (Thermo Fisher Scientific).

### Net carbon fixation and respiratory CO_2_ release measurements

For net carbon fixation measurements, the 0.5-ml culture sample described above was collected at the end of each day and night for 4 d. The collected sample was transferred to a 20-ml glass screw-top vial. An equal volume of 37% HCl was then injected to the sample to release CO_2_ into the headspace of the vial. The headspace was loaded into an Agilent Technologies 7890 gas chromatograph equipped with a 5977B mass spectrometer. A DB-FFAP column (Agilent Technologies, 0.32 mm × 30 m × 0.25 μm) was used to detect the amount of CO_2_ released from the sample (Fig. 4e,f). An NaHCO_3_ standard curve was prepared using 0–100 mM NaHCO_3_.

### RNA-seq

A total of 5 ml WT and *Δxpk* cultures (OD_730_ ~ 1, three cultures for each genotype) were collected at four time points (light 1 h, 1 h after switching to light; light 12 h, 12 h illumination; dark 1 h, 1 h after switching to dark; and dark 12 h, 12 h darkness). Cells were pelleted by full-speed centrifugation for 10 s, frozen with liquid nitrogen and then stored at −80 °C until samples were collected from all time points. Cell pellets were washed once with 300 μl QIAGEN RNAprotect cell reagent and centrifuged to remove the supernatant. Cells were frozen with liquid nitrogen and then thawed by adding 350 μl RLT solution, provided by the QIAGEN RNeasy kit. After three rounds of freeze–thaw–vortex steps, the samples were centrifuged at 15,000*g* for 10 min. Supernatant was transferred to the tubes and mixed with an equal volume of 100% ethanol. The mixture was then transferred to the RNeasy mini-spin columns. The following procedure was based on the QIAGEN RNeasy kit and the Invitrogen TURBO DNA-free kit. For RNA-seq experiments, RNA quality control was performed by Agilent fragment analyzer 5200. Library preparation and ribosomal RNA depletion were performed by using Illumina Ribo-Zero Plus rRNA depletion kit. All sequencing was performed on an Illumina Nextseq 2000 with P2 flowcell, 300 cycles and paired ends of 150 bp. Data were processed using CLC genomics workbench 21. Differential gene expression between WT and XPK was examined across all conditions (across group, ANOVA-like), with TMM normalization. The volcano plot *y* axis showed *P* value (−log_10_) between WT and *Δxpk* (Extended Data Fig. 3a). A heat map was generated using values normalized through trimmed mean of M values (TMM) and count per million (CPM) methods, with *k*-means clustering *k* = 4 for gene clustering (Extended Data Fig. 3b). The RNA-seq data are deposited in NCBI Gene Expression Omnibus datasets under accession no. GSE227397.

### Frequency-dependent phenotype experiments

To observe the growth phenotype of WT and *Δxpk* mutant under different frequency L/D cycles, the cell cultures (six batches for both genotypes) were first grown under continuous light until OD_730_ reading reached or just over 1. The starting cultures (1 ml) of the WT and *Δxpk* cells were adjusted with culture medium to obtain the same cell density with OD_730_ = 1 (the 10^0^ culture in Fig. 5a). Tenfold serial dilutions were made from the starting culture to obtain three additional cultures annotated as 10^−1^, 10^−2^ and 10^−3^. Six aliquots (10 μl each) of the individual culture were simultaneously dropped onto the same BG11 plates to give six replicated cell dots. After allowing dots to dry under continuous light, the plates were imaged (day 0) and then subjected to different L/D frequency cycles for 4 d. The images of the plates were taken every 24 h. Autofluorescence images were taken by Fujifilm LAS-4000 at 520 nm excitation and 575DF20 emission filter. The mean autofluorescence intensity of each cell dot (from 10^−1^) and the mean background intensity were analyzed by ImageJ. The calibrated autofluorescence of each cell dot was obtained (Fig. 5b) after subtraction of the background intensity.

### Cryo-EM specimen preparation and data collection

Cryo-EM samples were prepared by applying 4 µl freshly purified *Se*XPK (~0.4 mg ml^−1^, equivalent to 2.2 µM of dimer, protein buffer containing 20 mM HEPES–NaOH (pH 7.2), 0.1 M NaCl, 0.2 mM TPP and 1 mM MgCl_2_) to a holey carbon grid (Quantifoil R 1.2/1.3, 300 mesh copper). The grids were glow-charged at 25 mA for 40 s. For the bound form, AMPPNP was added to the protein sample at a final concentration of 2 mM. The mixture was incubated on ice for at least 30 min before applying to the cryo-grids. After sample application, the grids were subsequently blotted for 4 s at 4 °C in a 95% humidity-controlled chamber, followed by plunge freezing in liquid ethane using a Vitrobot Mark IV (Thermo Fisher Scientific). To obtain the top-viewed orientation of *Se*XPK particles, the grid surface was pre-modified with 0.1 mg ml^−1^ poly-l-lysine (Sigma-Aldrich) immediately after glow discharge. For *B.* *longum*, the XPK sample grid was prepared without using poly-l-lysine because the preferred orientation issue was improved by tilting the specimen stage for an angle of 30°. Cryo-EM specimen grids were imaged on a Titan Krios microscope (Thermo Fisher Scientific) operated at 300 kV. For *Se*XPK samples, dose-fractionated image stacks were recorded on a K2 Summit detector (Gatan) operating in counting mode at ×165,000 nominal magnification (corresponding to a pixel size of 0.82 Å per pixel). Sixty frames of non-gain normalized tiff stacks were recorded with a dose rate of ~13.1 e^−^ Å^−2^ s^−1^ and the total exposure time was set to 4.5 s, resulting in an accumulated dose of ~59 e^−^ Å^−2^ (~1 e^−^ Å^−2^ per frame). For the *B.* *longum* XPK sample, images were recorded on a BioQuantum K3 detector (Gatan) operating in super-resolution mode at ×105,000 nominal magnification (corresponding to a pixel size of 0.415 Å per pixel). Forty frames of non-gain normalized tiff stacks were recorded with a dose rate of ~16.8 e^−^ Å^−2^ s^−1^ and the total exposure time was set to 2.5 s, resulting in an accumulated dose of ~42 e^−^ Å^−2^ (~1 e^−^ Å^−2^ per frame). The datasets were obtained with nominal defocus values ranging from 1.5–2.5 µm and data collection parameters were controlled in an automated manner using EPU v.2.10 software (Thermo Fisher Scientific). No energy filter or objective aperture was implemented during data collection. The data collection parameters of individual datasets are listed in Supplementary Table 3.

### Cryo-EM data processing and 3D reconstruction

All three datasets were corrected for beam-induced motion by MotionCor2 (ref. ^58^) and contrast transfer function (CTF) estimation was performed with Gctf^59^ on the dose-weighted, aligned micrographs. For the *Se*XPK:TPP/Mg^2+^-AMPPNP-bound form and *Se*XPK:TPP/Mg^2+^ datasets collected in the counting mode, unbinned movies were motion corrected and the pixel size 0.82 Å per pixel was unchanged. For *B.* *longum* XPK:TPP/Mg^2+^ datasets collected in the super-resolution mode, the movies were binned twice during motion correction, resulting in a pixel size of 0.83 Å per pixel for the final motion-corrected images. For the *Se*XPK:TPP/Mg^2+^-AMPPNP-bound form, initial particle picking was manually conducted using Relion3.0 (ref. ^60^) with a small subset of micrographs. The picked particles were subjected to 2D classification. The good 2D classes were used as templates for auto-picking particles from 2,839 micrographs, which yielded particle images (*n* = 260,589) for 2D classification and eliminated bad particles. Further, approximately half of the particles (*n* = 123,625) were selected for three-dimensional (3D) initial model reconstruction and followed by auto-refine in C6 symmetry. The resulting 3D model and particles were then subjected to 3D classification (class 3). One of the classes possessing more than half the particle population yielded a poor 3D model and was not selected. Thus, the 61,183 particles accounting for the other two classes of 3D model were extracted with a box size of 384 pixels using Relion3.0 (ref. ^60^) for the following 3D auto-refine with C6 symmetry. The resolution of the resulting 3D model was improved to 2.83 Å. Next, the particles were subjected to CTF refinement and Bayesian polishing, which was followed by 3D auto-refine to improve the model resolution to 2.58 Å. The polished particle stacks were imported into cryoSPARC2.0 (ref. ^61^) for further processing. Additional 2D classification was performed to further discard poor particles, which yielded 59,127 particles for following homogeneous refinement in D6 symmetry using cryoSPARC2.0 (ref. ^61^). The resulting resolution of the 3D map was improved to 2.36 Å. Next, to further improve the resolution, we performed the focused refinement to refine the density of individual dimer. First, we performed the symmetry expansion (C6) and then the local refinement with a soft mask focused on one dimer without imposing symmetry (C1). The features of the focus-refined dimer were improved and the resolution was improved to 2.17 Å (Supplementary Fig. 4).

For *Se*XPK:TPP/Mg^2+^, the initial particle images (*n* = 228,398) were picked from 3,983 micrographs and extracted with a box size of 384 pixels by cisTEM 1.0.0-β^62^. The particles were subjected to 2D classification for filtering junk particles. The remaining particles were used for ab initio 3D model reconstruction followed by auto-refine with C6 symmetry using cisTEM 1.0.0-β^62^. The coordinates from cisTEM 1.0.0-β were then imported to Relion3.0 (ref. ^60^) and extracted with a box size of 384 pixels for 2D classification to further eliminate the bad particles. The 3D initial model obtained from cisTEM 1.0.0-β^62^ and CTF refined particles were then subjected to 3D refinement by Relion3.0. For further resolution improvement, the particle images (*n* = 44,708) and 3D model were then transferred to cryoSPARC2.0 (ref. ^61^) for 2D classification (*n* = 42,625) and 3D classification (class 4). The class possessing 71.9% of the particles was selected and subjected to homogeneous refinement with D6 symmetry. Further, particle stacks in C1 symmetry were generated by symmetry expansion (D6) for the following focused refinement. A soft mask corresponding to one of the dimers was generated for the refinement, which yielded a 3D dimer density map with 2.63 Å resolution (Supplementary Fig. 5).

For *B.* *longum* XPK:TPP/Mg^2+^, approximately 1.26 million particles were picked and extracted with a box size of 384 pixels by cisTEM 1.0.0-β^62^ from 6,289 micrographs. Initial iterations of 2D classification were conducted by cisTEM 1.0.0-β^62^ for filtering some junk particles. The coordinates of resulting particles were then transferred to Relion3.0 (ref. ^60^) and extracted with a box size of 64 pixels (384-pixel box size re-scaled to 64 pixels, bin 6) for 2D classification to further eliminate the bad particles. The good particles were re-extracted with a box size of 384 pixels and subjected to ab initio 3D model reconstruction with C4 symmetry. The resulting model and particles were used for 3D classification (class 3) with C4 symmetry. One of the classes, which accounted for 25.9% of the particles and yielded a poor 3D model, was not selected for further refinement. The remaining particles from the other two classes were used for particle polishing and 3D auto-refine by Relion3.0 (ref. ^60^), resulting in a 3D map at 2.72 Å. To further improve the resolution, the polished shiny particles and 3D maps were then transferred to cryoSPARC2.0 (ref. ^61^) for homogeneous refinement with D4 symmetry. Then symmetry expansion (D4) was applied to generate the new particle stacks. A soft mask corresponding to one of the dimers was generated for focused refinement, yielding a dimer 3D map with an improved resolution of 2.62 Å (Supplementary Fig. 6).

As the donut-shape or four-leaf clover shape-like particles or 2D class average images were not in perfect symmetry (Supplementary Figs. 4b, 5b and 6b), we further examined whether the assigned symmetries were pseudosymmetry. The final 3D cryo-EM map (D6 or D4) was subjected to symmetry expansion and generated particle stacks in C1 symmetry. One of the dimers was subjected to 3D classification (class 3). The resulting particles in each class were further used for 3D reconstruction in C1 symmetry. The animated illustrations (3D model and link shown in Supplementary Fig. 7) were made using the three reconstructed maps to highlight the conformational motions of the *Se*XPK dodecamer and *B.* *longum* XPK octamer molecules. Relative movements were observed between different dimers. On the other hand, animated illustrations of the focus-refined dimers from 3D classification (class 3) indicated negligible global variation within the dimer subunits, except a few subtle local differences. Based on these analyses, both *Se*XPK and *B.* *longum* XPK are suggested to exist in pseudosymmetry.

The Fourier shell correlation = 0.143 standard was used to estimate the overall resolution and the local resolution was calculated by cryoSPARC2.0 (ref. ^61^). UCSF-Chimera v.1.15 (ref. ^63^) was used to visualize the 3D density map. The detailed refinement statistics for both the dodecamer/octamer and the focus-refined dimer of individual datasets are listed in Supplementary Table 3.

### Atomic modeling, refinement and validation

An initial model of *Se*XPK was generated based on PDB 3AI7 (ref. ^28^) and *Se*XPK amino acid sequence by using Swiss-Model^64^. This initial model was fit into the cryo-EM focused refined map in UCSF-Chimera v.1.15 (ref. ^63^) and Coot v.0.8.8 (ref. ^65^). AMPPNP and TPP/Mg^2+^ molecules were manually built into the cryo-EM map with Coot v.0.8.8 (ref. ^65^). Initial rounds of rigid body and real-space refinement were performed using Phenix v.1.19 (ref. ^66^). Each subunit may be clearer at different residues. Some local discrepancy of model fitting was rebuilt manually using Coot v.0.8.8 (ref. ^65^) based on the densities from both dimer subunits. A similar approach was used for *B.* *longum* XPK, for which the crystal structure PDB 3AI7 (ref. ^29^) was used as an initial model for model building. The refined dimer was further fit into the D6 (*Se*XPK) or D4 (*B.* *longum* XPK)-symmetrized 3D cryo-EM map for obtaining the dodecamer or octamer assembly, respectively. Initial iterations of refinement were performed using real-space refinement in Phenix v.1.19 (ref. ^66^). Several iterations of manual adjustment of the protein coordinate in Coot v.0.8.8 (ref. ^65^) followed by real-space refinement in Rosetta^67^ were performed while monitoring model quality with MolProbity^68^. Several iterations of real-space refinement on the entire model were completed until refinement statistics converged. The detailed statistics of data processing, model refinement and validation are listed in Supplementary Table 3. Structural visualization and rendering of structural representation were performed using PyMOL Molecular Graphics System, v.2.4.1 Schrödinger and Chimera v.1.15 (ref. ^63^).

#### Protein Data Bank structural search procedures

Structures complexed with ATP or five ATP analogs (ANP, ACP, APC, ZAN and AGS) were obtained from the PDB^32^. There were a total of 3,063 structures (April 2021). The structures that match all of the following criteria were selected for analysis: (1) a distance of ≤5 Å between the atom C5 of the adenine ring and the atom CG (γ-carbon) of paired aromatic residues (Y, W, F and H); and (2) a distance of ≤5 Å between the phosphorus atom PG of the γ-phosphate group and the atom CZ of a nearby arginine residue. Another search only focusing on criterion (1) with an additional 2,870 structures complexed with ADP or AMP was also performed. The corresponding protein names of the matched structures are listed in Source Data File 1. Abbreviations for the molecules in this search were ANP, AMPPNP, phosphoaminophosphonic acid adenylate ester; ACP, AMP-PCP, phosphomethylphosphonic acid adenylate ester; APC, AMP-CPP, diphosphomethylphonic acid adenosyl ester; ZAN, AMP-NPP, adenosine-5′-(α,β)-imido triphosphate; AGS, ATPγS, phosphothiophosphoric acid adenylate ester; ADP, adenosine-5′-diphosphate; AMP, adenosine monophosphate; and ATP, adenosine-5′-triphosphate.

### Protein database search and alignment

The conservation of ATP-binding sequence motif in the phosphoketolase family was investigated using *Se*XPK protein sequence as a query to search in a non-reductant NCBI protein BLAST database^34^, including GeneBank CDS translations, PDB and SwissProt, with a total number of 369,802,622 sequences (5 April 2021). The BLASTP was used as a search algorithm and the alignment score was assigned based on Matrix BLOSUM62. The number of target search sequence was 5,000. Among the 5,000 sequences, 4,930 sequences share at least 60% identity with the query sequence. These sequences were subjected to multiple sequence alignment^69^.

Additional searches were performed for the specific genera listed in Fig. 7b (rows 9–12) but with <60% identity to the *Se*XPK, particularly in *Bifidobacterium* (NCBI taxid, 1678), *Lactococcus* (NCBI taxid, 1357) and *Lactobacillus* (NCBI taxid, 1578). None was found in the non-ATP-regulated *Bifidobacterium* (total 112 sequences, with sequence identity >50%) or *Lactobacillus* (462 sequences with sequence identity >40%). Finally, *Lactococcus* was also examined as it is representative of homofermentative bacteria used in synthetic biology and the XPK structure from *L.* *lactis* has been reported^27–29^. No ATP-regulatory site was found from 93 sequences with >40% identity to *S.* *elongatus*.

### Statistics and reproducibility

All the above measurements included three biological repeats for each genotype as the sampling size, except the phenotype experiment, for which six biological repeats for each genotype were used. With the sample size of three, the mean value, s.e.m. and the one-sided statistical *t*-test via one-way ANOVA can be determined. When the measuring values were under the detecting limit (by LC–MSMS) or extremely high/low from the kinetic curves, the data points were excluded. The samples were blindly numbered and randomly loaded to analytical devices. We repeated the experiments to assess the carbon incorporation rates, carbohydrate productions and enzymatic kinetics three times and to evaluate the growth phenotype twice. Similar results between different experimental repeats were obtained. For cryo-EM experiments, thousands of micrographs were collected for the individual dataset. The detailed data collection statistics are summarized in Supplementary Table 3.

### Reporting summary

Further information on research design is available in the Nature Portfolio Reporting Summary linked to this article.

## Supplementary information

Supplementary Information Supplementary Figs. 1–9, Supplementary Fig. legends 1–9, legends for Source Data Files 1 and 2 and Supplementary references.

Reporting Summary

Supplementary Table 1 Data from steady-state kinetics. Because this mutant reduced the enzyme activity and dramatically increased the *K*_m_, its Michaelis-Menten kinetic curve was difficult to reach the *V*_max_. Each value was calculated from three repeats of kinetic assays. *n* = 3, mean ± s.e.m.

Supplementary Table 2 Deletion of *Se*XPK shares the same photosynthesis and respiration rate as WT.

Supplementary Table 3 Cryo-EM data collection, refinement and validation statistics for the XPK structures.

Supplementary Data PDB validation reports for the cryo-EM structures of *Se*XPK and *Bl*XPK.

## Data Availability

The experimental data for all biochemical analyses, PDB validation files and Source Data files are available in Figshare with the identifier 10.6084/m9.figshare.22672618. The RNA-seq data are deposited in the NCBI Gene Expression Omnibus datasets with accession no. GSE227397. Protein structural coordinates and maps have been deposited in the PDB and the Electron Microscopy Data Bank (EMD), with the following accession codes: AMPPNP-bound *Se*XPK: dimer (PDB, 8IO8, EMD-35611); dodecamer (PDB, 8IO9, EMD-35612); free *Se*XPK: dimer (PDB, 8IOA, EMD-35613); dodecamer (PDB, 8IOE, EMD-35617); free *B.* *longum* XPK: dimer (PDB, 8IO7, EMD-35610); and octamer (PDB, 8IO6, EMD-35609). They are also listed in Supplementary Table 3. The validation reports of the corresponding coordinates files are provided. All other original data not described in Methods or Supplementary Information will be available upon reasonable request. Source data are provided with this paper.

## Source data

Source Data Fig. 1 *Se*XPK is inhibited by 1 mM ATP.
Source Data Fig. 2 Delayed cessation of CO_2_ incorporation by *Δxpk* after L/D switch.
Source Data Fig. 3 The increases of ^13^C-labeled RuBisCO substrate precursors in *Δxpk* after transition to darkness.
Source Data Fig. 4 High-density culture of the *Δxpk* strain enhanced net carbon fixation resulting in sucrose secretion at night.
Source Data Fig. 5 *Se*XPK functions as a regulator in rapid L/D cycles.
Source Data Fig. 7 NCBI accession number of all sequences for alignment.
Source Data Extended Data Fig. 1 Steady-state kinetic analysis of XPK.
Source Data Extended Data Fig. 2 Deletion of *Se*XPK enhanced sucrose production but did not alter biomass.
Source Data Extended Data Fig. 3 Transcriptional differences between WT and *Δxpk* across four time points.
Source Data Extended Data Fig. 8 PDB search.
Source Data Extended Data Fig. 9 Sequence alignment of phosphoketolase from the non-bacterial groups.
