# Author Correction: An ATP-sensitive phosphoketolase regulates carbon fixation in cyanobacteria

**DOI:** 10.1038/s42255-023-00906-8

**Published:** 2023-09-15

**Authors:** Kuan-Jen Lu, Chiung-Wen Chang, Chun-Hsiung Wang, Frederic Y-H Chen, Irene Y. Huang, Pin-Hsuan Huang, Cheng-Han Yang, Hsiang-Yi Wu, Wen-Jin Wu, Kai-Cheng Hsu, Meng-Chiao Ho, Ming-Daw Tsai, James C. Liao

**Affiliations:** 1 Institute of Biological Chemistry, Academia Sinica, Taipei, Taiwan; 2 Graduate Institute of Cancer Biology and Drug Discovery, Taipei Medical University, Taipei, Taiwan; 3 Institute of Biochemical Sciences, National Taiwan University, Taipei, Taiwan

**Keywords:** Metabolism, Cryoelectron microscopy, Photosynthesis

Correction to: *Nature Metabolism* 10.1038/s42255-023-00831-w, published online 22, June 2023.

In the version of this article originally published, panels b–k of Figure 6 were inadvertently mislabeled. Aside from the panel labels, the figure has not been changed, and the descriptions in the text and the legend are not affected. The errors have been corrected in the HTML and PDF versions of the article.

