# Supplementary material for: An ATP-sensitive phosphoketolase regulates carbon fixation in cyanobacteria
Source: Nat Metab. 2023 Jun 22;5(7):1111–26. doi: 10.1038/s42255-023-00831-w (PMC10365998; doi:10.1038/s42255-023-00831-w)
Supplement: Supplementary file 1 — Supplementary Figs. 1–9, Supplementary Fig. legends 1–9, legends for Source Data Files 1 and 2 and Supplementary references. [file 42255_2023_831_MOESM1_ESM.pdf]

nature metabolism

Article

https://doi.org/10.1038/s42255-023-00831-w

An ATP-sensitive phosphoketolase regulates carbon fixation in cyanobacteria

In the format provided by the
authors and unedited

nature metabolism

Article https://doi.org/10.1038/s42255-023-00831-w

An ATP-sensitive phosphoketolase regulates carbon fixation in cyanobacteria

In the format provided by the authors and unedited

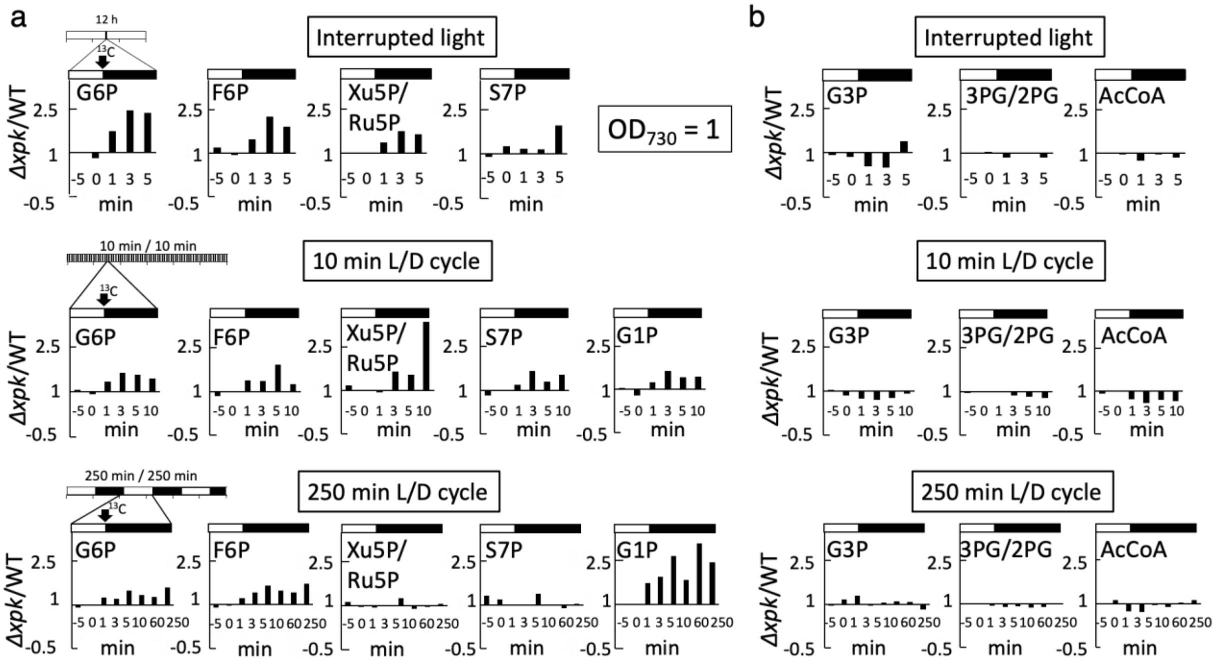

Supplementary Fig. 1. Changes in RuBisCo substrate precursors in rapid response to light-dark switch. Bar plots showing the *Δxpk*/WT ratios of ^{13}C-labelled sugar phosphate minutes after darkness, with detailed data and statistics shown in **Fig. 3**. WT and *Δxpk* cell cultures were grown under different light/dark conditions till OD_{730} reached 1. The cultures were refreshed with BG11 medium, then $NaH^{13}CO_3$ was added right before light to dark switching, which was set as time 0. The samples were harvested 5 and 0 min before ^{13}C-labelling, and at indicated time points afterwards. The % ^{13}C incorporation was analyzed as described in Methods. **a.** XPK substrates and their derived metabolites. Substrates includes F6P, Xu5P, and S7P. G6P can be derived from F6P. G1P can be derived from G6P. Ru5P, an intermediate in a pathway of RuBisCO substrate regeneration, was co-eluted with Xu5P. **b.** XPK products and their derived compounds. G3P is an XPK catalyzed product. 3PG, 2PG and AcCoA are intermediates and product in glycolysis.

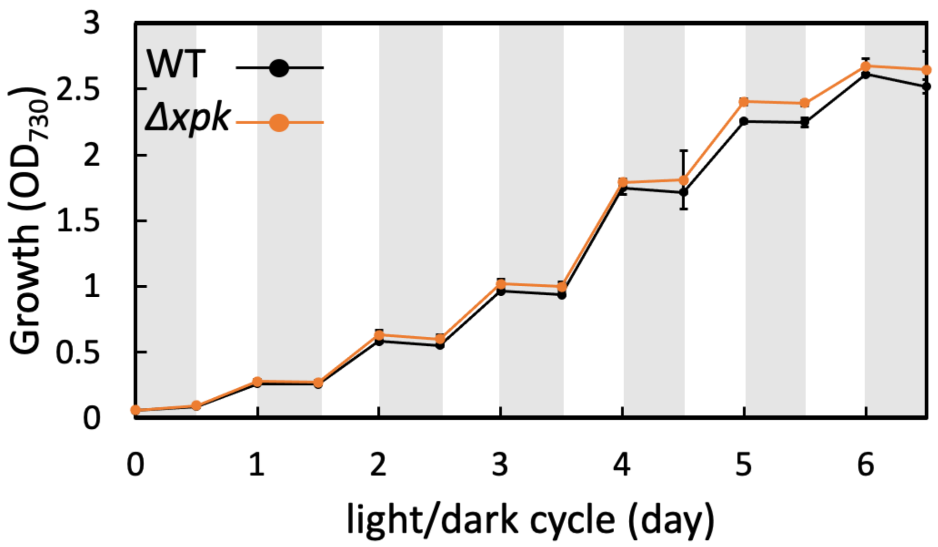

Supplementary Fig. 2. Growth and carbon incorporation of the WT and the *Δxpk* in 12h L/D cycle. Diurnal growth in a week from OD_{730} of 0.1. n= 3 biological repeats, mean ± SEM.

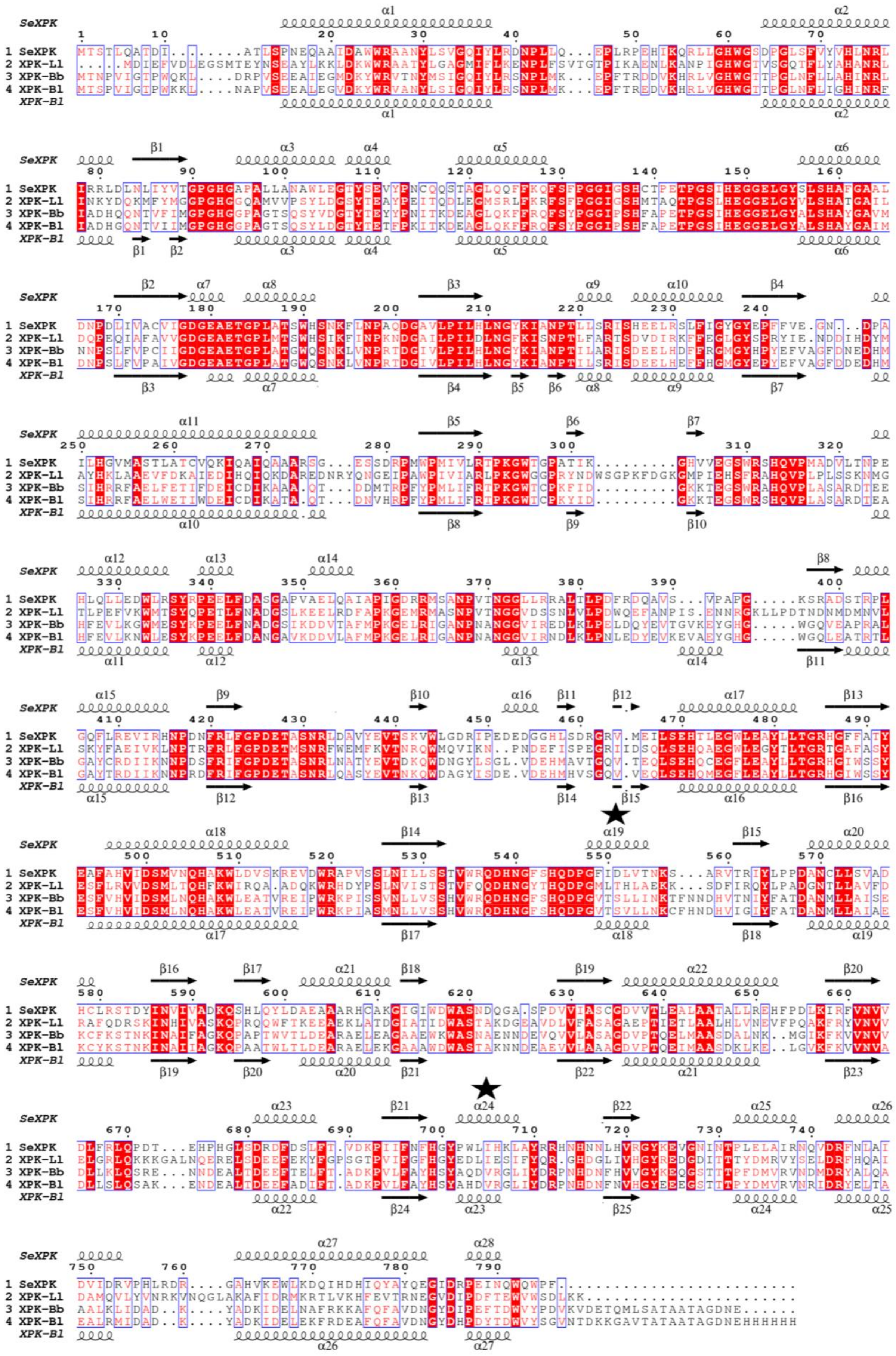

Supplementary Fig. 3. Structure-based alignment of the XPK proteins. Sequences are *Se*XPK (this study), *L. lactis* (XPK-Ll[1]), *B. breve* (XPK-Bb [2], and *B. longum* (XPK-Bl, this study). The sequence alignment was prepared with program Clustal Omega [3], and this final figure was produced with program ESPript 3.0 [4] using default setting. Residue numbering is based on the *Se*XPK. The secondary structure elements (helices: α-helices; arrow: β-strands) of *Se*XPK (PDB 8IOA; EMD-35613) and XPK-Bl (PDB 8IO7; EMD-35610) are illustrated above and under the sequence alignment, respectively. Sequences highlighted with red box and framed with blue lines indicate identical and conserved residues, correspondingly. The α19 and α24 helices of *Se*XPK, which include key residues involved in the allosteric mechanism, are specifically marked with black stars.

a

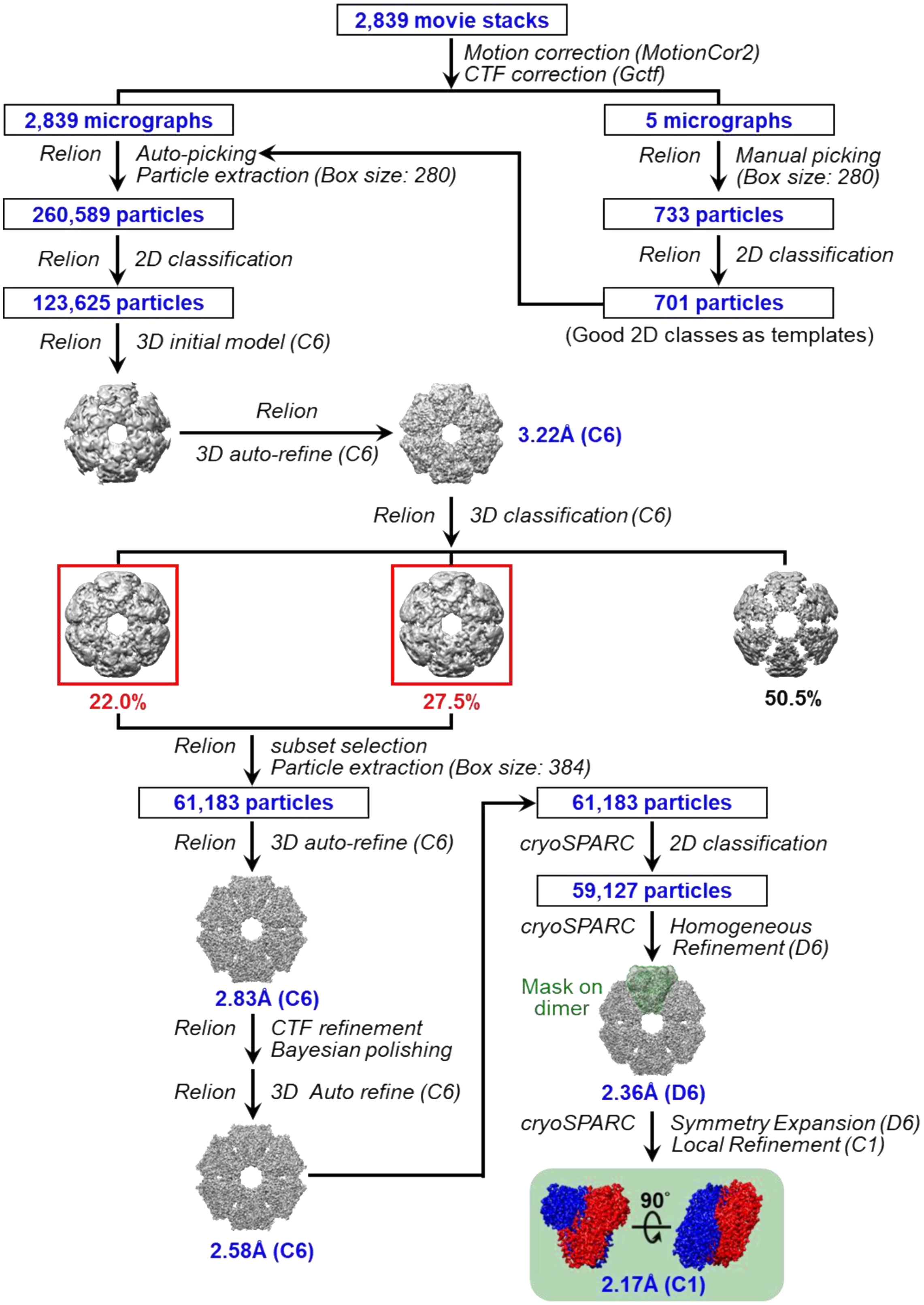
2,839 movie stacks
Motion correction (MotionCor2)
CTF correction (Gctf)
2,839 micrographs
5 micrographs
Relion
Auto-picking
Particle extraction (Box size: 280)
Relion
Manual picking
(Box size: 280)
260,589 particles
733 particles
Relion
2D classification
Relion
2D classification
123,625 particles
701 particles
(Good 2D classes as templates)
Relion
3D initial model (C6)
Relion
3D auto-refine (C6)
3.22Å (C6)
Relion
3D classification (C6)
22.0%
27.5%
50.5%
Relion
subset selection
Particle extraction (Box size: 384)
61,183 particles
61,183 particles
Relion
3D auto-refine (C6)
cryoSPARC
2D classification
59,127 particles
cryoSPARC
Homogeneous
Refinement (D6)
Mask on
dimer
2.83Å (C6)
Relion
CTF refinement
Bayesian polishing
2.36Å (D6)
Relion
3D Auto refine (C6)
cryoSPARC
Symmetry Expansion (D6)
Local Refinement (C1)
90°
2.58Å (C6)
2.17Å (C1)

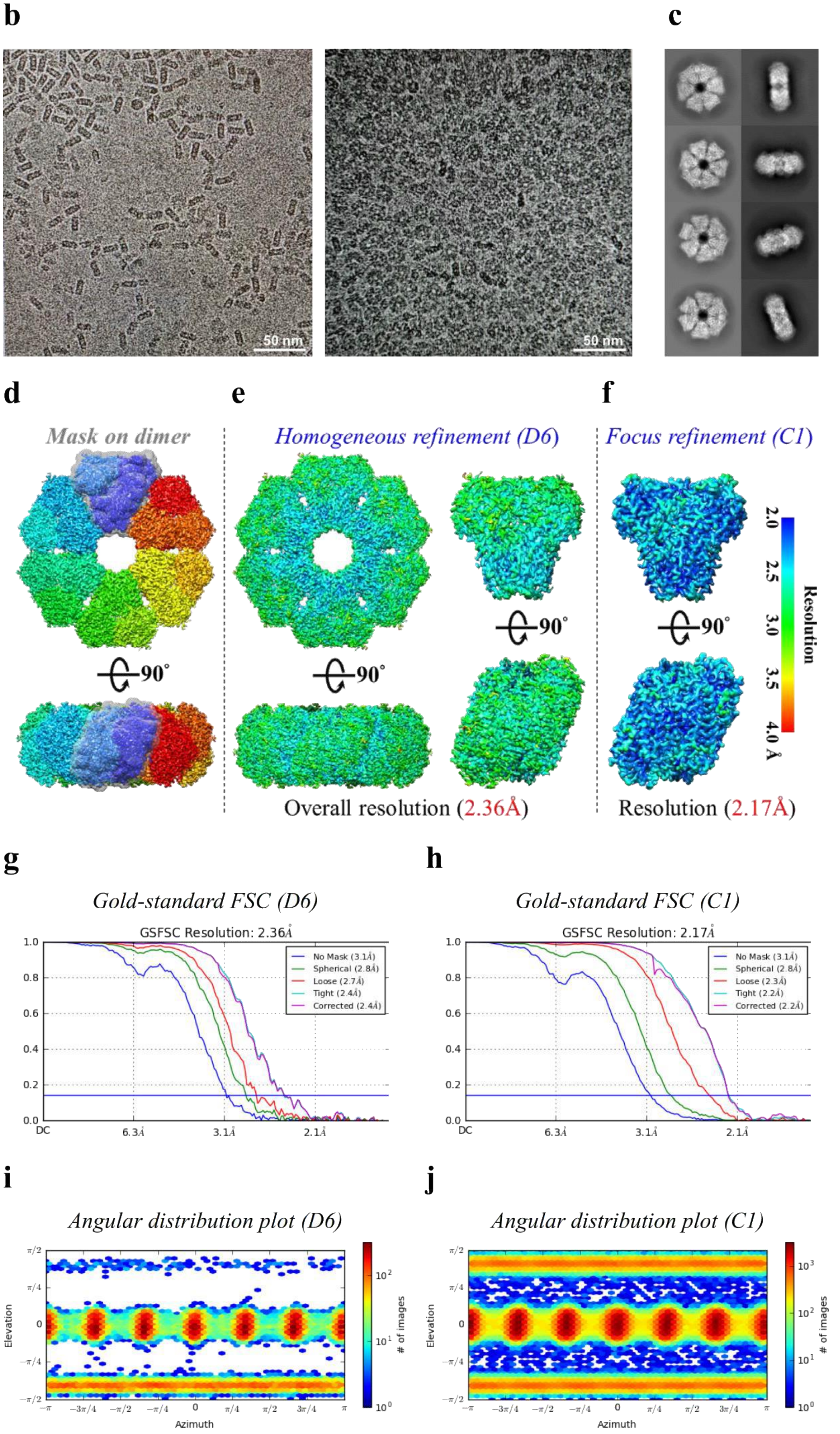
b
c
50 nm
50 nm
d
e
f
Mask on dimer
Homogeneous refinement (D6)
Focus refinement (C1)
90°
90°
90°
90°
2.0
2.5
3.0
3.5
4.0 Å
Resolution
Overall resolution (2.36Å)
Resolution (2.17Å)
g
h
Gold-standard FSC (D6)
Gold-standard FSC (C1)
GSFSC Resolution: 2.36Å
GSFSC Resolution: 2.17Å
No Mask (3.1Å)
Spherical (2.8Å)
Loose (2.7Å)
Tight (2.4Å)
Corrected (2.4Å)
No Mask (3.1Å)
Spherical (2.8Å)
Loose (2.3Å)
Tight (2.2Å)
Corrected (2.2Å)
DC
6.3Å
3.1Å
2.1Å
i
j
Angular distribution plot (D6)
Angular distribution plot (C1)
Elevation
Azimuth
# of images

Supplementary Fig. 4. Cryo-EM reconstruction of AMPPNP-bound *Se*XPK. a. The flow chart for the cryo-EM data processing and structure determination of the AMPPNP-bound *Se*XPK. Details can be found in the Materials and Methods. **b.** Representative motion-corrected and dose-weighted cryo-electron micrographs. Total 2,839 micrographs were analyzed. **c.** Representative 2D class averages show particle quality and orientation. **d.** Cryo-EM structure of AMPPNP-bound *Se*XPK colored by subunits (contoured at 3.4σ). The mask used for focus refinement is shown in transparent grey. **e.** Local resolution analysis of the homogeneous refined D6 symmetry map. A segmented dimer was shown in the right panel. **f.** Local resolution analysis of a masked dimer (contoured at 5.2σ) after symmetry expansion (D6) and focus refinement (C1). The cryo-EM map is colored according to the local resolution. A better map was obtained for a single dimer and the resolution was improved from 2.36 Å to 2.17 Å. **g.** The FSC curves with the gold standard algorithm (FSC= 0.143) of the homogeneous refined D6 symmetry map and **h.** the focus-refined C1 symmetry map after symmetry expansion. **i.** The angular distribution calculated in cryoSPARC2.0[5] for all particle projections in the final 3D reconstruction of the homogeneous refined D6 symmetry map and **j.** the focus-refined C1 symmetry map after symmetry expansion. The heat map shows the number of particles for each viewing angle. Red represents a view with more particles.

a

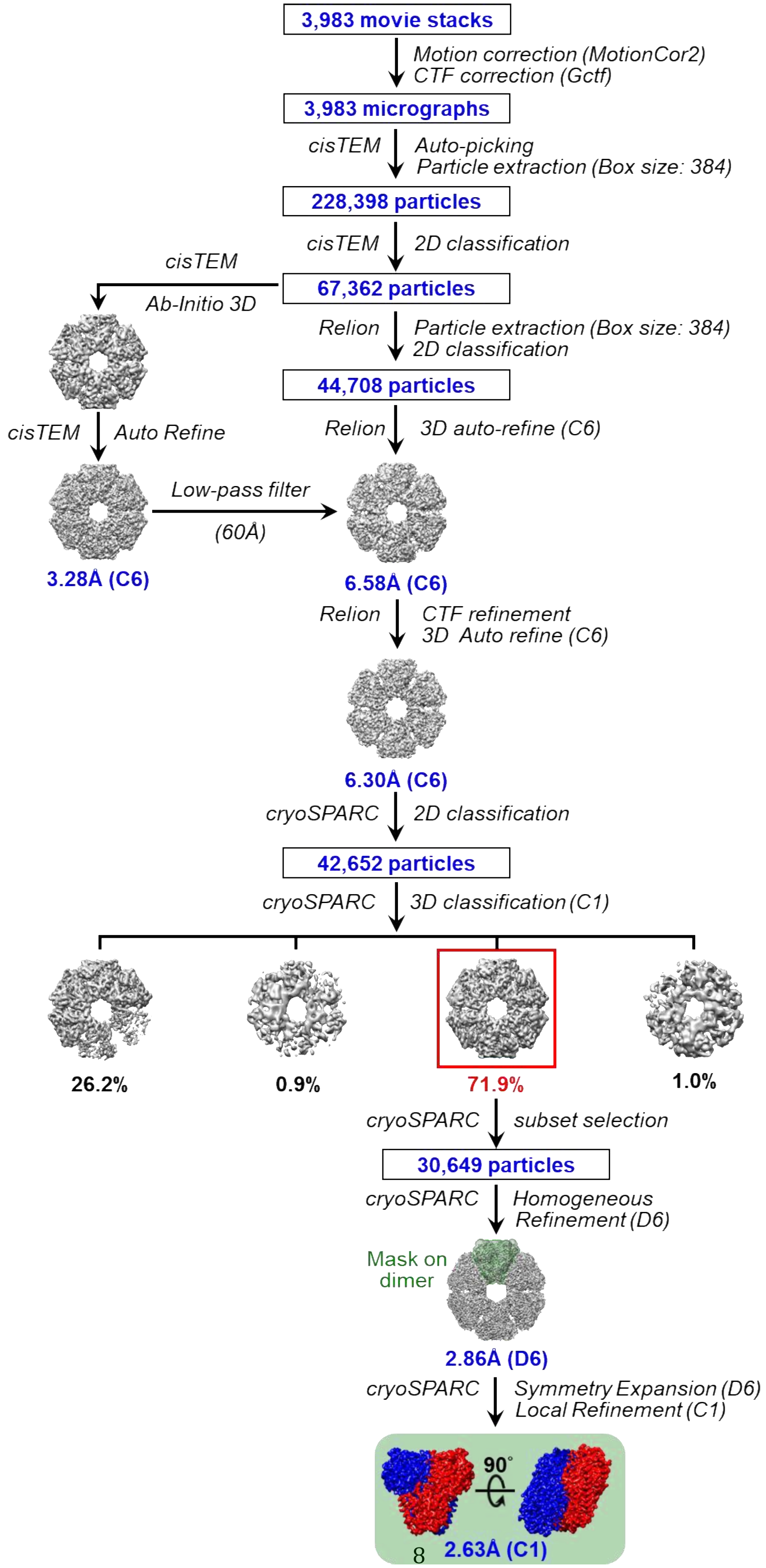
3,983 movie stacks
Motion correction (MotionCor2)
CTF correction (Gctf)
3,983 micrographs
cisTEM
Auto-picking
Particle extraction (Box size: 384)
228,398 particles
cisTEM
2D classification
cisTEM
Ab-Initio 3D
67,362 particles
Relion
Particle extraction (Box size: 384)
2D classification
44,708 particles
cisTEM
Auto Refine
Relion
3D auto-refine (C6)
Low-pass filter
(60Å)
3.28Å (C6)
6.58Å (C6)
Relion
CTF refinement
3D Auto refine (C6)
6.30Å (C6)
cryoSPARC
2D classification
42,652 particles
cryoSPARC
3D classification (C1)
26.2%
0.9%
71.9%
1.0%
cryoSPARC
subset selection
30,649 particles
cryoSPARC
Homogeneous
Refinement (D6)
Mask on
dimer
2.86Å (D6)
cryoSPARC
Symmetry Expansion (D6)
Local Refinement (C1)
90°
2.63Å (C1)

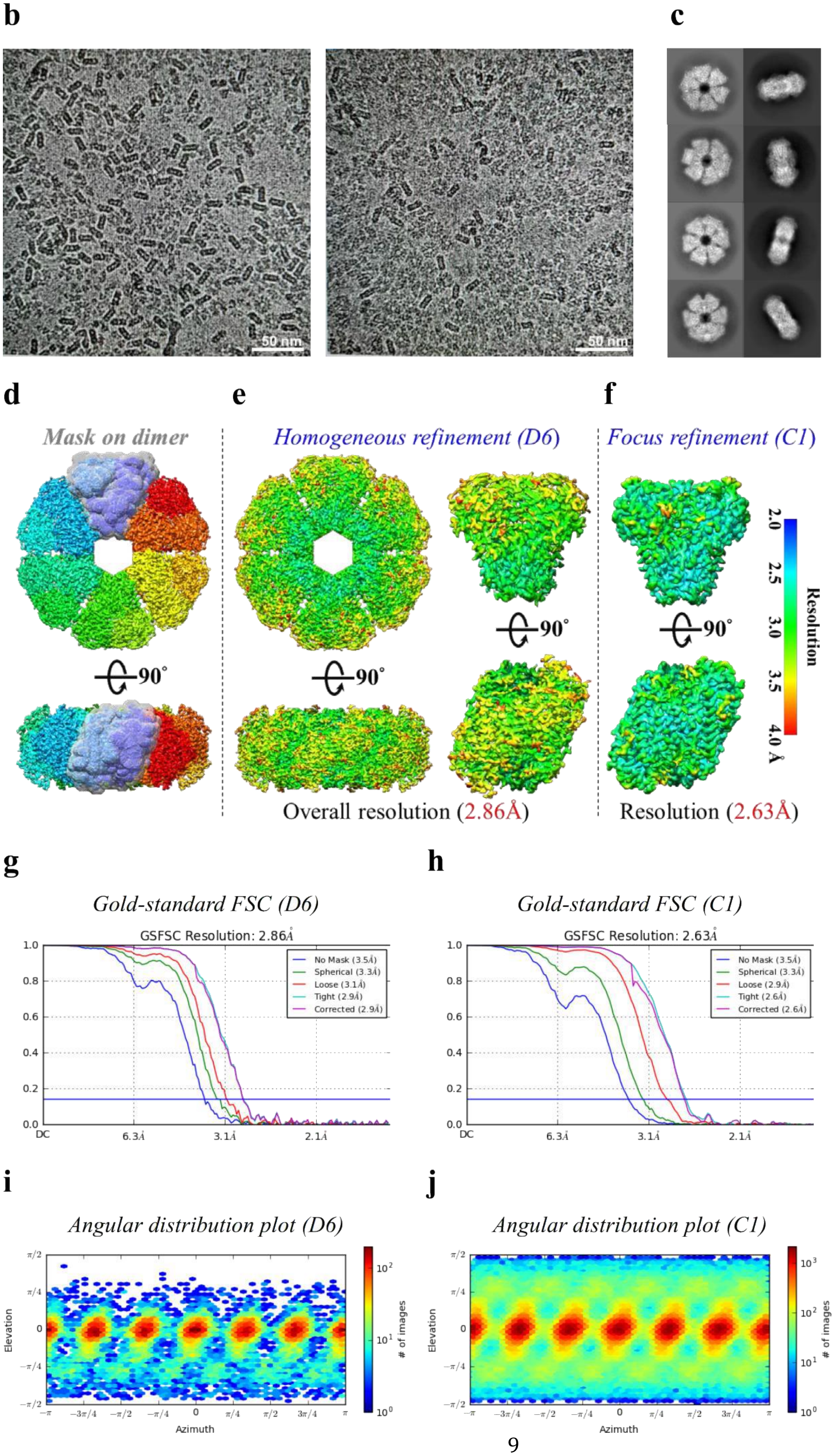
b
50 nm
50 nm
c
d
Mask on dimer
90°
e
Homogeneous refinement (D6)
90°
90°
Overall resolution (2.86Å)
f
Focus refinement (C1)
90°
Resolution (2.63Å)
2.0
2.5
3.0
3.5
4.0 Å
Resolution
g
Gold-standard FSC (D6)
GSFSC Resolution: 2.86Å
No Mask (3.5Å)
Spherical (3.3Å)
Loose (3.1Å)
Tight (2.9Å)
Corrected (2.9Å)
DC
6.3Å
3.1Å
2.1Å
h
Gold-standard FSC (C1)
GSFSC Resolution: 2.63Å
No Mask (3.5Å)
Spherical (3.3Å)
Loose (2.9Å)
Tight (2.6Å)
Corrected (2.6Å)
i
Angular distribution plot (D6)
Elevation
Azimuth
# of images
j
Angular distribution plot (C1)
Elevation
Azimuth
# of images

Supplementary Fig. 5. Cryo-EM reconstruction of *Se*XPK without ATP. a. The flow chart for the cryo-EM data processing and structure determination of the *Se*XPK without ATP. Details can be found in the Materials and Methods. **b.** Representative motion-corrected and dose-weighted cryo-electron micrographs. Total 3,983 micrographs were analyzed. **c.** Representative 2D class averages show particle quality and orientation. **d.** Cryo-EM structure of *Se*XPK without ATP colored by subunits (contoured at 3.4σ). The mask used for focus refinement is shown in transparent grey. **e.** Local resolution analysis of the homogeneous refined D6 symmetry map. A segmented dimer was shown in the right panel. **f.** Local resolution analysis of a masked dimer (contoured at 5.2σ) after symmetry expansion (D6) and focus refinement (C1). The cryo-EM map is colored according to the local resolution. A better map was obtained for a single dimer and the resolution was improved from 2.86 Å to 2.63 Å. **g.** The FSC curves with the gold standard algorithm (FSC= 0.143) of the homogeneous refined D6 symmetry map and **h.** the focus refined C1 symmetry map after symmetry expansion. **i.** The angular distribution calculated in cryoSPARC2.0[5] for all particle projections in the final 3D reconstruction of the homogeneous refined D6 symmetry map and **j.** the focus refined C1 symmetry map after symmetry expansion. The heat map shows the number of particles for each viewing angle. Red represents a view with more particles.

a

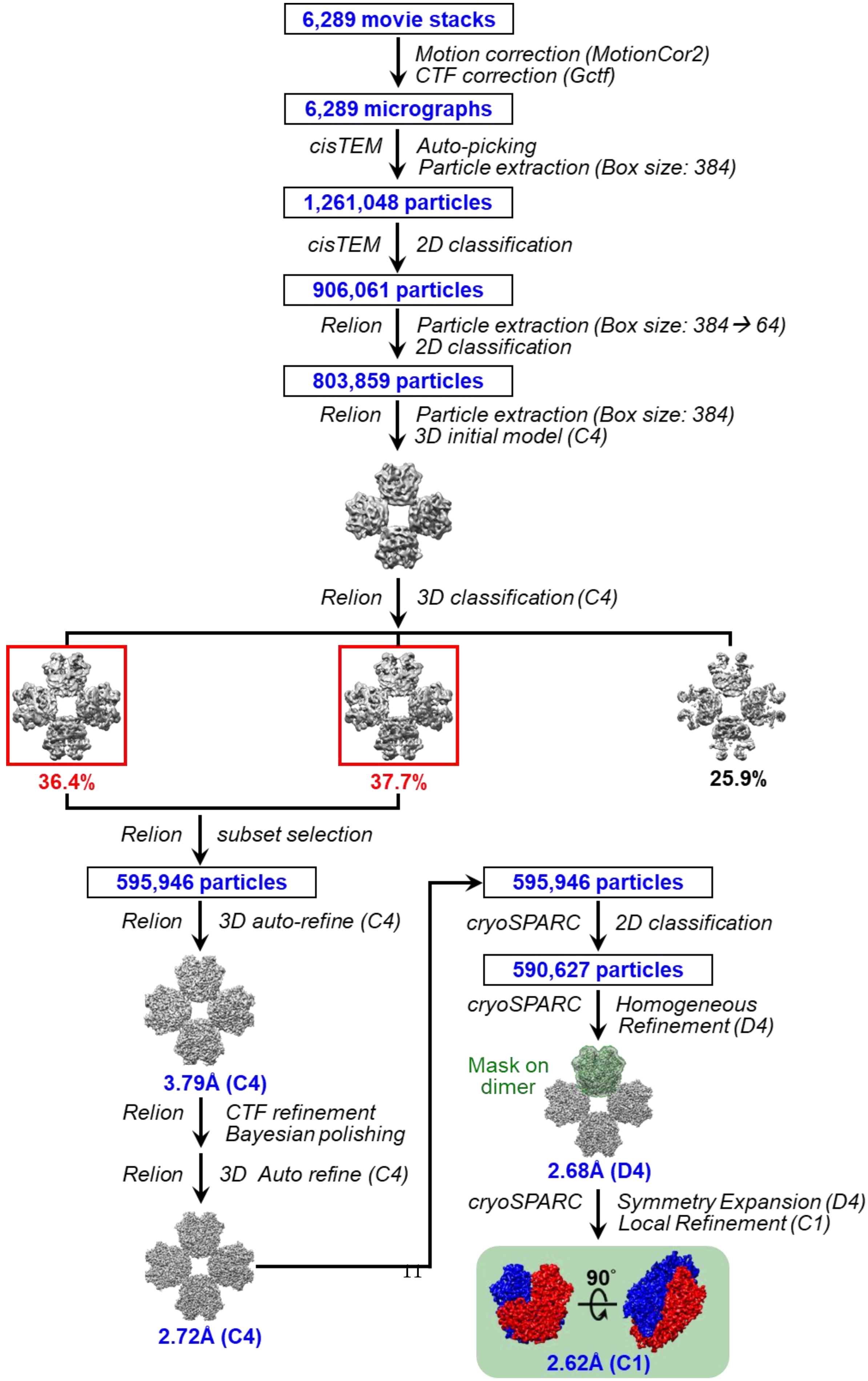
6,289 movie stacks
Motion correction (MotionCor2)
CTF correction (Gctf)
6,289 micrographs
cisTEM
Auto-picking
Particle extraction (Box size: 384)
1,261,048 particles
cisTEM
2D classification
906,061 particles
Relion
Particle extraction (Box size: 384→ 64)
2D classification
803,859 particles
Relion
Particle extraction (Box size: 384)
3D initial model (C4)
Relion
3D classification (C4)
36.4%
37.7%
25.9%
Relion
subset selection
595,946 particles
Relion
3D auto-refine (C4)
3.79Å (C4)
Relion
CTF refinement
Bayesian polishing
Relion
3D Auto refine (C4)
2.72Å (C4)
595,946 particles
cryoSPARC
2D classification
590,627 particles
cryoSPARC
Homogeneous
Refinement (D4)
Mask on
dimer
2.68Å (D4)
cryoSPARC
Symmetry Expansion (D4)
Local Refinement (C1)
90°
2.62Å (C1)

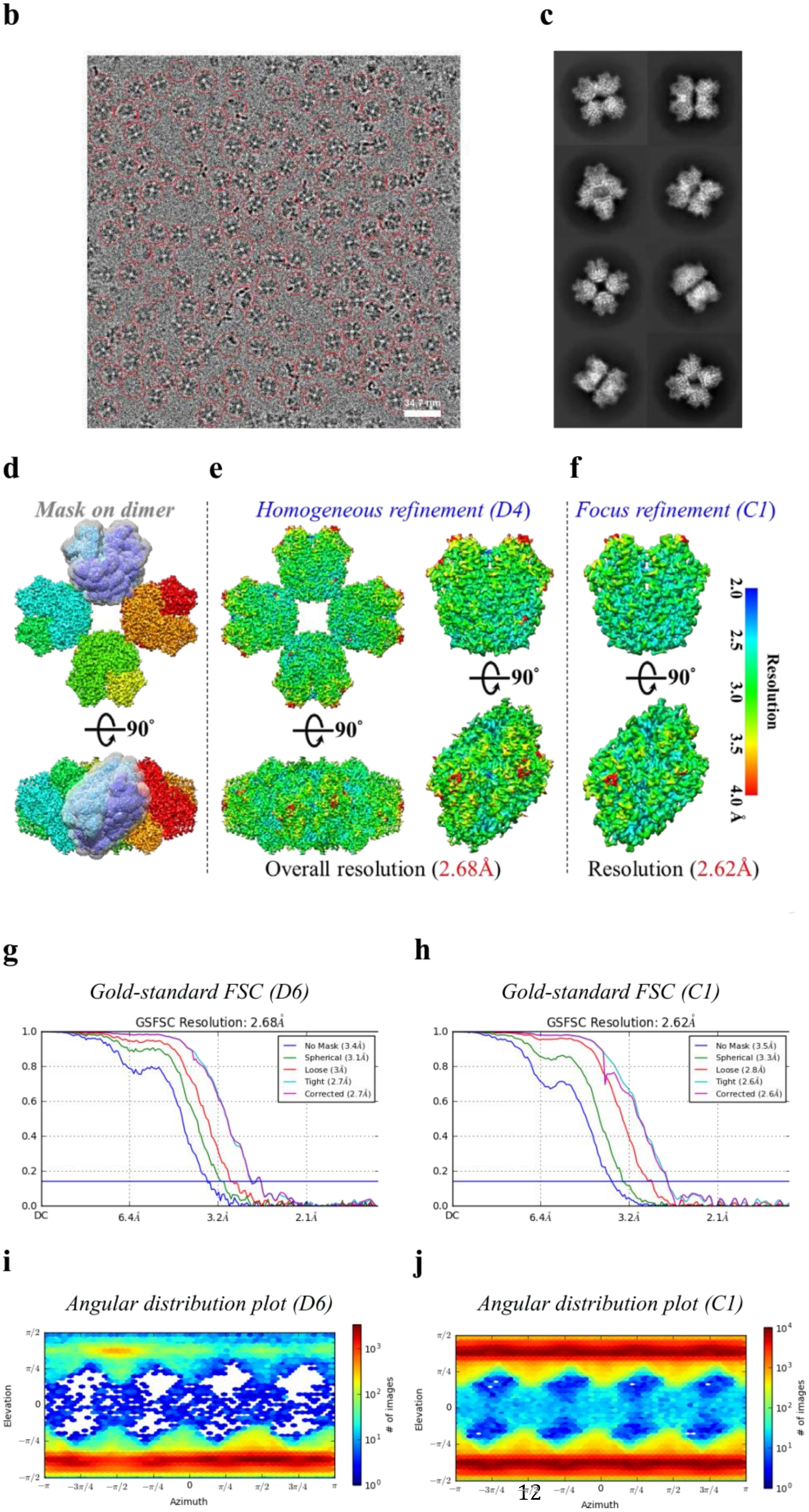
b
c
34.7 nm
d
e
f
Mask on dimer
Homogeneous refinement (D4)
Focus refinement (C1)
90°
Resolution
2.0
2.5
3.0
3.5
4.0 Å
Overall resolution (2.68Å)
Resolution (2.62Å)
g
h
Gold-standard FSC (D6)
GSFSC Resolution: 2.68Å
No Mask (3.4Å)
Spherical (3.1Å)
Loose (3Å)
Tight (2.7Å)
Corrected (2.7Å)
Gold-standard FSC (C1)
GSFSC Resolution: 2.62Å
No Mask (3.5Å)
Spherical (3.3Å)
Loose (2.8Å)
Tight (2.6Å)
Corrected (2.6Å)
DC
6.4Å
3.2Å
2.1Å
i
j
Angular distribution plot (D6)
Angular distribution plot (C1)
Elevation
Azimuth
# of images

Supplementary Fig. 6. Cryo-EM reconstruction of *B. longum* XPK. a. The flow chart for the cryo-EM data processing and structure determination of the *B. longum* XPK. Details can be found in the Materials and Methods. **b.** Representative motion-corrected and dose-weighted cryo-electron micrographs. Total 6,289 micrographs were analyzed. **c.** Representative 2D class averages show particle quality and orientation. **d.** Cryo-EM structure of *B. longum* XPK colored by subunits (contoured at 3.4σ). The mask used for focus refinement is shown in transparent grey. **e.** Local resolution analysis of the homogeneous refined D4 symmetry map. A segmented dimer was shown in the right panel. **f.** Local resolution analysis of a masked dimer (contoured at 7.4σ) after symmetry expansion (D4) and focus refinement (C1). The cryo-EM map is colored according to the local resolution. A better map was obtained for a single dimer and the resolution was improved from 2.68 Å to 2.62 Å. **g.** The FSC curves with the gold standard algorithm (FSC= 0.143) of the homogeneous refined D4 symmetry map and **h.** the focus refined C1 symmetry map after symmetry expansion. **i.** The angular distribution calculated in cryoSPARC2.0[5] for all particle projections in the final 3D reconstruction of the homogeneous refined D4 symmetry map and **j.** the focus refined C1 symmetry map after symmetry expansion. The heat map shows the number of particles for each viewing angle. Red represents a view with more particles.

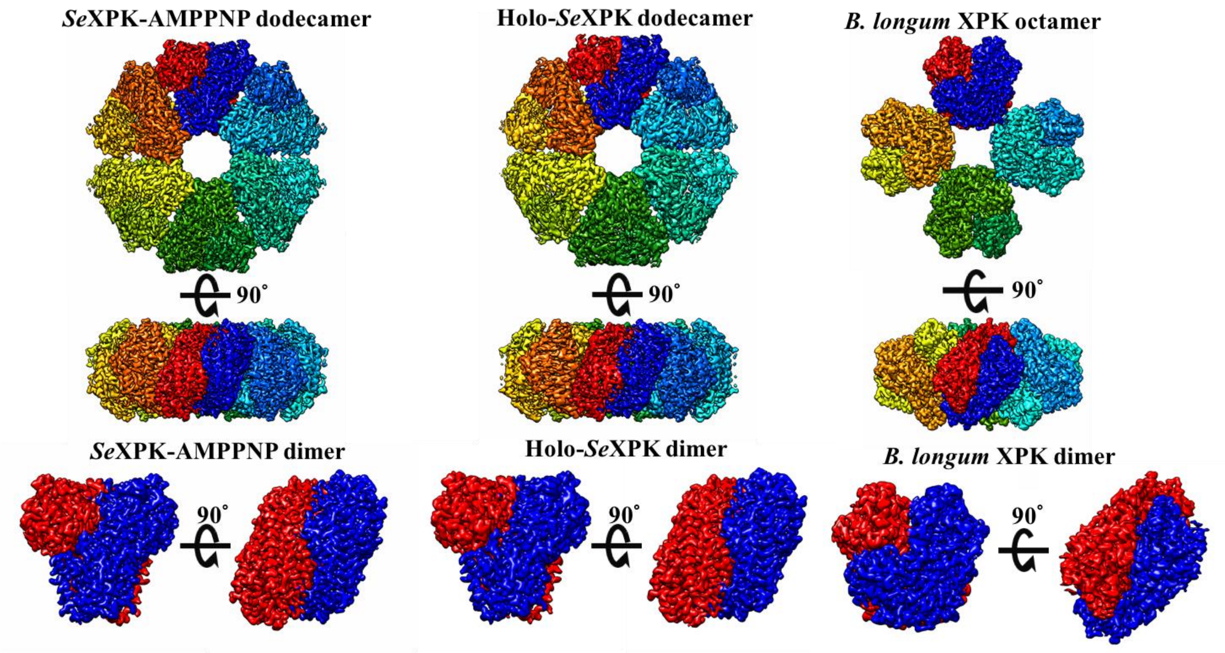

Supplementary Fig. 7. *Se*XPK and *B. longum* XPK in pseudo-symmetry.

The orthogonal views of the 3D volume model are shown for the *Se*XPK dodecameric and *B. longum* XPK octameric assemblies. One of the corresponding dimers is shown in the bottom panel. The animation illustrating the motions of the molecules can be accessed in the link: https://docs.google.com/presentation/d/1pZHYnUiS6ysphP8I8V_cA6o-l1vrBqYl/edit?usp=sharing&ouid=113218851231323592691&rtpof=true&sd=true

Sequence alignment of phosphoketolases, with sequence identity ≥ 70% to *Se*XPK. The 8 major residues are marked with black stars.

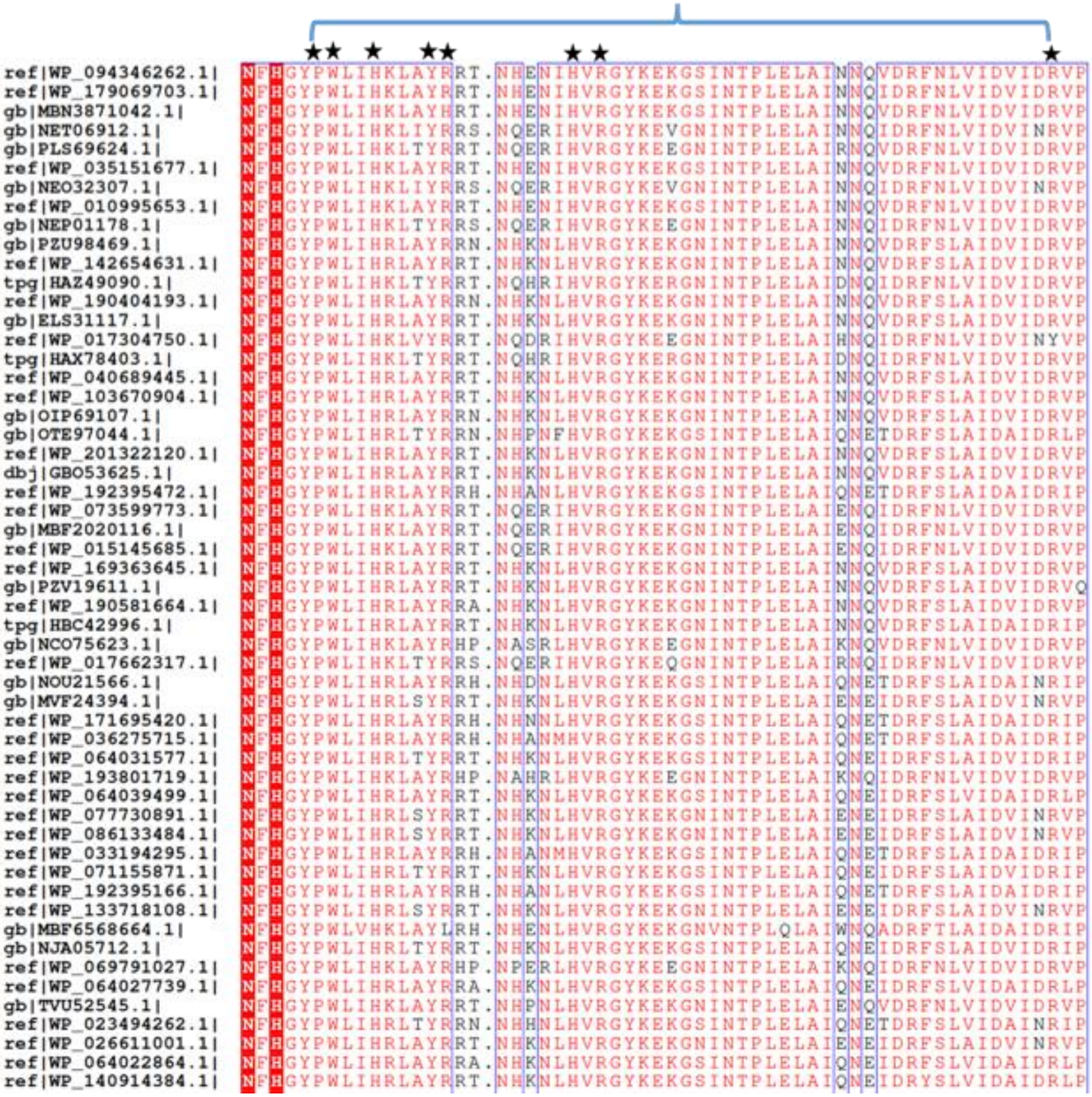

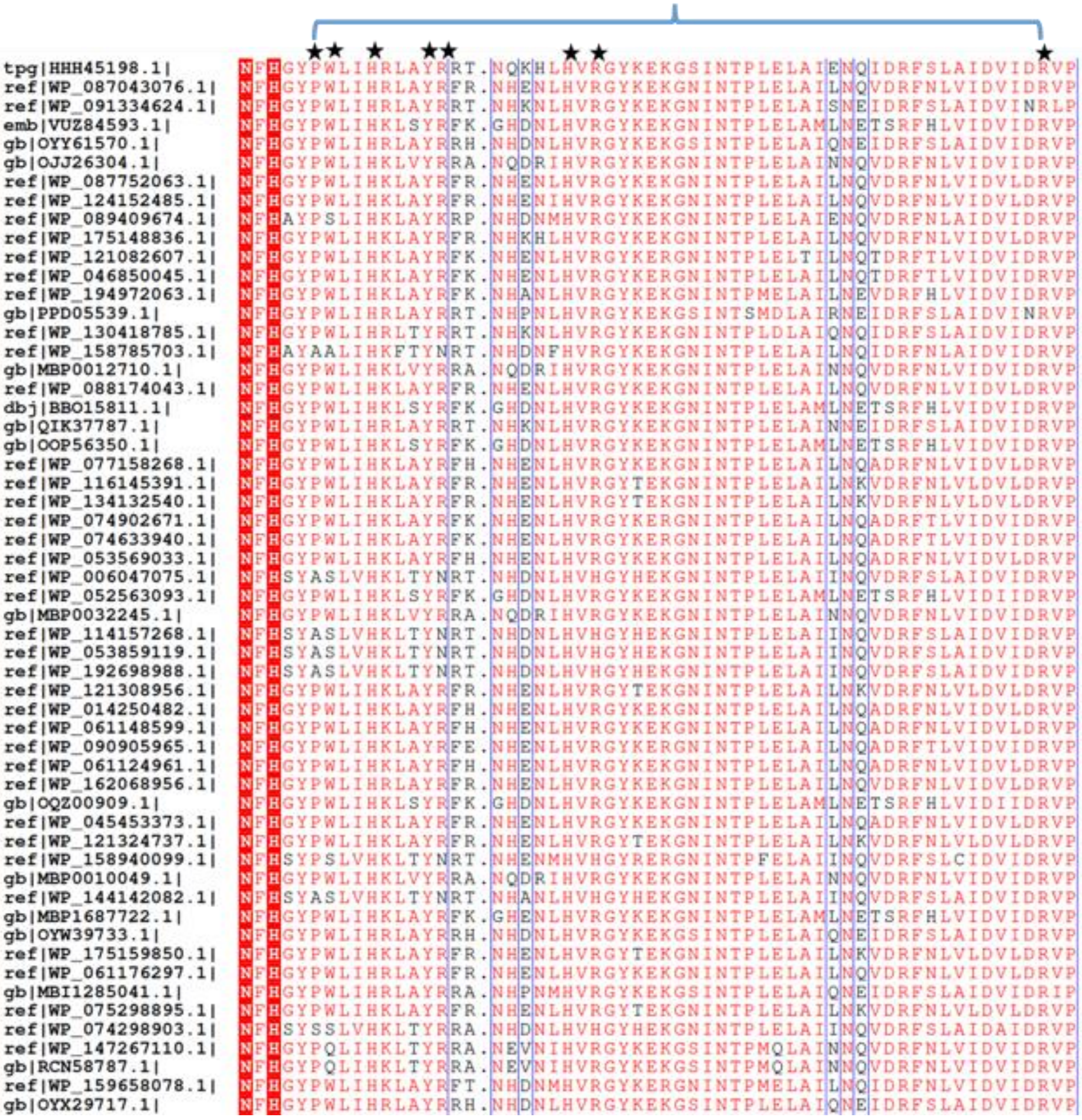
tpg|HHH45198.1| NFHGYPWLIHRLAYRRT.NQKHLHVRGYKEKGSINTPLELAIENQIDRFSLAIDVIDRVP
ref|WP_087043076.1| NFHGYPWLIHRLAYRFR.NHENLHVRGYKEKGNINTPLELAILNQVDRFNLVIDVIDRVP
ref|WP_091334624.1| NFHGYPWLIHRLAYRRT.NHKNLHVRGYKEKGNINTPLELAISNEIDRFSLAIDVINRLP
emb|VUZ84593.1| NFHGYPWLIHKLSYRFK.GHDNLHVRGYKEKGNINTPLELAMLNETSRFHLVIDVIDRVP
gb|OYY61570.1| NFHGYPWLIHRLAYRRH.NHDNLHVRGYKEKGSINTPLELAIQNEIDRFSLAIDVIDRVP
gb|OJJ26304.1| NFHGYPWLIHKLVYRRA.NQDRIHVRGYKEKGNINTPLELAINNQVDRFNLVIDVIDRVP
ref|WP_087752063.1| NFHGYPWLIHKLAYRFR.NHENLHVRGYKEKGNINTPLELAILNQVDRFNLVIDVLDRVP
ref|WP_124152485.1| NFHGYPWLIHKLAYRFR.NHENIHVRGYKEKGNINTPLELAILNQVDRFNLVIDVLDRVP
ref|WP_089409674.1| NFHAYPSLIHKLAYKRP.NHDNMHVRGYKEKGNINTPLELAIENQVDRFNLAIDVIDRVP
ref|WP_175148836.1| NFHGYPWLIHKLAYRFR.NHKHLHVRGYKEKGNINTPLELAILNQVDRFNLVIDVLDRVP
ref|WP_121082607.1| NFHGYPWLIHKLAYRFK.NHENLHVRGYKERGNINTPLELTILNQTDRFTLVIDVIDRVP
ref|WP_046850045.1| NFHGYPWLIHKLAYRFK.NHENLHVRGYKERGNINTPLELAILNQTDRFTLVIDVIDRVP
ref|WP_194972063.1| NFHGYPWLIHKLAYRFK.NHANLHVRGYKEKGNINTPMELAILNEVDRFHLVIDVIDRVP
gb|PPD05539.1| NFHGYPWLIHRLAYRRT.NHPNLHVRGYKEKGSINTSMDLAIRNEIDRFSLAIDVINRVP
ref|WP_130418785.1| NFHGYPWLIHRLTYRRT.NHKNLHVRGYKEKGNINTPLDLAIQNQIDRFSLAIDVIDRVP
ref|WP_158785703.1| NFHAYAALIHKFTYNRT.NHDNFHVRGYKEKGNINTPLELAILNQIDRFNLAIDVIDRVP
gb|MBP0012710.1| NFHGYPWLIHKLVYRRA.NQDRIHVRGYKEKGNINTPLELAINNQVDRFNLVIDVIDRVP
ref|WP_088174043.1| NFHGYPWLIHKLAYRFR.NHENLHVRGYKEKGNINTPLELAILNQVDRFNLVIDVLDRVP
dbj|BBO15811.1| NFHGYPWLIHKLSYRFK.GHDNLHVRGYKEKGNINTPLELAMLNETSRFHLVIDVIDRVP
gb|QIK37787.1| NFHGYPWLIHRLAYRRT.NHKNLHVRGYKEKGNINTPLELAINNEIDRFSLAIDVIDRVP
gb|OOP56350.1| NFHGYPWLIHKLSYRFK.GHDNLHVRGYKEKGNINTPLELAMLNETSRFHLVIDVIDRVP
ref|WP_077158268.1| NFHGYPWLIHKLAYRFH.NHENLHVRGYKEKGNINTPLELAILNQADRFNLVIDVLDRVP
ref|WP_116145391.1| NFHGYPWLIHKLAYRFR.NHENLHVRGYTEKGNINTPLELAILNKVDRFNLVLDVLDRVP
ref|WP_134132540.1| NFHGYPWLIHKLAYRFR.NHENLHVRGYTEKGNINTPLELAILNKVDRFNLVLDVLDRVP
ref|WP_074902671.1| NFHGYPWLIHKLAYRFK.NHENLHVRGYKERGNINTPLELAILNQADRFTLVIDVIDRVP
ref|WP_074633940.1| NFHGYPWLIHKLAYRFK.NHENLHVRGYKERGNINTPLELAILNQADRFTLVIDVIDRVP
ref|WP_053569033.1| NFHGYPWLIHKLAYRFH.NHENLHVRGYKEKGNINTPLELAILNQADRFNLVIDVLDRVP
ref|WP_006047075.1| NFHSYASLVHKLTYNRT.NHDNLHVHGYHEKGNINTPLELAIINQVDRFSLAIDVIDRVP
ref|WP_052563093.1| NFHGYPWLIHKLSYRFK.GHDNLHVRGYKEKGNINTPLELAMLNETSRFHLVIDIIDRVP
gb|MBP0032245.1| NFHGYPWLIHKLVYRRA.NQDRIHVRGYKEKGNINTPLELAINNQVDRFNLVIDVIDRVP
ref|WP_114157268.1| NFHSYASLVHKLTYNRT.NHDNLHVHGYHEKGNINTPLELAIINQVDRFSLAIDVIDRVP
ref|WP_053859119.1| NFHSYASLVHKLTYNRT.NHDNLHVHGYHEKGNINTPLELAIINQVDRFSLAIDVIDRVP
ref|WP_192698988.1| NFHSYASLVHKLTYNRT.NHDNLHVHGYHEKGNINTPLELAIINQVDRFSLAIDVIDRVP
ref|WP_121308956.1| NFHGYPWLIHKLAYRFR.NHENLHVRGYTEKGNINTPLELAILNKVDRFNLVLDVLDRVP
ref|WP_014250482.1| NFHGYPWLIHKLAYRFH.NHENLHVRGYKEKGNINTPLELAILNQADRFNLVIDVLDRVP
ref|WP_061148599.1| NFHGYPWLIHKLAYRFH.NHENLHVRGYKEKGNINTPLELAILNQADRFNLVIDVLDRVP
ref|WP_090905965.1| NFHGYPWLIHKLAYRFE.NHENLHVRGYKERGNINTPLELAILNQADRFTLVIDVIDRVP
ref|WP_061124961.1| NFHGYPWLIHKLAYRFH.NHENLHVRGYKEKGNINTPLELAILNQADRFNLVIDVLDRVP
ref|WP_162068956.1| NFHGYPWLIHRLAYRFR.NHENLHVRGYKEKGNINTPLELAILNQVDRFNLVIDVLDRVP
gb|OQZ00909.1| NFHGYPWLIHKLSYRFK.GHDNLHVRGYKEKGNINTPLELAMLNETSRFHLVIDIIDRVP
ref|WP_045453373.1| NFHGYPWLIHKLAYRFR.NHENLHVRGYKEKGNINTPLELAILNQADRFNLVIDVLDRVP
ref|WP_121324737.1| NFHGYPWLIHKLAYRFR.NHENLHVRGYTEKGNINTPLELAILNKVDRFNLVLDVLDRVP
ref|WP_158940099.1| NFHSYPSLVHKLTYNRT.NHENMHVHGYRERGNINTPFELAIINQVDRFSLCIDVIDRVP
gb|MBP0010049.1| NFHGYPWLIHKLVYRRA.NQDRIHVRGYKEKGNINTPLELAINNQVDRFNLVIDVIDRVP
ref|WP_144142082.1| NFHSYASLVHKLTYNRT.NHANLHVHGYHEKGNINTPLELAIINQVDRFSLAIDVIDRVP
gb|MBP1687722.1| NFHGYPWLIHKLAYRFK.GHENLHVRGYKEKGNINTPLELAMLNETSRFHLVIDVIDRVP
gb|OYW39733.1| NFHGYPWLIHRLAYRRH.NHDNLHVRGYKEKGSINTPLELAIQNEIDRFSLAIDVIDRVP
ref|WP_175159850.1| NFHGYPWLIHKLAYRFR.NHENLHVRGYTEKGNINTPLELAILNKVDRFNLVLDVLDRVP
ref|WP_061176297.1| NFHGYPWLIHRLAYRFR.NHENLHVRGYKEKGNINTPLELAILNQVDRFNLVIDVLDRVP
gb|MBI1285041.1| NFHGYPWLIHRLAYRRA.NHPNMHVRGYKEKGSINTPLELAIQNEIDRFSLAIDVIDRIP
ref|WP_075298895.1| NFHGYPWLIHKLAYRFR.NHENLHVRGYTEKGNINTPLELAILNKVDRFNLVLDVLDRVP
ref|WP_074298903.1| NFHSYSSLVHKLTYRRA.NHDNLHVHGYHEKGNINTPLELAIINQVDRFSLAIDAIDRVP
ref|WP_147267110.1| NFHGYPQLIHKLTYRRA.NEVNIHVRGYKEKGSINTPMQLAINNQVDRFNLVIDVIDRVP
gb|RCN58787.1| NFHGYPQLIHKLTYRRA.NEVNIHVRGYKEKGSINTPMQLAINNQVDRFNLVIDVIDRVP
ref|WP_159658078.1| NFHGYPWLIHKLAYRFT.NHDNMHVRGYKERGNINTPMELAILNQIDRFNLVIDVIDRVP
gb|OYX29717.1| NFHGYPWLIHRLAYRRH.NHDNLHVRGYKEKGSINTPLELAIQNEIDRFSLAIDVIDRVP

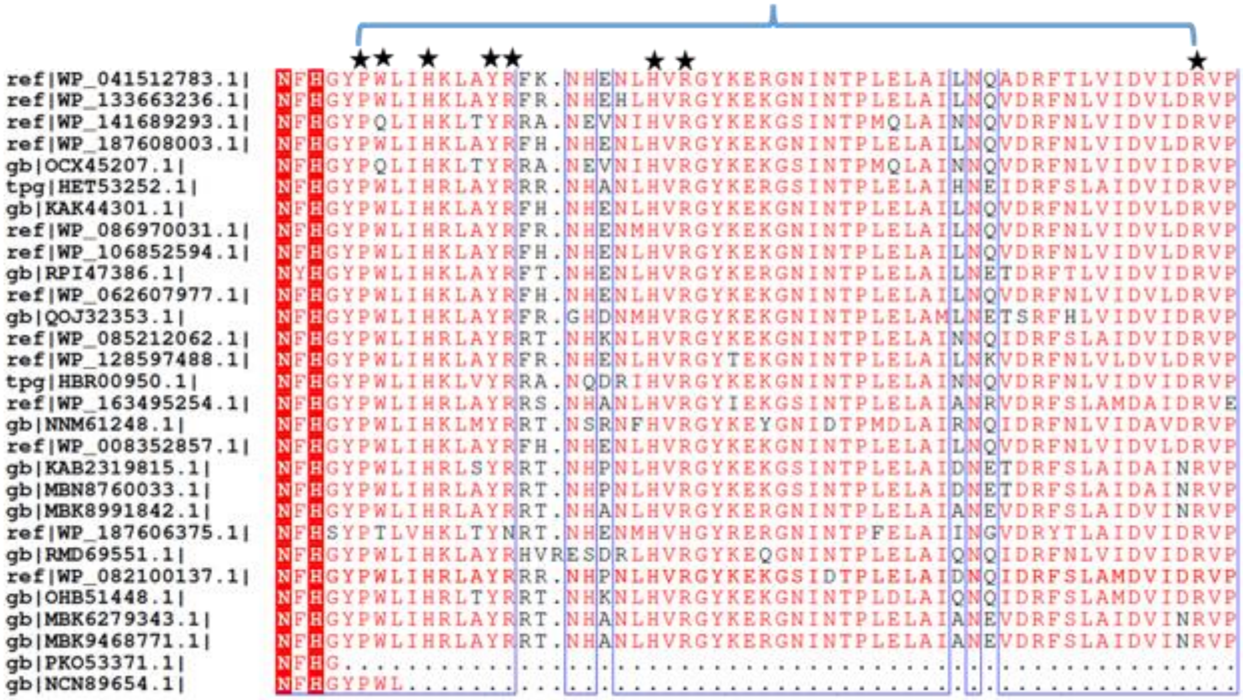

Supplementary Fig. 8. Multiple-sequence alignment of phosphoketolase sequences with identity ≥70%. The alignment only shows the region containing the ATP regulatory motif. Of the 289 sequences listed, 256 meet the criterion of 100% identity with the motif **PW**X_2**H_a**X_3**Y'R'$_a$**X_7**H_b**X**R_b**X_{31}**R_c**, where a,b,c designate histidine or arginine at different positions and the prime sign indicates residues from the reciprocal subunit.

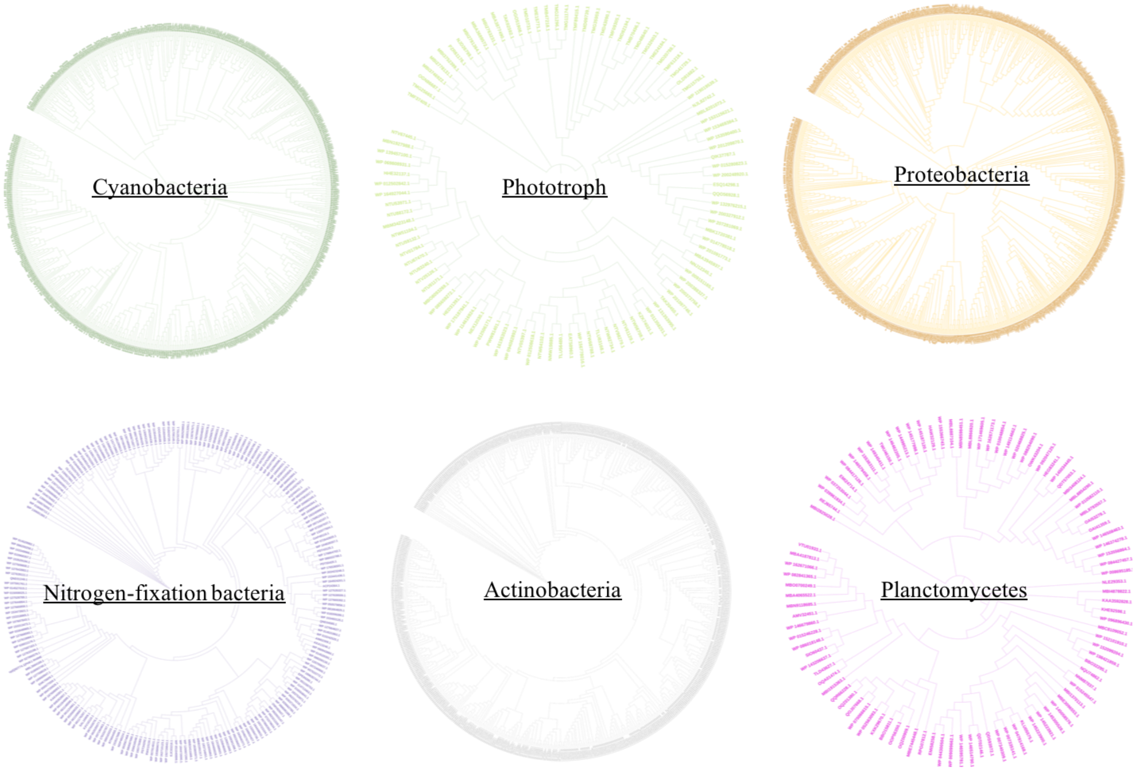

Supplementary Fig. 9. The representative subsets of ATP-regulatory motif containing XPKs. These phylogenetic trees were manipulated using iTOL[6]. Details of these subsets and other categorized ones are documented in **Source Data File II**.

Legends for Source Data Files (large excel files with thousands of items each available in source data files)

Source Data File 1. Protein Data Bank search. The file contains A-sheet: the PDB structures that match criterion (1), and B-sheet: the PDB structures that match both criteria. All listed structures are qualified without taking consideration of distinct protein chains. In the A-sheet: structures labelled in red match the criterion (1) but not (2). The structures listed in the B-sheet match both criteria in the requirement of specific atom distance (< 5 Å), but all have the histidine and tyrosine from the same protein chain. C-sheet: listed structures are qualified from the search data that not only embrace alternative aromatic residues, including F, W, Y, and H, but match both criteria. D-sheet: listed PDB ID [7] and the count of the structures are included in the search pool.

Source Data File 2. NCBI protein BLAST search. The 3190 phosphoketolase sequences containing the ATP regulatory motif are sorted into 14 categories based on species and specific properties, including cyanobacteria, photo(auto)troph, methanotroph, sulfur-oxidizing bacteria, nitrogen-fixation bacteria, thermophilic bacteria, iron-oxidizing bacteria, human pathogen, planctomycetes, actinobacteria, fungi, archeae, proteobacteria and others. The accession code (with NCBI weblink) and relevant information of the 3190 phosphoketolase sequences are listed in the file. Fifteen fungi sequences have a Lys replaced with an Arg right after the Tyr in the motif, equivalent to the *C. neo grubii*–H99 phosphoketolase, which was inhibited by ATP[8]. Therefore, these sequences are intentionally included here.

Supplementary References

1. Scheidig, A.J., Horvath, D. & Szedlacsek, S.E. Crystal structure of a xylulose 5-phosphate phosphoketolase. Insights into the substrate specificity for xylulose 5-phosphate. *J Struct Biol* **207**, 85-102 (2019).
2. Suzuki, R.*, et al.* Crystal structures of phosphoketolase: thiamine diphosphate-dependent dehydration mechanism. *J Biol Chem* **285**, 34279-34287 (2010).
3. Sievers, F.*, et al.* Fast, scalable generation of high-quality protein multiple sequence alignments using Clustal Omega. *Mol Syst Biol* **7**, 539 (2011).
4. Robert, X. & Gouet, P. Deciphering key features in protein structures with the new ENDscript server. *Nucleic Acids Res* **42**, W320-324 (2014).
5. Punjani, A., Rubinstein, J.L., Fleet, D.J. & Brubaker, M.A. cryoSPARC: algorithms for rapid unsupervised cryo-EM structure determination. *Nat Methods* **14**, 290-296 (2017).
6. Letunic, I. & Bork, P. Interactive Tree Of Life (iTOL) v5: an online tool for phylogenetic tree display and annotation. *Nucleic Acids Res* **49**, W293-W296 (2021).
7. Berman, H.M.*, et al.* The Protein Data Bank. *Nucleic Acids Res* **28**, 235-242 (2000).
8. Glenn, K., Ingram-Smith, C. & Smith, K.S. Biochemical and kinetic characterization of xylulose 5-phosphate/fructose 6-phosphate phosphoketolase 2 (Xfp2) from Cryptococcus neoformans. *Eukaryot Cell* **13**, 657-663 (2014).
