# Supplementary material for: An ATP-sensitive phosphoketolase regulates carbon fixation in cyanobacteria
Source: Nat Metab. 2023 Jun 22;5(7):1111–26. doi: 10.1038/s42255-023-00831-w (PMC10365998; doi:10.1038/s42255-023-00831-w)
Supplement: Supplementary file 2 — Reporting Summary [file 42255_2023_831_MOESM2_ESM.pdf]

nature portfolio

Corresponding author(s): James C. Liao, Ming-Daw Tsai

Last updated by author(s): Kuan-Jen Lu, Chiung-Wen Chang 2023/04/21

Reporting Summary

Nature Portfolio wishes to improve the reproducibility of the work that we publish. This form provides structure for consistency and transparency in reporting. For further information on Nature Portfolio policies, see our Editorial Policies and the Editorial Policy Checklist.

Statistics

For all statistical analyses, confirm that the following items are present in the figure legend, table legend, main text, or Methods section.

n/a	Confirmed	
☐	☒	The exact sample size (*n*) for each experimental group/condition, given as a discrete number and unit of measurement
☐	☒	A statement on whether measurements were taken from distinct samples or whether the same sample was measured repeatedly
☐	☒	The statistical test(s) used AND whether they are one- or two-sided<br>*Only common tests should be described solely by name; describe more complex techniques in the Methods section.*
☒	☐	A description of all covariates tested
☐	☒	A description of any assumptions or corrections, such as tests of normality and adjustment for multiple comparisons
☐	☒	A full description of the statistical parameters including central tendency (e.g. means) or other basic estimates (e.g. regression coefficient) AND variation (e.g. standard deviation) or associated estimates of uncertainty (e.g. confidence intervals)
☐	☒	For null hypothesis testing, the test statistic (e.g. *F*, *t*, *r*) with confidence intervals, effect sizes, degrees of freedom and *P* value noted<br>*Give P values as exact values whenever suitable.*
☒	☐	For Bayesian analysis, information on the choice of priors and Markov chain Monte Carlo settings
☒	☐	For hierarchical and complex designs, identification of the appropriate level for tests and full reporting of outcomes
☒	☐	Estimates of effect sizes (e.g. Cohen's *d*, Pearson's *r*), indicating how they were calculated

Our web collection on statistics for biologists contains articles on many of the points above.

Software and code

Policy information about availability of computer code

Data collection	The Data of RNA-seq were collected by Illumina Nextseq 2000. The commercial software used for collecting cryoEM data is Thermo Scientific EPU2.10.
Data analysis	The RNA-seq data were analyzed by CLC genomics workbench 21. cisTEM 1.0.0-beta, Relion 3.0, CryoSPARC 2.0, Coot v.0.8.8, UCSF-Chimera v.1.15, PyMOL-v.2.4.1, and Phenix v.1.19 softwares were used for solving XPK structures in this manuscript. All softwares are publicly available.

For manuscripts utilizing custom algorithms or software that are central to the research but not yet described in published literature, software must be made available to editors and reviewers. We strongly encourage code deposition in a community repository (e.g. GitHub). See the Nature Portfolio guidelines for submitting code & software for further information.

Data

Policy information about availability of data

All manuscripts must include a data availability statement. This statement should provide the following information, where applicable:

- Accession codes, unique identifiers, or web links for publicly available datasets
- A description of any restrictions on data availability
- For clinical datasets or third party data, please ensure that the statement adheres to our policy

The experimental data for biochemical analyses and physiological studies are available in Figshare with the identifier http://doi.org/10.6084/m9.figshare.22672618.

The RNA-seq data is deposited in NCBI GEO DataSets, and the accession number is GSE227397. Protein structural coordinates and maps have been deposited in the Protein Data Bank (PDB) and the Electron Microscopy Data Bank (EMDB), with the following accession codes: AMPPNP bound SeXPK: dimer (PDB 8IO8, EMD-35611); dodecamer (PDB 8IO9, EMD-35612); free SeXPK: dimer (PDB 8IOA, EMD-35613); dodecamer (PDB 8IOE, EMD-35617); free B. longum XPK: dimer (PDB 8IO7, EMD-35610); octamer (PDB 8IO6, EMD-35609). They are also listed in Supplementary Table 3. The validation reports of the corresponding coordinates files are provided. All other original data not described in Methods or Supplementary Information will be available upon reasonable request.

Human research participants

Policy information about studies involving human research participants and Sex and Gender in Research.

Reporting on sex and gender	*Use the terms sex (biological attribute) and gender (shaped by social and cultural circumstances) carefully in order to avoid confusing both terms. Indicate if findings apply to only one sex or gender; describe whether sex and gender were considered in study design whether sex and/or gender was determined based on self-reporting or assigned and methods used. Provide in the source data disaggregated sex and gender data where this information has been collected, and consent has been obtained for sharing of individual-level data; provide overall numbers in this Reporting Summary. Please state if this information has not been collected. Report sex- and gender-based analyses where performed, justify reasons for lack of sex- and gender-based analysis.*
Population characteristics	*Describe the covariate-relevant population characteristics of the human research participants (e.g. age, genotypic information, past and current diagnosis and treatment categories). If you filled out the behavioural & social sciences study design questions and have nothing to add here, write "See above."*
Recruitment	*Describe how participants were recruited. Outline any potential self-selection bias or other biases that may be present and how these are likely to impact results.*
Ethics oversight	*Identify the organization(s) that approved the study protocol.*

Note that full information on the approval of the study protocol must also be provided in the manuscript.

Field-specific reporting

Please select the one below that is the best fit for your research. If you are not sure, read the appropriate sections before making your selection.

☒ Life sciences ☐ Behavioural & social sciences ☐ Ecological, evolutionary & environmental sciences

For a reference copy of the document with all sections, see nature.com/documents/nr-reporting-summary-flat.pdf

Life sciences study design

All studies must disclose on these points even when the disclosure is negative.

Sample size	As three is the minimal sampling size for statistic analysis, we used three biological repeats to perform biochemical analyses and RNA-seq. Our results from the three biological repeats were very similar. We further repeated the same experiment twice to three times to confirm that the results from the simpling size (n=3 biological repeats) are similar between different experiments.
Data exclusions	The measured points were excluded if their values are out of the detection limit or outliers in the kinetic analyses.
Replication	The biochemical experiments including isotopic labeling experiment, carbohydrate measurements, and enzyme kinetics were repeated three times. The growth phenotypes of WT and the XPK deletion line were shown in two experimental repeats. The experimental results between repeated experiments are very similar.
Randomization	Three biological repeats of the WT and the XPK deletion line were randomly placed in the same incubator to avoid the position effect. The samples for each genotype were randomly and simultaneously harvested. The metabolite extractions from the WT and the XPK deletion samples were randomly proceeded and completed in one day. We loaded the samples to the analytic devices (e.g. LCMS-MS, HPLC, and GCMS) not in sequence of time.
Blinding	The samples were randomly numbered prior to applying to analytic devices, including 14C-labeling, 13C-labeling experiments, and metabolite measurements.

Reporting for specific materials, systems and methods

We require information from authors about some types of materials, experimental systems and methods used in many studies. Here, indicate whether each material, system or method listed is relevant to your study. If you are not sure if a list item applies to your research, read the appropriate section before selecting a response.

Materials & experimental systems

n/a	Involved in the study
☒	☐ Antibodies
☒	☐ Eukaryotic cell lines
☒	☐ Palaeontology and archaeology
☒	☐ Animals and other organisms
☒	☐ Clinical data
☒	☐ Dual use research of concern

Methods

n/a	Involved in the study
☒	☐ ChIP-seq
☒	☐ Flow cytometry
☒	☐ MRI-based neuroimaging
