# Supplementary material for: An ATP-sensitive phosphoketolase regulates carbon fixation in cyanobacteria
Source: Nat Metab. 2023 Jun 22;5(7):1111–26. doi: 10.1038/s42255-023-00831-w (PMC10365998; doi:10.1038/s42255-023-00831-w)
Supplement: Supplementary file 3 — Data from steady-state kinetics. Because this mutant reduced the enzyme activity and dramatically increased the Km, its Michaelis-Menten kinetic curve was difficult to reach the Vmax. Each value was calculated from three repeats of kinetic assays. n = 3, mean ± s.e.m. [file 42255_2023_831_MOESM3_ESM.docx]

**Supplementary Tables**

**Supplementary Table 1. Data from steady-state kinetics^b^**

| XFPK species Substrate | Vmax (umol [XPK mg^-1^] min^-1^) | K_m_ (mM) | k_cat_ (s^-1^) |
| --- | --- | --- | --- |
| S. elongatus7942 XPK |  |  |  |
| F6P | 10.8±0.1 | 19±1.5 | 18.2±0.1 |
| pi | 7.7±0.2 | 4.2±0.6 | 12.8±0.3 |
| B. longum XPK |  |  |  |
| F6P | 10.7±1.2 | 10.6±1.3 | 17.9±2.0 |
| S. elongatus7942 XPK H706R |  |  |  |
| F6P | 3.9±0.08 | 61.3±1.6 | 3.2±0.1 |
| S. elongatus7942 XPK H706A |  |  |  |
| F6P | >1^a^ | >80^a^ |  |
| S. elongatus7942 XPK Y710A |  |  |  |
| F6P | 0.2±0.0 | 18±1.2 | 0.2±0.0 |

^a^Because this mutant reduced the enzyme activity and dramatically increased the K_m_, its Michaelis-Menten kinetic curve was difficult to reach the V_max_.

^b^Each value was calculated from three repeats of kinetic assays. n=3, mean ± SE.
