# Supplementary material for: An ATP-sensitive phosphoketolase regulates carbon fixation in cyanobacteria
Source: Nat Metab. 2023 Jun 22;5(7):1111–26. doi: 10.1038/s42255-023-00831-w (PMC10365998; doi:10.1038/s42255-023-00831-w)
Supplement: Supplementary file 4 — Deletion of SeXPK shares the same photosynthesis and respiration rate as WT. [file 42255_2023_831_MOESM4_ESM.docx]

**Supplementary Table 2. Deletion of *Se*XPK shares the same photosynthesis and respiration rate as WT.**

|  | WT | *Δxpk* |
| --- | --- | --- |
| photosynthesis  (O_2_ evolution 𝜇mole *Chla* mg^-1^ h^-1^) | 262.4 ± 8.2 | 248.2 ± 8.9 |
| respiration (O_2_ consumption 𝜇mole *Chla* mg^-1^ h^-1^) | 36.5 ± 1 | 35.7 ± 1.4 |
