# Supplementary material for: An ATP-sensitive phosphoketolase regulates carbon fixation in cyanobacteria
Source: Nat Metab. 2023 Jun 22;5(7):1111–26. doi: 10.1038/s42255-023-00831-w (PMC10365998; doi:10.1038/s42255-023-00831-w)
Supplement: Supplementary file 5 — Cryo-EM data collection, refinement and validation statistics for the XPK structures. [file 42255_2023_831_MOESM5_ESM.docx]

**Supplementary Table 3. Cryo-EM data collection, refinement and validation statistics for the XPK structures**

|  | AMPPNP-bound *Se*XPK  dimer: EMD-35611  PDB 8IO8  dodecamer: EMD-35612  PDB 8IO9 | *Se*XPK  dimer: EMD-35613  PDB 8IOA  dodecamer: EMD-35617  PDB 8IOE | | *B. longum* XPK  dimer: EMD-35610  PDB 8IO7  octamer: EMD-35609  PDB 8IO6 | | |
| --- | --- | --- | --- | --- | --- | --- |
| **Data collection and processing** |  | |  | |  |  |
| Microscope | Titan Krios | | Titan Krios | | Titan Krios |  |
| Detector | K2 | | K2 | | K3 |  |
| Magnification (nominal) | 165,000 | | 165,000 | | 105,000 |  |
| Voltage (kV) | 300 | | 300 | | 300 |  |
| Electron exposure (e–/Å^2^) | 50 | | 50 | | 42 |  |
| Defocus range (μm) | -1.5 ~-2.5 | | -1.5 ~-2.5 | | -1.5 ~-2.5 |  |
| Pixel size (Å) | 0.82 | | 0.82 | | 0.83 |  |
| Symmetry imposed | D6 (C1) | | D6 (C1) | | D4 (C1) |  |
| Initial particle images (no.) | 260,589 | | 228,398 | | 1,261,048 |  |
| Final particle images (no.) | 59,127 | | 30,649 | | 590,627 |  |
| Map final resolution (Å)  FSC threshold | 2.36 (2.17)  0.143 | | 2.86 (2.63)  0.143 | | 2.68 (2.62)  0.143 |  |
| Map resolution range (Å) | 2.30-7.0 (2.0-5.8) | | 2.5-7.0 (2.5-7.0) | | 2.5-9.0 (2.5-7.5) |  |
| Map sharpening B-factor (Å) | -61.5 (-49.0) | | -88.6(-74.3) | | -85.5(-83.4) |  |
|  |  | |  | |  |  |
| **Refinement** |  | |  | |  |  |
| Initial model used (PDB code) | SWISS-Modeler[^18^](#_ENREF_18) | | 8IO8 | | 3AI7[^17^](#_ENREF_17) |  |
| Model composition  Non-hydrogen atoms  Protein residues  Ligands | 75,744 (12,594)  9,456(1,576)  AMPPNP:12 (2)  TPP/Mg^2+^:12 (2) | | 75,420(12,570)  9,456(1,576)  TPP/Mg^2+^:12 (2) | | 51,504(12,876)  6,448(1,612)  TPP/Mg^2+^:12 (2) |  |
| Q-score[^31^](#_ENREF_31) | 0.56 (0.65) | | 0.49(0.57) | | 0.55(0.56) |  |
| Map CC (around atoms) | 0.84(0.91) | | 0.85(0.89) | | 0.82(0.87) |  |
| *B* factors (Å^2^)  Protein  Ligand | 91.82(70.38)  91.52(78.67) | | 134.98(101.99)  174.58(132.99) | | 37.57 (99.97)  26.32 (95.22) |  |
| R.m.s. deviations  Bond lengths (Å)  Bond angles (°) | 0.004 (0.005)  0.505(0.550) | | 0.002 (0.008)  0.508(1.031) | | 0.007(0.006)  1.046(0.978) |  |
| Validation  MolProbity score[^23^](#_ENREF_23)  Clashscore[^20^](#_ENREF_20)  Poor rotamers (%) | 1.69 (1.50)  8.55 (5.25)  0.48(0) | | 1.63(1.55)  8.72 (8.4)  0.50 (0.53) | | 1.65 (1.61)  8.22 (7.93)  0 (0) |  |
| Ramachandran plot  Favored (%)  Allowed (%)  Disallowed (%) | 96.51(96.63)  3.49(3.37)  0(0) | | 97.12(96.44)  2.88(3.56)  0(0) | | 96.78(96.95)  3.22(3.05)  0(0) |  |

The statistics of the focus-refined dimer coordinates are shown in parentheses. The focus-refined map of the individual dimer is deposited separately from the corresponding dodecamer or octamer map and coordinates under different PDB and EMDB codes. The Q-scores with recommended contour levels are illustrated in the wwPDB validation reports.
