# Supplementary material for: An ATP-sensitive phosphoketolase regulates carbon fixation in cyanobacteria
Source: Nat Metab. 2023 Jun 22;5(7):1111–26. doi: 10.1038/s42255-023-00831-w (PMC10365998; doi:10.1038/s42255-023-00831-w)
Supplement: Supplementary file 6 — PDB validation reports for the cryo-EM structures of SeXPK and BlXPK. [file 42255_2023_831_MOESM6_ESM.zip › NATMETAB-A22127983A XPK_PDB-Validation report revision1/D_1300036096_val-report-full_8IO9.pdf]

Full wwPDB EM Validation Report (i)

Mar 24, 2023 – 03:47 PM JST

PDB ID	:	8IO9
EMDB ID	:	EMD-35612
Title	:	Cryo-EM structure of cyanobacteria phosphoketolase complexed with AMPPNP in dodecameric assembly
Deposited on	:	2023-03-10
Resolution	:	2.36 Å(reported)
Based on initial model	:	8IO8

This wwPDB validation report is for manuscript review

This is a Full wwPDB EM Validation Report.

This report is produced by the wwPDB biocuration pipeline after annotation of the structure.

We welcome your comments at *validation@mail.wwpdb.org*
A user guide is available at
https://www.wwpdb.org/validation/2017/EMValidationReportHelp
with specific help available everywhere you see the (i) symbol.

The types of validation reports are described at
http://www.wwpdb.org/validation/2017/FAQs#types.

For Manuscript Review

The following versions of software and data (see references (i)) were used in the production of this report:

EMDB validation analysis	:	0.0.1.dev43
Mogul	:	1.8.5 (274361), CSD as541be (2020)
MolProbity	:	4.02b-467
buster-report	:	1.1.7 (2018)
Percentile statistics	:	20191225.v01 (using entries in the PDB archive December 25th 2019)
MapQ	:	1.9.9
Ideal geometry (proteins)	:	Engh & Huber (2001)

1 Overall quality at a glance (i)

The following experimental techniques were used to determine the structure:
ELECTRON MICROSCOPY

The reported resolution of this entry is 2.36 Å.

Percentile scores (ranging between 0-100) for global validation metrics of the entry are shown in the following graphic. The table shows the number of entries on which the scores are based.

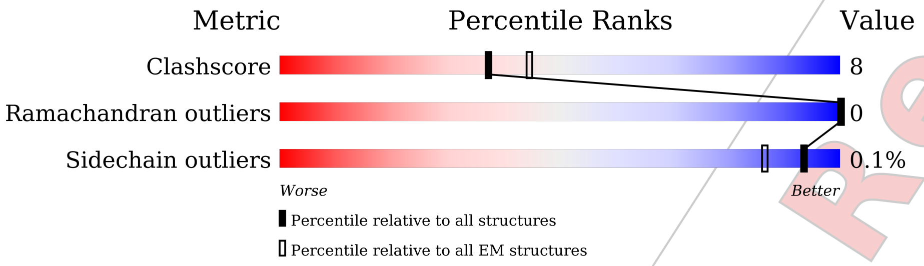

Metric	Whole archive (#Entries)	EM structures (#Entries)
Clashscore	158937	4297
Ramachandran outliers	154571	4023
Sidechain outliers	154315	3826

The table below summarises the geometric issues observed across the polymeric chains and their fit to the map. The red, orange, yellow and green segments of the bar indicate the fraction of residues that contain outliers for >=3, 2, 1 and 0 types of geometric quality criteria respectively. A grey segment represents the fraction of residues that are not modelled. The numeric value for each fraction is indicated below the corresponding segment, with a dot representing fractions <=5% The upper red bar (where present) indicates the fraction of residues that have poor fit to the EM map (all-atom inclusion < 40%). The numeric value is given above the bar.

Mol	Chain	Length	Quality of chain
1	A	796	78% 21% •
1	B	796	80% 19% •
1	C	796	82% 17% •
1	D	796	80% 19% •
1	E	796	81% 18% •
1	F	796	80% 19% •

Continued on next page...

Ideal geometry (DNA, RNA) : Parkinson et al. (1996)
Validation Pipeline (wwPDB-VP) : 2.32.1

For Manuscript Review

Continued from previous page...

Mol	Chain	Length	Quality of chain
1	G	796	81% 18% •
1	H	796	80% 19% •
1	I	796	80% 19% •
1	J	796	80% 19% •
1	K	796	80% 19% •
1	L	796	79% 20% •

For Manuscript Review

2 Entry composition (i)

There are 4 unique types of molecules in this entry. The entry contains 75744 atoms, of which 0 are hydrogens and 0 are deuteriums.

In the tables below, the AltConf column contains the number of residues with at least one atom in alternate conformation and the Trace column contains the number of residues modelled with at most 2 atoms.

- Molecule 1 is a protein called Probable phosphoketolase.

Mol	Chain	Residues	Atoms					AltConf	Trace
			Total	C	N	O	S		
1	A	788	6254	3970	1121	1148	15	1	0
1	B	788	6254	3970	1121	1148	15	1	0
1	C	788	6254	3970	1121	1148	15	1	0
1	D	788	6254	3970	1121	1148	15	1	0
1	E	788	6254	3970	1121	1148	15	1	0
1	F	788	6254	3970	1121	1148	15	1	0
1	G	788	6254	3970	1121	1148	15	1	0
1	H	788	6254	3970	1121	1148	15	1	0
1	I	788	6254	3970	1121	1148	15	1	0
1	J	788	6254	3970	1121	1148	15	1	0
1	K	788	6254	3970	1121	1148	15	1	0
1	L	788	6254	3970	1121	1148	15	1	0

- Molecule 2 is PHOSPHOAMINOPHOSPHONIC ACID-ADENYLATE ESTER (three-letter code: ANP) (formula: $C_{10}H_{17}N_6O_{12}P_3$) (labeled as "Ligand of Interest" by depositor).

For Manuscript Review

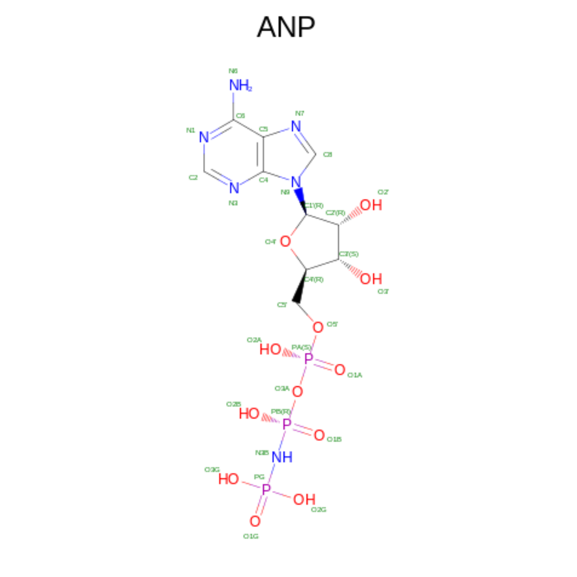

Mol	Chain	Residues	Atoms					AltConf
2	A	1	Total 31	C 10	N 6	O 12	P 3	0
2	B	1	Total 31	C 10	N 6	O 12	P 3	0
2	C	1	Total 31	C 10	N 6	O 12	P 3	0
2	D	1	Total 31	C 10	N 6	O 12	P 3	0
2	E	1	Total 31	C 10	N 6	O 12	P 3	0
2	F	1	Total 31	C 10	N 6	O 12	P 3	0
2	G	1	Total 31	C 10	N 6	O 12	P 3	0
2	H	1	Total 31	C 10	N 6	O 12	P 3	0
2	I	1	Total 31	C 10	N 6	O 12	P 3	0
2	J	1	Total 31	C 10	N 6	O 12	P 3	0
2	K	1	Total 31	C 10	N 6	O 12	P 3	0
2	L	1	Total 31	C 10	N 6	O 12	P 3	0

- Molecule 3 is THIAMINE DIPHOSPHATE (three-letter code: TPP) (formula: $C_{12}H_{19}N_4O_7P_2S$) (labeled as "Ligand of Interest" by depositor).

For Manuscript Review

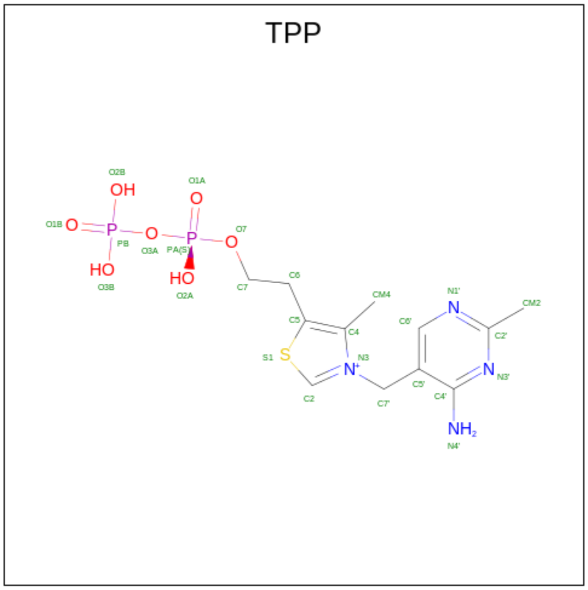

Mol	Chain	Residues	Atoms	AltConf
3	A	1	Total C N O P S<br>26 12 4 7 2 1	0
3	B	1	Total C N O P S<br>26 12 4 7 2 1	0
3	C	1	Total C N O P S<br>26 12 4 7 2 1	0
3	D	1	Total C N O P S<br>26 12 4 7 2 1	0
3	E	1	Total C N O P S<br>26 12 4 7 2 1	0
3	F	1	Total C N O P S<br>26 12 4 7 2 1	0
3	G	1	Total C N O P S<br>26 12 4 7 2 1	0
3	H	1	Total C N O P S<br>26 12 4 7 2 1	0
3	I	1	Total C N O P S<br>26 12 4 7 2 1	0
3	J	1	Total C N O P S<br>26 12 4 7 2 1	0
3	K	1	Total C N O P S<br>26 12 4 7 2 1	0
3	L	1	Total C N O P S<br>26 12 4 7 2 1	0

- Molecule 4 is MAGNESIUM ION (three-letter code: MG) (formula: Mg) (labeled as "Ligand of Interest" by depositor).

For Manuscript Review

Mol	Chain	Residues	Atoms		AltConf
			Total	Mg	
4	A	1	1	1	0
4	B	1	1	1	0
4	C	1	1	1	0
4	D	1	1	1	0
4	E	1	1	1	0
4	F	1	1	1	0
4	G	1	1	1	0
4	H	1	1	1	0
4	I	1	1	1	0
4	J	1	1	1	0
4	K	1	1	1	0
4	L	1	1	1	0

For Manuscript Review

3 Residue-property plots ⓘ

These plots are drawn for all protein, RNA, DNA and oligosaccharide chains in the entry. The first graphic for a chain summarises the proportions of the various outlier classes displayed in the second graphic. The second graphic shows the sequence view annotated by issues in geometry and atom inclusion in map density. Residues are color-coded according to the number of geometric quality criteria for which they contain at least one outlier: green = 0, yellow = 1, orange = 2 and red = 3 or more. A red diamond above a residue indicates a poor fit to the EM map for this residue (all-atom inclusion < 40%). Stretches of 2 or more consecutive residues without any outlier are shown as a green connector. Residues present in the sample, but not in the model, are shown in grey.

• Molecule 1: Probable phosphoketolase

Chain A:

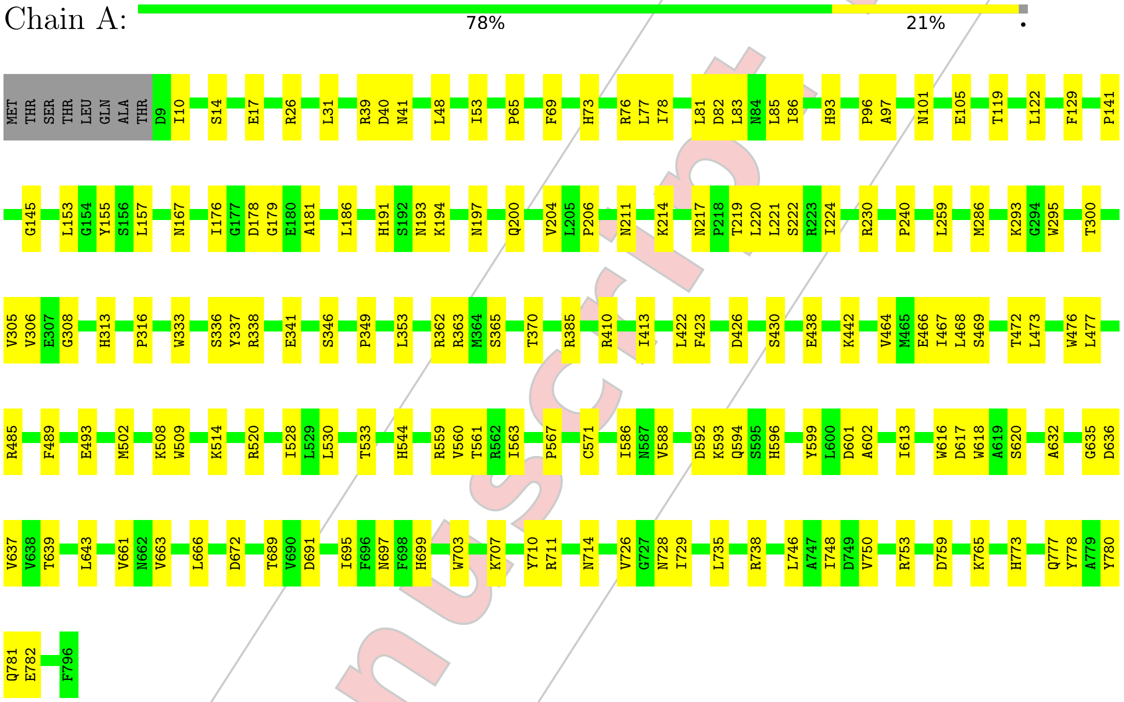

• Molecule 1: Probable phosphoketolase

Chain B:

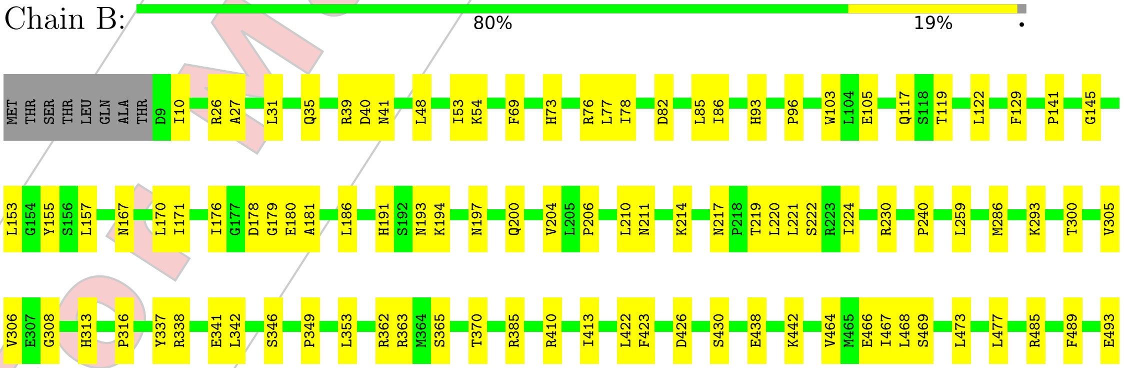

For Manuscript Review

M502 K508 W509 K514 R515 E516 R520 I528 L529 L530 T533 S543 H544 R559 V560 T561 R562 I563 N570 I586 N587 V588 D592 K593 Q594 S595 H596 L600 D601 A602 A632 G635 T639 L643 V661 L666 D672 T689 V690 D691

H699 P702 W703 K707 Y710 R711 N714 V726 G727 N728 I729 L735 R738 I748 D759 K765 H773 Q777 Y778 A779 Y780 Q781 E782 F796

• Molecule 1: Probable phosphoketolase

Chain C: 82% 17% •

MET THR SER THR LEU GLN ALA THR D9 I10 R26 L31 R39 D40 N41 L48 I53 F69 H73 R76 L77 D82 L85 I86 H93 P96 E105 T119 L122 F129 P141 L153 G154 Y155 S156 L157 N167 L170 I171

I176 G177 D178 G179 E180 A181 L186 H191 S192 N193 K194 N197 Q200 V204 L205 P206 L210 N211 G212 Y213 K214 N217 P218 T219 L220 L221 S222 R223 I224 R230 P240 L259 M286 K293 T300 V305 V306 E307 G308 H313 P316 Y337 R338

E341 S346 P349 L353 R362 R363 T370 R385 R410 I413 L422 F423 D426 E438 K442 V464 M465 E466 I467 L468 S469 L473 L477 R485 F489 E493 M502 K508 W509 K514 R520 I528 L529 L530 T533

H544 R559 V560 T561 R562 I563 D568 C571 I586 N587 V588 D592 L600 D601 A602 I613 D617 A632 V661 N662 V663 D672 T689 V690 D691 N697 F698 H699 W703 K707 Y710 R711 N714 V726 G727 N728 I729 L735 R738

L746 A747 I748 D759 K765 H773 Q777 Y778 A779 Y780 Q781 E782 F796

• Molecule 1: Probable phosphoketolase

Chain D: 80% 19% •

MET THR SER THR LEU GLN ALA THR D9 I10 R26 A27 L31 R39 D40 N41 L48 I53 P65 F69 H73 R76 L77 D82 L85 I86 H93 P96 W103 L104 E105 Q117 S118 T119 L122 F129 P141 E142 L153 G154 Y155

S156 L157 D166 N167 L170 I171 I176 G177 D178 G179 E180 A181 L186 H191 S192 N193 K194 N197 Q200 V204 L205 P206 L210 N211 K214 N217 P218 T219 L220 L221 S222 R223 I224 R230 P240 L259 M286 K293 G294 W295 T300 V305

V306 E307 G308 H313 P316 Y337 R338 E341 L342 S346 P349 L353 R362 R363 T370 R385 R410 I413 L422 F423 D426 E438 K442 V464 M465 E466 I467 L468 S469 L473 L477 Y480 R485 F489 E493 M502

K508 W509 K514 V517 R520 I528 L529 L530 T533 S543 H544 N555 R559 V560 T561 R562 I563 N570 I586 N587 V588 D592 K593 Q594 S595 H596 L600 D601 A602 I613 D617 A632 V661 N662 V663 L666 D672 T689 V690 D691

N697 F698 H699 P702 W703 K707 Y710 R711 N714 V726 G727 N728 I729 L735 R738 L746 A747 I748 D759 K765 H773 Q777 Y778 A779 Y780 Q781 E782 F796

• Molecule 1: Probable phosphoketolase

Chain E: 81% 18% •

For Manuscript Review

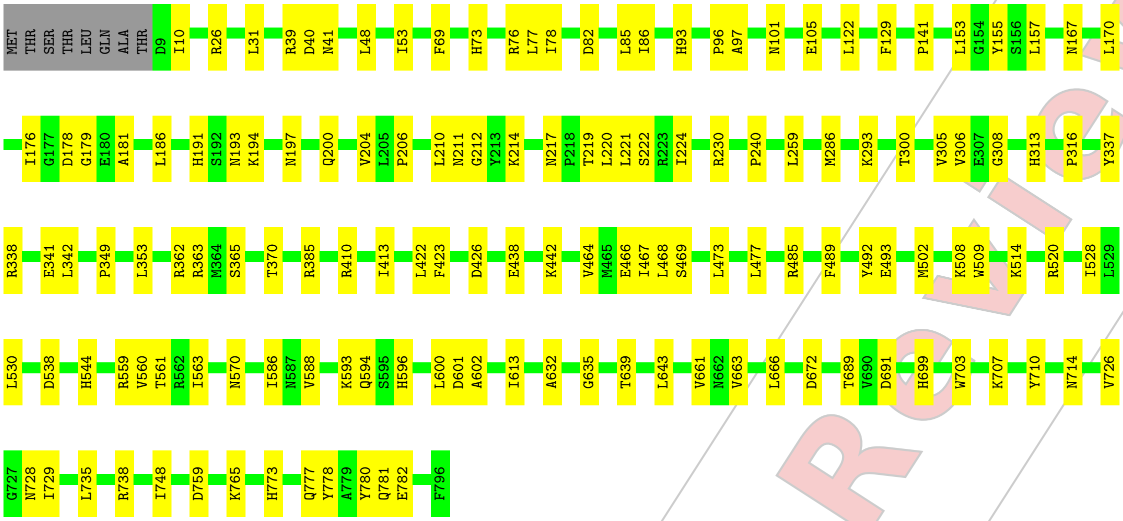

- Molecule 1: Probable phosphoketolase

Chain F:

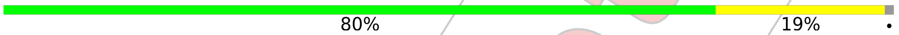

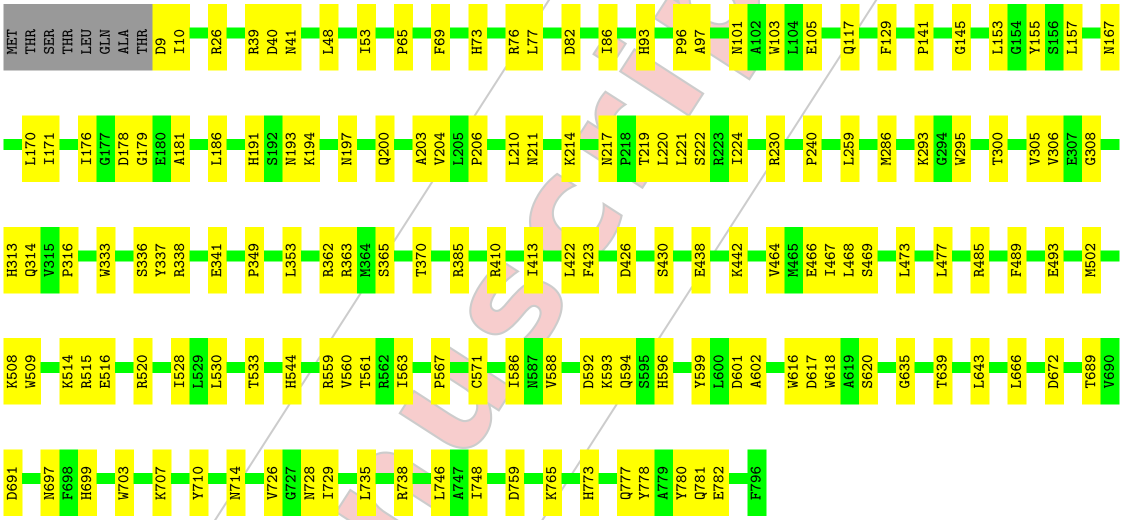

- Molecule 1: Probable phosphoketolase

Chain G:

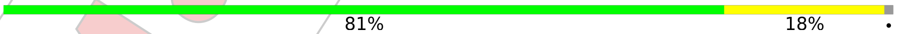

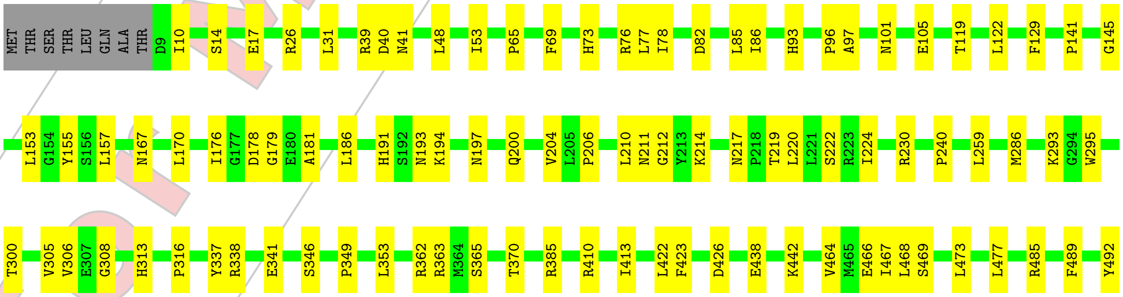

For Manuscript Review

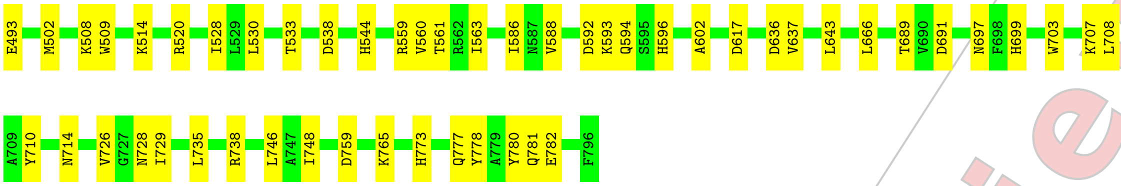

• Molecule 1: Probable phosphoketolase

Chain H:

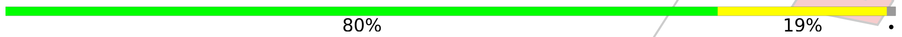

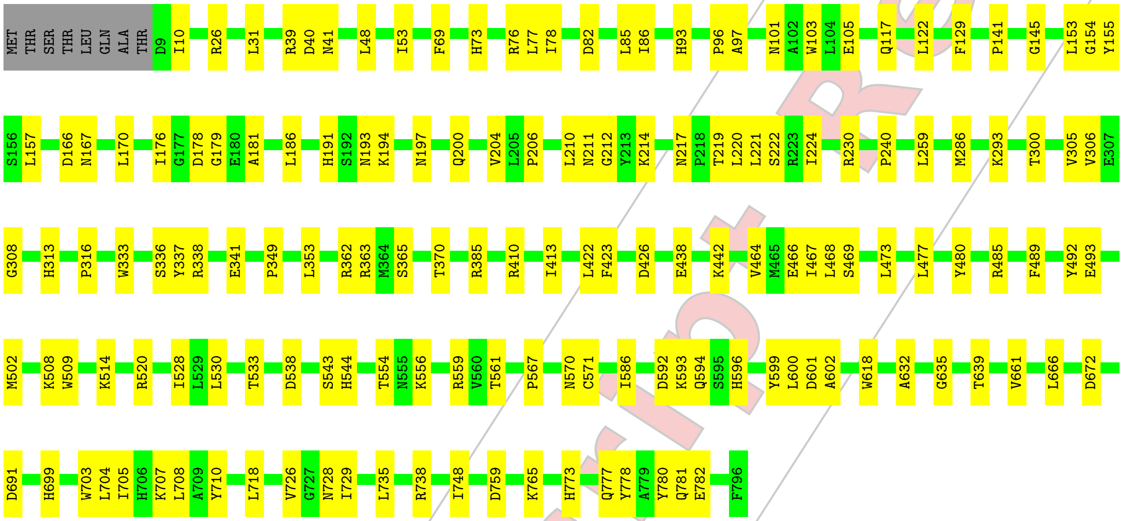

• Molecule 1: Probable phosphoketolase

Chain I:

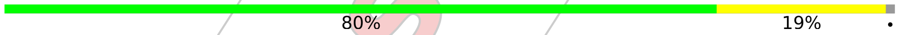

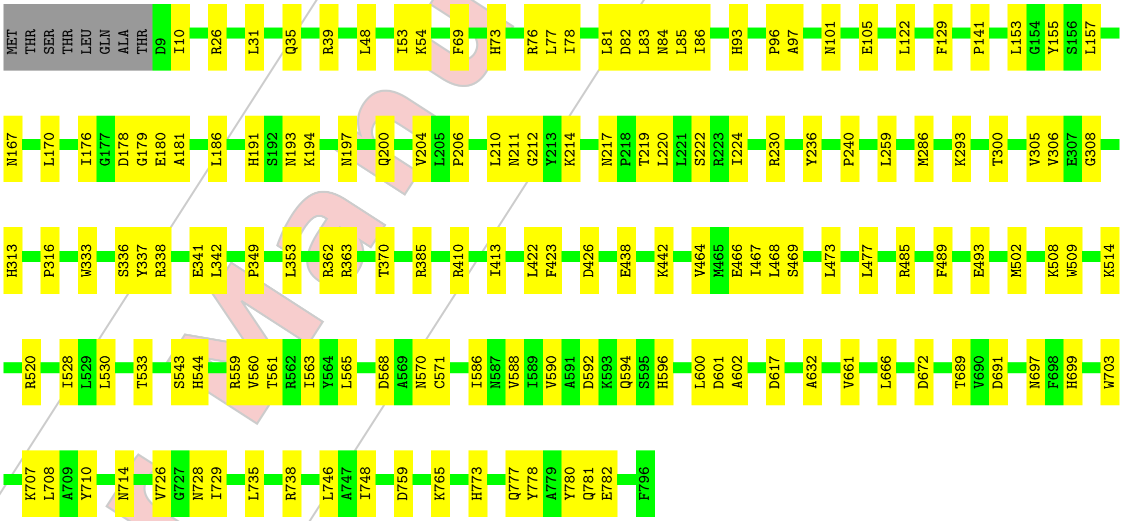

• Molecule 1: Probable phosphoketolase

Chain J:

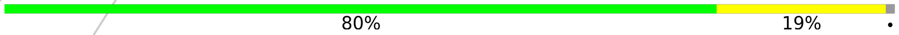

For Manuscript Review

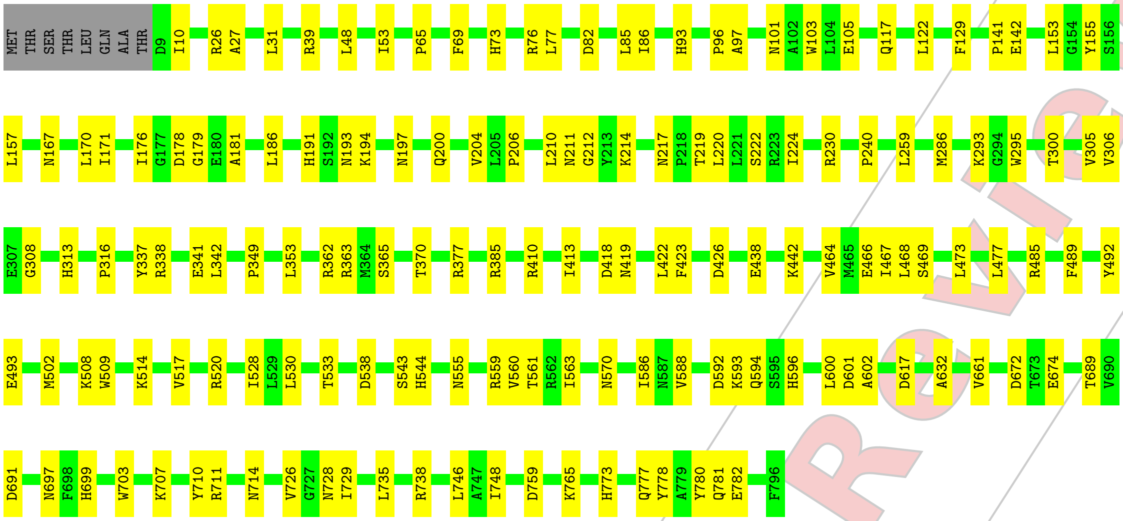

- Molecule 1: Probable phosphoketolase

Chain K:

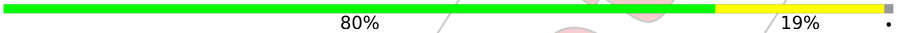

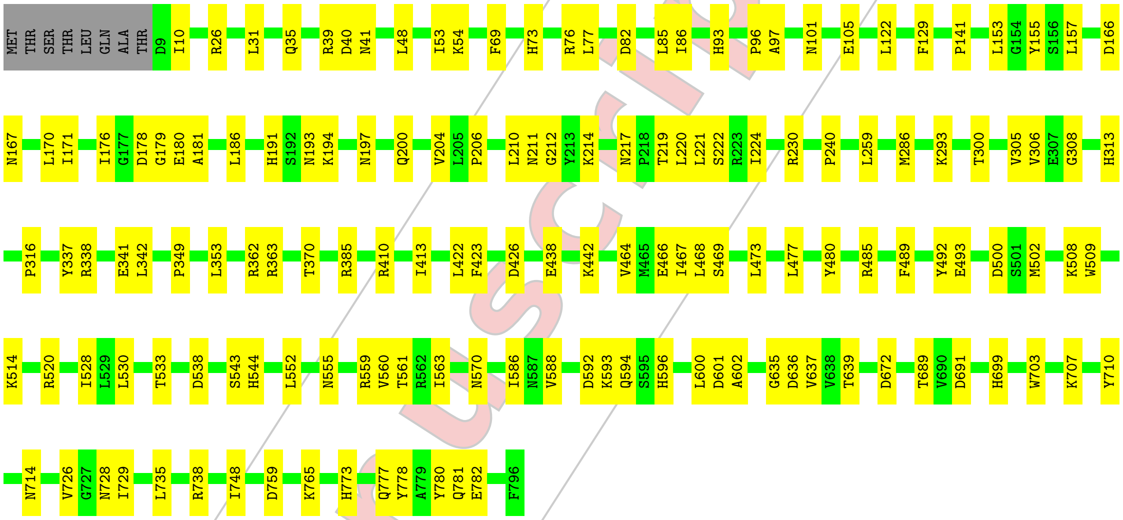

- Molecule 1: Probable phosphoketolase

Chain L:

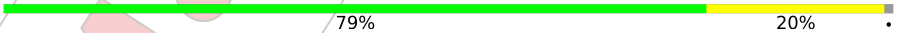

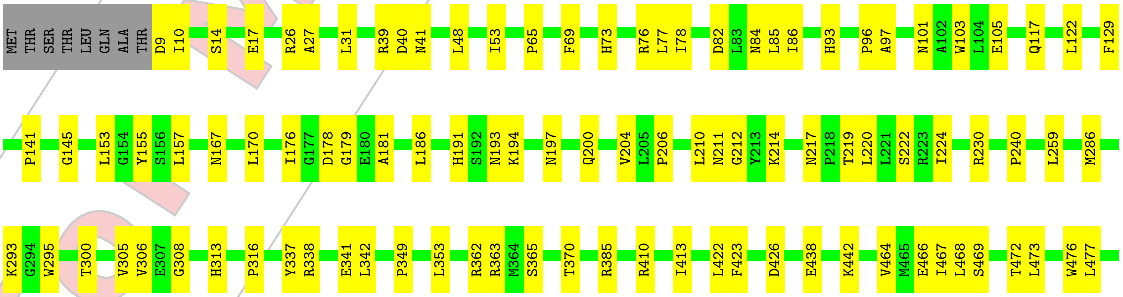

For Manuscript Review

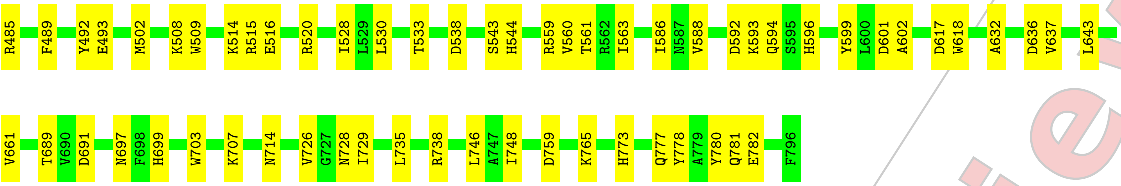

For Manuscript Review

4 Experimental information (i)

Property	Value	Source
EM reconstruction method	SINGLE PARTICLE	Depositor
Imposed symmetry	POINT, Not provided	
Number of particles used	59127	Depositor
Resolution determination method	FSC 0.143 CUT-OFF	Depositor
CTF correction method	PHASE FLIPPING AND AMPLITUDE CORRECTION	Depositor
Microscope	FEI TITAN KRIOS	Depositor
Voltage (kV)	300	Depositor
Electron dose ($e^-/Å^2$)	59	Depositor
Minimum defocus (nm)	1500	Depositor
Maximum defocus (nm)	2500	Depositor
Magnification	165000	Depositor
Image detector	GATAN K3 BIOQUANTUM (6k x 4k)	Depositor
Maximum map value	0.983	Depositor
Minimum map value	-0.338	Depositor
Average map value	0.005	Depositor
Map value standard deviation	0.050	Depositor
Recommended contour level	0.12	Depositor
Map size (Å)	314.88, 314.88, 314.88	wwPDB
Map dimensions	384, 384, 384	wwPDB
Map angles (°)	90.0, 90.0, 90.0	wwPDB
Pixel spacing (Å)	0.82, 0.82, 0.82	Depositor

For Manuscript Review

5 Model quality (i)

5.1 Standard geometry (i)

Bond lengths and bond angles in the following residue types are not validated in this section: TPP, ANP, MG

The Z score for a bond length (or angle) is the number of standard deviations the observed value is removed from the expected value. A bond length (or angle) with $|Z| > 5$ is considered an outlier worth inspection. RMSZ is the root-mean-square of all Z scores of the bond lengths (or angles).

Mol	Chain	Bond lengths		Bond angles	
		RMSZ	$\#\|Z\| > 5$	RMSZ	$\#\|Z\| > 5$
1	A	0.29	0/6432	0.47	0/8775
1	B	0.29	0/6432	0.48	0/8775
1	C	0.29	0/6432	0.48	0/8775
1	D	0.29	0/6432	0.48	0/8775
1	E	0.29	0/6432	0.47	0/8775
1	F	0.29	0/6432	0.48	0/8775
1	G	0.29	0/6432	0.47	0/8775
1	H	0.29	0/6432	0.47	0/8775
1	I	0.29	0/6432	0.48	0/8775
1	J	0.29	0/6432	0.48	0/8775
1	K	0.29	0/6432	0.48	0/8775
1	L	0.29	0/6432	0.47	0/8775
All	All	0.29	0/77184	0.48	0/105300

There are no bond length outliers.

There are no bond angle outliers.

There are no chirality outliers.

There are no planarity outliers.

5.2 Too-close contacts (i)

In the following table, the Non-H and H(model) columns list the number of non-hydrogen atoms and hydrogen atoms in the chain respectively. The H(added) column lists the number of hydrogen atoms added and optimized by MolProbity. The Clashes column lists the number of clashes within the asymmetric unit, whereas Symm-Clashes lists symmetry-related clashes.

Mol	Chain	Non-H	H(model)	H(added)	Clashes	Symm-Clashes
1	A	6254	0	6052	110	0

Continued on next page...

For Manuscript Review

Continued from previous page...

Mol	Chain	Non-H	H(model)	H(added)	Clashes	Symm-Clashes
1	B	6254	0	6052	106	0
1	C	6254	0	6052	96	0
1	D	6254	0	6052	106	0
1	E	6254	0	6052	100	0
1	F	6254	0	6052	105	0
1	G	6254	0	6052	100	0
1	H	6254	0	6052	106	0
1	I	6254	0	6052	119	0
1	J	6254	0	6052	109	0
1	K	6254	0	6052	105	0
1	L	6254	0	6052	116	0
2	A	31	0	13	3	0
2	B	31	0	12	1	0
2	C	31	0	13	2	0
2	D	31	0	12	1	0
2	E	31	0	13	2	0
2	F	31	0	13	2	0
2	G	31	0	13	3	0
2	H	31	0	13	3	0
2	I	31	0	13	2	0
2	J	31	0	13	3	0
2	K	31	0	13	0	0
2	L	31	0	13	3	0
3	A	26	0	16	6	0
3	B	26	0	16	4	0
3	C	26	0	16	5	0
3	D	26	0	16	4	0
3	E	26	0	16	5	0
3	F	26	0	16	5	0
3	G	26	0	16	5	0
3	H	26	0	16	4	0
3	I	26	0	16	5	0
3	J	26	0	16	4	0
3	K	26	0	16	5	0
3	L	26	0	16	5	0
4	A	1	0	0	0	0
4	B	1	0	0	0	0
4	C	1	0	0	0	0
4	D	1	0	0	0	0
4	E	1	0	0	0	0
4	F	1	0	0	0	0
4	G	1	0	0	0	0

Continued on next page...

For Manuscript Review

Continued from previous page...

Mol	Chain	Non-H	H(model)	H(added)	Clashes	Symm-Clashes
4	H	1	0	0	0	0
4	I	1	0	0	0	0
4	J	1	0	0	0	0
4	K	1	0	0	0	0
4	L	1	0	0	0	0
All	All	75744	0	72970	1217	0

The all-atom clashscore is defined as the number of clashes found per 1000 atoms (including hydrogen atoms). The all-atom clashscore for this structure is 8.

All (1217) close contacts within the same asymmetric unit are listed below, sorted by their clash magnitude.

Atom-1	Atom-2	Interatomic distance (Å)	Clash overlap (Å)
1:I:666:LEU:HD21	1:I:708:LEU:CD1	1.51	1.36
1:I:666:LEU:CD2	1:I:708:LEU:HD11	1.55	1.34
1:I:666:LEU:CD2	1:I:708:LEU:CD1	2.13	1.21
1:C:703[C]:TRP:HH2	1:D:555:ASN:OD1	1.33	1.10
1:A:178:ASP:CG	1:A:219:THR:HG21	1.73	1.09
1:F:171:ILE:HD12	1:F:203:ALA:HB3	1.26	1.07
1:I:193:ASN:OD1	1:I:194:LYS:HD3	1.58	1.03
1:I:703[B]:TRP:HH2	1:J:555:ASN:OD1	1.43	0.99
1:L:178:ASP:CG	1:L:219:THR:HG21	1.89	0.93
1:F:178:ASP:CG	1:F:219:THR:HG21	1.89	0.93
1:G:178:ASP:CG	1:G:219:THR:HG21	1.89	0.92
1:C:703[C]:TRP:CH2	1:D:555:ASN:OD1	2.22	0.92
1:F:171:ILE:CD1	1:F:203:ALA:HB3	2.00	0.91
1:H:178:ASP:CG	1:H:219:THR:HG21	1.92	0.90
1:B:178:ASP:CG	1:B:219:THR:HG21	1.92	0.90
1:D:178:ASP:CG	1:D:219:THR:HG21	1.93	0.89
1:I:178:ASP:CG	1:I:219:THR:HG21	1.92	0.88
1:K:178:ASP:CG	1:K:219:THR:HG21	1.95	0.87
1:E:178:ASP:CG	1:E:219:THR:HG21	1.94	0.87
1:I:703[B]:TRP:CH2	1:J:555:ASN:OD1	2.28	0.87
1:I:666:LEU:HD23	1:I:708:LEU:CD1	2.03	0.87
1:J:178:ASP:CG	1:J:219:THR:HG21	1.94	0.87
1:C:178:ASP:CG	1:C:219:THR:HG21	1.97	0.85
1:I:666:LEU:HD23	1:I:708:LEU:HD13	1.60	0.83
1:A:472:THR:HG22	1:A:476:TRP:CE2	2.16	0.80
1:L:472:THR:HG22	1:L:476:TRP:CE2	2.17	0.80
1:I:193:ASN:OD1	1:I:194:LYS:CD	2.32	0.78
1:A:178:ASP:OD2	1:A:219:THR:HG21	1.85	0.77

Continued on next page...

For Manuscript Review

Continued from previous page...

Atom-1	Atom-2	Interatomic distance (Å)	Clash overlap (Å)
1:K:85:LEU:HD23	1:K:171:ILE:HB	1.66	0.77
1:I:666:LEU:CD2	1:I:708:LEU:HD13	2.15	0.76
1:I:666:LEU:HD21	1:I:708:LEU:HD11	0.78	0.74
1:J:193:ASN:OD1	1:J:194:LYS:NZ	2.19	0.73
1:H:567:PRO:HD2	1:H:571:CYS:SG	2.28	0.73
1:F:338:ARG:NH1	1:F:341:GLU:OE2	2.22	0.72
1:A:119:THR:HG23	1:A:346:SER:O	1.89	0.72
1:F:193:ASN:OD1	1:F:194:LYS:NZ	2.21	0.72
1:G:119:THR:HG23	1:G:346:SER:O	1.89	0.72
1:A:338:ARG:NH1	1:A:341:GLU:OE2	2.23	0.71
1:E:193:ASN:OD1	1:E:194:LYS:NZ	2.20	0.71
1:A:178:ASP:OD1	1:A:179:GLY:N	2.24	0.71
1:H:338:ARG:NH1	1:H:341:GLU:OE2	2.24	0.71
1:D:119:THR:HG23	1:D:346:SER:O	1.91	0.70
1:I:193:ASN:OD1	1:I:194:LYS:N	2.25	0.70
1:C:571:CYS:SG	1:C:600:LEU:HD12	2.31	0.70
1:B:119:THR:HG23	1:B:346:SER:O	1.92	0.70
1:G:508:LYS:HE2	1:H:544:HIS:HB2	1.74	0.70
1:A:508:LYS:HE2	1:B:544:HIS:HB2	1.74	0.69
1:C:338:ARG:NH1	1:C:341:GLU:OE2	2.25	0.69
1:A:567:PRO:HD2	1:A:571:CYS:SG	2.32	0.69
1:C:119:THR:HG23	1:C:346:SER:O	1.92	0.69
1:G:206:PRO:HB2	1:G:286:MET:HG3	1.75	0.69
1:C:508:LYS:HE2	1:D:544:HIS:HB2	1.75	0.69
1:F:567:PRO:HD2	1:F:571:CYS:SG	2.32	0.69
1:E:206:PRO:HB2	1:E:286:MET:HG3	1.75	0.69
1:I:508:LYS:HE2	1:J:544:HIS:HB2	1.75	0.69
1:B:206:PRO:HB2	1:B:286:MET:HG3	1.75	0.68
1:G:193:ASN:OD1	1:G:194:LYS:NZ	2.19	0.68
1:G:514:LYS:NZ	1:G:559:ARG:O	2.26	0.68
1:H:410:ARG:NH1	1:H:438:GLU:OE1	2.25	0.68
1:K:193:ASN:OD1	1:K:194:LYS:NZ	2.19	0.68
1:G:338:ARG:NH1	1:G:341:GLU:OE2	2.24	0.68
1:K:544:HIS:HB2	1:L:508:LYS:HE2	1.76	0.68
1:A:206:PRO:HB2	1:A:286:MET:HG3	1.75	0.68
1:L:206:PRO:HB2	1:L:286:MET:HG3	1.76	0.68
1:C:544:HIS:HB2	1:D:508:LYS:HE2	1.76	0.68
1:E:39:ARG:NH2	1:F:781:GLN:O	2.27	0.68
1:B:178:ASP:OD1	1:B:219:THR:HG21	1.93	0.68
1:K:508:LYS:HE2	1:L:544:HIS:HB2	1.75	0.68
1:E:508:LYS:HE2	1:F:544:HIS:HB2	1.74	0.68

Continued on next page...

For Manuscript Review

Continued from previous page...

Atom-1	Atom-2	Interatomic distance (Å)	Clash overlap (Å)
1:I:514:LYS:NZ	1:I:559:ARG:O	2.28	0.68
1:L:472:THR:CG2	1:L:476:TRP:CZ2	2.77	0.68
1:I:571:CYS:SG	1:I:600:LEU:HD12	2.34	0.67
1:A:472:THR:CG2	1:A:476:TRP:CZ2	2.77	0.67
1:J:338:ARG:NH1	1:J:341:GLU:OE2	2.27	0.67
1:H:193:ASN:OD1	1:H:194:LYS:NZ	2.20	0.67
1:I:206:PRO:HB2	1:I:286:MET:HG3	1.75	0.67
1:C:206:PRO:HB2	1:C:286:MET:HG3	1.76	0.67
1:D:206:PRO:HB2	1:D:286:MET:HG3	1.76	0.67
1:I:178:ASP:OD1	1:I:219:THR:HG21	1.95	0.67
1:L:178:ASP:OD1	1:L:219:THR:HG21	1.94	0.67
1:E:178:ASP:OD1	1:E:219:THR:HG21	1.93	0.67
1:G:178:ASP:OD1	1:G:219:THR:HG21	1.94	0.67
1:E:544:HIS:HB2	1:F:508:LYS:HE2	1.76	0.67
1:F:206:PRO:HB2	1:F:286:MET:HG3	1.76	0.67
1:D:410:ARG:NH1	1:D:438:GLU:OE1	2.25	0.67
1:H:178:ASP:OD1	1:H:219:THR:HG21	1.93	0.67
1:H:206:PRO:HB2	1:H:286:MET:HG3	1.75	0.67
1:I:544:HIS:HB2	1:J:508:LYS:HE2	1.76	0.67
1:L:338:ARG:NH1	1:L:341:GLU:OE2	2.26	0.67
1:B:514:LYS:NZ	1:B:559:ARG:O	2.28	0.66
1:J:410:ARG:NH1	1:J:438:GLU:OE1	2.25	0.66
1:K:178:ASP:OD1	1:K:219:THR:HG21	1.94	0.66
1:K:206:PRO:HB2	1:K:286:MET:HG3	1.76	0.66
1:A:544:HIS:HB2	1:B:508:LYS:HE2	1.76	0.66
1:F:514:LYS:NZ	1:F:559:ARG:O	2.28	0.66
1:G:181:ALA:HA	1:G:186:LEU:HD22	1.77	0.66
1:L:193:ASN:OD1	1:L:194:LYS:NZ	2.21	0.66
1:A:514:LYS:NZ	1:A:559:ARG:O	2.28	0.66
1:B:410:ARG:NH1	1:B:438:GLU:OE1	2.25	0.66
1:F:178:ASP:OD1	1:F:219:THR:HG21	1.94	0.66
1:J:206:PRO:HB2	1:J:286:MET:HG3	1.76	0.66
1:E:781:GLN:O	1:F:39:ARG:NH2	2.27	0.66
1:H:514:LYS:NZ	1:H:559:ARG:O	2.29	0.66
1:J:514:LYS:NZ	1:J:559:ARG:O	2.29	0.66
1:G:544:HIS:HB2	1:H:508:LYS:HE2	1.76	0.66
1:I:338:ARG:NH1	1:I:341:GLU:OE2	2.28	0.66
1:L:514:LYS:NZ	1:L:559:ARG:O	2.29	0.66
1:E:514:LYS:NZ	1:E:559:ARG:O	2.30	0.65
1:K:338:ARG:NH1	1:K:341:GLU:OE2	2.28	0.65
1:C:514:LYS:NZ	1:C:559:ARG:O	2.29	0.65

Continued on next page...

For Manuscript Review

Continued from previous page...

Atom-1	Atom-2	Interatomic distance (Å)	Clash overlap (Å)
1:I:181:ALA:HA	1:I:186:LEU:HD22	1.78	0.65
1:J:181:ALA:HA	1:J:186:LEU:HD22	1.78	0.65
1:C:410:ARG:NH1	1:C:438:GLU:OE1	2.26	0.65
1:D:178:ASP:OD1	1:D:219:THR:HG21	1.95	0.65
1:J:178:ASP:OD1	1:J:219:THR:HG21	1.97	0.65
1:L:181:ALA:HA	1:L:186:LEU:HD22	1.78	0.65
1:D:514:LYS:NZ	1:D:559:ARG:O	2.29	0.65
1:K:514:LYS:NZ	1:K:559:ARG:O	2.30	0.65
1:L:410:ARG:NH1	1:L:438:GLU:OE1	2.25	0.65
1:C:781:GLN:O	1:D:39:ARG:NH2	2.26	0.65
1:I:410:ARG:NH1	1:I:438:GLU:OE1	2.26	0.65
1:C:178:ASP:OD1	1:C:219:THR:HG21	1.96	0.64
1:G:410:ARG:NH1	1:G:438:GLU:OE1	2.26	0.64
1:L:472:THR:HG22	1:L:476:TRP:CZ2	2.32	0.64
1:I:781:GLN:O	1:J:39:ARG:NH2	2.26	0.64
1:A:39:ARG:NH2	1:B:781:GLN:O	2.27	0.64
1:B:230:ARG:HG3	1:B:240:PRO:HG2	1.80	0.64
1:F:181:ALA:HA	1:F:186:LEU:HD22	1.77	0.64
1:A:781:GLN:O	1:B:39:ARG:NH2	2.28	0.64
1:C:39:ARG:NH2	1:D:781:GLN:O	2.26	0.64
1:F:410:ARG:NH1	1:F:438:GLU:OE1	2.25	0.64
1:A:181:ALA:HA	1:A:186:LEU:HD22	1.77	0.64
1:K:26:ARG:NH1	1:K:337:TYR:O	2.31	0.64
1:E:26:ARG:NH1	1:E:337:TYR:O	2.31	0.64
1:H:181:ALA:HA	1:H:186:LEU:HD22	1.80	0.64
1:I:39:ARG:NH2	1:J:781:GLN:O	2.26	0.63
1:E:410:ARG:NH1	1:E:438:GLU:OE1	2.26	0.63
1:C:181:ALA:HA	1:C:186:LEU:HD22	1.78	0.63
1:A:472:THR:HG22	1:A:476:TRP:CZ2	2.33	0.63
1:B:338:ARG:NH1	1:B:341:GLU:OE2	2.27	0.63
1:D:338:ARG:NH1	1:D:341:GLU:OE2	2.27	0.63
1:J:86:ILE:HD12	1:J:370:THR:HG23	1.81	0.63
1:L:230:ARG:HG3	1:L:240:PRO:HG2	1.80	0.63
1:D:26:ARG:NH1	1:D:337:TYR:O	2.32	0.63
1:K:39:ARG:NH2	1:L:781:GLN:O	2.27	0.63
1:L:26:ARG:NH1	1:L:337:TYR:O	2.32	0.63
1:J:26:ARG:NH1	1:J:337:TYR:O	2.32	0.63
1:C:86:ILE:HD12	1:C:370:THR:HG23	1.81	0.63
1:D:230:ARG:HG3	1:D:240:PRO:HG2	1.81	0.63
1:H:230:ARG:HG3	1:H:240:PRO:HG2	1.80	0.63
1:A:410:ARG:NH1	1:A:438:GLU:OE1	2.26	0.63

Continued on next page...

For Manuscript Review

Continued from previous page...

Atom-1	Atom-2	Interatomic distance (Å)	Clash overlap (Å)
1:J:69:PHE:O	1:J:73:HIS:ND1	2.31	0.62
1:J:413:ILE:HD11	1:J:422:LEU:HD23	1.82	0.62
1:D:181:ALA:HA	1:D:186:LEU:HD22	1.79	0.62
1:K:781:GLN:O	1:L:39:ARG:NH2	2.28	0.62
1:E:230:ARG:HG3	1:E:240:PRO:HG2	1.82	0.62
1:K:181:ALA:HA	1:K:186:LEU:HD22	1.81	0.62
1:B:181:ALA:HA	1:B:186:LEU:HD22	1.80	0.62
1:E:181:ALA:HA	1:E:186:LEU:HD22	1.80	0.62
1:L:84:ASN:C	1:L:85:LEU:HD12	2.19	0.62
1:E:338:ARG:NH1	1:E:341:GLU:OE2	2.28	0.62
1:F:230:ARG:HG3	1:F:240:PRO:HG2	1.82	0.62
1:G:781:GLN:O	1:H:39:ARG:NH2	2.27	0.62
1:B:26:ARG:NH1	1:B:337:TYR:O	2.32	0.62
1:C:230:ARG:HG3	1:C:240:PRO:HG2	1.82	0.62
1:K:410:ARG:NH1	1:K:438:GLU:OE1	2.26	0.61
1:C:69:PHE:O	1:C:73:HIS:ND1	2.31	0.61
1:D:86:ILE:HD12	1:D:370:THR:HG23	1.83	0.61
1:D:69:PHE:O	1:D:73:HIS:ND1	2.31	0.61
1:H:69:PHE:O	1:H:73:HIS:ND1	2.31	0.61
1:H:86:ILE:HD12	1:H:370:THR:HG23	1.82	0.61
1:K:69:PHE:O	1:K:73:HIS:ND1	2.31	0.61
1:K:86:ILE:HD12	1:K:370:THR:HG23	1.83	0.61
1:K:230:ARG:HG3	1:K:240:PRO:HG2	1.82	0.61
1:A:230:ARG:HG3	1:A:240:PRO:HG2	1.83	0.61
1:I:86:ILE:HD12	1:I:370:THR:HG23	1.83	0.61
1:E:86:ILE:HD12	1:E:370:THR:HG23	1.83	0.61
1:I:26:ARG:NH1	1:I:337:TYR:O	2.34	0.61
1:J:230:ARG:HG3	1:J:240:PRO:HG2	1.81	0.61
1:G:230:ARG:HG3	1:G:240:PRO:HG2	1.83	0.60
1:D:702:PRO:HB2	2:D:901:ANP:H4'	1.83	0.60
1:A:69:PHE:O	1:A:73:HIS:ND1	2.30	0.60
1:A:211:ASN:ND2	3:A:902:TPP:O2B	2.33	0.60
1:F:69:PHE:O	1:F:73:HIS:ND1	2.30	0.60
1:I:230:ARG:HG3	1:I:240:PRO:HG2	1.82	0.60
1:A:178:ASP:OD2	1:A:219:THR:CG2	2.49	0.60
1:I:84:ASN:C	1:I:85:LEU:HD12	2.22	0.60
1:G:39:ARG:NH2	1:H:781:GLN:O	2.27	0.60
1:F:211:ASN:ND2	3:F:902:TPP:O1B	2.33	0.60
1:F:86:ILE:HD12	1:F:370:THR:HG23	1.83	0.59
1:J:418:ASP:OD1	1:J:418:ASP:N	2.31	0.59
1:L:86:ILE:HD12	1:L:370:THR:HG23	1.84	0.59

Continued on next page...

For Manuscript Review

Continued from previous page...

Atom-1	Atom-2	Interatomic distance (Å)	Clash overlap (Å)
1:I:413:ILE:HD11	1:I:422:LEU:HD23	1.85	0.59
1:L:472:THR:CG2	1:L:476:TRP:CE2	2.85	0.59
1:G:211:ASN:ND2	3:G:902:TPP:O2B	2.33	0.58
1:C:385:ARG:HG2	1:C:602:ALA:HB1	1.85	0.58
1:G:413:ILE:HD11	1:G:422:LEU:HD23	1.86	0.58
1:H:538:ASP:OD2	1:H:593:LYS:NZ	2.34	0.58
1:A:86:ILE:HD12	1:A:370:THR:HG23	1.85	0.58
1:A:472:THR:CG2	1:A:476:TRP:CE2	2.84	0.58
1:J:193:ASN:OD1	1:J:194:LYS:N	2.37	0.58
1:L:413:ILE:HD11	1:L:422:LEU:HD23	1.86	0.58
1:B:86:ILE:HD12	1:B:370:THR:HG23	1.86	0.58
1:H:385:ARG:HG2	1:H:602:ALA:HB1	1.86	0.58
1:I:560:VAL:HG13	1:I:561:THR:HG23	1.86	0.58
1:G:538:ASP:OD2	1:G:593:LYS:NZ	2.34	0.58
1:K:385:ARG:HG2	1:K:602:ALA:HB1	1.86	0.58
1:C:413:ILE:HD11	1:C:422:LEU:HD23	1.85	0.58
1:E:385:ARG:HG2	1:E:602:ALA:HB1	1.86	0.57
1:G:86:ILE:HD12	1:G:370:THR:HG23	1.84	0.57
1:I:342:LEU:HD12	1:I:353:LEU:HD11	1.86	0.57
1:B:385:ARG:HG2	1:B:602:ALA:HB1	1.87	0.57
1:D:385:ARG:HG2	1:D:602:ALA:HB1	1.87	0.57
1:E:69:PHE:O	1:E:73:HIS:ND1	2.31	0.57
1:J:560:VAL:HG13	1:J:561:THR:HG23	1.86	0.57
1:A:385:ARG:HG2	1:A:602:ALA:HB1	1.87	0.57
1:E:193:ASN:OD1	1:E:194:LYS:N	2.38	0.57
1:K:211:ASN:ND2	3:K:902:TPP:O2B	2.32	0.57
1:D:413:ILE:HD11	1:D:422:LEU:HD23	1.86	0.57
1:E:413:ILE:HD11	1:E:422:LEU:HD23	1.86	0.57
1:I:385:ARG:HG2	1:I:602:ALA:HB1	1.85	0.57
1:J:385:ARG:HG2	1:J:602:ALA:HB1	1.87	0.57
1:K:413:ILE:HD11	1:K:422:LEU:HD23	1.86	0.57
1:B:413:ILE:HD11	1:B:422:LEU:HD23	1.86	0.57
1:A:413:ILE:HD11	1:A:422:LEU:HD23	1.86	0.56
1:F:385:ARG:HG2	1:F:602:ALA:HB1	1.87	0.56
1:E:211:ASN:ND2	3:E:902:TPP:O2B	2.32	0.56
1:F:193:ASN:OD1	1:F:194:LYS:N	2.38	0.56
1:B:69:PHE:O	1:B:73:HIS:ND1	2.31	0.56
1:G:93:HIS:NE2	3:G:902:TPP:O3B	2.37	0.56
1:H:211:ASN:ND2	3:H:902:TPP:O1B	2.34	0.56
1:H:413:ILE:HD11	1:H:422:LEU:HD23	1.86	0.56
1:L:193:ASN:OD1	1:L:194:LYS:N	2.38	0.56

Continued on next page...

For Manuscript Review

Continued from previous page...

Atom-1	Atom-2	Interatomic distance (Å)	Clash overlap (Å)
1:L:538:ASP:OD2	1:L:593:LYS:NZ	2.36	0.56
1:F:300:THR:HG22	1:F:305:VAL:HA	1.88	0.56
1:A:178:ASP:OD1	1:A:219:THR:HG21	2.04	0.56
1:F:413:ILE:HD11	1:F:422:LEU:HD23	1.86	0.56
1:H:214:LYS:HG3	1:H:219:THR:HG22	1.88	0.56
1:B:211:ASN:ND2	3:B:902:TPP:O1B	2.33	0.56
1:D:306:VAL:HG11	1:D:316:PRO:HB3	1.88	0.56
1:G:167:ASN:OD1	1:G:485:ARG:NH1	2.33	0.56
1:H:300:THR:HG22	1:H:305:VAL:HA	1.88	0.56
1:B:214:LYS:HG3	1:B:219:THR:HG22	1.88	0.56
1:G:193:ASN:OD1	1:G:194:LYS:N	2.39	0.56
1:L:211:ASN:ND2	3:L:902:TPP:O1B	2.32	0.56
1:L:69:PHE:O	1:L:73:HIS:ND1	2.30	0.56
1:A:82:ASP:OD1	1:A:362:ARG:NE	2.38	0.56
1:E:293:LYS:HE2	1:E:313:HIS:HA	1.88	0.56
1:C:560:VAL:HG13	1:C:561:THR:HG23	1.87	0.55
1:D:85:LEU:HD23	1:D:171:ILE:HB	1.88	0.55
1:G:69:PHE:O	1:G:73:HIS:ND1	2.30	0.55
1:G:560:VAL:HG13	1:G:561:THR:HG23	1.88	0.55
1:L:560:VAL:HG13	1:L:561:THR:HG23	1.88	0.55
1:D:214:LYS:HG3	1:D:219:THR:HG22	1.88	0.55
1:D:560:VAL:HG13	1:D:561:THR:HG23	1.87	0.55
1:G:385:ARG:HG2	1:G:602:ALA:HB1	1.87	0.55
1:K:560:VAL:HG13	1:K:561:THR:HG23	1.88	0.55
1:L:93:HIS:NE2	3:L:902:TPP:O3B	2.38	0.55
1:L:385:ARG:HG2	1:L:602:ALA:HB1	1.88	0.55
1:E:214:LYS:HG3	1:E:219:THR:HG22	1.89	0.55
1:I:193:ASN:ND2	1:I:236:TYR:O	2.39	0.55
1:H:306:VAL:HG11	1:H:316:PRO:HB3	1.87	0.55
1:J:193:ASN:OD1	1:J:194:LYS:HD3	2.06	0.55
1:J:211:ASN:ND2	3:J:902:TPP:O1B	2.33	0.55
1:K:214:LYS:HG3	1:K:219:THR:HG22	1.88	0.55
1:C:300:THR:HG22	1:C:305:VAL:HA	1.89	0.55
1:K:300:THR:HG22	1:K:305:VAL:HA	1.89	0.55
1:A:93:HIS:NE2	3:A:902:TPP:O3B	2.37	0.55
1:G:300:THR:HG22	1:G:305:VAL:HA	1.88	0.55
1:H:193:ASN:OD1	1:H:194:LYS:N	2.40	0.55
1:B:78:ILE:HD11	1:B:85:LEU:HB2	1.89	0.55
1:B:300:THR:HG22	1:B:305:VAL:HA	1.88	0.55
1:K:293:LYS:HE2	1:K:313:HIS:HA	1.88	0.55
1:F:93:HIS:NE2	3:F:902:TPP:O3B	2.38	0.55

Continued on next page...

For Manuscript Review

Continued from previous page...

Atom-1	Atom-2	Interatomic distance (Å)	Clash overlap (Å)
1:F:560:VAL:HG13	1:F:561:THR:HG23	1.88	0.55
1:F:442:LYS:HD2	1:F:466:GLU:HG3	1.88	0.54
1:K:561:THR:HG22	1:K:586:ILE:HB	1.89	0.54
1:J:538:ASP:OD2	1:J:593:LYS:NZ	2.35	0.54
1:C:214:LYS:HG3	1:C:219:THR:HG22	1.89	0.54
1:D:300:THR:HG22	1:D:305:VAL:HA	1.90	0.54
1:F:105:GLU:HB3	1:F:363:ARG:HD2	1.90	0.54
1:I:96:PRO:HG3	1:I:129:PHE:CZ	2.43	0.54
1:K:193:ASN:OD1	1:K:194:LYS:N	2.40	0.54
1:E:300:THR:HG22	1:E:305:VAL:HA	1.89	0.54
1:F:82:ASP:OD1	1:F:362:ARG:NE	2.39	0.54
1:I:211:ASN:ND2	3:I:902:TPP:O2B	2.33	0.54
1:B:306:VAL:HG11	1:B:316:PRO:HB3	1.90	0.54
1:A:105:GLU:HB3	1:A:363:ARG:HD2	1.90	0.54
1:C:306:VAL:HG11	1:C:316:PRO:HB3	1.90	0.54
1:D:601:ASP:OD1	1:D:601:ASP:N	2.35	0.54
1:E:538:ASP:OD2	1:E:593:LYS:NZ	2.34	0.54
1:H:293:LYS:HE2	1:H:313:HIS:HA	1.89	0.54
1:L:442:LYS:HD2	1:L:466:GLU:HG3	1.89	0.54
1:A:560:VAL:HG13	1:A:561:THR:HG23	1.88	0.54
1:G:492:TYR:OH	1:G:593:LYS:NZ	2.41	0.54
1:H:96:PRO:HG3	1:H:129:PHE:CZ	2.43	0.54
1:I:300:THR:HG22	1:I:305:VAL:HA	1.89	0.54
1:B:560:VAL:HG13	1:B:561:THR:HG23	1.88	0.54
1:I:69:PHE:O	1:I:73:HIS:ND1	2.31	0.54
1:A:96:PRO:HG3	1:A:129:PHE:CZ	2.43	0.54
1:J:96:PRO:HG3	1:J:129:PHE:CZ	2.43	0.54
1:K:96:PRO:HG3	1:K:129:PHE:CZ	2.43	0.54
1:D:293:LYS:HE2	1:D:313:HIS:HA	1.90	0.54
1:J:178:ASP:OD1	1:J:179:GLY:N	2.41	0.54
1:A:78:ILE:HD11	1:A:85:LEU:HB2	1.89	0.53
1:B:442:LYS:HD2	1:B:466:GLU:HG3	1.90	0.53
1:B:702:PRO:HB2	2:B:901:ANP:H4’	1.90	0.53
1:C:93:HIS:NE2	3:C:902:TPP:O3B	2.40	0.53
1:C:96:PRO:HG3	1:C:129:PHE:CZ	2.42	0.53
1:C:293:LYS:HE2	1:C:313:HIS:HA	1.90	0.53
1:E:306:VAL:HG11	1:E:316:PRO:HB3	1.90	0.53
1:F:96:PRO:HG3	1:F:129:PHE:CZ	2.43	0.53
1:K:306:VAL:HG11	1:K:316:PRO:HB3	1.89	0.53
1:I:167:ASN:OD1	1:I:485:ARG:NH1	2.32	0.53
1:I:699:HIS:O	1:I:699:HIS:ND1	2.41	0.53

Continued on next page...

For Manuscript Review

Continued from previous page...

Atom-1	Atom-2	Interatomic distance (Å)	Clash overlap (Å)
1:L:105:GLU:HB3	1:L:363:ARG:HD2	1.90	0.53
1:L:178:ASP:OD1	1:L:179:GLY:N	2.42	0.53
1:E:560:VAL:HG13	1:E:561:THR:HG23	1.88	0.53
1:H:492:TYR:OH	1:H:593:LYS:NZ	2.41	0.53
1:I:178:ASP:OD1	1:I:179:GLY:N	2.42	0.53
1:A:300:THR:HG22	1:A:305:VAL:HA	1.91	0.53
1:B:293:LYS:HE2	1:B:313:HIS:HA	1.91	0.53
1:B:82:ASP:OD1	1:B:362:ARG:NE	2.40	0.53
1:G:105:GLU:HB3	1:G:363:ARG:HD2	1.90	0.53
1:G:178:ASP:OD1	1:G:179:GLY:N	2.42	0.53
1:G:214:LYS:HG3	1:G:219:THR:HG22	1.91	0.53
1:H:442:LYS:HD2	1:H:466:GLU:HG3	1.90	0.53
1:K:492:TYR:OH	1:K:593:LYS:NZ	2.42	0.53
1:L:601:ASP:OD1	1:L:601:ASP:N	2.35	0.53
1:I:217:ASN:ND2	1:J:426:ASP:OD1	2.28	0.53
1:J:193:ASN:OD1	1:J:194:LYS:CD	2.57	0.53
1:C:211:ASN:ND2	3:C:902:TPP:O2B	2.34	0.53
1:C:217:ASN:ND2	1:D:426:ASP:OD1	2.29	0.53
1:J:300:THR:HG22	1:J:305:VAL:HA	1.90	0.53
1:D:96:PRO:HG3	1:D:129:PHE:CZ	2.43	0.53
1:D:442:LYS:HD2	1:D:466:GLU:HG3	1.90	0.53
1:K:423:PHE:HB3	1:K:473:LEU:HD22	1.91	0.53
1:D:157:LEU:HD13	1:D:176:ILE:HD11	1.91	0.52
1:J:699:HIS:O	1:J:699:HIS:ND1	2.42	0.52
1:G:96:PRO:HG3	1:G:129:PHE:CZ	2.43	0.52
1:L:306:VAL:HG11	1:L:316:PRO:HB3	1.92	0.52
1:C:82:ASP:OD1	1:C:362:ARG:NE	2.39	0.52
1:C:426:ASP:OD1	1:D:217:ASN:ND2	2.34	0.52
1:D:93:HIS:NE2	3:D:902:TPP:O3B	2.41	0.52
1:D:167:ASN:OD1	1:D:485:ARG:NH1	2.33	0.52
1:B:96:PRO:HG3	1:B:129:PHE:CZ	2.43	0.52
1:B:157:LEU:HD13	1:B:176:ILE:HD11	1.92	0.52
1:E:96:PRO:HG3	1:E:129:PHE:CZ	2.43	0.52
1:E:561:THR:HG22	1:E:586:ILE:HB	1.90	0.52
1:G:82:ASP:OD1	1:G:362:ARG:NE	2.40	0.52
1:H:26:ARG:NH1	1:H:337:TYR:O	2.43	0.52
1:L:96:PRO:HG3	1:L:129:PHE:CZ	2.44	0.52
1:B:423:PHE:HB3	1:B:473:LEU:HD22	1.92	0.52
1:B:699:HIS:ND1	1:B:699:HIS:O	2.43	0.52
1:C:105:GLU:HB3	1:C:363:ARG:HD2	1.92	0.52
1:A:193:ASN:OD1	1:A:193:ASN:N	2.43	0.52

Continued on next page...

For Manuscript Review

Continued from previous page...

Atom-1	Atom-2	Interatomic distance (Å)	Clash overlap (Å)
1:B:561:THR:HG22	1:B:586:ILE:HB	1.91	0.52
1:C:699:HIS:O	1:C:699:HIS:ND1	2.41	0.52
1:J:214:LYS:HG3	1:J:219:THR:HG22	1.92	0.52
1:L:699:HIS:O	1:L:699:HIS:ND1	2.43	0.52
1:D:82:ASP:OD1	1:D:362:ARG:NE	2.39	0.51
1:E:93:HIS:NE2	3:E:902:TPP:O3B	2.39	0.51
1:E:157:LEU:HD13	1:E:176:ILE:HD11	1.92	0.51
1:F:489:PHE:HD2	1:F:528:ILE:HG23	1.76	0.51
1:G:666:LEU:HG	1:G:708:LEU:CD1	2.40	0.51
1:G:699:HIS:ND1	1:G:699:HIS:O	2.43	0.51
1:J:442:LYS:HD2	1:J:466:GLU:HG3	1.91	0.51
3:J:902:TPP:HN42	3:J:902:TPP:C2	2.23	0.51
1:A:214:LYS:HG3	1:A:219:THR:HG22	1.91	0.51
1:A:306:VAL:HG11	1:A:316:PRO:HB3	1.92	0.51
1:E:699:HIS:O	1:E:699:HIS:ND1	2.44	0.51
1:F:197:ASN:HD22	1:F:200:GLN:HG2	1.75	0.51
1:H:423:PHE:HB3	1:H:473:LEU:HD22	1.92	0.51
1:H:493:GLU:HG3	1:H:530:LEU:HD13	1.92	0.51
1:I:214:LYS:HG3	1:I:219:THR:HG22	1.92	0.51
1:K:726:VAL:HG12	1:K:735:LEU:HD11	1.92	0.51
1:L:300:THR:HG22	1:L:305:VAL:HA	1.90	0.51
1:E:78:ILE:HD11	1:E:85:LEU:HB2	1.92	0.51
1:H:157:LEU:HD13	1:H:176:ILE:HD11	1.91	0.51
1:L:293:LYS:HE2	1:L:313:HIS:HA	1.91	0.51
1:A:489:PHE:HD2	1:A:528:ILE:HG23	1.76	0.51
1:C:157:LEU:HD13	1:C:176:ILE:HD11	1.92	0.51
1:E:728:ASN:OD1	1:E:729:ILE:N	2.42	0.51
1:F:26:ARG:NH1	1:F:337:TYR:O	2.44	0.51
3:I:902:TPP:C2	3:I:902:TPP:HN42	2.24	0.51
1:A:699:HIS:O	1:A:699:HIS:ND1	2.43	0.51
1:A:728:ASN:OD1	1:A:729:ILE:N	2.42	0.51
1:C:167:ASN:OD1	1:C:485:ARG:NH1	2.33	0.51
1:E:105:GLU:HB3	1:E:363:ARG:HD2	1.92	0.51
1:E:193:ASN:OD1	1:E:194:LYS:HD3	2.11	0.51
1:K:105:GLU:HB3	1:K:363:ARG:HD2	1.93	0.51
1:B:726:VAL:HG12	1:B:735:LEU:HD11	1.92	0.51
1:D:699:HIS:O	1:D:699:HIS:ND1	2.42	0.51
3:G:902:TPP:HN42	3:G:902:TPP:C2	2.24	0.51
1:H:726:VAL:HG12	1:H:735:LEU:HD11	1.93	0.51
1:I:82:ASP:OD1	1:I:362:ARG:NE	2.42	0.51
1:D:211:ASN:ND2	3:D:902:TPP:O1B	2.36	0.51

Continued on next page...

For Manuscript Review

Continued from previous page...

Atom-1	Atom-2	Interatomic distance (Å)	Clash overlap (Å)
1:E:82:ASP:OD1	1:E:362:ARG:NE	2.41	0.51
1:K:155:TYR:CZ	1:K:502:MET:HG2	2.46	0.51
1:K:167:ASN:OD1	1:K:485:ARG:NH1	2.34	0.51
3:L:902:TPP:HN42	3:L:902:TPP:C2	2.24	0.51
1:D:105:GLU:HB3	1:D:363:ARG:HD2	1.93	0.51
1:E:423:PHE:HB3	1:E:473:LEU:HD22	1.91	0.51
1:E:601:ASP:OD1	1:E:601:ASP:N	2.39	0.51
1:H:166:ASP:OD2	1:H:480:TYR:OH	2.24	0.51
1:J:157:LEU:HD13	1:J:176:ILE:HD11	1.92	0.51
1:J:467:ILE:HG13	1:J:469:SER:H	1.75	0.51
1:A:155:TYR:CZ	1:A:502:MET:HG2	2.46	0.51
1:K:157:LEU:HD13	1:K:176:ILE:HD11	1.92	0.51
3:K:902:TPP:C2	3:K:902:TPP:HN42	2.24	0.51
1:L:157:LEU:HD13	1:L:176:ILE:HD11	1.93	0.51
1:L:214:LYS:HG3	1:L:219:THR:HG22	1.93	0.51
1:A:157:LEU:HD13	1:A:176:ILE:HD11	1.92	0.51
1:B:105:GLU:HB3	1:B:363:ARG:HD2	1.93	0.51
1:B:193:ASN:OD1	1:B:193:ASN:N	2.44	0.51
1:F:178:ASP:OD1	1:F:179:GLY:N	2.42	0.51
1:F:493:GLU:HG3	1:F:530:LEU:HD13	1.93	0.51
1:F:699:HIS:O	1:F:699:HIS:ND1	2.44	0.51
1:I:105:GLU:HB3	1:I:363:ARG:HD2	1.92	0.51
1:I:157:LEU:HD13	1:I:176:ILE:HD11	1.92	0.51
1:L:543:SER:HG	1:L:544:HIS:HD1	1.55	0.51
1:H:105:GLU:HB3	1:H:363:ARG:HD2	1.93	0.50
1:H:155:TYR:CZ	1:H:502:MET:HG2	2.46	0.50
1:B:493:GLU:HG3	1:B:530:LEU:HD13	1.94	0.50
1:C:193:ASN:OD1	1:C:193:ASN:N	2.43	0.50
1:E:726:VAL:HG12	1:E:735:LEU:HD11	1.93	0.50
1:H:82:ASP:OD1	1:H:362:ARG:NE	2.41	0.50
1:I:26:ARG:HH22	1:I:342:LEU:HD21	1.77	0.50
1:K:82:ASP:OD1	1:K:362:ARG:NE	2.41	0.50
1:A:197:ASN:HD22	1:A:200:GLN:HG2	1.76	0.50
1:F:155:TYR:CZ	1:F:502:MET:HG2	2.47	0.50
1:F:467:ILE:HG13	1:F:469:SER:H	1.76	0.50
1:G:157:LEU:HD13	1:G:176:ILE:HD11	1.93	0.50
1:J:377:ARG:NH2	1:J:419:ASN:OD1	2.43	0.50
1:K:166:ASP:OD2	1:K:480:TYR:OH	2.24	0.50
1:A:493:GLU:HG3	1:A:530:LEU:HD13	1.94	0.50
3:A:902:TPP:HN42	3:A:902:TPP:C2	2.24	0.50
3:C:902:TPP:HN42	3:C:902:TPP:C2	2.24	0.50

Continued on next page...

For Manuscript Review

Continued from previous page...

Atom-1	Atom-2	Interatomic distance (Å)	Clash overlap (Å)
1:E:155:TYR:CZ	1:E:502:MET:HG2	2.45	0.50
1:F:157:LEU:HD13	1:F:176:ILE:HD11	1.93	0.50
3:F:902:TPP:C2	3:F:902:TPP:HN42	2.24	0.50
1:H:167:ASN:OD1	1:H:485:ARG:NH1	2.34	0.50
1:K:699:HIS:O	1:K:699:HIS:ND1	2.42	0.50
1:A:26:ARG:NH1	1:A:337:TYR:O	2.45	0.50
3:E:902:TPP:HN42	3:E:902:TPP:C2	2.25	0.50
1:I:728:ASN:OD1	1:I:729:ILE:N	2.42	0.50
1:J:82:ASP:OD1	1:J:362:ARG:NE	2.39	0.50
1:B:178:ASP:OD1	1:B:179:GLY:N	2.45	0.50
1:H:219:THR:O	1:H:222:SER:OG	2.30	0.50
1:J:492:TYR:OH	1:J:593:LYS:NZ	2.45	0.50
1:K:219:THR:O	1:K:222:SER:OG	2.30	0.50
1:F:193:ASN:OD1	1:F:194:LYS:HD3	2.11	0.50
1:F:214:LYS:HG3	1:F:219:THR:HG22	1.92	0.50
1:G:155:TYR:CZ	1:G:502:MET:HG2	2.46	0.50
1:J:105:GLU:HB3	1:J:363:ARG:HD2	1.93	0.50
1:K:217:ASN:ND2	1:L:426:ASP:OD1	2.28	0.50
1:K:493:GLU:HG3	1:K:530:LEU:HD13	1.94	0.50
1:B:219:THR:O	1:B:222:SER:OG	2.29	0.50
1:E:492:TYR:OH	1:E:593:LYS:NZ	2.45	0.50
1:G:26:ARG:NH1	1:G:337:TYR:O	2.45	0.50
1:G:78:ILE:HD11	1:G:85:LEU:HB2	1.94	0.50
1:G:726:VAL:HG12	1:G:735:LEU:HD11	1.94	0.50
1:H:178:ASP:OD1	1:H:179:GLY:N	2.45	0.50
1:I:193:ASN:HD21	1:I:194:LYS:HZ1	1.59	0.50
1:A:726:VAL:HG12	1:A:735:LEU:HD11	1.94	0.49
1:B:220:LEU:O	1:B:224:ILE:HG12	2.12	0.49
1:B:467:ILE:HG13	1:B:469:SER:H	1.76	0.49
1:D:467:ILE:HG13	1:D:469:SER:H	1.77	0.49
1:G:442:LYS:HD2	1:G:466:GLU:HG3	1.94	0.49
1:L:82:ASP:OD1	1:L:362:ARG:NE	2.42	0.49
1:L:193:ASN:OD1	1:L:194:LYS:HD3	2.11	0.49
1:L:489:PHE:HD2	1:L:528:ILE:HG23	1.75	0.49
3:B:902:TPP:HN42	3:B:902:TPP:C2	2.25	0.49
1:E:178:ASP:OD1	1:E:179:GLY:N	2.45	0.49
1:F:726:VAL:HG12	1:F:735:LEU:HD11	1.94	0.49
3:H:902:TPP:HN42	3:H:902:TPP:C2	2.25	0.49
1:E:219:THR:O	1:E:222:SER:OG	2.30	0.49
1:E:493:GLU:HG3	1:E:530:LEU:HD13	1.95	0.49
1:I:349:PRO:HB3	1:I:353:LEU:HD12	1.92	0.49

Continued on next page...

For Manuscript Review

Continued from previous page...

Atom-1	Atom-2	Interatomic distance (Å)	Clash overlap (Å)
1:K:178:ASP:OD1	1:K:179:GLY:N	2.46	0.49
1:L:197:ASN:HD22	1:L:200:GLN:HG2	1.77	0.49
1:L:492:TYR:OH	1:L:593:LYS:NZ	2.45	0.49
1:A:167:ASN:OD1	1:A:485:ARG:NH1	2.33	0.49
1:E:426:ASP:OD1	1:F:217:ASN:ND2	2.35	0.49
1:H:467:ILE:HG13	1:H:469:SER:H	1.76	0.49
1:I:93:HIS:NE2	3:I:902:TPP:O3B	2.41	0.49
1:J:93:HIS:NE2	3:J:902:TPP:O3B	2.41	0.49
1:K:97:ALA:O	1:K:101:ASN:ND2	2.37	0.49
1:K:570:ASN:ND2	1:K:600:LEU:O	2.34	0.49
1:L:155:TYR:CZ	1:L:502:MET:HG2	2.47	0.49
1:A:219:THR:O	1:A:222:SER:OG	2.31	0.49
1:B:155:TYR:CZ	1:B:502:MET:HG2	2.46	0.49
1:C:155:TYR:CZ	1:C:502:MET:HG2	2.47	0.49
1:D:155:TYR:CZ	1:D:502:MET:HG2	2.47	0.49
1:H:554:THR:HG21	1:H:704:LEU:HD13	1.95	0.49
1:D:178:ASP:OD1	1:D:179:GLY:N	2.45	0.49
1:G:489:PHE:HD2	1:G:528:ILE:HG23	1.76	0.49
1:H:220:LEU:O	1:H:224:ILE:HG12	2.12	0.49
1:I:78:ILE:HD11	1:I:85:LEU:HB2	1.94	0.49
1:L:178:ASP:HB3	1:L:210:LEU:HD12	1.95	0.49
1:L:493:GLU:HG3	1:L:530:LEU:HD13	1.94	0.49
1:D:561:THR:HG22	1:D:586:ILE:HB	1.94	0.49
3:D:902:TPP:C2	3:D:902:TPP:HN42	2.25	0.49
1:G:467:ILE:HG13	1:G:469:SER:H	1.76	0.49
1:C:26:ARG:NH1	1:C:337:TYR:O	2.46	0.49
1:C:561:THR:HG22	1:C:586:ILE:HB	1.94	0.49
1:F:306:VAL:HG11	1:F:316:PRO:HB3	1.95	0.49
1:H:489:PHE:HD2	1:H:528:ILE:HG23	1.78	0.49
1:H:699:HIS:O	1:H:699:HIS:ND1	2.44	0.49
1:I:197:ASN:HD22	1:I:200:GLN:HG2	1.78	0.49
1:L:349:PRO:HB3	1:L:353:LEU:HD12	1.94	0.49
1:L:703[B]:TRP:HD1	2:L:901:ANP:HO3'	1.60	0.49
1:L:726:VAL:HG12	1:L:735:LEU:HD11	1.95	0.49
1:C:467:ILE:HG13	1:C:469:SER:H	1.78	0.49
1:E:193:ASN:OD1	1:E:194:LYS:CD	2.60	0.49
1:G:217:ASN:ND2	1:H:426:ASP:OD1	2.28	0.49
1:G:728:ASN:OD1	1:G:729:ILE:N	2.42	0.49
1:I:155:TYR:CZ	1:I:502:MET:HG2	2.47	0.49
1:J:561:THR:HG22	1:J:586:ILE:HB	1.94	0.49
1:C:442:LYS:HD2	1:C:466:GLU:HG3	1.95	0.49

Continued on next page...

For Manuscript Review

Continued from previous page...

Atom-1	Atom-2	Interatomic distance (Å)	Clash overlap (Å)
1:D:220:LEU:O	1:D:224:ILE:HG12	2.12	0.49
1:D:349:PRO:HB3	1:D:353:LEU:HD12	1.94	0.49
1:K:489:PHE:HD2	1:K:528:ILE:HG23	1.78	0.49
1:L:193:ASN:OD1	1:L:194:LYS:CD	2.61	0.49
1:F:167:ASN:OD1	1:F:485:ARG:NH1	2.33	0.48
1:G:219:THR:O	1:G:222:SER:OG	2.30	0.48
1:J:155:TYR:CZ	1:J:502:MET:HG2	2.47	0.48
1:J:197:ASN:HD22	1:J:200:GLN:HG2	1.78	0.48
1:A:426:ASP:OD1	1:B:217:ASN:ND2	2.35	0.48
1:B:728:ASN:OD1	1:B:729:ILE:N	2.45	0.48
1:F:178:ASP:HB3	1:F:210:LEU:HD12	1.95	0.48
1:G:197:ASN:HD22	1:G:200:GLN:HG2	1.77	0.48
1:G:349:PRO:HB3	1:G:353:LEU:HD12	1.95	0.48
1:E:635:GLY:O	1:E:639:THR:OG1	2.23	0.48
1:H:349:PRO:HB3	1:H:353:LEU:HD12	1.95	0.48
1:I:219:THR:O	1:I:222:SER:OG	2.31	0.48
1:I:442:LYS:HD2	1:I:466:GLU:HG3	1.95	0.48
1:A:561:THR:HG22	1:A:586:ILE:HB	1.96	0.48
1:C:689:THR:O	1:C:714:ASN:ND2	2.47	0.48
1:G:493:GLU:HG3	1:G:530:LEU:HD13	1.95	0.48
1:B:93:HIS:NE2	3:B:902:TPP:O3B	2.42	0.48
1:C:197:ASN:HD22	1:C:200:GLN:HG2	1.77	0.48
1:D:219:THR:O	1:D:222:SER:OG	2.32	0.48
1:I:193:ASN:HD21	1:I:194:LYS:NZ	2.11	0.48
1:K:93:HIS:NE2	3:K:902:TPP:O3B	2.40	0.48
1:K:728:ASN:OD1	1:K:729:ILE:N	2.42	0.48
1:L:178:ASP:OD2	1:L:219:THR:HG21	2.12	0.48
1:A:293:LYS:HE2	1:A:313:HIS:HA	1.95	0.48
1:A:349:PRO:HB3	1:A:353:LEU:HD12	1.94	0.48
1:A:467:ILE:HG13	1:A:469:SER:H	1.79	0.48
1:C:703[C]:TRP:CE2	1:D:707:LYS:HE2	2.48	0.48
1:D:197:ASN:HD22	1:D:200:GLN:HG2	1.78	0.48
1:F:738:ARG:NH2	1:F:780:TYR:OH	2.47	0.48
1:J:422:LEU:HB2	1:J:464:VAL:HG22	1.94	0.48
1:B:738:ARG:NH2	1:B:780:TYR:OH	2.47	0.48
1:D:689:THR:O	1:D:714:ASN:ND2	2.47	0.48
1:E:10:ILE:O	1:E:76:ARG:NH2	2.46	0.48
1:E:349:PRO:HB3	1:E:353:LEU:HD12	1.96	0.48
1:B:349:PRO:HB3	1:B:353:LEU:HD12	1.95	0.48
1:F:561:THR:HG22	1:F:586:ILE:HB	1.96	0.48
1:G:178:ASP:HB3	1:G:210:LEU:HD12	1.96	0.48

Continued on next page...

For Manuscript Review

Continued from previous page...

Atom-1	Atom-2	Interatomic distance (Å)	Clash overlap (Å)
1:K:426:ASP:OD1	1:L:217:ASN:ND2	2.35	0.48
1:B:489:PHE:HD2	1:B:528:ILE:HG23	1.78	0.48
1:F:178:ASP:OD2	1:F:219:THR:HG21	2.12	0.48
1:F:349:PRO:HB3	1:F:353:LEU:HD12	1.94	0.48
1:H:193:ASN:OD1	1:H:194:LYS:HD3	2.13	0.48
1:D:726:VAL:HG12	1:D:735:LEU:HD11	1.96	0.48
1:E:220:LEU:O	1:E:224:ILE:HG12	2.14	0.48
1:F:193:ASN:OD1	1:F:194:LYS:CD	2.61	0.48
1:I:193:ASN:CG	1:I:194:LYS:HZ2	2.16	0.48
1:A:778:TYR:CZ	1:A:782:GLU:HG3	2.49	0.47
1:B:635:GLY:O	1:B:639:THR:OG1	2.23	0.47
1:F:293:LYS:HE2	1:F:313:HIS:HA	1.95	0.47
1:I:467:ILE:HG13	1:I:469:SER:H	1.79	0.47
1:J:728:ASN:OD1	1:J:729:ILE:N	2.45	0.47
1:K:220:LEU:O	1:K:224:ILE:HG12	2.14	0.47
1:L:167:ASN:OD1	1:L:485:ARG:NH1	2.33	0.47
1:C:349:PRO:HB3	1:C:353:LEU:HD12	1.96	0.47
1:F:219:THR:O	1:F:222:SER:OG	2.32	0.47
1:G:759:ASP:N	1:G:759:ASP:OD1	2.47	0.47
1:B:10:ILE:O	1:B:76:ARG:NH2	2.47	0.47
1:G:778:TYR:CZ	1:G:782:GLU:HG3	2.49	0.47
1:L:778:TYR:CZ	1:L:782:GLU:HG3	2.49	0.47
1:D:193:ASN:OD1	1:D:193:ASN:N	2.44	0.47
1:D:493:GLU:HG3	1:D:530:LEU:HD13	1.97	0.47
1:D:563:ILE:HD13	1:D:588:VAL:HB	1.97	0.47
1:F:778:TYR:CZ	1:F:782:GLU:HG3	2.49	0.47
1:H:93:HIS:NE2	3:H:902:TPP:O3B	2.41	0.47
1:J:306:VAL:HG11	1:J:316:PRO:HB3	1.96	0.47
1:K:178:ASP:OD2	1:K:219:THR:OG1	2.28	0.47
1:L:759:ASP:N	1:L:759:ASP:OD1	2.48	0.47
1:A:601:ASP:OD1	1:A:601:ASP:N	2.39	0.47
1:E:178:ASP:HB3	1:E:210:LEU:HD12	1.96	0.47
1:J:167:ASN:OD1	1:J:485:ARG:NH1	2.34	0.47
1:J:349:PRO:HB3	1:J:353:LEU:HD12	1.94	0.47
1:K:707:LYS:HE2	1:L:703[B]:TRP:CE2	2.49	0.47
1:A:442:LYS:HD2	1:A:466:GLU:HG3	1.95	0.47
1:F:601:ASP:OD1	1:F:601:ASP:N	2.35	0.47
1:F:728:ASN:OD1	1:F:729:ILE:N	2.45	0.47
1:H:97:ALA:O	1:H:101:ASN:ND2	2.38	0.47
1:I:293:LYS:HE2	1:I:313:HIS:HA	1.97	0.47
1:J:293:LYS:HE2	1:J:313:HIS:HA	1.97	0.47

Continued on next page...

For Manuscript Review

Continued from previous page...

Atom-1	Atom-2	Interatomic distance (Å)	Clash overlap (Å)
1:C:601:ASP:OD1	1:C:601:ASP:N	2.39	0.47
1:E:489:PHE:HD2	1:E:528:ILE:HG23	1.78	0.47
1:G:178:ASP:OD2	1:G:219:THR:HG21	2.13	0.47
1:G:710:TYR:CZ	2:H:901:ANP:H2'	2.50	0.47
1:H:178:ASP:OD2	1:H:219:THR:HG21	2.14	0.47
1:H:666:LEU:HG	1:H:708:LEU:CD1	2.44	0.47
1:H:759:ASP:OD1	1:H:759:ASP:N	2.47	0.47
1:I:778:TYR:CZ	1:I:782:GLU:HG3	2.50	0.47
1:J:220:LEU:O	1:J:224:ILE:HG12	2.14	0.47
1:J:563:ILE:HD13	1:J:588:VAL:HB	1.97	0.47
1:J:778:TYR:CZ	1:J:782:GLU:HG3	2.50	0.47
1:K:86:ILE:HG12	1:K:170:LEU:HD11	1.96	0.47
1:K:197:ASN:HD22	1:K:200:GLN:HG2	1.80	0.47
1:K:759:ASP:OD1	1:K:759:ASP:N	2.47	0.47
1:L:219:THR:O	1:L:222:SER:OG	2.32	0.47
1:I:220:LEU:O	1:I:224:ILE:HG12	2.14	0.47
1:I:561:THR:HG22	1:I:586:ILE:HB	1.95	0.47
1:J:219:THR:O	1:J:222:SER:OG	2.32	0.47
1:J:726:VAL:HG12	1:J:735:LEU:HD11	1.97	0.47
1:K:422:LEU:HB2	1:K:464:VAL:HG22	1.97	0.47
1:A:220:LEU:O	1:A:224:ILE:HG12	2.15	0.47
1:D:570:ASN:ND2	1:D:600:LEU:O	2.32	0.47
1:D:778:TYR:CZ	1:D:782:GLU:HG3	2.50	0.47
1:H:778:TYR:CZ	1:H:782:GLU:HG3	2.50	0.47
1:I:707:LYS:HE2	1:J:703[B]:TRP:CE2	2.49	0.47
1:I:759:ASP:N	1:I:759:ASP:OD1	2.48	0.47
1:K:349:PRO:HB3	1:K:353:LEU:HD12	1.96	0.47
1:B:85:LEU:HD23	1:B:171:ILE:HB	1.97	0.47
1:E:167:ASN:OD1	1:E:485:ARG:NH1	2.33	0.47
1:E:442:LYS:HD2	1:E:466:GLU:HG3	1.97	0.47
1:G:193:ASN:OD1	1:G:194:LYS:CD	2.63	0.47
1:G:561:THR:HG22	1:G:586:ILE:HB	1.96	0.47
1:H:193:ASN:OD1	1:H:194:LYS:CD	2.63	0.47
1:I:726:VAL:HG12	1:I:735:LEU:HD11	1.97	0.47
1:J:689:THR:O	1:J:714:ASN:ND2	2.47	0.47
1:L:561:THR:HG22	1:L:586:ILE:HB	1.96	0.47
1:C:220:LEU:O	1:C:224:ILE:HG12	2.14	0.46
1:C:778:TYR:CZ	1:C:782:GLU:HG3	2.50	0.46
1:F:220:LEU:O	1:F:224:ILE:HG12	2.15	0.46
1:G:220:LEU:O	1:G:224:ILE:HG12	2.15	0.46
1:H:78:ILE:HD11	1:H:85:LEU:HB2	1.96	0.46

Continued on next page...

For Manuscript Review

Continued from previous page...

Atom-1	Atom-2	Interatomic distance (Å)	Clash overlap (Å)
1:H:422:LEU:HB2	1:H:464:VAL:HG22	1.97	0.46
1:J:759:ASP:OD1	1:J:759:ASP:N	2.48	0.46
1:L:220:LEU:O	1:L:224:ILE:HG12	2.15	0.46
1:L:467:ILE:HG13	1:L:469:SER:H	1.79	0.46
1:A:472:THR:HG21	1:A:476:TRP:CZ2	2.50	0.46
1:C:86:ILE:HG12	1:C:170:LEU:HD11	1.98	0.46
1:C:219:THR:O	1:C:222:SER:OG	2.33	0.46
1:G:193:ASN:OD1	1:G:194:LYS:HD3	2.14	0.46
1:H:86:ILE:HG12	1:H:170:LEU:HD11	1.97	0.46
1:K:778:TYR:CZ	1:K:782:GLU:HG3	2.51	0.46
1:A:753:ARG:NH2	2:A:901:ANP:O2B	2.48	0.46
1:C:726:VAL:HG12	1:C:735:LEU:HD11	1.97	0.46
1:H:738:ARG:NH2	1:H:780:TYR:OH	2.47	0.46
1:I:306:VAL:HG11	1:I:316:PRO:HB3	1.97	0.46
1:L:738:ARG:NH2	1:L:780:TYR:OH	2.48	0.46
1:A:703[C]:TRP:CE2	1:B:707:LYS:HE2	2.51	0.46
1:B:167:ASN:OD1	1:B:485:ARG:NH1	2.34	0.46
1:F:759:ASP:OD1	1:F:759:ASP:N	2.48	0.46
1:I:178:ASP:HB3	1:I:210:LEU:HD12	1.97	0.46
1:J:570:ASN:ND2	1:J:600:LEU:O	2.32	0.46
1:K:467:ILE:HG13	1:K:469:SER:H	1.79	0.46
1:A:759:ASP:OD1	1:A:759:ASP:N	2.47	0.46
1:B:778:TYR:CZ	1:B:782:GLU:HG3	2.51	0.46
1:G:703[C]:TRP:CE2	1:H:707:LYS:HE2	2.50	0.46
1:J:86:ILE:HG12	1:J:170:LEU:HD11	1.96	0.46
1:K:710:TYR:CZ	2:L:901:ANP:H2’	2.51	0.46
1:H:10:ILE:O	1:H:76:ARG:NH2	2.47	0.46
1:H:601:ASP:N	1:H:601:ASP:OD1	2.36	0.46
1:K:193:ASN:OD1	1:K:194:LYS:HD3	2.15	0.46
1:B:141:PRO:O	1:B:520:ARG:HD2	2.16	0.46
2:C:901:ANP:O5’	2:C:901:ANP:H8	2.15	0.46
1:G:306:VAL:HG11	1:G:316:PRO:HB3	1.98	0.46
1:I:77:LEU:HD11	1:I:259:LEU:HB3	1.97	0.46
2:J:901:ANP:O5’	2:J:901:ANP:H8	2.15	0.46
1:K:442:LYS:HD2	1:K:466:GLU:HG3	1.98	0.46
1:B:570:ASN:ND2	1:B:600:LEU:O	2.32	0.46
1:C:728:ASN:OD1	1:C:729:ILE:N	2.42	0.46
1:G:141:PRO:O	1:G:520:ARG:HD2	2.16	0.46
1:I:601:ASP:OD1	1:I:601:ASP:N	2.39	0.46
1:J:703[B]:TRP:HD1	2:J:901:ANP:HO3’	1.63	0.46
1:A:178:ASP:OD1	3:A:902:TPP:O2A	2.34	0.46

Continued on next page...

For Manuscript Review

Continued from previous page...

Atom-1	Atom-2	Interatomic distance (Å)	Clash overlap (Å)
1:A:179:GLY:O	1:B:468:LEU:HD23	2.16	0.46
1:A:217:ASN:ND2	1:B:426:ASP:OD1	2.28	0.46
1:B:178:ASP:HB3	1:B:210:LEU:HD12	1.98	0.46
1:C:563:ILE:HD13	1:C:588:VAL:HB	1.98	0.46
1:E:467:ILE:HG13	1:E:469:SER:H	1.80	0.46
1:E:703[C]:TRP:CE2	1:F:707:LYS:HE2	2.51	0.46
1:E:778:TYR:CZ	1:E:782:GLU:HG3	2.51	0.46
1:I:703[B]:TRP:CE2	1:J:707:LYS:HE2	2.51	0.46
1:K:35:GLN:O	1:K:54:LYS:NZ	2.41	0.46
1:L:141:PRO:O	1:L:520:ARG:HD2	2.16	0.46
1:L:563:ILE:HD13	1:L:588:VAL:HB	1.98	0.46
1:B:197:ASN:HD22	1:B:200:GLN:HG2	1.81	0.46
1:D:710:TYR:CE2	1:D:711:ARG:HG3	2.51	0.46
1:E:141:PRO:O	1:E:520:ARG:HD2	2.16	0.46
1:J:178:ASP:HB3	1:J:210:LEU:HD12	1.97	0.46
1:K:193:ASN:OD1	1:K:194:LYS:CD	2.64	0.46
1:K:468:LEU:HD12	1:K:468:LEU:HA	1.78	0.46
1:E:422:LEU:HB2	1:E:464:VAL:HG22	1.97	0.45
1:G:10:ILE:O	1:G:76:ARG:NH2	2.48	0.45
1:I:35:GLN:O	1:I:54:LYS:NZ	2.45	0.45
1:B:153:LEU:HD23	3:B:902:TPP:N4'	2.31	0.45
1:G:426:ASP:OD1	1:H:217:ASN:ND2	2.34	0.45
1:H:153:LEU:HD23	3:H:902:TPP:N4'	2.31	0.45
1:L:77:LEU:HD11	1:L:259:LEU:HB3	1.98	0.45
1:E:153:LEU:HD23	3:E:902:TPP:N4'	2.32	0.45
1:E:197:ASN:HD22	1:E:200:GLN:HG2	1.81	0.45
1:E:468:LEU:HD23	1:F:179:GLY:O	2.17	0.45
1:H:197:ASN:HD22	1:H:200:GLN:HG2	1.82	0.45
1:I:153:LEU:HD23	3:I:902:TPP:N4'	2.32	0.45
1:K:10:ILE:O	1:K:76:ARG:NH2	2.48	0.45
1:L:153:LEU:HD23	3:L:902:TPP:N4'	2.31	0.45
1:A:710:TYR:CE2	1:A:711:ARG:HG3	2.52	0.45
1:B:422:LEU:HB2	1:B:464:VAL:HG22	1.97	0.45
1:C:141:PRO:O	1:C:520:ARG:HD2	2.16	0.45
1:C:493:GLU:HG3	1:C:530:LEU:HD13	1.99	0.45
1:D:153:LEU:HD23	3:D:902:TPP:N4'	2.31	0.45
1:E:710:TYR:CZ	2:F:901:ANP:H2'	2.51	0.45
1:F:153:LEU:HD23	3:F:902:TPP:N4'	2.31	0.45
1:I:426:ASP:OD1	1:J:217:ASN:ND2	2.33	0.45
1:J:493:GLU:HG3	1:J:530:LEU:HD13	1.98	0.45
1:L:10:ILE:O	1:L:76:ARG:NH2	2.48	0.45

Continued on next page...

For Manuscript Review

Continued from previous page...

Atom-1	Atom-2	Interatomic distance (Å)	Clash overlap (Å)
1:L:472:THR:HG21	1:L:476:TRP:CZ2	2.50	0.45
1:C:153:LEU:HD23	3:C:902:TPP:N4’	2.32	0.45
1:D:141:PRO:O	1:D:520:ARG:HD2	2.16	0.45
1:D:489:PHE:HD2	1:D:528:ILE:HG23	1.81	0.45
1:F:10:ILE:O	1:F:76:ARG:NH2	2.49	0.45
1:F:141:PRO:O	1:F:520:ARG:HD2	2.16	0.45
1:G:77:LEU:HD11	1:G:259:LEU:HB3	1.99	0.45
1:G:153:LEU:HD23	3:G:902:TPP:N4’	2.31	0.45
1:H:691:ASP:OD1	1:H:691:ASP:N	2.50	0.45
1:J:153:LEU:HD23	3:J:902:TPP:N4’	2.32	0.45
1:K:691:ASP:OD1	1:K:691:ASP:N	2.50	0.45
1:A:141:PRO:O	1:A:520:ARG:HD2	2.16	0.45
1:A:689:THR:O	1:A:714:ASN:ND2	2.50	0.45
1:J:601:ASP:OD1	1:J:601:ASP:N	2.35	0.45
1:A:153:LEU:HD23	3:A:902:TPP:N4’	2.32	0.45
1:C:178:ASP:OD1	1:C:179:GLY:N	2.49	0.45
1:G:508:LYS:HG3	1:H:544:HIS:CG	2.52	0.45
1:I:84:ASN:O	1:I:85:LEU:HD12	2.17	0.45
1:J:141:PRO:O	1:J:520:ARG:HD2	2.16	0.45
1:K:153:LEU:HD23	3:K:902:TPP:N4’	2.31	0.45
1:L:78:ILE:HD11	1:L:85:LEU:HB2	1.97	0.45
1:A:508:LYS:HG3	1:B:544:HIS:CG	2.51	0.45
1:C:759:ASP:OD1	1:C:759:ASP:N	2.48	0.45
1:E:759:ASP:N	1:E:759:ASP:OD1	2.47	0.45
1:K:178:ASP:HB3	1:K:210:LEU:HD12	1.97	0.45
1:K:563:ILE:HD13	1:K:588:VAL:HB	1.98	0.45
1:L:703[B]:TRP:HD1	2:L:901:ANP:HO2’	1.63	0.45
1:A:10:ILE:O	1:A:76:ARG:NH2	2.49	0.45
1:D:759:ASP:OD1	1:D:759:ASP:N	2.48	0.45
1:G:293:LYS:HE2	1:G:313:HIS:HA	1.98	0.45
1:H:570:ASN:ND2	1:H:600:LEU:O	2.37	0.45
1:I:141:PRO:O	1:I:520:ARG:HD2	2.16	0.45
1:J:423:PHE:HB3	1:J:473:LEU:HD22	1.98	0.45
1:J:489:PHE:HD2	1:J:528:ILE:HG23	1.81	0.45
1:C:178:ASP:HB3	1:C:210:LEU:HD12	1.99	0.45
1:C:489:PHE:HD2	1:C:528:ILE:HG23	1.82	0.45
1:E:217:ASN:ND2	1:F:426:ASP:OD1	2.27	0.45
2:E:901:ANP:O5’	2:E:901:ANP:H8	2.16	0.45
1:F:293:LYS:H	1:F:308:GLY:HA2	1.81	0.45
1:H:293:LYS:H	1:H:308:GLY:HA2	1.82	0.45
1:A:77:LEU:HD11	1:A:259:LEU:HB3	1.99	0.44

Continued on next page...

For Manuscript Review

Continued from previous page...

Atom-1	Atom-2	Interatomic distance (Å)	Clash overlap (Å)
1:H:40:ASP:OD1	1:H:41:ASN:N	2.49	0.44
1:H:594:GLN:HB2	1:H:596:HIS:CE1	2.52	0.44
1:K:293:LYS:H	1:K:308:GLY:HA2	1.82	0.44
1:L:422:LEU:HB2	1:L:464:VAL:HG22	1.99	0.44
1:B:178:ASP:OD2	1:B:219:THR:HG21	2.14	0.44
1:B:748:ILE:HG23	1:B:765:LYS:HE2	1.98	0.44
1:D:422:LEU:HB2	1:D:464:VAL:HG22	1.99	0.44
1:F:430:SER:O	1:F:593:LYS:HD2	2.17	0.44
1:F:703[B]:TRP:HD1	2:F:901:ANP:O3'	2.01	0.44
1:K:500:ASP:HB2	1:K:552:LEU:HD23	1.97	0.44
1:L:689:THR:O	1:L:714:ASN:ND2	2.49	0.44
1:B:48:LEU:HD21	1:B:53:ILE:HD11	2.00	0.44
1:C:179:GLY:O	1:D:468:LEU:HD23	2.17	0.44
1:E:613:ILE:HB	1:E:663:VAL:HG23	1.99	0.44
1:F:48:LEU:HD21	1:F:53:ILE:HD11	1.98	0.44
2:G:901:ANP:H2'	1:H:710:TYR:CZ	2.53	0.44
1:H:178:ASP:HB3	1:H:210:LEU:HD12	1.98	0.44
1:I:493:GLU:HG3	1:I:530:LEU:HD13	1.99	0.44
1:J:77:LEU:HD11	1:J:259:LEU:HB3	1.99	0.44
1:J:738:ARG:NH2	1:J:780:TYR:OH	2.51	0.44
1:K:748:ILE:HG23	1:K:765:LYS:HE2	1.98	0.44
1:L:728:ASN:OD1	1:L:729:ILE:N	2.46	0.44
1:B:293:LYS:H	1:B:308:GLY:HA2	1.81	0.44
1:C:77:LEU:HD11	1:C:259:LEU:HB3	1.99	0.44
1:C:422:LEU:HB2	1:C:464:VAL:HG22	1.99	0.44
1:C:568:ASP:OD1	1:C:571:CYS:SG	2.74	0.44
1:D:10:ILE:O	1:D:76:ARG:NH2	2.49	0.44
1:D:543:SER:HG	1:D:544:HIS:CE1	2.36	0.44
1:E:508:LYS:HG3	1:F:544:HIS:CG	2.52	0.44
1:E:748:ILE:HG23	1:E:765:LYS:HE2	1.98	0.44
1:G:422:LEU:HB2	1:G:464:VAL:HG22	1.99	0.44
1:I:86:ILE:HG12	1:I:170:LEU:HD11	1.99	0.44
1:I:193:ASN:ND2	1:I:194:LYS:NZ	2.65	0.44
1:I:489:PHE:HD2	1:I:528:ILE:HG23	1.82	0.44
1:K:538:ASP:OD2	1:K:593:LYS:NZ	2.32	0.44
1:K:552:LEU:O	1:K:555:ASN:HB2	2.17	0.44
1:L:48:LEU:HD21	1:L:53:ILE:HD11	1.98	0.44
1:A:293:LYS:H	1:A:308:GLY:HA2	1.81	0.44
1:E:643:LEU:HD23	1:E:643:LEU:HA	1.86	0.44
1:G:179:GLY:O	1:H:468:LEU:HD23	2.16	0.44
1:H:141:PRO:O	1:H:520:ARG:HD2	2.16	0.44

Continued on next page...

For Manuscript Review

Continued from previous page...

Atom-1	Atom-2	Interatomic distance (Å)	Clash overlap (Å)
1:H:748:ILE:HG23	1:H:765:LYS:HE2	1.98	0.44
1:C:10:ILE:O	1:C:76:ARG:NH2	2.50	0.44
1:C:293:LYS:H	1:C:308:GLY:HA2	1.82	0.44
1:D:613:ILE:HB	1:D:663:VAL:HG23	2.00	0.44
1:F:77:LEU:HD11	1:F:259:LEU:HB3	2.00	0.44
1:F:423:PHE:HB3	1:F:473:LEU:HD22	1.98	0.44
2:I:901:ANP:H2'	1:J:710:TYR:CZ	2.53	0.44
1:L:543:SER:HG	1:L:544:HIS:CE1	2.35	0.44
1:A:423:PHE:CE1	1:A:477:LEU:HD13	2.53	0.44
1:B:40:ASP:OD1	1:B:41:ASN:N	2.50	0.44
1:B:430:SER:O	1:B:593:LYS:HD2	2.18	0.44
1:G:293:LYS:H	1:G:308:GLY:HA2	1.82	0.44
1:G:423:PHE:HB3	1:G:473:LEU:HD22	1.99	0.44
1:I:508:LYS:HG3	1:J:544:HIS:CG	2.52	0.44
1:K:40:ASP:OD1	1:K:41:ASN:N	2.50	0.44
1:K:141:PRO:O	1:K:520:ARG:HD2	2.16	0.44
1:K:468:LEU:HD23	1:L:179:GLY:O	2.17	0.44
1:K:594:GLN:HB2	1:K:596:HIS:CE1	2.53	0.44
1:L:423:PHE:HB3	1:L:473:LEU:HD22	2.00	0.44
1:A:422:LEU:HB2	1:A:464:VAL:HG22	1.99	0.44
1:A:468:LEU:HD23	1:B:179:GLY:O	2.17	0.44
1:B:86:ILE:HG12	1:B:170:LEU:HD11	2.00	0.44
1:B:423:PHE:CE1	1:B:477:LEU:HD13	2.53	0.44
1:B:759:ASP:N	1:B:759:ASP:OD1	2.47	0.44
1:D:77:LEU:HD11	1:D:259:LEU:HB3	2.00	0.44
1:D:86:ILE:HG12	1:D:170:LEU:HD11	2.00	0.44
1:E:179:GLY:O	1:F:468:LEU:HD23	2.17	0.44
1:E:293:LYS:H	1:E:308:GLY:HA2	1.82	0.44
1:H:728:ASN:OD1	1:H:729:ILE:N	2.45	0.44
1:D:142:GLU:HG2	1:D:517:VAL:HG22	2.00	0.44
1:D:738:ARG:NH2	1:D:780:TYR:OH	2.51	0.44
1:E:544:HIS:CG	1:F:508:LYS:HG3	2.53	0.44
1:E:738:ARG:NH2	1:E:780:TYR:OH	2.51	0.44
1:G:423:PHE:CE1	1:G:477:LEU:HD13	2.53	0.44
3:G:902:TPP:HM42	1:H:468:LEU:HD11	2.00	0.44
1:H:561:THR:HG22	1:H:586:ILE:HB	2.00	0.44
1:K:179:GLY:O	1:L:468:LEU:HD23	2.17	0.44
1:K:508:LYS:HG3	1:L:544:HIS:CG	2.52	0.44
1:L:423:PHE:CE1	1:L:477:LEU:HD13	2.53	0.44
1:A:423:PHE:HB3	1:A:473:LEU:HD22	1.99	0.43
1:E:423:PHE:CE1	1:E:477:LEU:HD13	2.53	0.43

Continued on next page...

For Manuscript Review

Continued from previous page...

Atom-1	Atom-2	Interatomic distance (Å)	Clash overlap (Å)
1:G:468:LEU:HA	1:G:468:LEU:HD12	1.75	0.43
1:G:468:LEU:HD23	1:H:179:GLY:O	2.17	0.43
1:I:568:ASP:OD1	1:I:571:CYS:SG	2.71	0.43
1:J:748:ILE:HG23	1:J:765:LYS:HE2	2.00	0.43
1:K:533:THR:OG1	1:K:592:ASP:OD2	2.36	0.43
1:A:691:ASP:OD1	1:A:691:ASP:N	2.50	0.43
1:A:738:ARG:NH2	1:A:780:TYR:OH	2.52	0.43
1:B:643:LEU:HD23	1:B:643:LEU:HA	1.87	0.43
1:C:613:ILE:HB	1:C:663:VAL:HG23	2.00	0.43
1:C:691:ASP:N	1:C:691:ASP:OD1	2.50	0.43
1:C:710:TYR:CE2	1:C:711:ARG:HG3	2.52	0.43
1:D:293:LYS:H	1:D:308:GLY:HA2	1.83	0.43
1:D:691:ASP:OD1	1:D:691:ASP:N	2.50	0.43
1:F:86:ILE:HG12	1:F:170:LEU:HD11	2.00	0.43
1:F:691:ASP:OD1	1:F:691:ASP:N	2.50	0.43
1:G:707:LYS:HE2	1:H:703[C]:TRP:CE2	2.53	0.43
2:G:901:ANP:O5'	2:G:901:ANP:H8	2.17	0.43
1:I:617:ASP:OD1	1:I:617:ASP:N	2.51	0.43
1:A:48:LEU:HD21	1:A:53:ILE:HD11	2.00	0.43
1:A:594:GLN:HB2	1:A:596:HIS:CE1	2.54	0.43
1:C:508:LYS:HG3	1:D:544:HIS:CG	2.52	0.43
1:D:423:PHE:CE1	1:D:477:LEU:HD13	2.54	0.43
1:E:77:LEU:HD11	1:E:259:LEU:HB3	2.01	0.43
1:E:86:ILE:HG12	1:E:170:LEU:HD11	2.00	0.43
1:H:635:GLY:O	1:H:639:THR:OG1	2.23	0.43
1:I:689:THR:O	1:I:714:ASN:ND2	2.50	0.43
1:J:674:GLU:OE2	1:J:711:ARG:NH2	2.44	0.43
1:L:293:LYS:H	1:L:308:GLY:HA2	1.83	0.43
1:B:35:GLN:O	1:B:54:LYS:NZ	2.41	0.43
1:C:423:PHE:CE1	1:C:477:LEU:HD13	2.54	0.43
1:I:422:LEU:HB2	1:I:464:VAL:HG22	1.99	0.43
1:L:204:VAL:O	1:L:206:PRO:HD3	2.19	0.43
1:B:105:GLU:OE1	1:B:365:SER:OG	2.28	0.43
1:B:468:LEU:HD12	1:B:468:LEU:HA	1.72	0.43
1:B:543:SER:HG	1:B:544:HIS:CE1	2.37	0.43
1:E:48:LEU:HD21	1:E:53:ILE:HD11	2.00	0.43
1:F:65:PRO:HG3	1:F:295:TRP:H	1.83	0.43
1:F:422:LEU:HB2	1:F:464:VAL:HG22	1.99	0.43
1:G:86:ILE:HG12	1:G:170:LEU:HD11	2.01	0.43
1:I:179:GLY:O	1:J:468:LEU:HD23	2.18	0.43
1:I:423:PHE:HB3	1:I:473:LEU:HD22	2.00	0.43

Continued on next page...

For Manuscript Review

Continued from previous page...

Atom-1	Atom-2	Interatomic distance (Å)	Clash overlap (Å)
2:I:901:ANP:O5’	2:I:901:ANP:H8	2.18	0.43
1:K:635:GLY:O	1:K:639:THR:OG1	2.23	0.43
1:A:97:ALA:O	1:A:101:ASN:ND2	2.39	0.43
2:A:901:ANP:O5’	2:A:901:ANP:H8	2.18	0.43
1:B:533:THR:OG1	1:B:592:ASP:OD2	2.34	0.43
1:C:468:LEU:HD23	1:D:179:GLY:O	2.18	0.43
1:C:544:HIS:CG	1:D:508:LYS:HG3	2.54	0.43
1:D:191:HIS:O	1:D:194:LYS:HG2	2.19	0.43
1:D:728:ASN:OD1	1:D:729:ILE:N	2.45	0.43
1:F:423:PHE:CE1	1:F:477:LEU:HD13	2.54	0.43
1:F:533:THR:OG1	1:F:592:ASP:OD2	2.35	0.43
1:F:594:GLN:HB2	1:F:596:HIS:CE1	2.54	0.43
1:F:635:GLY:O	1:F:639:THR:OG1	2.24	0.43
1:H:204:VAL:O	1:H:206:PRO:HD3	2.19	0.43
1:J:97:ALA:O	1:J:101:ASN:ND2	2.38	0.43
1:J:142:GLU:HG2	1:J:517:VAL:HG22	2.00	0.43
3:K:902:TPP:HM42	1:L:468:LEU:HD11	2.00	0.43
1:C:204:VAL:O	1:C:206:PRO:HD3	2.19	0.43
1:D:204:VAL:O	1:D:206:PRO:HD3	2.19	0.43
1:I:570:ASN:ND2	1:I:600:LEU:O	2.36	0.43
1:J:533:THR:OG1	1:J:592:ASP:OD2	2.37	0.43
1:A:204:VAL:O	1:A:206:PRO:HD3	2.19	0.43
1:A:635:GLY:O	1:A:639:THR:OG1	2.24	0.43
1:B:691:ASP:OD1	1:B:691:ASP:N	2.50	0.43
3:C:902:TPP:HM42	1:D:468:LEU:HD11	2.00	0.43
1:J:423:PHE:CE1	1:J:477:LEU:HD13	2.53	0.43
1:A:333:TRP:O	1:A:336:SER:OG	2.32	0.43
1:C:707:LYS:HE2	1:D:703[B]:TRP:CE2	2.54	0.43
1:E:468:LEU:HD11	3:F:902:TPP:HM42	2.00	0.43
1:G:48:LEU:HD21	1:G:53:ILE:HD11	2.00	0.43
1:G:544:HIS:CG	1:H:508:LYS:HG3	2.54	0.43
1:H:48:LEU:HD21	1:H:53:ILE:HD11	2.00	0.43
1:H:423:PHE:CE1	1:H:477:LEU:HD13	2.53	0.43
1:I:423:PHE:CE1	1:I:477:LEU:HD13	2.54	0.43
1:J:468:LEU:HA	1:J:468:LEU:HD12	1.73	0.43
1:J:691:ASP:N	1:J:691:ASP:OD1	2.50	0.43
1:K:738:ARG:NH2	1:K:780:TYR:OH	2.51	0.43
1:L:594:GLN:HB2	1:L:596:HIS:CE1	2.53	0.43
1:A:643:LEU:HD23	1:A:643:LEU:HA	1.89	0.43
1:C:468:LEU:HD12	1:C:468:LEU:HA	1.75	0.43
1:E:563:ILE:HD13	1:E:588:VAL:HB	2.01	0.43

Continued on next page...

For Manuscript Review

Continued from previous page...

Atom-1	Atom-2	Interatomic distance (Å)	Clash overlap (Å)
1:E:707:LYS:HE2	1:F:703[B]:TRP:CE2	2.54	0.43
1:F:204:VAL:O	1:F:206:PRO:HD3	2.19	0.43
1:F:689:THR:O	1:F:714:ASN:ND2	2.51	0.43
1:G:204:VAL:O	1:G:206:PRO:HD3	2.19	0.43
1:J:10:ILE:O	1:J:76:ARG:NH2	2.49	0.43
1:J:293:LYS:H	1:J:308:GLY:HA2	1.83	0.43
1:K:423:PHE:CE1	1:K:477:LEU:HD13	2.53	0.43
1:C:773:HIS:O	1:C:777:GLN:HG2	2.19	0.42
1:D:423:PHE:HB3	1:D:473:LEU:HD22	2.00	0.42
1:E:204:VAL:O	1:E:206:PRO:HD3	2.19	0.42
1:I:10:ILE:O	1:I:76:ARG:NH2	2.49	0.42
1:I:48:LEU:HD21	1:I:53:ILE:HD11	2.00	0.42
1:I:204:VAL:O	1:I:206:PRO:HD3	2.18	0.42
1:K:468:LEU:HD11	3:L:902:TPP:HM42	2.00	0.42
1:L:65:PRO:HG3	1:L:295:TRP:H	1.83	0.42
1:A:773:HIS:O	1:A:777:GLN:HG2	2.19	0.42
2:A:901:ANP:H2'	1:B:710:TYR:CZ	2.55	0.42
1:D:178:ASP:HB3	1:D:210:LEU:HD12	2.01	0.42
1:E:691:ASP:N	1:E:691:ASP:OD1	2.50	0.42
1:E:773:HIS:O	1:E:777:GLN:HG2	2.20	0.42
1:I:691:ASP:OD1	1:I:691:ASP:N	2.50	0.42
1:I:748:ILE:HG23	1:I:765:LYS:HE2	2.01	0.42
1:K:204:VAL:O	1:K:206:PRO:HD3	2.19	0.42
1:K:544:HIS:CG	1:L:508:LYS:HG3	2.54	0.42
1:L:691:ASP:N	1:L:691:ASP:OD1	2.50	0.42
1:A:636:ASP:OD1	1:A:637:VAL:N	2.53	0.42
1:B:204:VAL:O	1:B:206:PRO:HD3	2.19	0.42
1:C:31:LEU:HD22	1:C:122:LEU:HD21	2.02	0.42
1:C:191:HIS:O	1:C:194:LYS:HG2	2.19	0.42
1:C:748:ILE:HG23	1:C:765:LYS:HE2	2.00	0.42
1:D:27:ALA:HB2	1:D:342:LEU:HD13	2.01	0.42
1:D:672:ASP:OD1	1:D:672:ASP:N	2.53	0.42
1:E:97:ALA:O	1:E:101:ASN:ND2	2.37	0.42
1:G:691:ASP:OD1	1:G:691:ASP:N	2.50	0.42
1:J:672:ASP:N	1:J:672:ASP:OD1	2.52	0.42
1:K:48:LEU:HD21	1:K:53:ILE:HD11	2.00	0.42
1:K:178:ASP:OD2	1:K:219:THR:HG21	2.19	0.42
1:L:178:ASP:OD2	1:L:219:THR:OG1	2.28	0.42
1:L:748:ILE:HG23	1:L:765:LYS:HE2	2.01	0.42
1:A:40:ASP:OD1	1:A:41:ASN:N	2.50	0.42
1:A:430:SER:O	1:A:593:LYS:HD2	2.20	0.42

Continued on next page...

For Manuscript Review

Continued from previous page...

Atom-1	Atom-2	Interatomic distance (Å)	Clash overlap (Å)
1:B:710:TYR:CE2	1:B:711:ARG:HG3	2.55	0.42
1:C:48:LEU:HD21	1:C:53:ILE:HD11	2.00	0.42
1:G:738:ARG:NH2	1:G:780:TYR:OH	2.52	0.42
1:H:705:ILE:HG22	1:H:718:LEU:HD21	2.02	0.42
1:I:738:ARG:NH2	1:I:780:TYR:OH	2.53	0.42
1:J:617:ASP:OD1	1:J:617:ASP:N	2.52	0.42
1:E:570:ASN:ND2	1:E:600:LEU:O	2.34	0.42
1:F:97:ALA:O	1:F:101:ASN:ND2	2.39	0.42
1:H:77:LEU:HD11	1:H:259:LEU:HB3	2.01	0.42
1:H:672:ASP:N	1:H:672:ASP:OD1	2.51	0.42
1:I:293:LYS:H	1:I:308:GLY:HA2	1.83	0.42
1:K:703[B]:TRP:CE2	1:L:707:LYS:HE2	2.54	0.42
3:A:902:TPP:HM42	1:B:468:LEU:HD11	2.00	0.42
1:C:423:PHE:HB3	1:C:473:LEU:HD22	2.00	0.42
1:C:672:ASP:N	1:C:672:ASP:OD1	2.53	0.42
1:C:697:ASN:ND2	1:C:746:LEU:HD11	2.35	0.42
1:D:748:ILE:HG23	1:D:765:LYS:HE2	2.00	0.42
1:E:26:ARG:NH2	1:E:342:LEU:HD21	2.35	0.42
1:F:314:GLN:OE1	1:F:314:GLN:N	2.51	0.42
1:F:643:LEU:HD23	1:F:643:LEU:HA	1.89	0.42
1:F:672:ASP:N	1:F:672:ASP:OD1	2.52	0.42
1:F:773:HIS:O	1:F:777:GLN:HG2	2.20	0.42
1:H:533:THR:OG1	1:H:592:ASP:OD2	2.37	0.42
1:I:468:LEU:HD23	1:J:179:GLY:O	2.19	0.42
1:I:544:HIS:CG	1:J:508:LYS:HG3	2.54	0.42
3:I:902:TPP:HM42	1:J:468:LEU:HD11	2.01	0.42
1:J:191:HIS:O	1:J:194:LYS:HG2	2.19	0.42
1:J:204:VAL:O	1:J:206:PRO:HD3	2.19	0.42
1:K:707:LYS:HE2	1:L:703[B]:TRP:CD2	2.54	0.42
1:B:191:HIS:O	1:B:194:LYS:HG2	2.20	0.42
1:B:220:LEU:HD12	1:B:221:LEU:N	2.35	0.42
2:C:901:ANP:H2'	1:D:710:TYR:CZ	2.55	0.42
1:E:40:ASP:OD1	1:E:41:ASN:N	2.50	0.42
1:E:594:GLN:HB2	1:E:596:HIS:CE1	2.53	0.42
1:G:40:ASP:OD1	1:G:41:ASN:N	2.50	0.42
1:G:617:ASP:OD1	1:G:617:ASP:N	2.51	0.42
1:J:594:GLN:HB2	1:J:596:HIS:CE1	2.55	0.42
1:K:26:ARG:NH2	1:K:342:LEU:HD21	2.35	0.42
1:L:145:GLY:HA3	1:L:365:SER:HA	2.02	0.42
1:A:544:HIS:CG	1:B:508:LYS:HG3	2.54	0.42
1:A:563:ILE:HD13	1:A:588:VAL:HB	2.01	0.42

Continued on next page...

For Manuscript Review

Continued from previous page...

Atom-1	Atom-2	Interatomic distance (Å)	Clash overlap (Å)
1:A:695:ILE:HG13	1:A:750:VAL:HG22	2.01	0.42
1:B:77:LEU:HD11	1:B:259:LEU:HB3	2.02	0.42
1:B:594:GLN:HB2	1:B:596:HIS:CE1	2.54	0.42
1:C:153:LEU:HD13	1:C:153:LEU:HA	1.93	0.42
1:I:191:HIS:O	1:I:194:LYS:HG2	2.19	0.42
1:I:533:THR:OG1	1:I:592:ASP:OD2	2.38	0.42
1:I:543:SER:O	1:I:726:VAL:HG21	2.20	0.42
1:L:86:ILE:HG12	1:L:170:LEU:HD11	2.02	0.42
1:B:773:HIS:O	1:B:777:GLN:HG2	2.20	0.42
1:D:48:LEU:HD21	1:D:53:ILE:HD11	2.01	0.42
1:E:672:ASP:N	1:E:672:ASP:OD1	2.52	0.42
1:F:515:ARG:NH1	1:F:516:GLU:OE2	2.53	0.42
1:G:97:ALA:O	1:G:101:ASN:ND2	2.39	0.42
1:I:672:ASP:OD1	1:I:672:ASP:N	2.53	0.42
1:K:601:ASP:OD1	1:K:601:ASP:N	2.39	0.42
1:L:515:ARG:NH1	1:L:516:GLU:OE2	2.53	0.42
1:A:65:PRO:HG3	1:A:295:TRP:H	1.84	0.42
1:A:533:THR:OG1	1:A:592:ASP:OD2	2.36	0.42
1:B:515:ARG:NH1	1:B:516:GLU:OE2	2.53	0.42
1:B:672:ASP:OD1	1:B:672:ASP:N	2.52	0.42
1:D:141:PRO:HG3	1:D:509:TRP:CD2	2.55	0.42
1:D:697:ASN:ND2	1:D:746:LEU:HD11	2.35	0.42
1:D:773:HIS:O	1:D:777:GLN:HG2	2.20	0.42
1:F:40:ASP:OD1	1:F:41:ASN:N	2.50	0.42
1:F:599:TYR:HB3	1:F:618:TRP:HZ2	1.85	0.42
1:G:594:GLN:HB2	1:G:596:HIS:CE1	2.53	0.42
1:I:632:ALA:HA	1:I:661:VAL:O	2.20	0.42
1:J:48:LEU:HD21	1:J:53:ILE:HD11	2.02	0.42
1:J:103:TRP:CZ2	1:J:117:GLN:HA	2.55	0.42
1:K:773:HIS:O	1:K:777:GLN:HG2	2.20	0.42
1:L:468:LEU:HD12	1:L:468:LEU:HA	1.74	0.42
1:D:153:LEU:HD13	1:D:153:LEU:HA	1.93	0.41
1:D:166:ASP:OD2	1:D:480:TYR:OH	2.23	0.41
1:D:468:LEU:HD12	1:D:468:LEU:HA	1.73	0.41
1:E:31:LEU:HD22	1:E:122:LEU:HD21	2.02	0.41
1:E:632:ALA:HA	1:E:661:VAL:O	2.20	0.41
1:I:97:ALA:O	1:I:101:ASN:ND2	2.38	0.41
1:J:65:PRO:HG3	1:J:295:TRP:H	1.85	0.41
1:J:141:PRO:HG3	1:J:509:TRP:CD2	2.55	0.41
1:K:672:ASP:OD1	1:K:672:ASP:N	2.52	0.41
1:L:636:ASP:OD1	1:L:637:VAL:N	2.52	0.41

Continued on next page...

For Manuscript Review

Continued from previous page...

Atom-1	Atom-2	Interatomic distance (Å)	Clash overlap (Å)
1:B:153:LEU:HD13	1:B:153:LEU:HA	1.94	0.41
1:G:636:ASP:OD1	1:G:637:VAL:N	2.52	0.41
1:H:773:HIS:O	1:H:777:GLN:HG2	2.20	0.41
1:I:594:GLN:HB2	1:I:596:HIS:CE1	2.55	0.41
1:L:191:HIS:O	1:L:194:LYS:HG2	2.20	0.41
1:B:563:ILE:HD13	1:B:588:VAL:HB	2.03	0.41
1:B:632:ALA:HA	1:B:661:VAL:O	2.21	0.41
1:D:594:GLN:HB2	1:D:596:HIS:CE1	2.55	0.41
3:E:902:TPP:HM42	1:F:468:LEU:HD11	2.01	0.41
1:F:616:TRP:O	1:F:620:SER:OG	2.32	0.41
1:G:31:LEU:HD22	1:G:122:LEU:HD21	2.02	0.41
1:G:703[C]:TRP:HD1	2:G:901:ANP:HO3'	1.69	0.41
1:G:773:HIS:O	1:G:777:GLN:HG2	2.19	0.41
1:J:517:VAL:HG11	1:J:520:ARG:HG3	2.02	0.41
1:J:543:SER:O	1:J:726:VAL:HG21	2.20	0.41
1:A:748:ILE:HG23	1:A:765:LYS:HE2	2.01	0.41
1:C:617:ASP:OD1	1:C:617:ASP:N	2.51	0.41
1:E:689:THR:O	1:E:714:ASN:ND2	2.54	0.41
1:F:748:ILE:HG23	1:F:765:LYS:HE2	2.01	0.41
1:G:643:LEU:HA	1:G:643:LEU:HD23	1.89	0.41
1:G:697:ASN:ND2	1:G:746:LEU:HD11	2.36	0.41
1:H:141:PRO:HG3	1:H:509:TRP:CD2	2.56	0.41
1:H:220:LEU:HD12	1:H:221:LEU:N	2.34	0.41
1:H:599:TYR:HB3	1:H:618:TRP:HZ2	1.85	0.41
1:I:697:ASN:ND2	1:I:746:LEU:HD11	2.36	0.41
1:K:31:LEU:HD22	1:K:122:LEU:HD21	2.02	0.41
1:L:40:ASP:OD1	1:L:41:ASN:N	2.50	0.41
1:L:533:THR:OG1	1:L:592:ASP:OD2	2.37	0.41
1:L:632:ALA:HA	1:L:661:VAL:O	2.21	0.41
1:L:697:ASN:ND2	1:L:746:LEU:HD11	2.36	0.41
1:A:672:ASP:N	1:A:672:ASP:OD1	2.52	0.41
1:B:103:TRP:CZ2	1:B:117:GLN:HA	2.56	0.41
1:C:153:LEU:HB3	1:C:180:GLU:HG3	2.03	0.41
1:C:738:ARG:NH2	1:C:780:TYR:OH	2.53	0.41
1:D:65:PRO:HG3	1:D:295:TRP:H	1.85	0.41
1:D:103:TRP:CZ2	1:D:117:GLN:HA	2.55	0.41
1:D:178:ASP:OD2	1:D:219:THR:HG21	2.16	0.41
1:G:212:GLY:HA2	1:G:222:SER:CB	2.51	0.41
1:H:703[C]:TRP:HD1	2:H:901:ANP:O3'	2.04	0.41
1:I:141:PRO:HG3	1:I:509:TRP:CD2	2.56	0.41
1:I:773:HIS:O	1:I:777:GLN:HG2	2.19	0.41

Continued on next page...

For Manuscript Review

Continued from previous page...

Atom-1	Atom-2	Interatomic distance (Å)	Clash overlap (Å)
1:J:212:GLY:HA2	1:J:222:SER:CB	2.51	0.41
1:K:141:PRO:HG3	1:K:509:TRP:CD2	2.56	0.41
1:L:27:ALA:HB2	1:L:342:LEU:HD13	2.01	0.41
1:B:145:GLY:HA3	1:B:365:SER:HA	2.03	0.41
1:B:689:THR:O	1:B:714:ASN:ND2	2.54	0.41
1:C:40:ASP:OD1	1:C:41:ASN:N	2.51	0.41
1:C:85:LEU:CD2	1:C:171:ILE:HB	2.50	0.41
1:C:533:THR:OG1	1:C:592:ASP:OD2	2.36	0.41
1:C:632:ALA:HA	1:C:661:VAL:O	2.21	0.41
1:E:105:GLU:OE1	1:E:365:SER:OG	2.28	0.41
1:E:191:HIS:O	1:E:194:LYS:HG2	2.21	0.41
1:F:333:TRP:O	1:F:336:SER:OG	2.32	0.41
1:H:31:LEU:HD22	1:H:122:LEU:HD21	2.02	0.41
1:I:81:LEU:HB2	1:I:83:LEU:HG	2.01	0.41
1:I:710:TYR:CZ	2:J:901:ANP:H2’	2.55	0.41
1:J:85:LEU:CD2	1:J:171:ILE:HB	2.50	0.41
1:J:105:GLU:OE1	1:J:365:SER:OG	2.31	0.41
1:K:212:GLY:HA2	1:K:222:SER:CB	2.51	0.41
1:K:543:SER:O	1:K:726:VAL:HG21	2.21	0.41
1:A:191:HIS:O	1:A:194:LYS:HG2	2.21	0.41
1:A:599:TYR:HB3	1:A:618:TRP:HZ2	1.85	0.41
1:B:180:GLU:HG2	1:B:186:LEU:HD13	2.03	0.41
1:D:40:ASP:OD1	1:D:41:ASN:N	2.50	0.41
1:D:220:LEU:HD12	1:D:221:LEU:N	2.34	0.41
1:F:191:HIS:O	1:F:194:LYS:HG2	2.21	0.41
1:G:65:PRO:HG3	1:G:295:TRP:H	1.85	0.41
1:G:191:HIS:O	1:G:194:LYS:HG2	2.21	0.41
1:G:748:ILE:HG23	1:G:765:LYS:HE2	2.03	0.41
1:H:632:ALA:HA	1:H:661:VAL:O	2.20	0.41
1:I:563:ILE:HD13	1:I:588:VAL:HB	2.02	0.41
1:I:565:LEU:HG	1:I:590:VAL:HB	2.02	0.41
1:J:632:ALA:HA	1:J:661:VAL:O	2.21	0.41
1:J:697:ASN:ND2	1:J:746:LEU:HD11	2.36	0.41
1:A:707:LYS:HE2	1:B:703[C]:TRP:CE2	2.56	0.41
1:A:707:LYS:HE2	1:B:703[C]:TRP:CD2	2.56	0.41
1:B:26:ARG:HH22	1:B:342:LEU:HD21	1.86	0.41
1:C:141:PRO:HG3	1:C:509:TRP:CD2	2.56	0.41
1:D:533:THR:OG1	1:D:592:ASP:OD2	2.36	0.41
1:G:145:GLY:HA3	1:G:365:SER:HA	2.03	0.41
1:H:103:TRP:CZ2	1:H:117:GLN:HA	2.56	0.41
1:H:212:GLY:HA2	1:H:222:SER:CB	2.51	0.41

Continued on next page...

For Manuscript Review

Continued from previous page...

Atom-1	Atom-2	Interatomic distance (Å)	Clash overlap (Å)
1:I:333:TRP:O	1:I:336:SER:OG	2.31	0.41
1:I:422:LEU:HD13	1:I:422:LEU:HA	1.97	0.41
1:J:773:HIS:O	1:J:777:GLN:HG2	2.20	0.41
1:L:643:LEU:HA	1:L:643:LEU:HD23	1.89	0.41
1:L:773:HIS:O	1:L:777:GLN:HG2	2.20	0.41
1:A:31:LEU:HD22	1:A:122:LEU:HD21	2.01	0.41
1:A:220:LEU:HD12	1:A:221:LEU:N	2.36	0.41
1:A:616:TRP:O	1:A:620:SER:OG	2.32	0.41
1:B:141:PRO:HG3	1:B:509:TRP:CD2	2.55	0.41
1:D:632:ALA:HA	1:D:661:VAL:O	2.21	0.41
1:E:220:LEU:HD12	1:E:221:LEU:N	2.35	0.41
1:F:145:GLY:HA3	1:F:365:SER:HA	2.02	0.41
1:F:617:ASP:OD1	1:F:617:ASP:N	2.52	0.41
1:G:14:SER:HB3	1:G:17:GLU:HG2	2.02	0.41
1:I:31:LEU:HD22	1:I:122:LEU:HD21	2.03	0.41
1:I:153:LEU:HB3	1:I:180:GLU:HG3	2.03	0.41
1:I:212:GLY:HA2	1:I:222:SER:CB	2.51	0.41
1:K:153:LEU:HB3	1:K:180:GLU:HG3	2.03	0.41
1:K:689:THR:O	1:K:714:ASN:ND2	2.54	0.41
1:L:103:TRP:CZ2	1:L:117:GLN:HA	2.56	0.41
1:L:212:GLY:HA2	1:L:222:SER:CB	2.51	0.41
1:A:14:SER:HB3	1:A:17:GLU:HG2	2.02	0.41
1:A:697:ASN:ND2	1:A:746:LEU:HD11	2.36	0.41
1:D:26:ARG:NH2	1:D:342:LEU:HD21	2.36	0.41
1:D:31:LEU:HD22	1:D:122:LEU:HD21	2.03	0.41
1:E:26:ARG:HH22	1:E:342:LEU:HD21	1.86	0.41
1:E:178:ASP:OD2	1:E:219:THR:OG1	2.27	0.41
1:E:212:GLY:HA2	1:E:222:SER:CB	2.51	0.41
1:F:220:LEU:HD12	1:F:221:LEU:N	2.36	0.41
1:G:141:PRO:HG3	1:G:509:TRP:CD2	2.56	0.41
1:G:563:ILE:HD13	1:G:588:VAL:HB	2.03	0.41
1:H:145:GLY:HA3	1:H:365:SER:HA	2.03	0.41
1:J:27:ALA:HB2	1:J:342:LEU:HD13	2.01	0.41
1:L:141:PRO:HG3	1:L:509:TRP:CD2	2.56	0.41
1:L:617:ASP:OD1	1:L:617:ASP:N	2.52	0.41
1:A:613:ILE:HB	1:A:663:VAL:HG23	2.04	0.40
1:F:103:TRP:CZ2	1:F:117:GLN:HA	2.56	0.40
1:F:697:ASN:ND2	1:F:746:LEU:HD11	2.36	0.40
1:G:422:LEU:HD13	1:G:422:LEU:HA	1.97	0.40
1:G:689:THR:O	1:G:714:ASN:ND2	2.53	0.40
1:H:154:GLY:HA2	1:H:186:LEU:HB2	2.04	0.40

Continued on next page...

For Manuscript Review

Continued from previous page...

Atom-1	Atom-2	Interatomic distance (Å)	Clash overlap (Å)
1:J:31:LEU:HD22	1:J:122:LEU:HD21	2.03	0.40
1:L:97:ALA:O	1:L:101:ASN:ND2	2.39	0.40
1:L:599:TYR:HB3	1:L:618:TRP:HZ2	1.85	0.40
1:B:31:LEU:HD22	1:B:122:LEU:HD21	2.03	0.40
1:D:544:HIS:HA	1:D:726:VAL:HG21	2.03	0.40
2:E:901:ANP:H2'	1:F:710:TYR:CZ	2.56	0.40
1:H:191:HIS:O	1:H:194:LYS:HG2	2.20	0.40
1:H:556:LYS:HD2	1:H:556:LYS:HA	1.89	0.40
1:I:468:LEU:HD12	1:I:468:LEU:HA	1.75	0.40
1:K:77:LEU:HD11	1:K:259:LEU:HB3	2.03	0.40
1:L:14:SER:HB3	1:L:17:GLU:HG2	2.03	0.40
1:A:145:GLY:HA3	1:A:365:SER:HA	2.03	0.40
1:C:707:LYS:HE2	1:D:703[B]:TRP:CD2	2.57	0.40
1:D:617:ASP:OD1	1:D:617:ASP:N	2.53	0.40
1:E:141:PRO:HG3	1:E:509:TRP:CD2	2.56	0.40
1:F:141:PRO:HG3	1:F:509:TRP:CD2	2.56	0.40
1:G:533:THR:OG1	1:G:592:ASP:OD2	2.38	0.40
1:J:178:ASP:OD2	1:J:219:THR:HG21	2.19	0.40
1:K:220:LEU:HD12	1:K:221:LEU:N	2.36	0.40
1:L:422:LEU:HD13	1:L:422:LEU:HA	1.98	0.40
1:A:617:ASP:N	1:A:617:ASP:OD1	2.51	0.40
1:A:632:ALA:HA	1:A:661:VAL:O	2.20	0.40
1:B:193:ASN:OD1	1:B:194:LYS:NZ	2.51	0.40
1:C:422:LEU:HD13	1:C:422:LEU:HA	1.97	0.40
1:F:563:ILE:HD13	1:F:588:VAL:HB	2.03	0.40
1:H:333:TRP:O	1:H:336:SER:OG	2.33	0.40
1:J:26:ARG:NH2	1:J:342:LEU:HD21	2.37	0.40
1:K:191:HIS:O	1:K:194:LYS:HG2	2.20	0.40
1:K:636:ASP:OD1	1:K:637:VAL:N	2.53	0.40
1:A:81:LEU:HB2	1:A:83:LEU:HG	2.04	0.40
1:A:141:PRO:HG3	1:A:509:TRP:CD2	2.56	0.40
1:B:27:ALA:HB2	1:B:342:LEU:HD13	2.03	0.40
1:C:212:GLY:HA2	1:C:222:SER:CB	2.51	0.40
1:E:178:ASP:OD2	1:E:219:THR:HG21	2.19	0.40
1:F:9:ASP:N	1:F:9:ASP:OD1	2.55	0.40
1:H:543:SER:O	1:H:726:VAL:HG21	2.22	0.40
1:H:703[C]:TRP:HD1	2:H:901:ANP:HO3'	1.69	0.40
1:I:26:ARG:NH2	1:I:342:LEU:HD21	2.35	0.40
1:I:178:ASP:OD2	1:I:219:THR:HG21	2.18	0.40
1:L:9:ASP:N	1:L:9:ASP:OD1	2.55	0.40
1:L:31:LEU:HD22	1:L:122:LEU:HD21	2.03	0.40

For Manuscript Review

There are no symmetry-related clashes.

5.3 Torsion angles (i)

5.3.1 Protein backbone (i)

In the following table, the Percentiles column shows the percent Ramachandran outliers of the chain as a percentile score with respect to all PDB entries followed by that with respect to all EM entries.

The Analysed column shows the number of residues for which the backbone conformation was analysed, and the total number of residues.

Mol	Chain	Analysed	Favoured	Allowed	Outliers	Percentiles	
1	A	787/796 (99%)	757 (96%)	30 (4%)	0	100	100
1	B	787/796 (99%)	759 (96%)	28 (4%)	0	100	100
1	C	787/796 (99%)	758 (96%)	29 (4%)	0	100	100
1	D	787/796 (99%)	759 (96%)	28 (4%)	0	100	100
1	E	787/796 (99%)	761 (97%)	26 (3%)	0	100	100
1	F	787/796 (99%)	760 (97%)	27 (3%)	0	100	100
1	G	787/796 (99%)	762 (97%)	25 (3%)	0	100	100
1	H	787/796 (99%)	761 (97%)	26 (3%)	0	100	100
1	I	787/796 (99%)	760 (97%)	27 (3%)	0	100	100
1	J	787/796 (99%)	758 (96%)	29 (4%)	0	100	100
1	K	787/796 (99%)	761 (97%)	26 (3%)	0	100	100
1	L	787/796 (99%)	759 (96%)	28 (4%)	0	100	100
All	All	9444/9552 (99%)	9115 (96%)	329 (4%)	0	100	100

There are no Ramachandran outliers to report.

5.3.2 Protein sidechains (i)

In the following table, the Percentiles column shows the percent sidechain outliers of the chain as a percentile score with respect to all PDB entries followed by that with respect to all EM entries.

The Analysed column shows the number of residues for which the sidechain conformation was analysed, and the total number of residues.

For Manuscript Review

Mol	Chain	Analysed	Rotameric	Outliers	Percentiles	
1	A	664/670 (99%)	663 (100%)	1 (0%)	93	97
1	B	664/670 (99%)	663 (100%)	1 (0%)	93	97
1	C	664/670 (99%)	664 (100%)	0	100	100
1	D	664/670 (99%)	663 (100%)	1 (0%)	93	97
1	E	664/670 (99%)	663 (100%)	1 (0%)	93	97
1	F	664/670 (99%)	663 (100%)	1 (0%)	93	97
1	G	664/670 (99%)	664 (100%)	0	100	100
1	H	664/670 (99%)	664 (100%)	0	100	100
1	I	664/670 (99%)	664 (100%)	0	100	100
1	J	664/670 (99%)	664 (100%)	0	100	100
1	K	664/670 (99%)	664 (100%)	0	100	100
1	L	664/670 (99%)	664 (100%)	0	100	100
All	All	7968/8040 (99%)	7963 (100%)	5 (0%)	93	97

All (5) residues with a non-rotameric sidechain are listed below:

Mol	Chain	Res	Type
1	A	666	LEU
1	B	666	LEU
1	D	666	LEU
1	E	666	LEU
1	F	666	LEU

Sometimes sidechains can be flipped to improve hydrogen bonding and reduce clashes. All (4) such sidechains are listed below:

Mol	Chain	Res	Type
1	C	45	GLN
1	D	45	GLN
1	I	45	GLN
1	J	45	GLN

5.3.3 RNA (i)

There are no RNA molecules in this entry.

For Manuscript Review

5.4 Non-standard residues in protein, DNA, RNA chains (i)

There are no non-standard protein/DNA/RNA residues in this entry.

5.5 Carbohydrates (i)

There are no monosaccharides in this entry.

5.6 Ligand geometry (i)

Of 36 ligands modelled in this entry, 12 are monoatomic - leaving 24 for Mogul analysis.

In the following table, the Counts columns list the number of bonds (or angles) for which Mogul statistics could be retrieved, the number of bonds (or angles) that are observed in the model and the number of bonds (or angles) that are defined in the Chemical Component Dictionary. The Link column lists molecule types, if any, to which the group is linked. The Z score for a bond length (or angle) is the number of standard deviations the observed value is removed from the expected value. A bond length (or angle) with $|Z| > 2$ is considered an outlier worth inspection. RMSZ is the root-mean-square of all Z scores of the bond lengths (or angles).

Mol	Type	Chain	Res	Link	Bond lengths			Bond angles		
					Counts	RMSZ	$\#\|Z\| > 2$	Counts	RMSZ	$\#\|Z\| > 2$
3	TPP	J	902	4	22,27,27	0.60	0	29,40,40	0.89	2 (6%)
2	ANP	G	901	-	29,33,33	1.10	4 (13%)	31,52,52	1.07	2 (6%)
3	TPP	I	902	4	22,27,27	0.56	0	29,40,40	0.73	2 (6%)
2	ANP	L	901	-	29,33,33	1.10	4 (13%)	31,52,52	1.13	2 (6%)
2	ANP	F	901	-	29,33,33	1.10	4 (13%)	31,52,52	1.11	2 (6%)
3	TPP	L	902	4	22,27,27	0.59	0	29,40,40	0.89	2 (6%)
3	TPP	D	902	4	22,27,27	0.61	0	29,40,40	0.89	2 (6%)
3	TPP	G	902	4	22,27,27	0.56	0	29,40,40	0.74	2 (6%)
3	TPP	E	902	4	22,27,27	0.57	0	29,40,40	0.74	2 (6%)
2	ANP	B	901	1	29,33,33	1.07	4 (13%)	31,52,52	1.02	2 (6%)
2	ANP	I	901	-	29,33,33	1.10	4 (13%)	31,52,52	1.06	2 (6%)
2	ANP	H	901	-	29,33,33	1.10	4 (13%)	31,52,52	1.07	2 (6%)
3	TPP	A	902	4	22,27,27	0.56	0	29,40,40	0.73	2 (6%)
3	TPP	F	902	4	22,27,27	0.59	0	29,40,40	0.89	2 (6%)
2	ANP	A	901	-	29,33,33	1.10	4 (13%)	31,52,52	1.08	2 (6%)
2	ANP	D	901	1	29,33,33	1.09	4 (13%)	31,52,52	1.02	3 (9%)
2	ANP	J	901	-	29,33,33	1.10	4 (13%)	31,52,52	1.07	3 (9%)
2	ANP	E	901	-	29,33,33	1.09	4 (13%)	31,52,52	1.06	2 (6%)

For Manuscript Review

Mol	Type	Chain	Res	Link	Bond lengths			Bond angles		
					Counts	RMSZ	#|Z| > 2	Counts	RMSZ	#|Z| > 2
3	TPP	H	902	4	22,27,27	0.60	0	29,40,40	0.89	2 (6%)
2	ANP	C	901	-	29,33,33	1.10	4 (13%)	31,52,52	1.06	2 (6%)
3	TPP	B	902	4	22,27,27	0.59	0	29,40,40	0.89	2 (6%)
2	ANP	K	901	-	29,33,33	1.09	4 (13%)	31,52,52	1.04	2 (6%)
3	TPP	C	902	4	22,27,27	0.57	0	29,40,40	0.74	2 (6%)
3	TPP	K	902	4	22,27,27	0.57	0	29,40,40	0.73	2 (6%)

In the following table, the Chirals column lists the number of chiral outliers, the number of chiral centers analysed, the number of these observed in the model and the number defined in the Chemical Component Dictionary. Similar counts are reported in the Torsion and Rings columns. '-' means no outliers of that kind were identified.

Mol	Type	Chain	Res	Link	Chirals	Torsions	Rings
3	TPP	J	902	4	-	5/16/17/17	0/2/2/2
2	ANP	G	901	-	-	4/14/38/38	0/3/3/3
3	TPP	I	902	4	-	8/16/17/17	0/2/2/2
2	ANP	L	901	-	-	4/14/38/38	0/3/3/3
2	ANP	F	901	-	-	4/14/38/38	0/3/3/3
3	TPP	L	902	4	-	5/16/17/17	0/2/2/2
3	TPP	D	902	4	-	5/16/17/17	0/2/2/2
3	TPP	G	902	4	-	7/16/17/17	0/2/2/2
3	TPP	E	902	4	-	6/16/17/17	0/2/2/2
2	ANP	B	901	1	-	4/14/38/38	0/3/3/3
2	ANP	I	901	-	-	4/14/38/38	0/3/3/3
2	ANP	H	901	-	-	4/14/38/38	0/3/3/3
3	TPP	A	902	4	-	8/16/17/17	0/2/2/2
3	TPP	F	902	4	-	5/16/17/17	0/2/2/2
2	ANP	A	901	-	-	4/14/38/38	0/3/3/3
2	ANP	D	901	1	-	4/14/38/38	0/3/3/3
2	ANP	J	901	-	-	4/14/38/38	0/3/3/3
2	ANP	E	901	-	-	4/14/38/38	0/3/3/3
3	TPP	H	902	4	-	5/16/17/17	0/2/2/2
2	ANP	C	901	-	-	4/14/38/38	0/3/3/3
3	TPP	B	902	4	-	5/16/17/17	0/2/2/2
2	ANP	K	901	-	-	4/14/38/38	0/3/3/3
3	TPP	C	902	4	-	8/16/17/17	0/2/2/2

Continued on next page...

For Manuscript Review

Continued from previous page...

Mol	Type	Chain	Res	Link	Chirals	Torsions	Rings
3	TPP	K	902	4	-	6/16/17/17	0/2/2/2

All (48) bond length outliers are listed below:

Mol	Chain	Res	Type	Atoms	Z	Observed(Å)	Ideal(Å)
2	L	901	ANP	PB-O3A	-2.76	1.55	1.59
2	I	901	ANP	PB-O3A	-2.75	1.55	1.59
2	F	901	ANP	PB-O3A	-2.75	1.55	1.59
2	H	901	ANP	PB-O3A	-2.74	1.55	1.59
2	G	901	ANP	PB-O3A	-2.73	1.55	1.59
2	E	901	ANP	PB-O3A	-2.73	1.55	1.59
2	C	901	ANP	PB-O3A	-2.72	1.55	1.59
2	J	901	ANP	PB-O3A	-2.72	1.55	1.59
2	A	901	ANP	PB-O3A	-2.70	1.55	1.59
2	K	901	ANP	PB-O3A	-2.65	1.55	1.59
2	A	901	ANP	PG-N3B	2.42	1.69	1.63
2	D	901	ANP	PG-N3B	2.41	1.69	1.63
2	F	901	ANP	PG-N3B	2.40	1.69	1.63
2	E	901	ANP	PG-N3B	2.40	1.69	1.63
2	B	901	ANP	PG-N3B	2.39	1.69	1.63
2	D	901	ANP	PG-O1G	2.39	1.49	1.46
2	C	901	ANP	PG-N3B	2.39	1.69	1.63
2	H	901	ANP	PG-N3B	2.39	1.69	1.63
2	G	901	ANP	PG-N3B	2.37	1.69	1.63
2	H	901	ANP	PG-O1G	2.37	1.49	1.46
2	I	901	ANP	PG-N3B	2.36	1.69	1.63
2	L	901	ANP	PG-N3B	2.36	1.69	1.63
2	K	901	ANP	PG-N3B	2.36	1.69	1.63
2	J	901	ANP	PG-N3B	2.36	1.69	1.63
2	B	901	ANP	PG-O1G	2.36	1.49	1.46
2	J	901	ANP	PG-O1G	2.34	1.49	1.46
2	K	901	ANP	PG-O1G	2.32	1.49	1.46
2	G	901	ANP	PG-O1G	2.32	1.49	1.46
2	C	901	ANP	PG-O1G	2.31	1.49	1.46
2	A	901	ANP	PG-O1G	2.30	1.49	1.46
2	F	901	ANP	PG-O1G	2.30	1.49	1.46
2	D	901	ANP	PB-O3A	-2.29	1.56	1.59
2	L	901	ANP	PG-O1G	2.29	1.49	1.46
2	I	901	ANP	PG-O1G	2.28	1.49	1.46
2	E	901	ANP	PG-O1G	2.26	1.49	1.46
2	B	901	ANP	PB-O3A	-2.23	1.56	1.59
2	D	901	ANP	PB-O1B	2.17	1.49	1.46

Continued on next page...

For Manuscript Review

Continued from previous page...

Mol	Chain	Res	Type	Atoms	Z	Observed(Å)	Ideal(Å)
2	B	901	ANP	PB-O1B	2.16	1.49	1.46
2	A	901	ANP	PB-O1B	2.15	1.49	1.46
2	J	901	ANP	PB-O1B	2.15	1.49	1.46
2	G	901	ANP	PB-O1B	2.12	1.49	1.46
2	H	901	ANP	PB-O1B	2.12	1.49	1.46
2	E	901	ANP	PB-O1B	2.12	1.49	1.46
2	I	901	ANP	PB-O1B	2.10	1.49	1.46
2	L	901	ANP	PB-O1B	2.10	1.49	1.46
2	K	901	ANP	PB-O1B	2.10	1.49	1.46
2	F	901	ANP	PB-O1B	2.09	1.49	1.46
2	C	901	ANP	PB-O1B	2.07	1.49	1.46

All (50) bond angle outliers are listed below:

Mol	Chain	Res	Type	Atoms	Z	Observed(^{o})	Ideal(^{o})
2	F	901	ANP	PB-O3A-PA	-3.97	118.62	132.62
2	L	901	ANP	PB-O3A-PA	-3.94	118.74	132.62
2	H	901	ANP	PB-O3A-PA	-3.89	118.93	132.62
2	C	901	ANP	PB-O3A-PA	-3.79	119.28	132.62
2	J	901	ANP	PB-O3A-PA	-3.78	119.29	132.62
2	I	901	ANP	PB-O3A-PA	-3.77	119.34	132.62
2	G	901	ANP	PB-O3A-PA	-3.74	119.45	132.62
2	A	901	ANP	PB-O3A-PA	-3.73	119.48	132.62
2	E	901	ANP	PB-O3A-PA	-3.70	119.58	132.62
2	K	901	ANP	PB-O3A-PA	-3.63	119.83	132.62
2	B	901	ANP	PB-O3A-PA	-3.49	120.33	132.62
2	D	901	ANP	PB-O3A-PA	-3.42	120.57	132.62
2	B	901	ANP	C5-C6-N6	2.29	123.83	120.35
2	I	901	ANP	C5-C6-N6	2.29	123.83	120.35
2	K	901	ANP	C5-C6-N6	2.28	123.82	120.35
2	A	901	ANP	C5-C6-N6	2.28	123.81	120.35
2	C	901	ANP	C5-C6-N6	2.27	123.81	120.35
2	D	901	ANP	C5-C6-N6	2.27	123.80	120.35
2	F	901	ANP	C5-C6-N6	2.26	123.79	120.35
2	E	901	ANP	C5-C6-N6	2.25	123.78	120.35
2	G	901	ANP	C5-C6-N6	2.23	123.75	120.35
2	J	901	ANP	C5-C6-N6	2.23	123.75	120.35
2	H	901	ANP	C5-C6-N6	2.21	123.72	120.35
2	L	901	ANP	C5-C6-N6	2.21	123.71	120.35
3	F	902	TPP	C5-C4-N3	2.18	111.94	107.57
3	L	902	TPP	C5-C4-N3	2.18	111.93	107.57
3	K	902	TPP	C5-C4-N3	2.17	111.91	107.57

Continued on next page...

For Manuscript Review

Continued from previous page...

Mol	Chain	Res	Type	Atoms	Z	Observed(°)	Ideal(°)
3	C	902	TPP	C5-C4-N3	2.17	111.91	107.57
3	J	902	TPP	C5-C4-N3	2.17	111.90	107.57
3	D	902	TPP	C5-C4-N3	2.16	111.90	107.57
3	E	902	TPP	C5-C4-N3	2.16	111.90	107.57
3	G	902	TPP	C5-C4-N3	2.16	111.90	107.57
3	H	902	TPP	C5-C4-N3	2.16	111.90	107.57
3	B	902	TPP	C5-C4-N3	2.15	111.88	107.57
3	A	902	TPP	C5-C4-N3	2.15	111.87	107.57
3	I	902	TPP	C5-C4-N3	2.15	111.87	107.57
3	G	902	TPP	PA-O3A-PB	2.07	139.92	132.83
3	I	902	TPP	PA-O3A-PB	2.06	139.91	132.83
3	E	902	TPP	PA-O3A-PB	2.06	139.90	132.83
3	J	902	TPP	PA-O3A-PB	2.06	139.89	132.83
3	F	902	TPP	PA-O3A-PB	2.05	139.87	132.83
3	L	902	TPP	PA-O3A-PB	2.05	139.87	132.83
3	C	902	TPP	PA-O3A-PB	2.05	139.86	132.83
3	A	902	TPP	PA-O3A-PB	2.05	139.85	132.83
3	B	902	TPP	PA-O3A-PB	2.04	139.81	132.83
3	K	902	TPP	PA-O3A-PB	2.03	139.81	132.83
3	H	902	TPP	PA-O3A-PB	2.03	139.80	132.83
3	D	902	TPP	PA-O3A-PB	2.03	139.79	132.83
2	D	901	ANP	O2G-PG-O1G	-2.01	108.41	113.45
2	J	901	ANP	O2G-PG-O1G	-2.00	108.42	113.45

There are no chirality outliers.

All (121) torsion outliers are listed below:

Mol	Chain	Res	Type	Atoms
2	A	901	ANP	PB-N3B-PG-O1G
2	A	901	ANP	O4'-C4'-C5'-O5'
2	B	901	ANP	PB-N3B-PG-O1G
2	B	901	ANP	O4'-C4'-C5'-O5'
2	C	901	ANP	PB-N3B-PG-O1G
2	C	901	ANP	O4'-C4'-C5'-O5'
2	D	901	ANP	PB-N3B-PG-O1G
2	D	901	ANP	O4'-C4'-C5'-O5'
2	E	901	ANP	PB-N3B-PG-O1G
2	E	901	ANP	O4'-C4'-C5'-O5'
2	F	901	ANP	PB-N3B-PG-O1G
2	F	901	ANP	O4'-C4'-C5'-O5'
2	G	901	ANP	PB-N3B-PG-O1G
2	G	901	ANP	O4'-C4'-C5'-O5'

Continued on next page...

For Manuscript Review

Continued from previous page...

Mol	Chain	Res	Type	Atoms
2	H	901	ANP	PB-N3B-PG-O1G
2	H	901	ANP	O4'-C4'-C5'-O5'
2	I	901	ANP	PB-N3B-PG-O1G
2	I	901	ANP	O4'-C4'-C5'-O5'
2	J	901	ANP	PB-N3B-PG-O1G
2	J	901	ANP	O4'-C4'-C5'-O5'
2	K	901	ANP	PB-N3B-PG-O1G
2	K	901	ANP	O4'-C4'-C5'-O5'
2	L	901	ANP	PB-N3B-PG-O1G
2	L	901	ANP	O4'-C4'-C5'-O5'
3	A	902	TPP	C4'-C5'-C7'-N3
3	A	902	TPP	C5-C6-C7-O7
3	A	902	TPP	PA-O3A-PB-O3B
3	B	902	TPP	C4'-C5'-C7'-N3
3	B	902	TPP	C5-C6-C7-O7
3	C	902	TPP	C4'-C5'-C7'-N3
3	C	902	TPP	C5-C6-C7-O7
3	C	902	TPP	PA-O3A-PB-O2B
3	D	902	TPP	C4'-C5'-C7'-N3
3	D	902	TPP	C5-C6-C7-O7
3	E	902	TPP	C4'-C5'-C7'-N3
3	E	902	TPP	C5-C6-C7-O7
3	E	902	TPP	PA-O3A-PB-O2B
3	F	902	TPP	C4'-C5'-C7'-N3
3	F	902	TPP	C5-C6-C7-O7
3	F	902	TPP	PA-O3A-PB-O3B
3	G	902	TPP	C4'-C5'-C7'-N3
3	G	902	TPP	C5-C6-C7-O7
3	G	902	TPP	PA-O3A-PB-O2B
3	G	902	TPP	PA-O3A-PB-O3B
3	H	902	TPP	C4'-C5'-C7'-N3
3	H	902	TPP	C5-C6-C7-O7
3	I	902	TPP	C4'-C5'-C7'-N3
3	I	902	TPP	C5-C6-C7-O7
3	I	902	TPP	PA-O3A-PB-O2B
3	J	902	TPP	C4'-C5'-C7'-N3
3	J	902	TPP	C5-C6-C7-O7
3	J	902	TPP	PA-O3A-PB-O3B
3	K	902	TPP	C4'-C5'-C7'-N3
3	K	902	TPP	C5-C6-C7-O7
3	K	902	TPP	PA-O3A-PB-O2B
3	L	902	TPP	C4'-C5'-C7'-N3

Continued on next page...

For Manuscript Review

Continued from previous page...

Mol	Chain	Res	Type	Atoms
3	L	902	TPP	C5-C6-C7-O7
3	L	902	TPP	PA-O3A-PB-O3B
2	A	901	ANP	C3'-C4'-C5'-O5'
2	B	901	ANP	C3'-C4'-C5'-O5'
2	C	901	ANP	C3'-C4'-C5'-O5'
2	D	901	ANP	C3'-C4'-C5'-O5'
2	E	901	ANP	C3'-C4'-C5'-O5'
2	F	901	ANP	C3'-C4'-C5'-O5'
2	G	901	ANP	C3'-C4'-C5'-O5'
2	H	901	ANP	C3'-C4'-C5'-O5'
2	I	901	ANP	C3'-C4'-C5'-O5'
2	J	901	ANP	C3'-C4'-C5'-O5'
2	K	901	ANP	C3'-C4'-C5'-O5'
2	L	901	ANP	C3'-C4'-C5'-O5'
2	A	901	ANP	C4'-C5'-O5'-PA
2	G	901	ANP	C4'-C5'-O5'-PA
2	I	901	ANP	C4'-C5'-O5'-PA
2	D	901	ANP	C4'-C5'-O5'-PA
2	F	901	ANP	C4'-C5'-O5'-PA
2	H	901	ANP	C4'-C5'-O5'-PA
3	A	902	TPP	PA-O3A-PB-O2B
3	E	902	TPP	PA-O3A-PB-O3B
3	I	902	TPP	PA-O3A-PB-O3B
3	K	902	TPP	PA-O3A-PB-O3B
3	B	902	TPP	PB-O3A-PA-O1A
3	C	902	TPP	PB-O3A-PA-O2A
3	D	902	TPP	PB-O3A-PA-O1A
3	H	902	TPP	PB-O3A-PA-O1A
2	B	901	ANP	C4'-C5'-O5'-PA
2	C	901	ANP	C4'-C5'-O5'-PA
2	J	901	ANP	C4'-C5'-O5'-PA
2	K	901	ANP	C4'-C5'-O5'-PA
2	E	901	ANP	C4'-C5'-O5'-PA
2	L	901	ANP	C4'-C5'-O5'-PA
3	E	902	TPP	PB-O3A-PA-O2A
3	G	902	TPP	PB-O3A-PA-O2A
3	I	902	TPP	PB-O3A-PA-O2A
3	K	902	TPP	PB-O3A-PA-O2A
3	C	902	TPP	PA-O3A-PB-O1B
3	B	902	TPP	PA-O3A-PB-O3B
3	C	902	TPP	PA-O3A-PB-O3B
3	D	902	TPP	PA-O3A-PB-O3B

Continued on next page...

For Manuscript Review

Continued from previous page...

Mol	Chain	Res	Type	Atoms
3	H	902	TPP	PA-O3A-PB-O3B
3	A	902	TPP	PB-O3A-PA-O1A
3	A	902	TPP	PB-O3A-PA-O2A
3	C	902	TPP	PB-O3A-PA-O1A
3	F	902	TPP	PB-O3A-PA-O2A
3	J	902	TPP	PB-O3A-PA-O2A
3	L	902	TPP	PB-O3A-PA-O2A
3	A	902	TPP	C6'-C5'-C7'-N3
3	A	902	TPP	C7-O7-PA-O1A
3	B	902	TPP	C6'-C5'-C7'-N3
3	C	902	TPP	C6'-C5'-C7'-N3
3	D	902	TPP	C6'-C5'-C7'-N3
3	E	902	TPP	C6'-C5'-C7'-N3
3	F	902	TPP	C6'-C5'-C7'-N3
3	G	902	TPP	C6'-C5'-C7'-N3
3	G	902	TPP	C7-O7-PA-O1A
3	H	902	TPP	C6'-C5'-C7'-N3
3	I	902	TPP	C6'-C5'-C7'-N3
3	I	902	TPP	C7-O7-PA-O1A
3	J	902	TPP	C6'-C5'-C7'-N3
3	K	902	TPP	C6'-C5'-C7'-N3
3	L	902	TPP	C6'-C5'-C7'-N3
3	I	902	TPP	PA-O3A-PB-O1B

There are no ring outliers.

23 monomers are involved in 82 short contacts:

Mol	Chain	Res	Type	Clashes	Symm-Clashes
3	J	902	TPP	4	0
2	G	901	ANP	3	0
3	I	902	TPP	5	0
2	L	901	ANP	3	0
2	F	901	ANP	2	0
3	L	902	TPP	5	0
3	D	902	TPP	4	0
3	G	902	TPP	5	0
3	E	902	TPP	5	0
2	B	901	ANP	1	0
2	I	901	ANP	2	0
2	H	901	ANP	3	0
3	A	902	TPP	6	0
3	F	902	TPP	5	0

Continued on next page...

For Manuscript Review

Continued from previous page...

Mol	Chain	Res	Type	Clashes	Symm-Clashes
2	A	901	ANP	3	0
2	D	901	ANP	1	0
2	J	901	ANP	3	0
2	E	901	ANP	2	0
3	H	902	TPP	4	0
2	C	901	ANP	2	0
3	B	902	TPP	4	0
3	C	902	TPP	5	0
3	K	902	TPP	5	0

The following is a two-dimensional graphical depiction of Mogul quality analysis of bond lengths, bond angles, torsion angles, and ring geometry for all instances of the Ligand of Interest. In addition, ligands with molecular weight > 250 and outliers as shown on the validation Tables will also be included. For torsion angles, if less then 5% of the Mogul distribution of torsion angles is within 10 degrees of the torsion angle in question, then that torsion angle is considered an outlier. Any bond that is central to one or more torsion angles identified as an outlier by Mogul will be highlighted in the graph. For rings, the root-mean-square deviation (RMSD) between the ring in question and similar rings identified by Mogul is calculated over all ring torsion angles. If the average RMSD is greater than 60 degrees and the minimal RMSD between the ring in question and any Mogul-identified rings is also greater than 60 degrees, then that ring is considered an outlier. The outliers are highlighted in purple. The color gray indicates Mogul did not find sufficient equivalents in the CSD to analyse the geometry.

Ligand TPP J 902

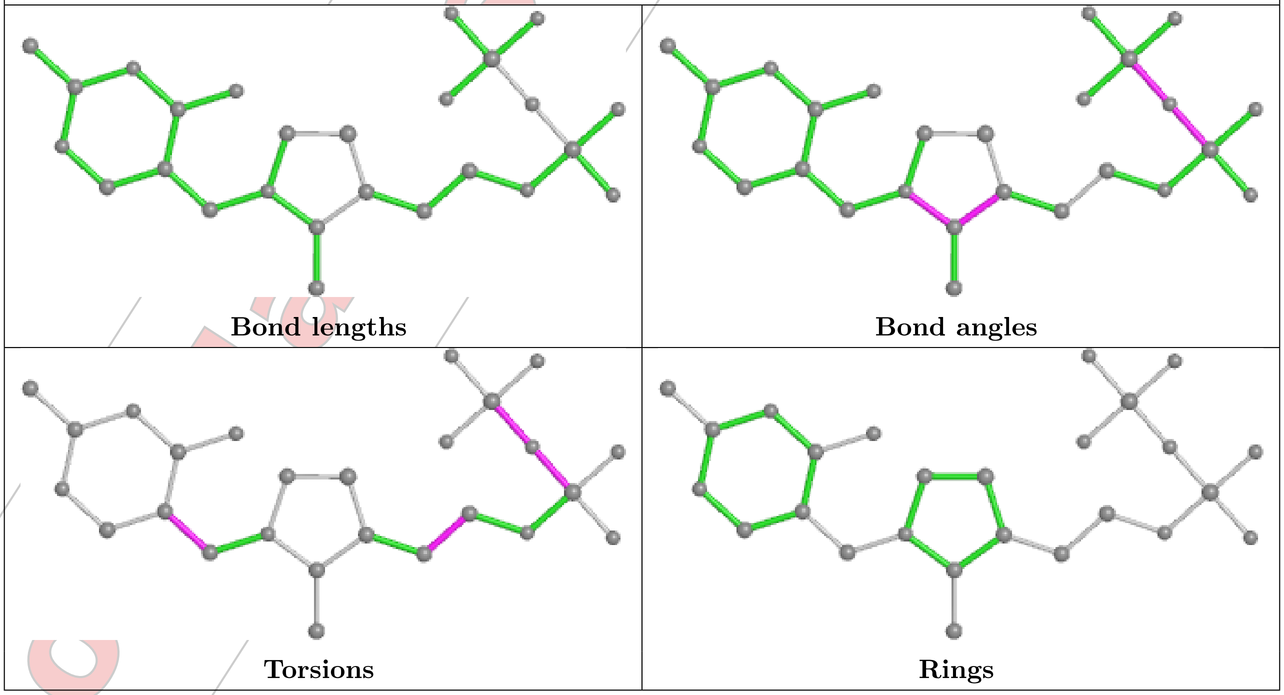

For Manuscript Review

Ligand ANP G 901

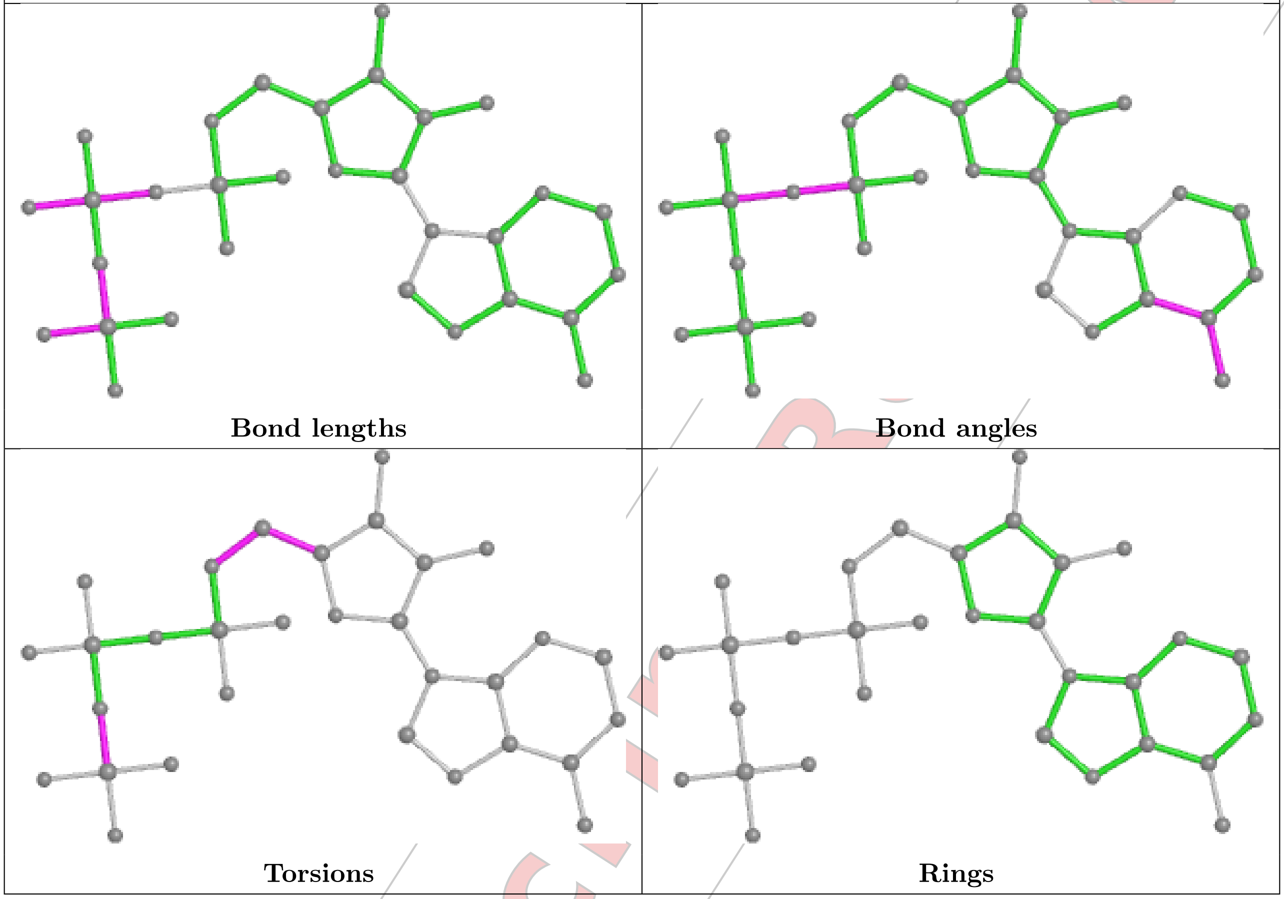

Ligand TPP I 902

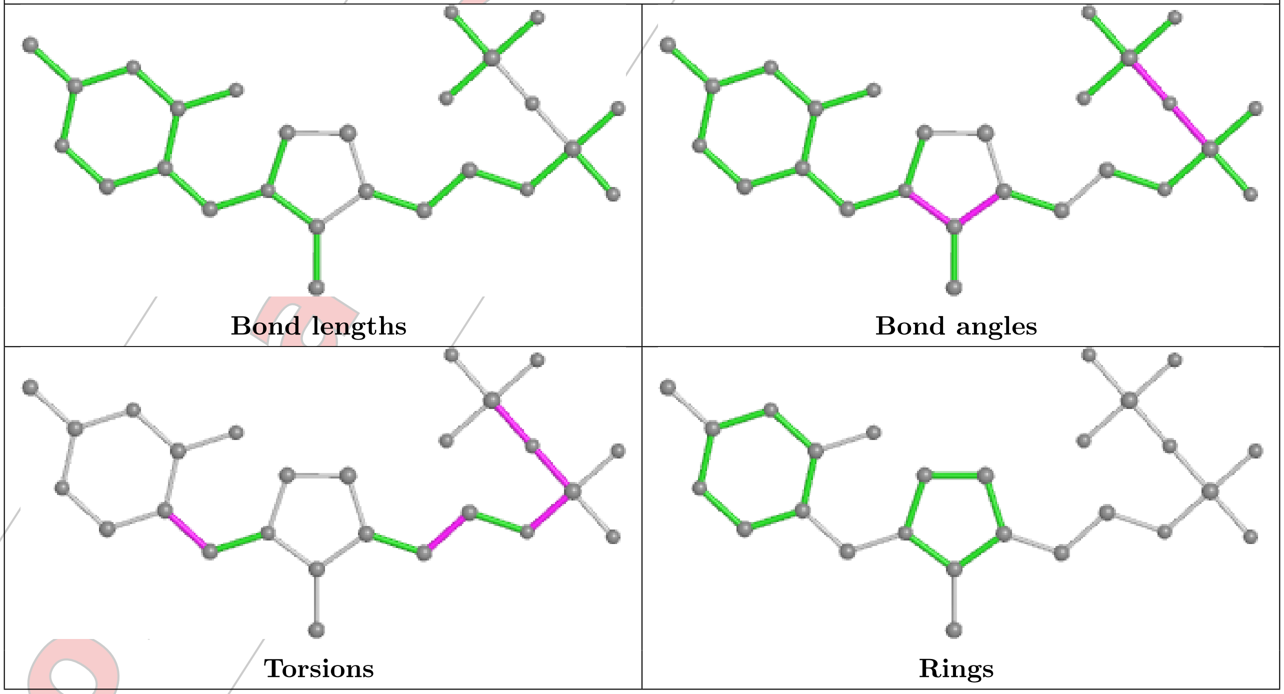

Ligand ANP L 901

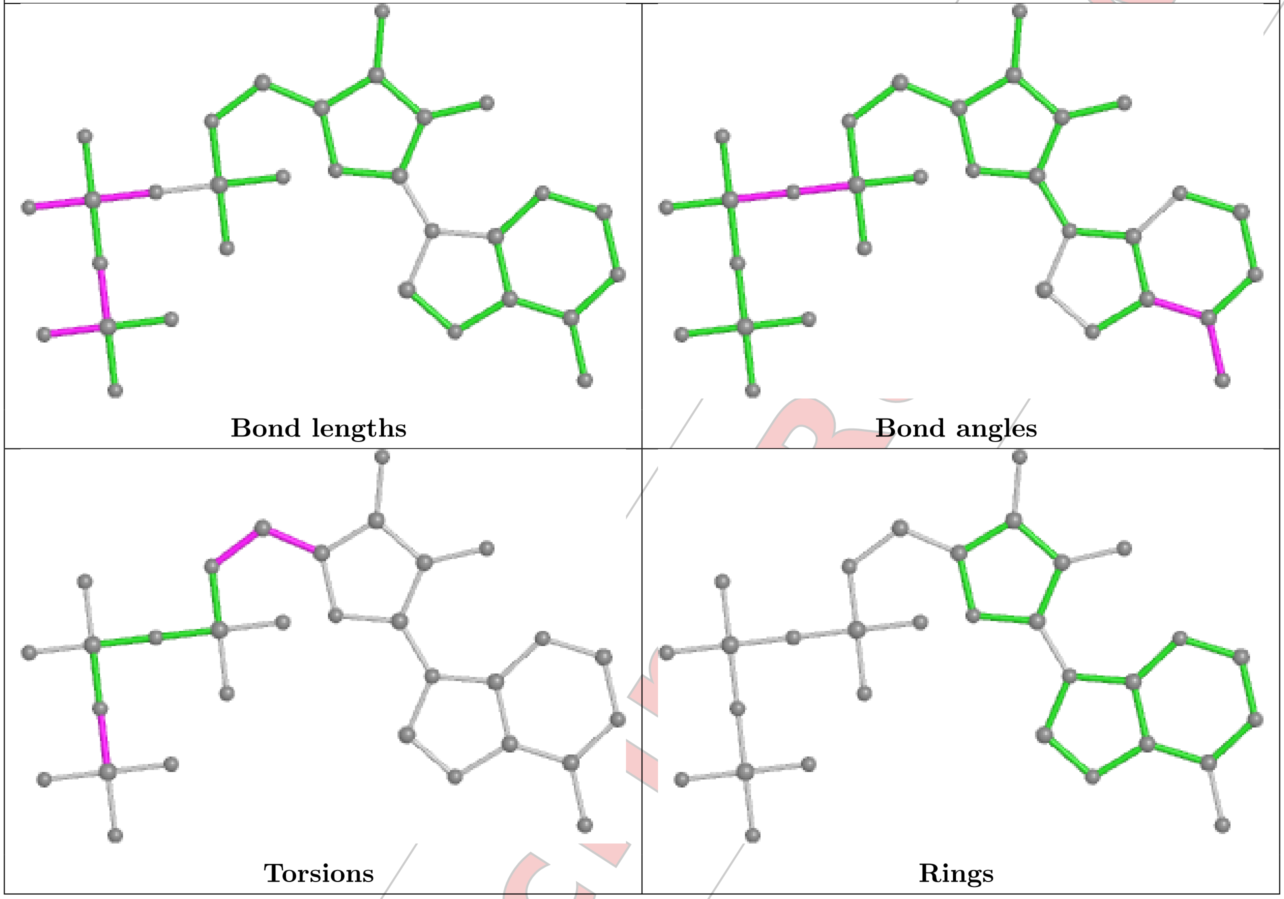

For Manuscript Review

Ligand ANP F 901

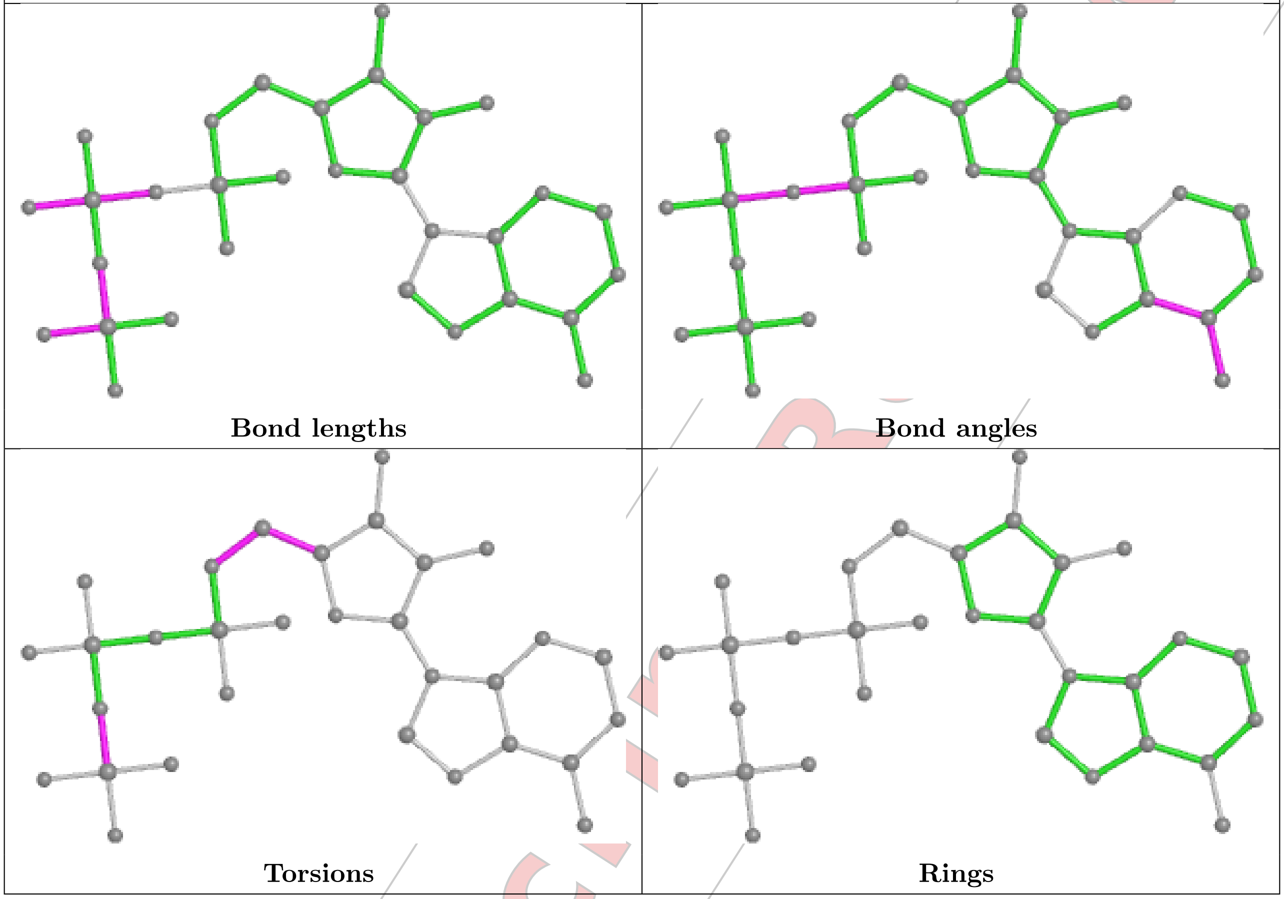

Ligand TPP L 902

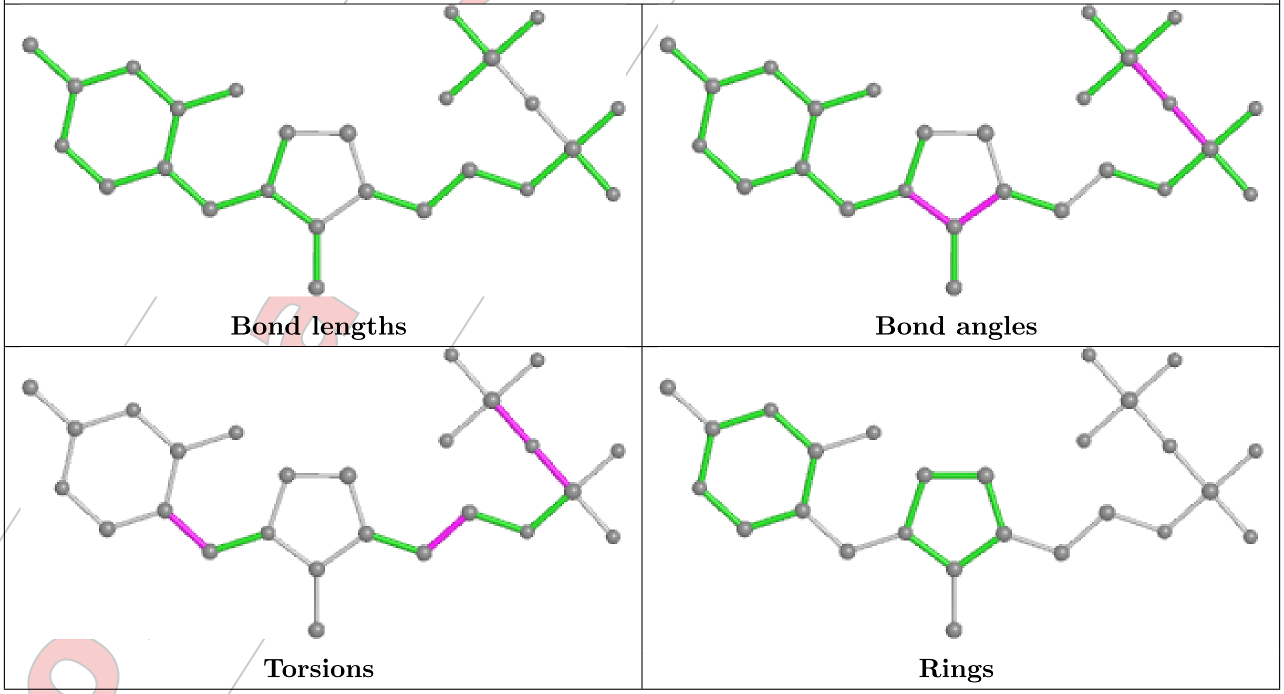

Ligand TPP D 902

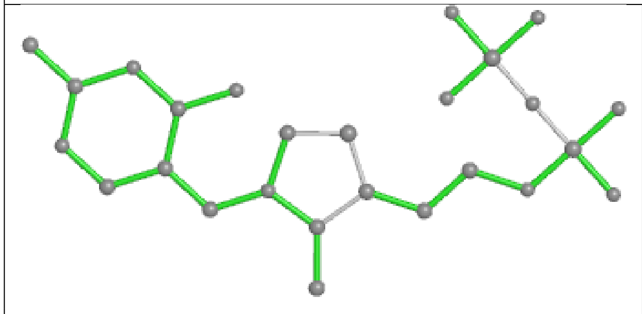

Bond lengths

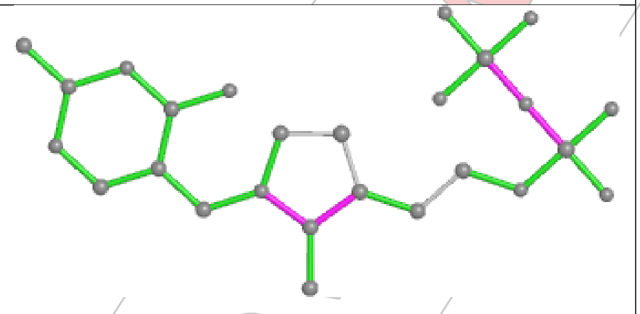

Bond angles

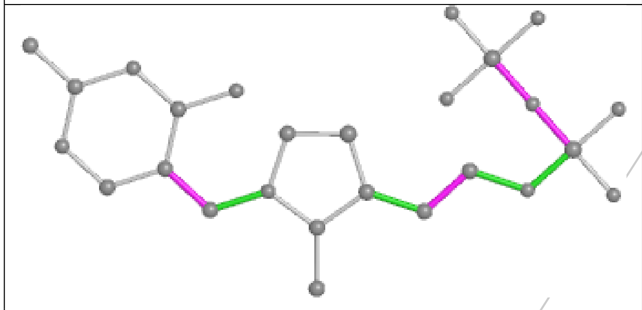

Torsions

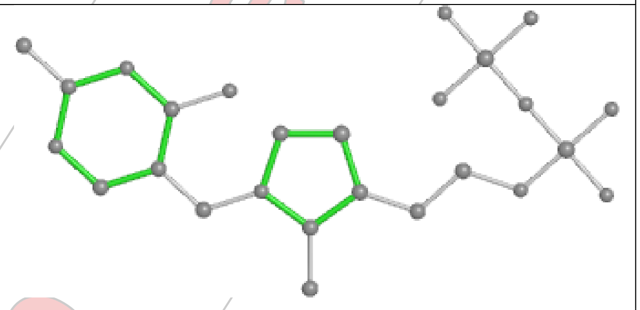

Rings

Ligand TPP G 902

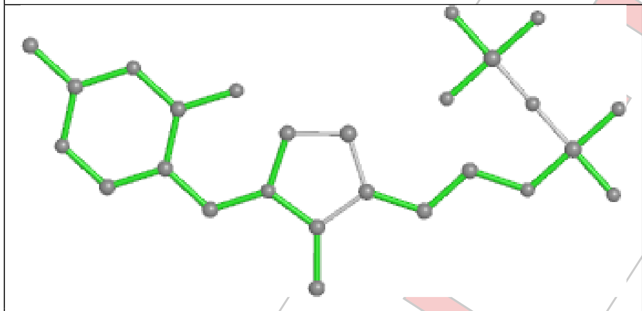

Bond lengths

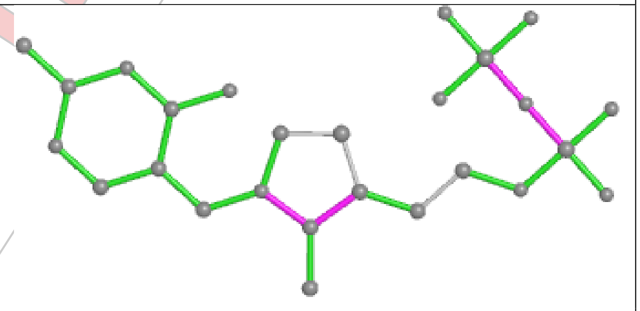

Bond angles

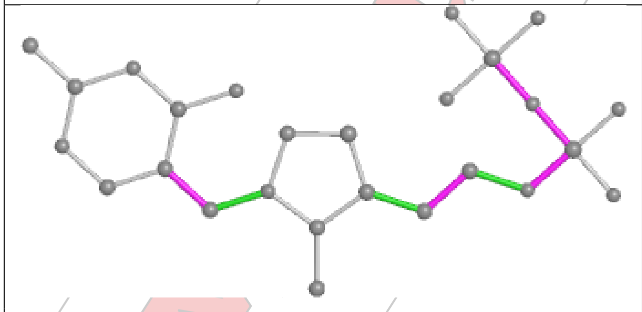

Torsions

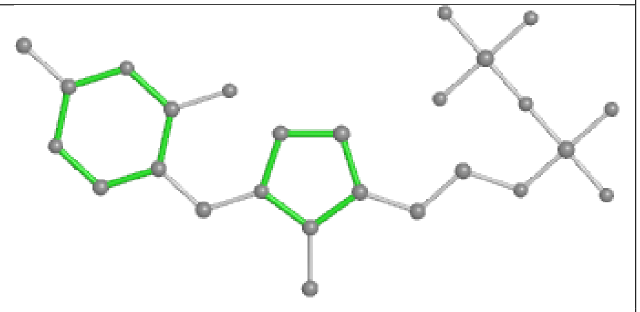

Rings

Ligand TPP E 902

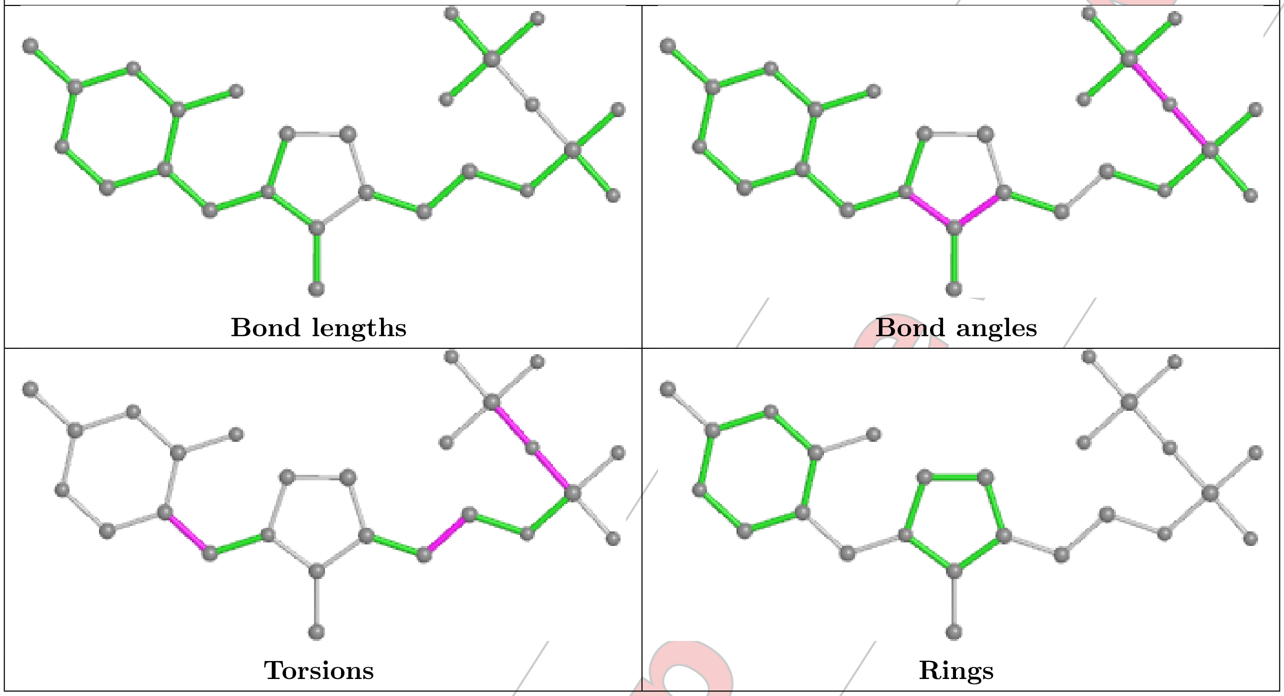

Ligand ANP B 901

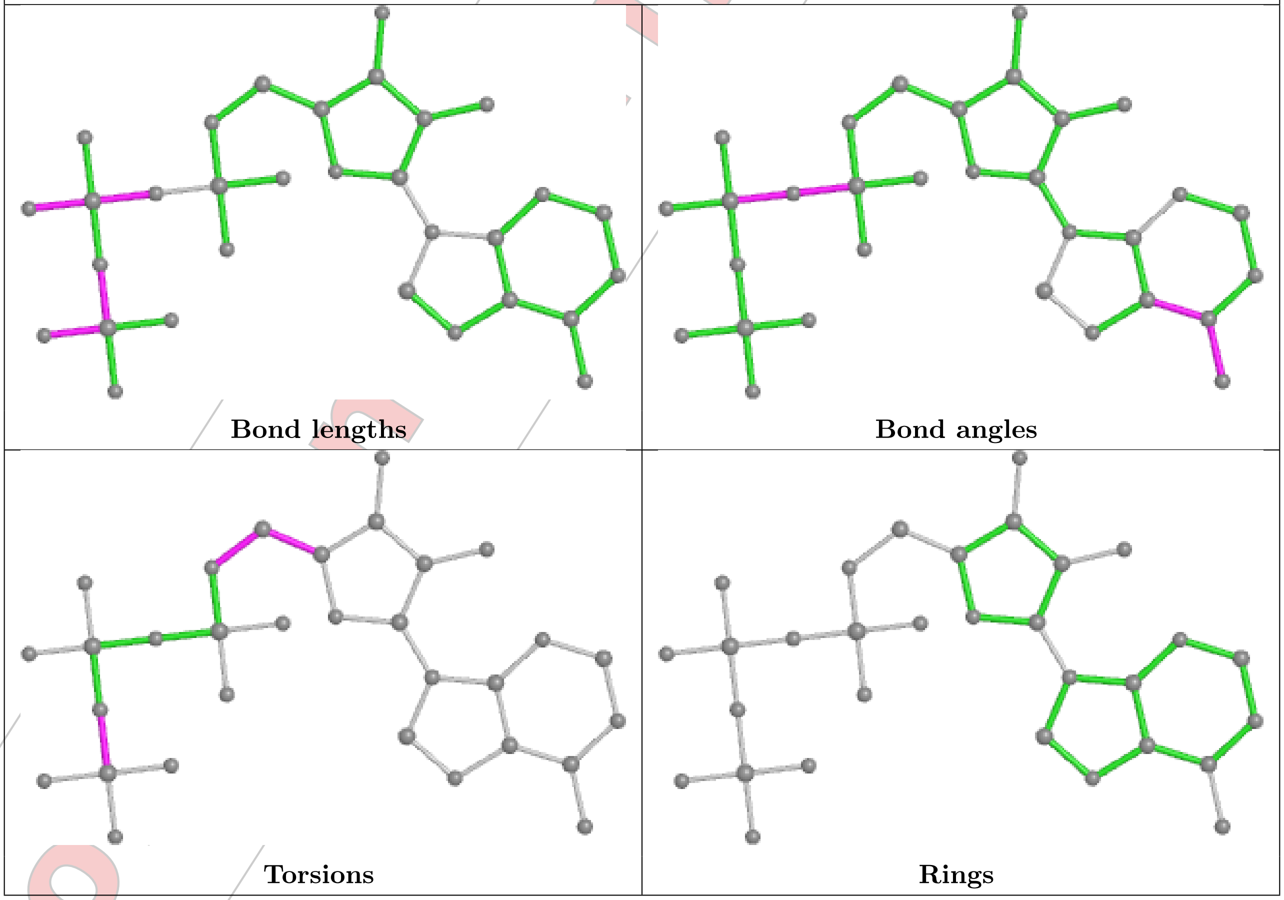

Ligand ANP I 901

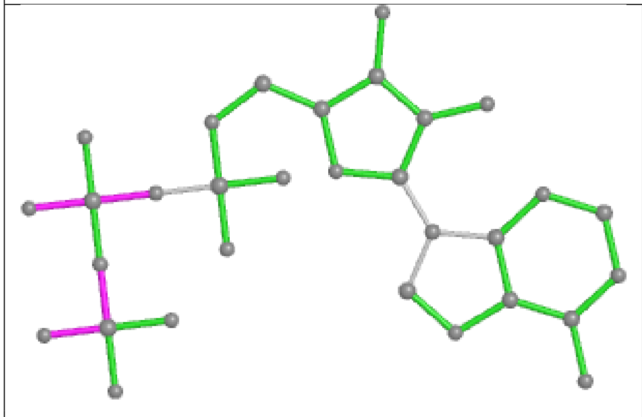

Bond lengths

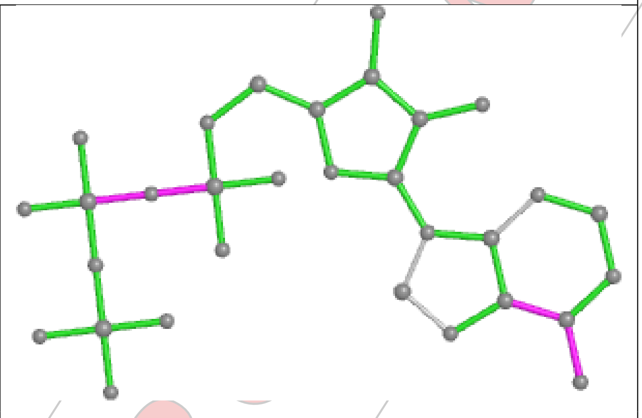

Bond angles

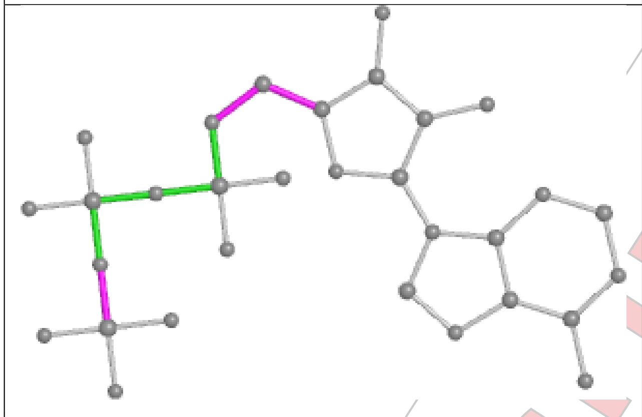

Torsions

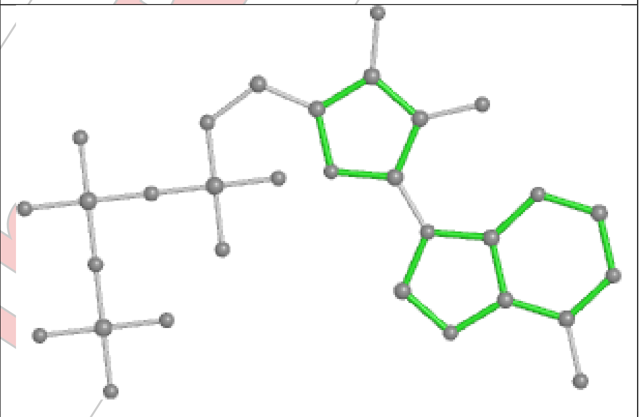

Rings

For Manuscript Review

Ligand ANP H 901

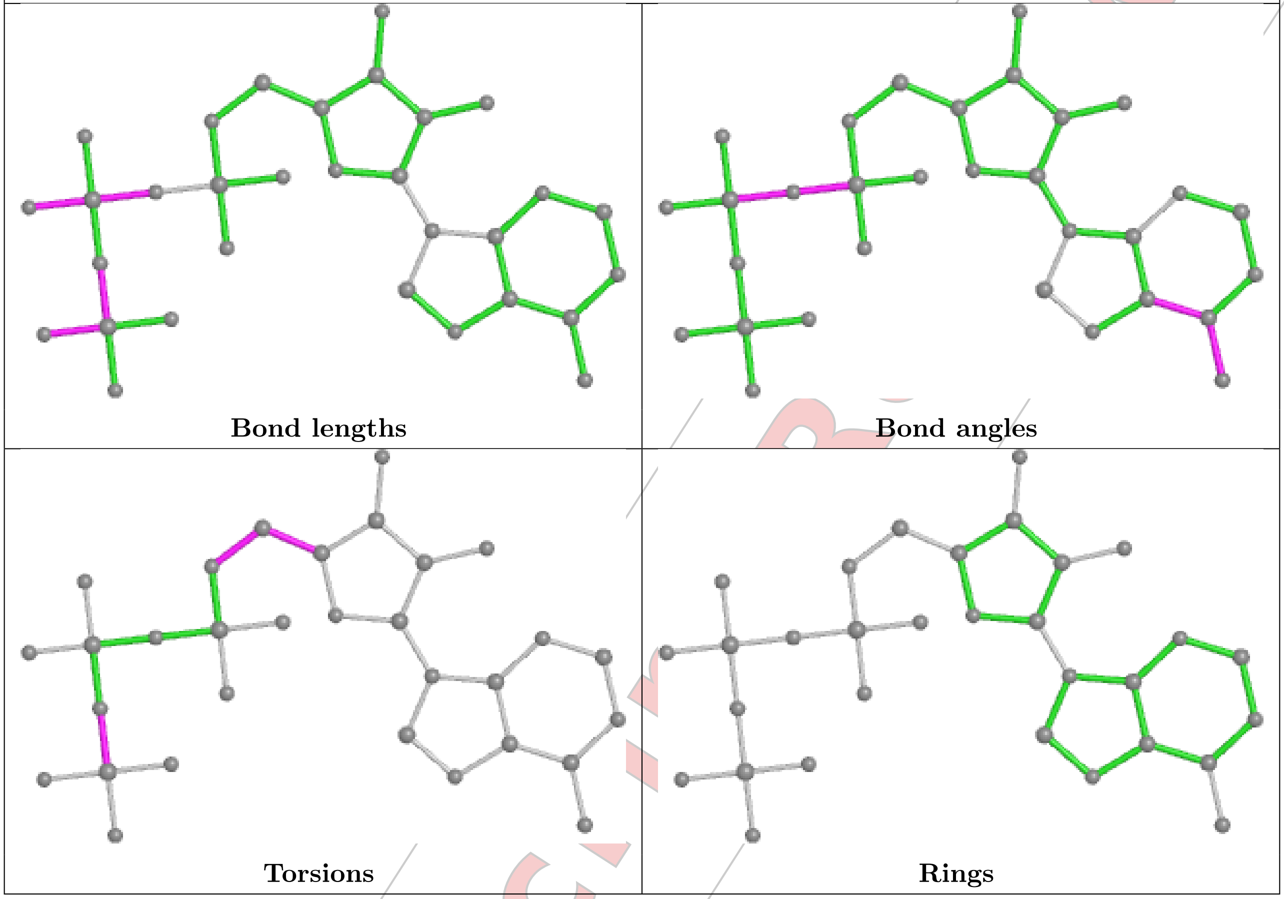

Ligand TPP A 902

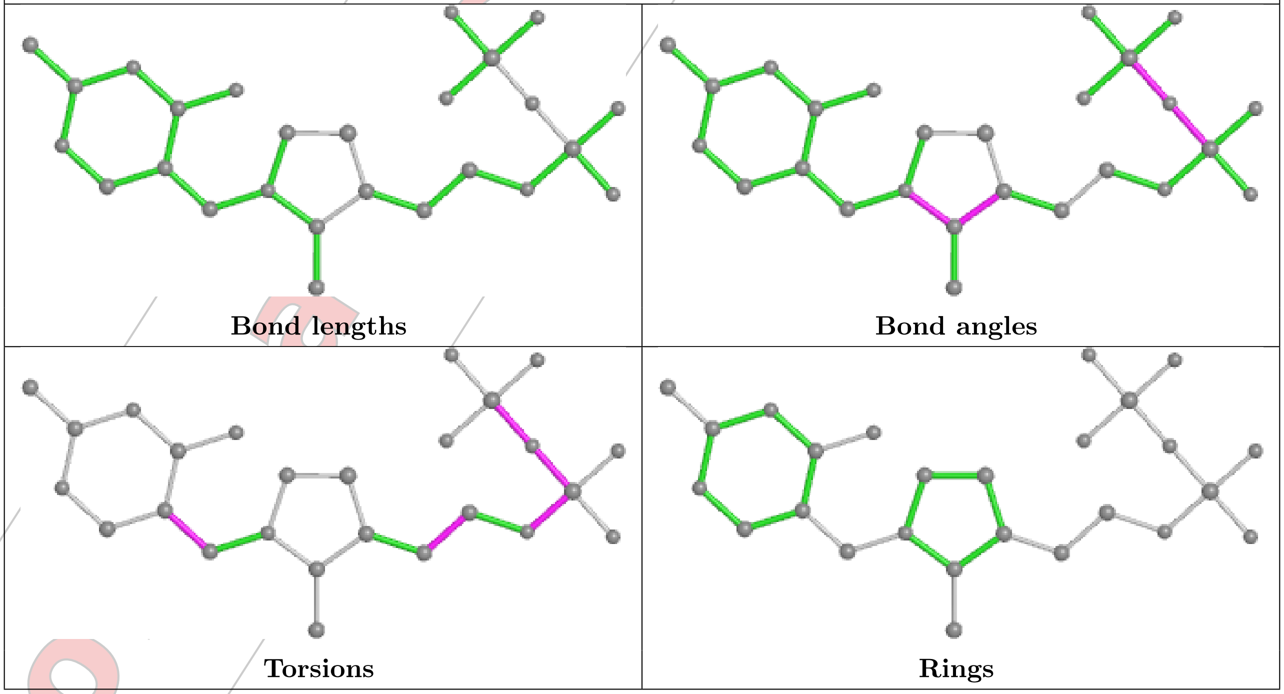

Ligand TPP F 902

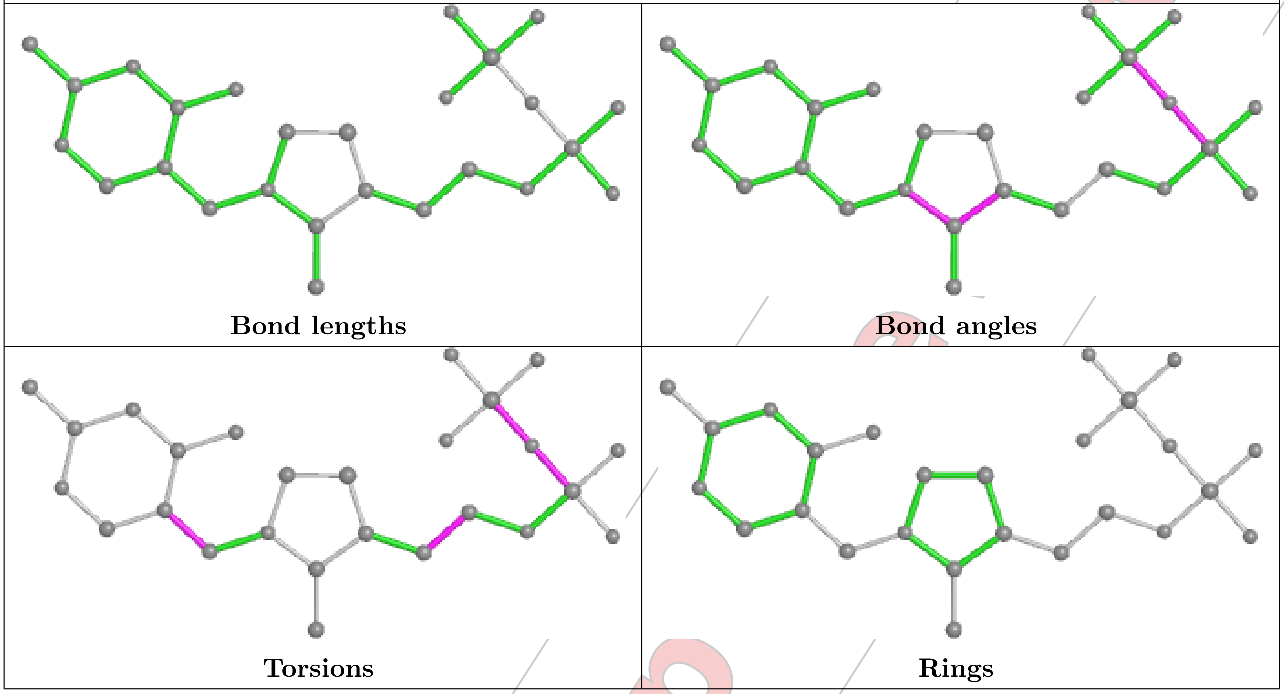

Ligand ANP A 901

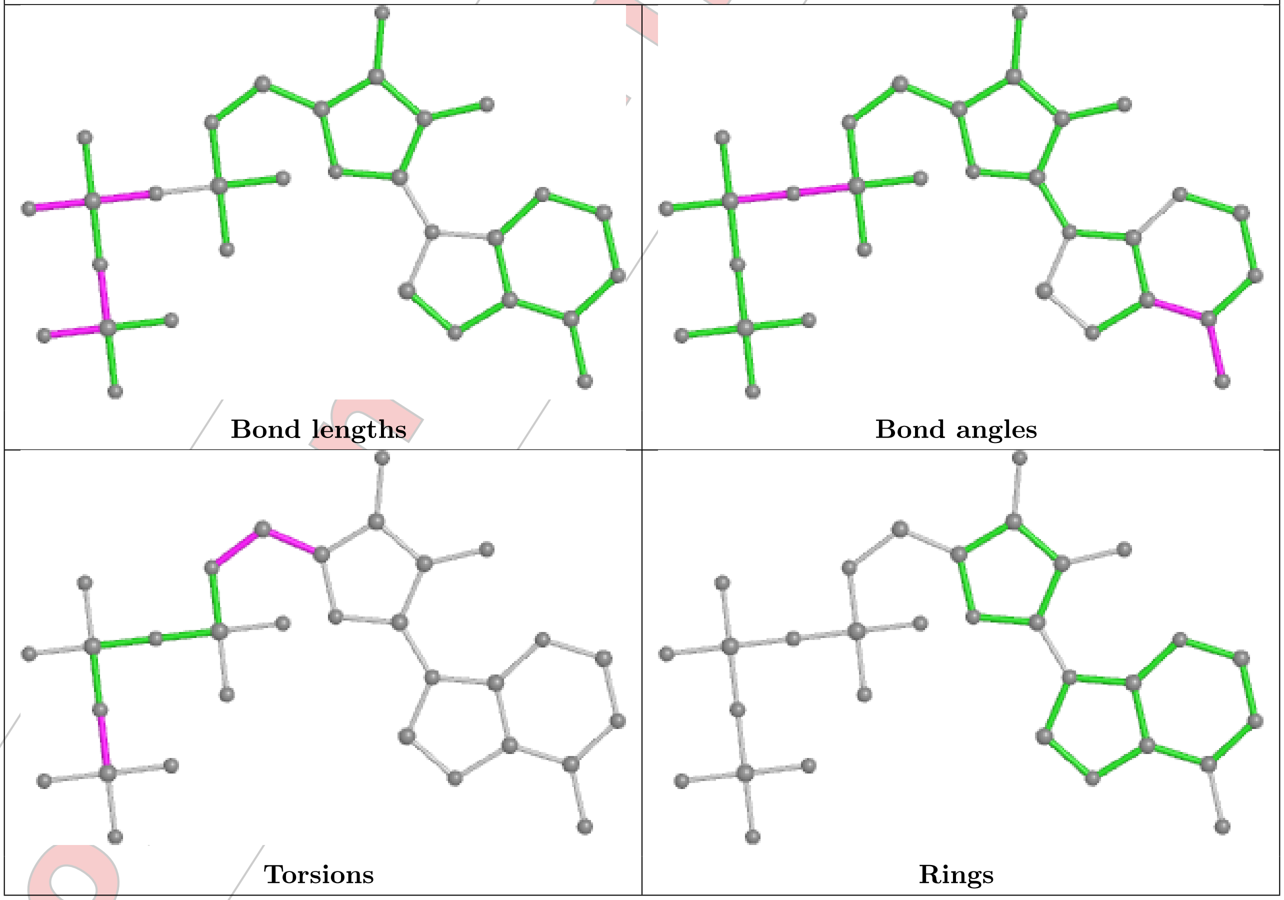

Ligand ANP D 901	
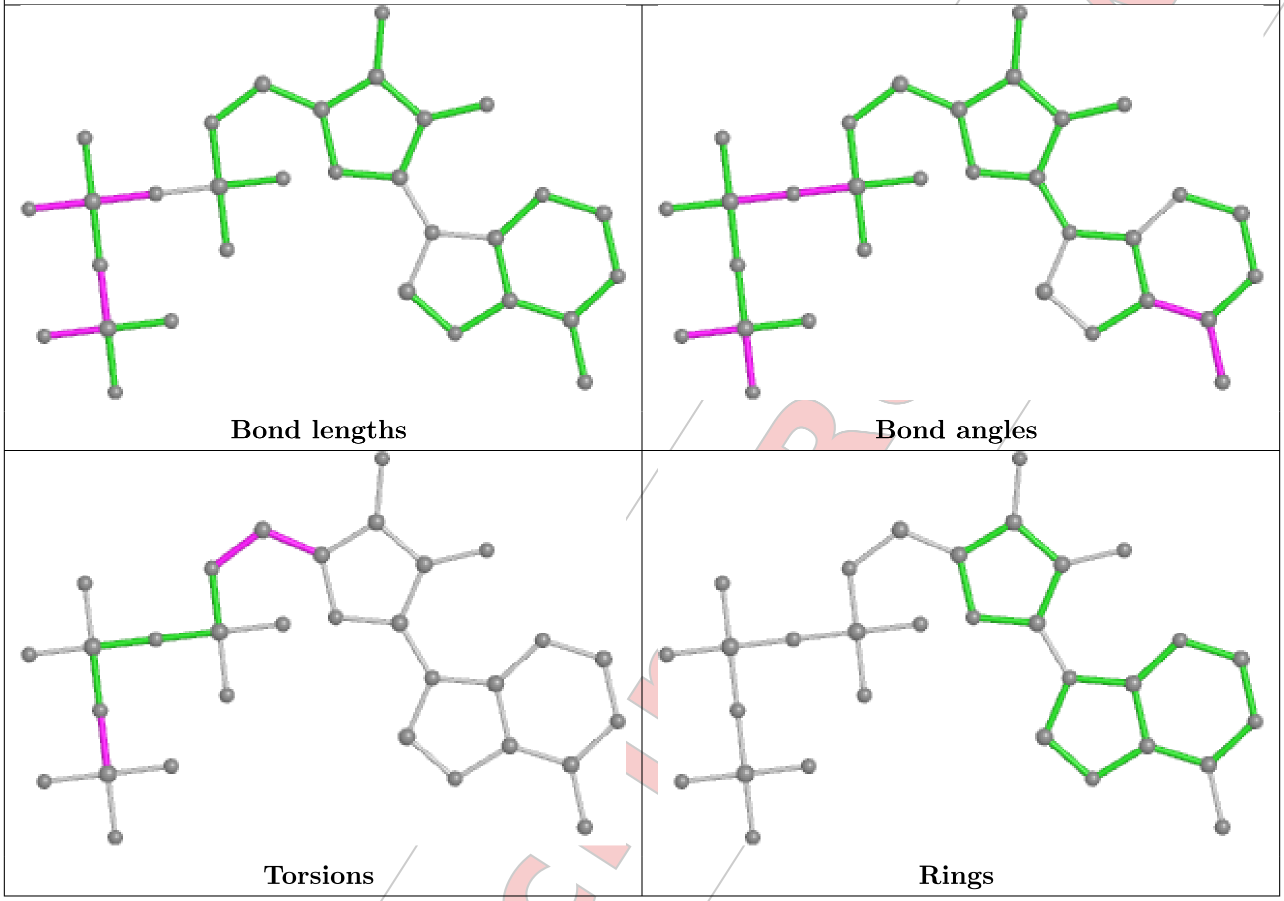 Bond lengths	Bond angles
Torsions	Rings

For Manuscript Review

Ligand ANP J 901

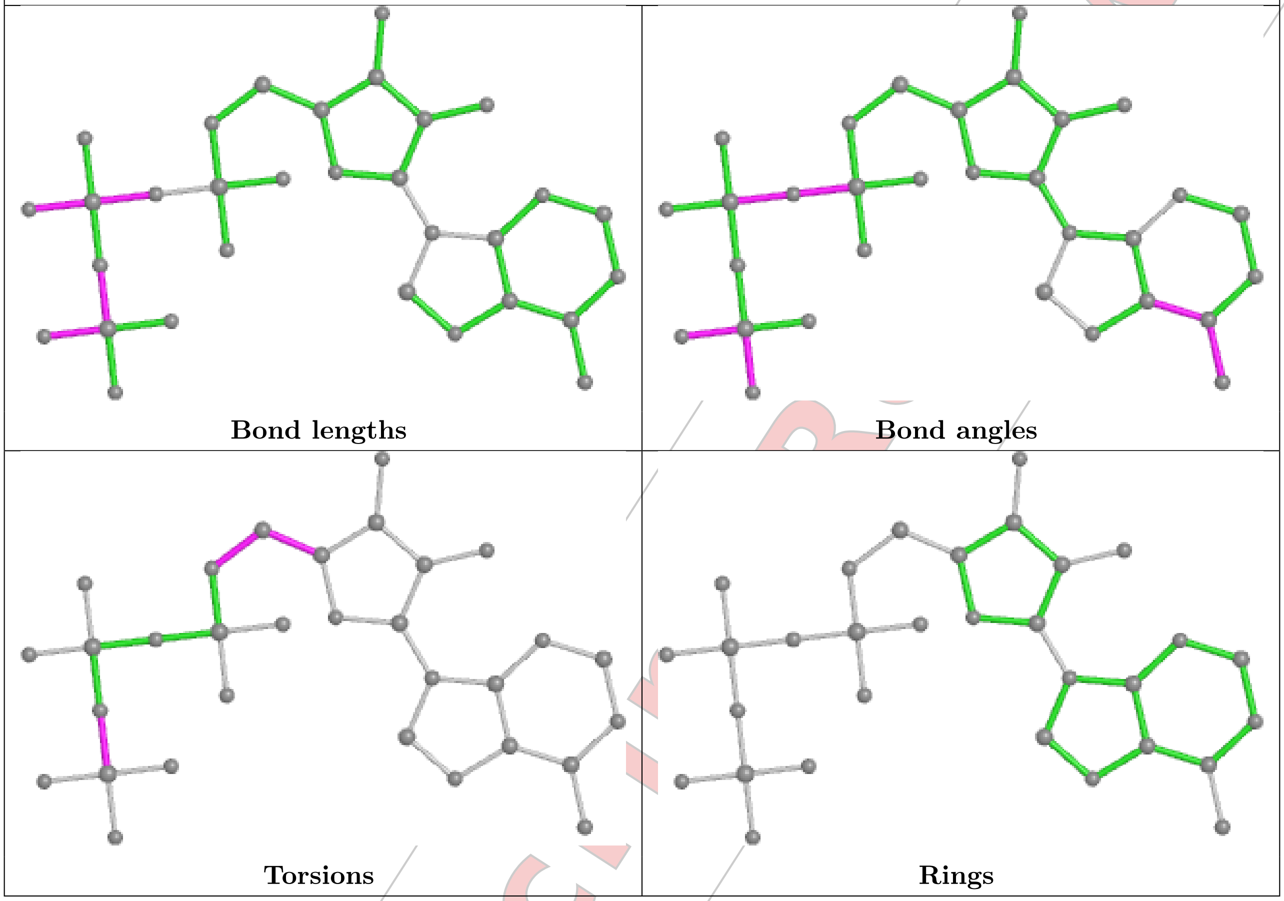

For Manuscript Review

Ligand ANP E 901

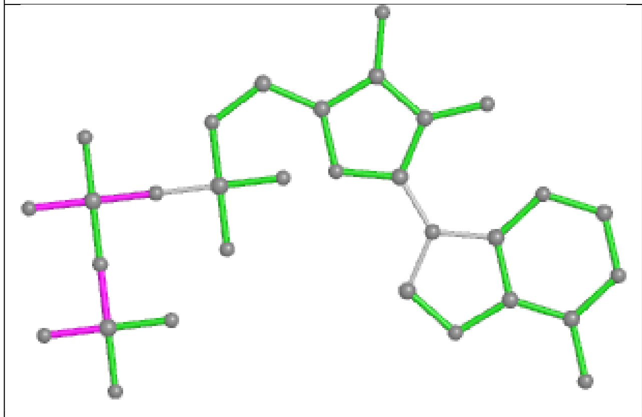

Bond lengths

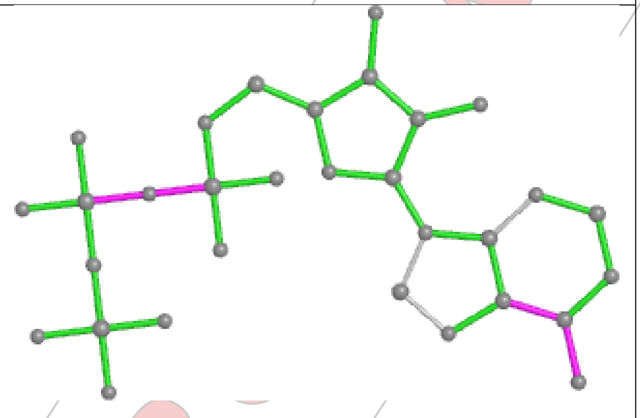

Bond angles

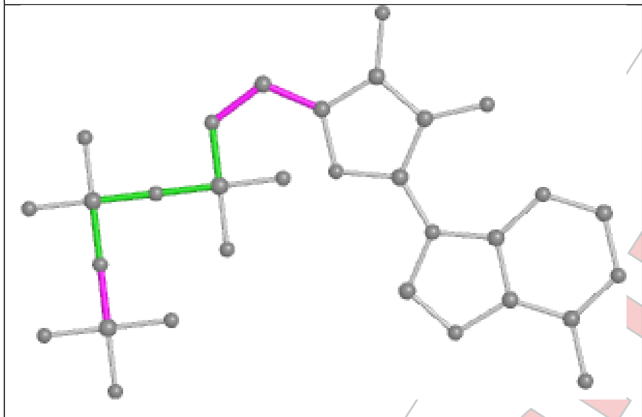

Torsions

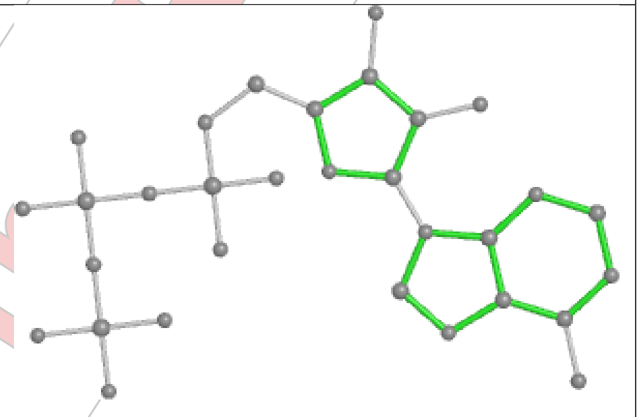

Rings

Ligand TPP H 902

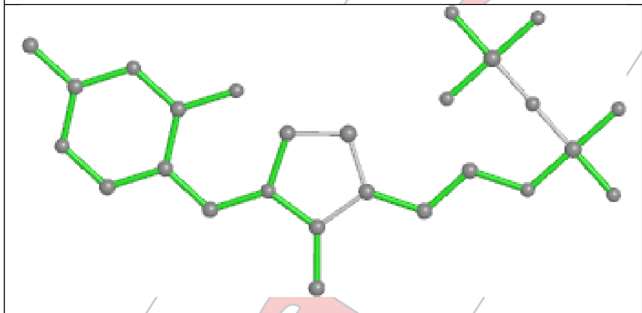

Bond lengths

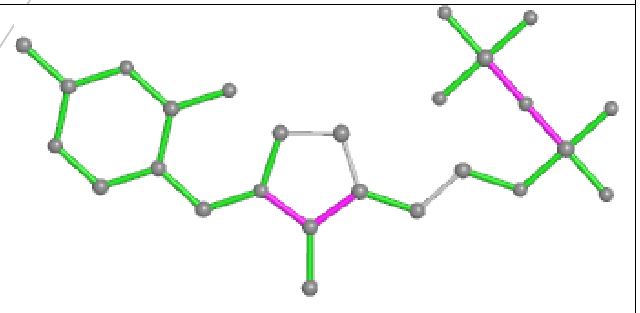

Bond angles

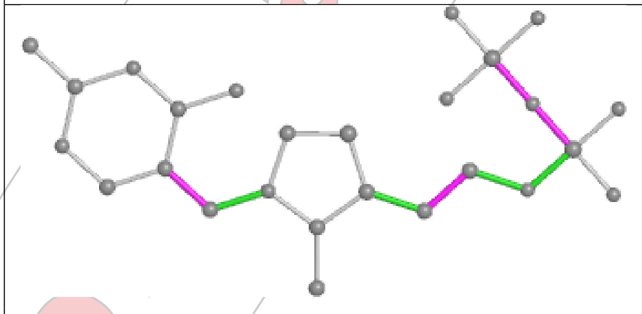

Torsions

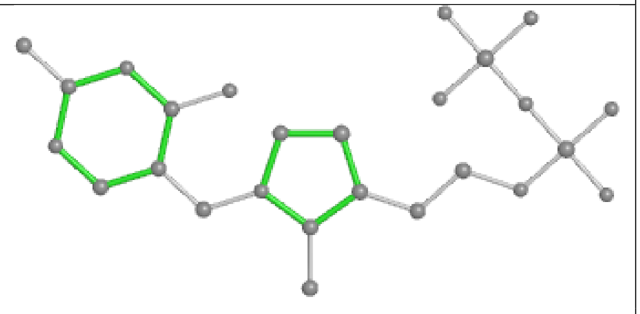

Rings

Ligand ANP C 901

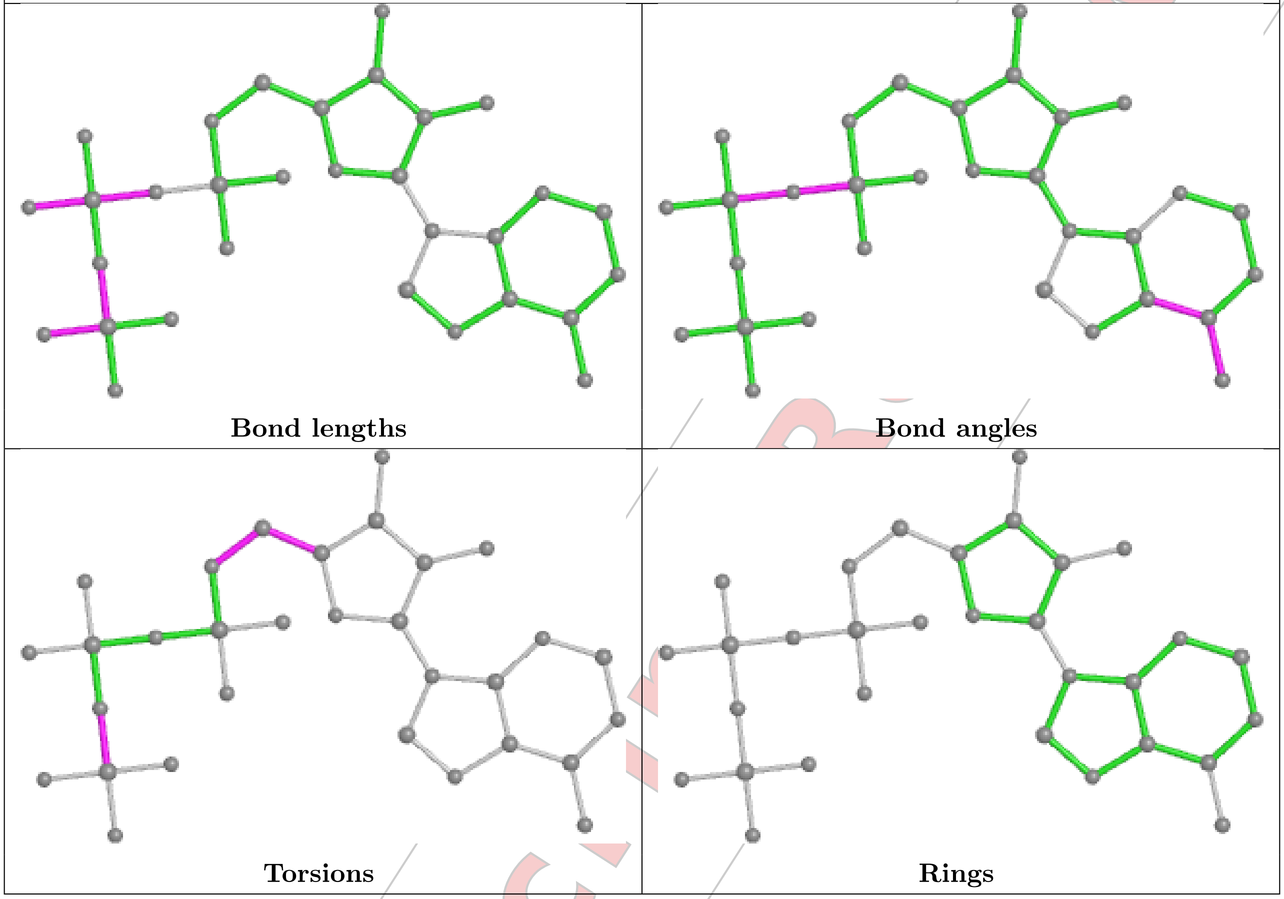

Ligand TPP B 902

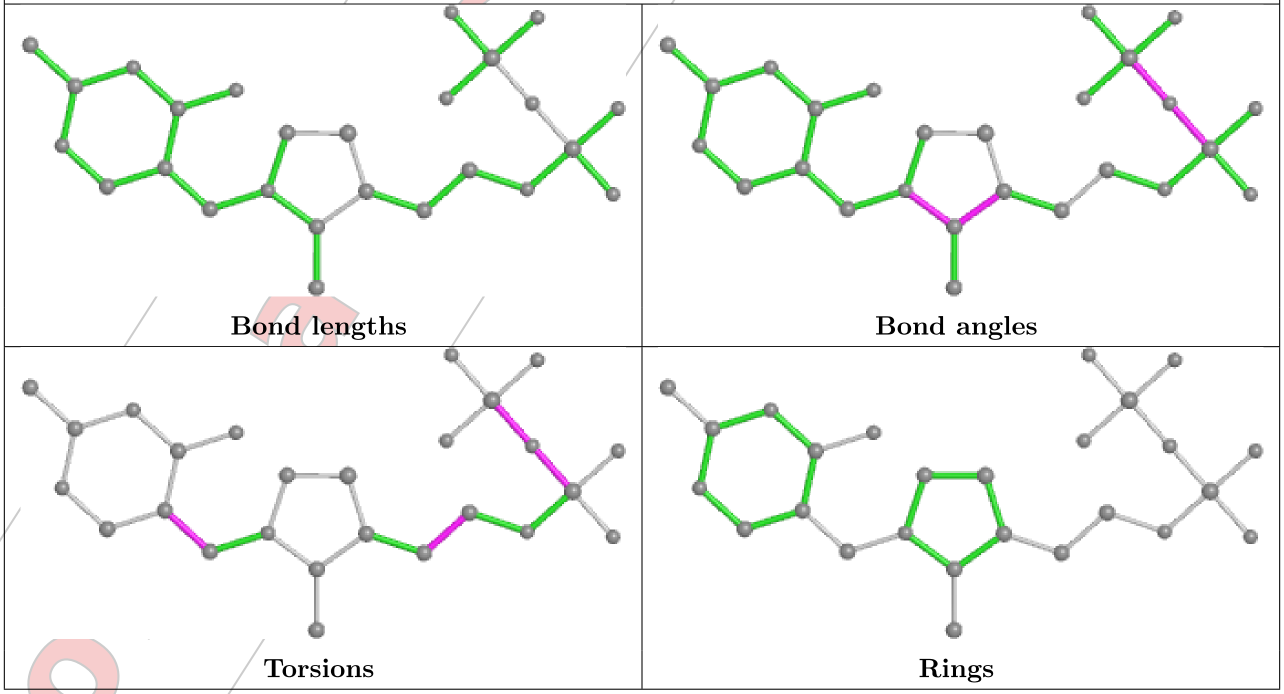

Ligand ANP K 901

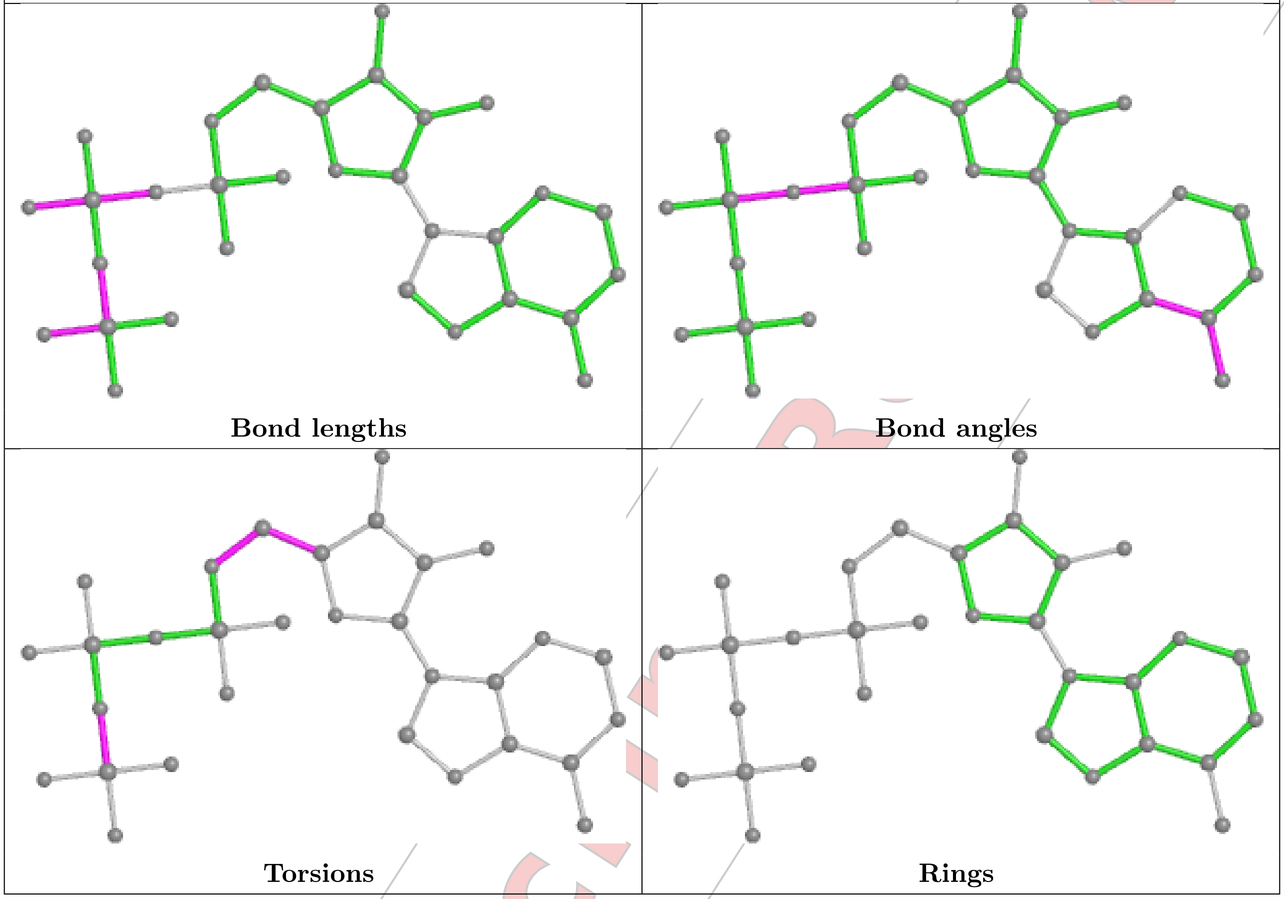

Ligand TPP C 902

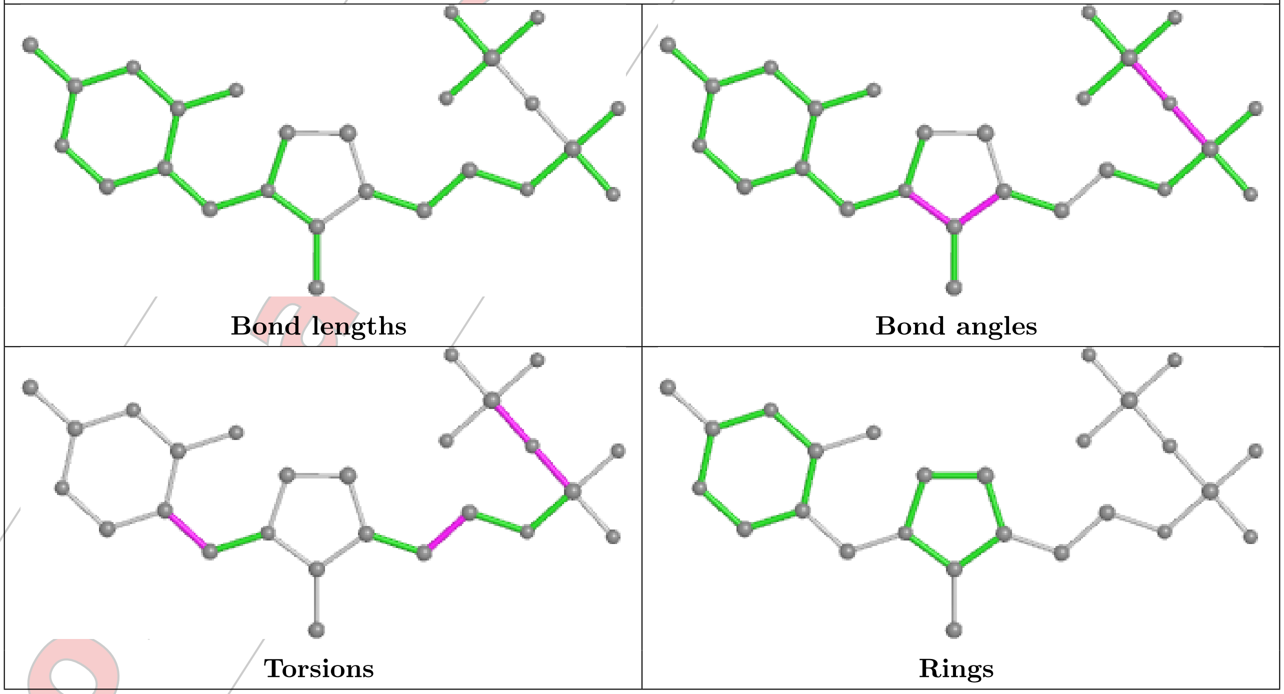

Ligand TPP K 902

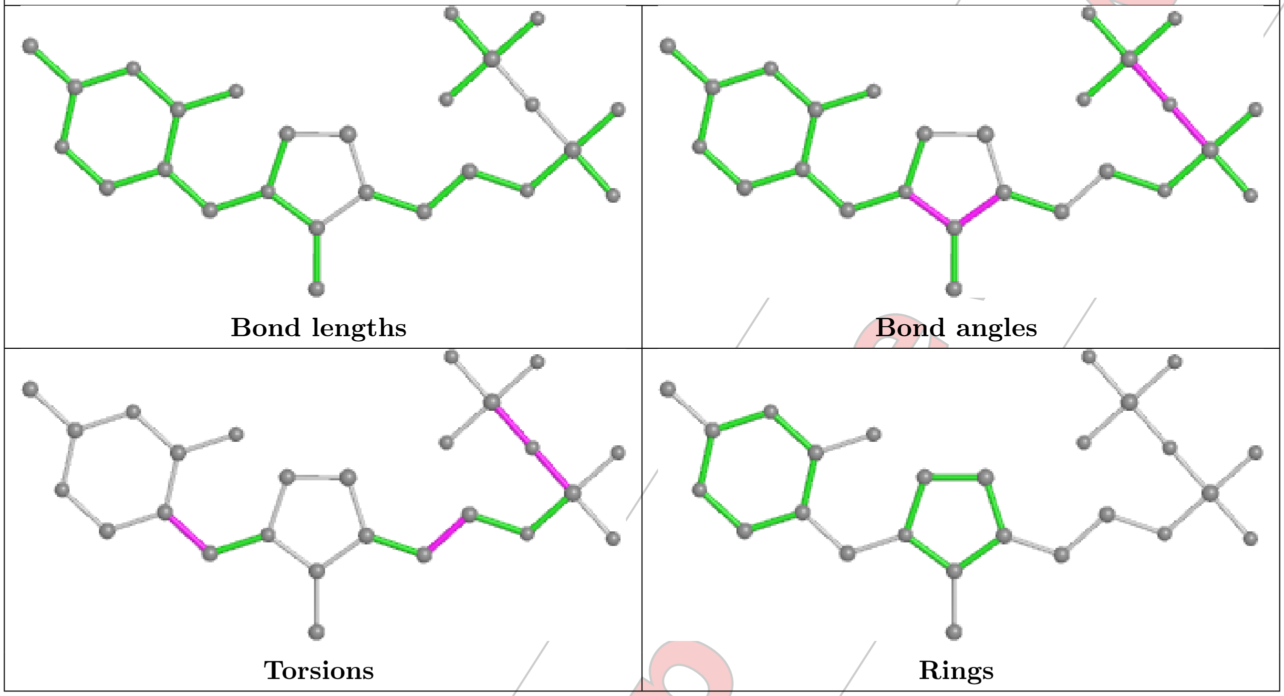

5.7 Other polymers (i)

There are no such residues in this entry.

5.8 Polymer linkage issues (i)

There are no chain breaks in this entry.

For Manuscript Review

6 Map visualisation (i)

This section contains visualisations of the EMDB entry EMD-35612. These allow visual inspection of the internal detail of the map and identification of artifacts.

Images derived from a raw map, generated by summing the deposited half-maps, are presented below the corresponding image components of the primary map to allow further visual inspection and comparison with those of the primary map.

6.1 Orthogonal projections (i)

6.1.1 Primary map

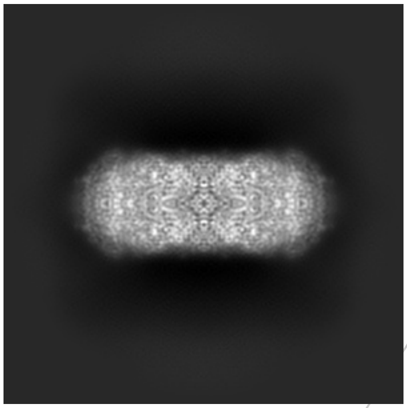
X

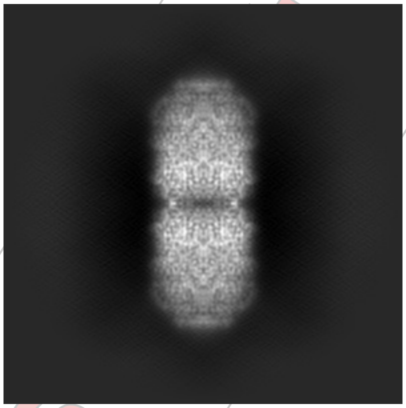
Y

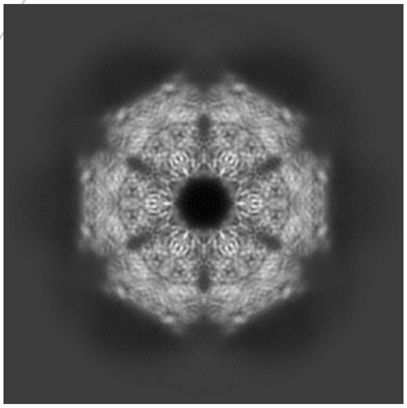
Z

6.1.2 Raw map

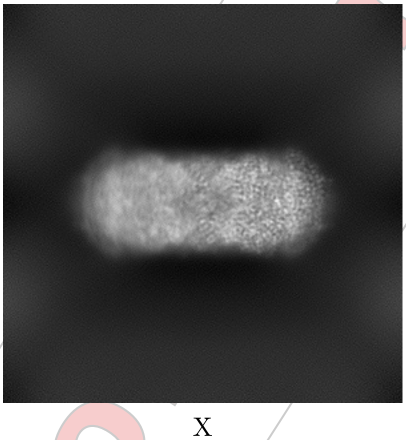
X

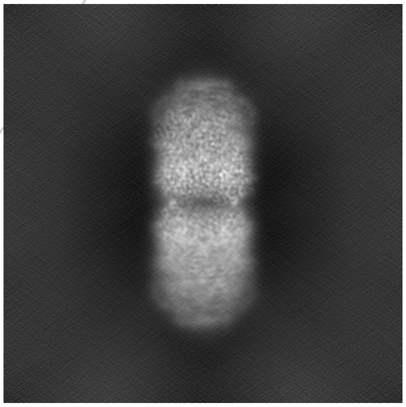
Y

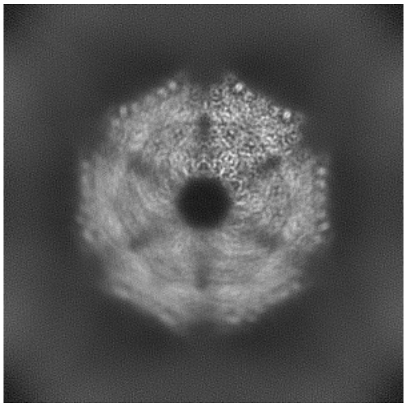
Z

The images above show the map projected in three orthogonal directions.

For Manuscript Review

6.2 Central slices (i)

6.2.1 Primary map

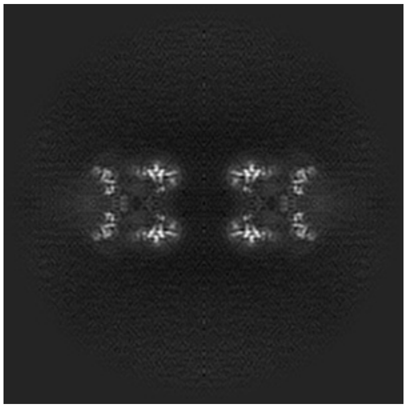

X Index: 192

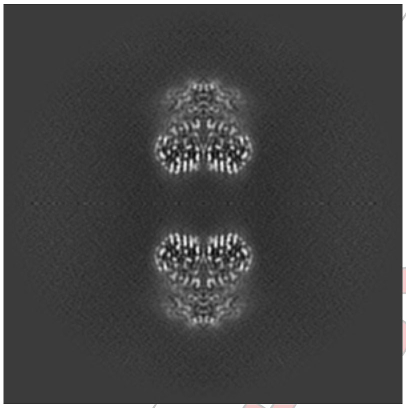

Y Index: 192

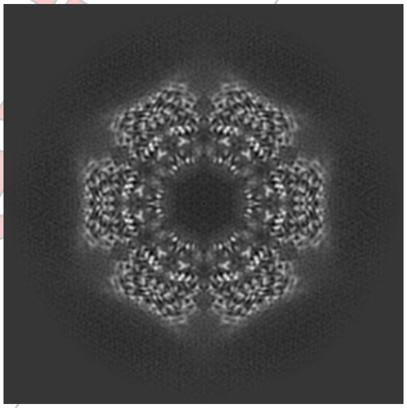

Z Index: 192

6.2.2 Raw map

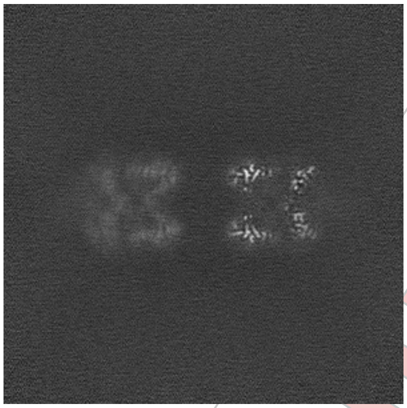

X Index: 192

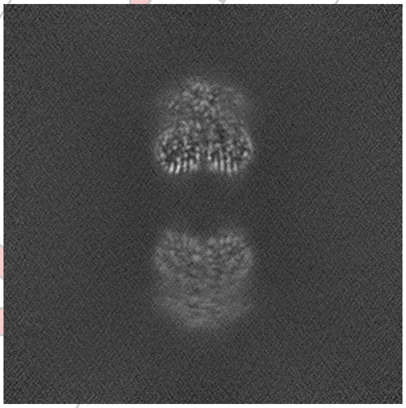

Y Index: 192

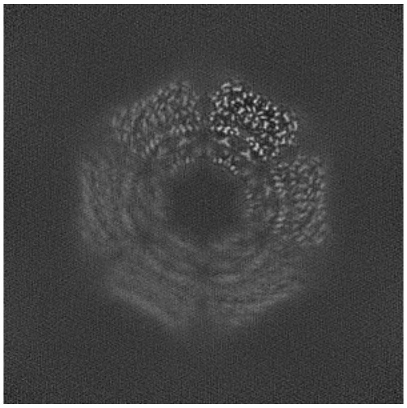

Z Index: 192

The images above show central slices of the map in three orthogonal directions.

For Manuscript Review

6.3 Largest variance slices

6.3.1 Primary map

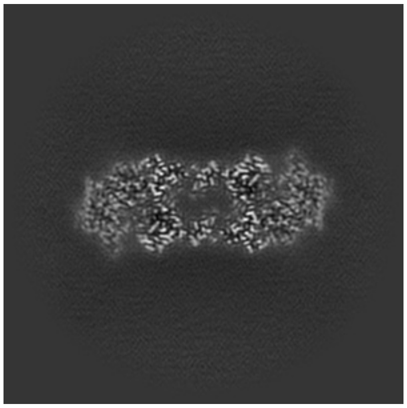
X Index: 223

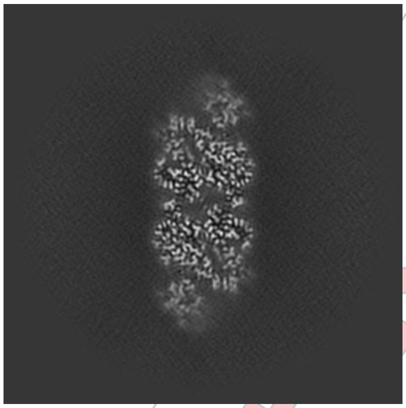
Y Index: 238

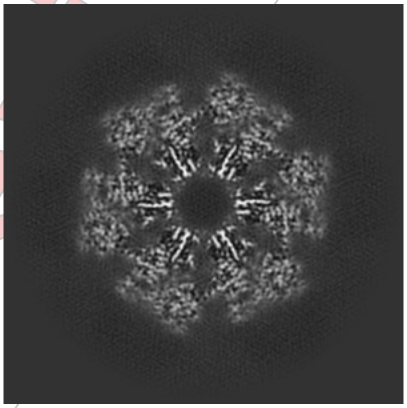
Z Index: 210

6.3.2 Raw map

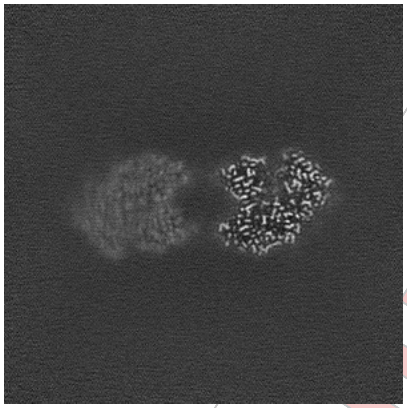
X Index: 213

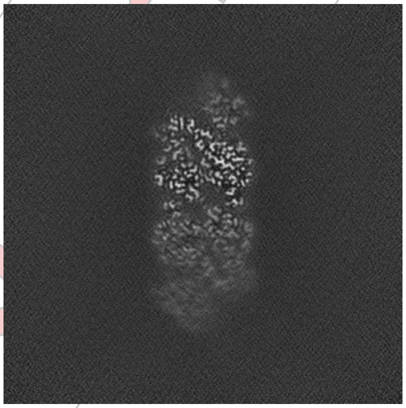
Y Index: 239

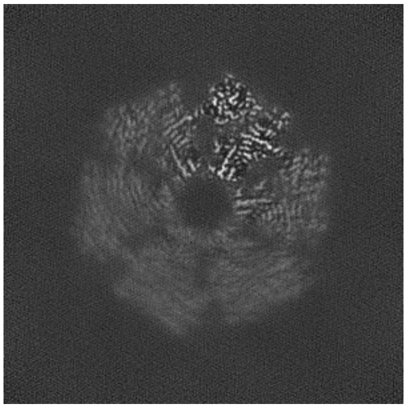
Z Index: 210

The images above show the largest variance slices of the map in three orthogonal directions.

For Manuscript Review

6.4 Orthogonal surface views (i)

6.4.1 Primary map

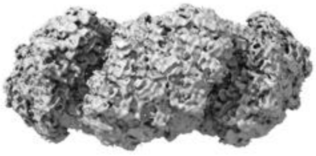

X

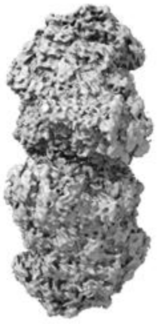

Y

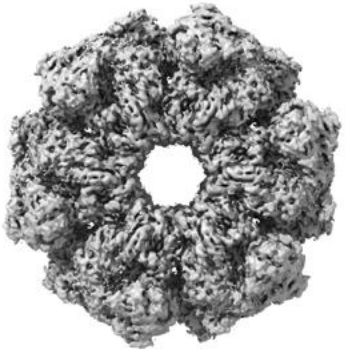

Z

The images above show the 3D surface view of the map at the recommended contour level 0.12. These images, in conjunction with the slice images, may facilitate assessment of whether an appropriate contour level has been provided.

6.4.2 Raw map

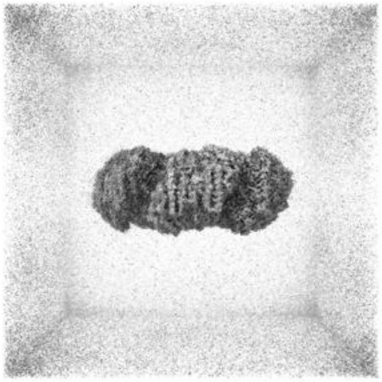

X

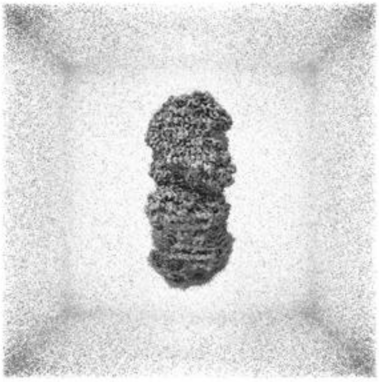

Y

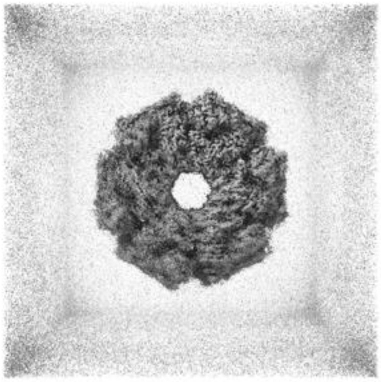

Z

These images show the 3D surface of the raw map. The raw map's contour level was selected so that its surface encloses the same volume as the primary map does at its recommended contour level.

6.5 Mask visualisation (i)

This section was not generated. No masks/segmentation were deposited.

For Manuscript Review

7 Map analysis (i)

This section contains the results of statistical analysis of the map.

7.1 Map-value distribution (i)

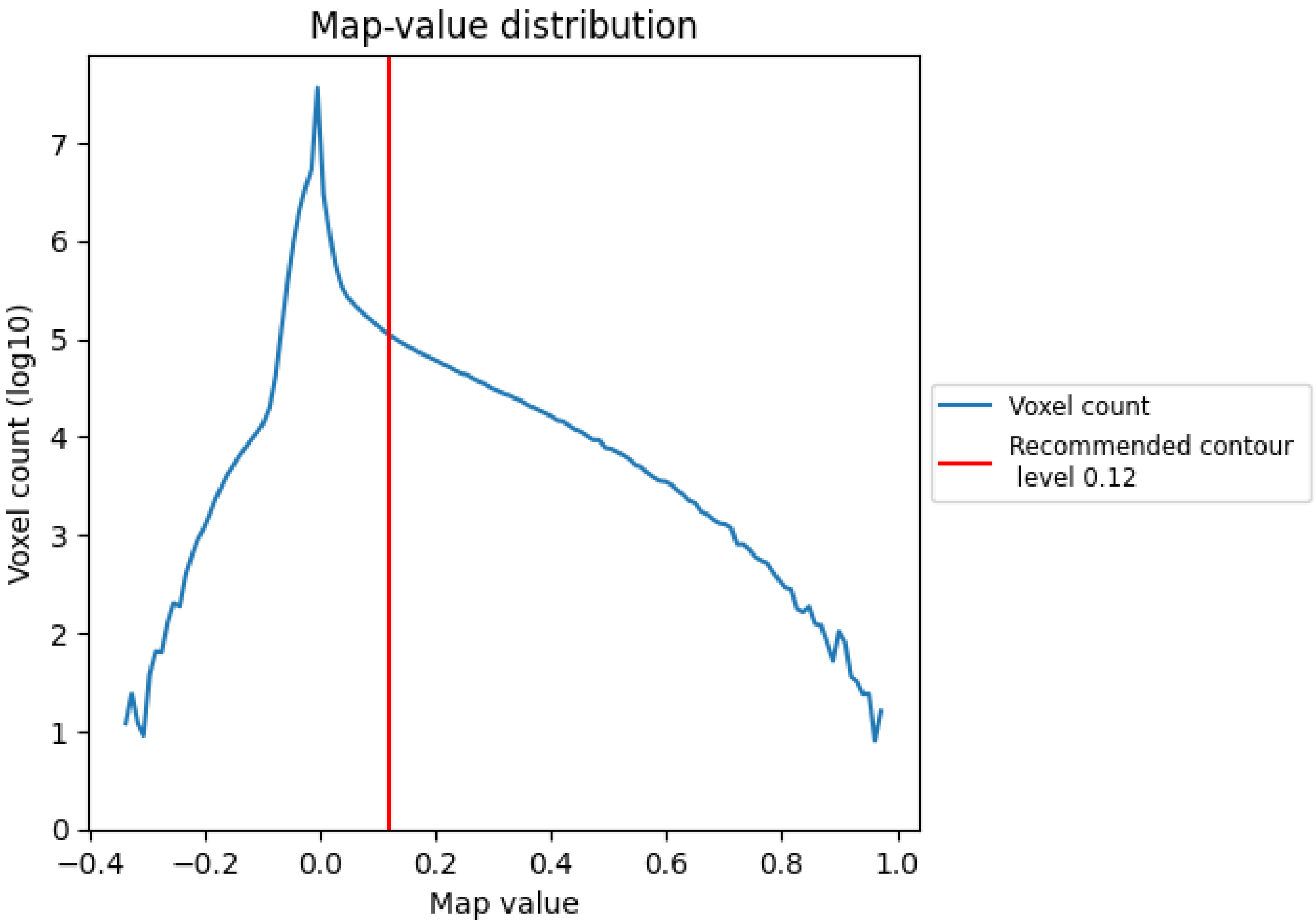

The map-value distribution is plotted in 128 intervals along the x-axis. The y-axis is logarithmic. A spike in this graph at zero usually indicates that the volume has been masked.

For Manuscript Review

7.2 Volume estimate ⓘ

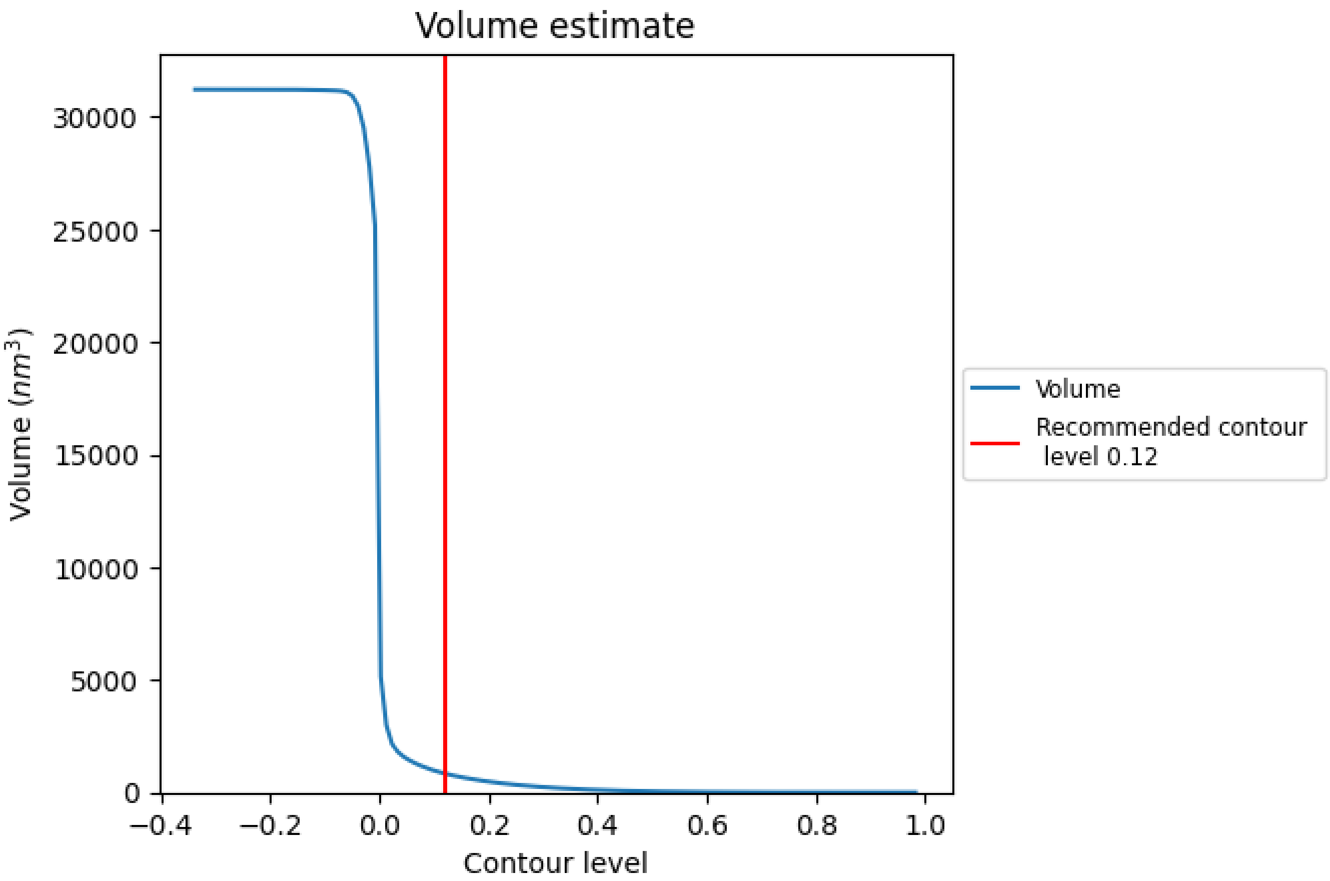

The volume at the recommended contour level is 837 nm^3; this corresponds to an approximate mass of 756 kDa.

The volume estimate graph shows how the enclosed volume varies with the contour level. The recommended contour level is shown as a vertical line and the intersection between the line and the curve gives the volume of the enclosed surface at the given level.

For Manuscript Review

7.3 Rotationally averaged power spectrum (i)

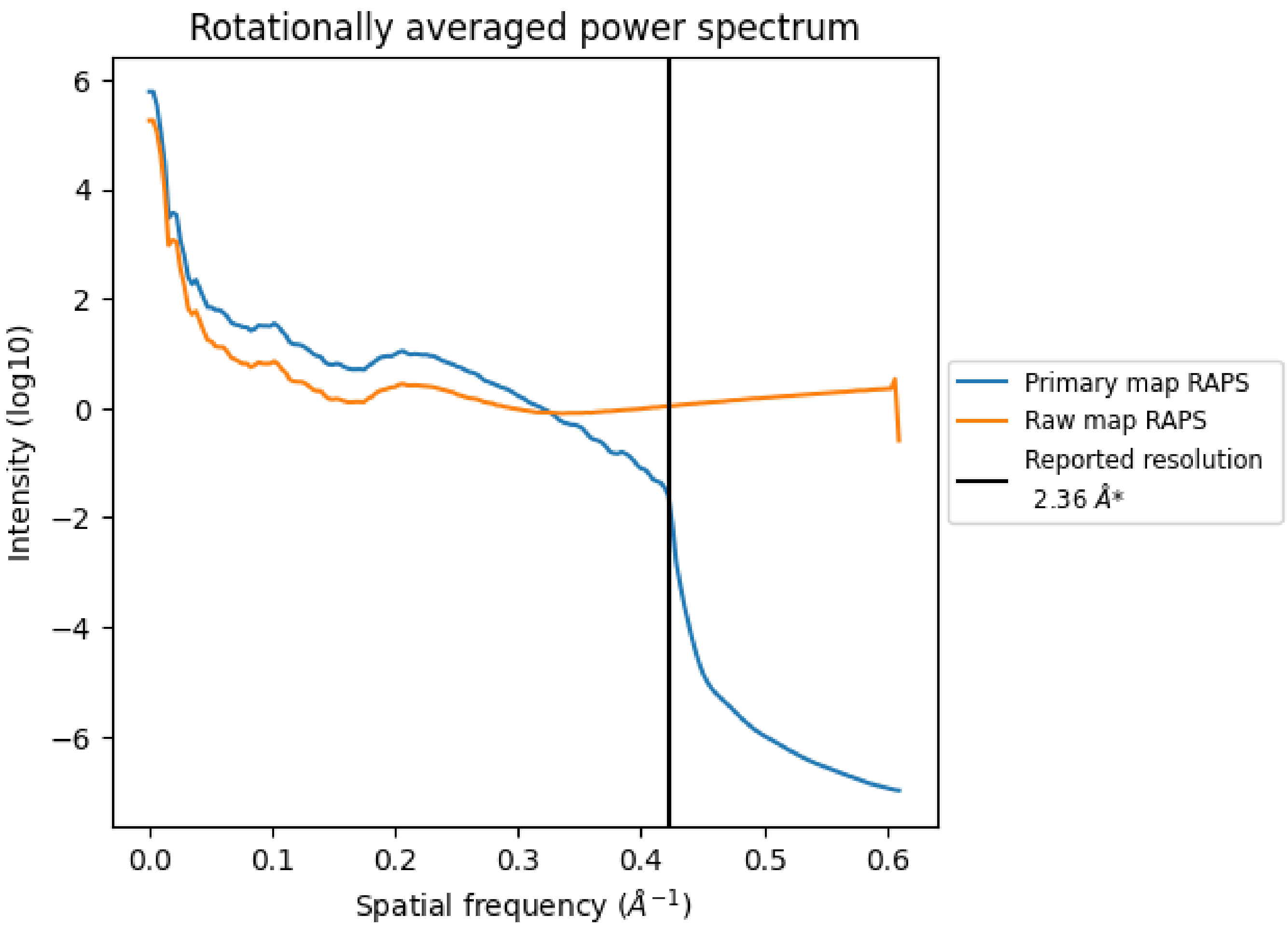

*Reported resolution corresponds to spatial frequency of 0.424 Å^{-1}

For Manuscript Review

8 Fourier-Shell correlation ⓘ

Fourier-Shell Correlation (FSC) is the most commonly used method to estimate the resolution of single-particle and subtomogram-averaged maps. The shape of the curve depends on the imposed symmetry, mask and whether or not the two 3D reconstructions used were processed from a common reference. The reported resolution is shown as a black line. A curve is displayed for the half-bit criterion in addition to lines showing the 0.143 gold standard cut-off and 0.5 cut-off.

8.1 FSC ⓘ

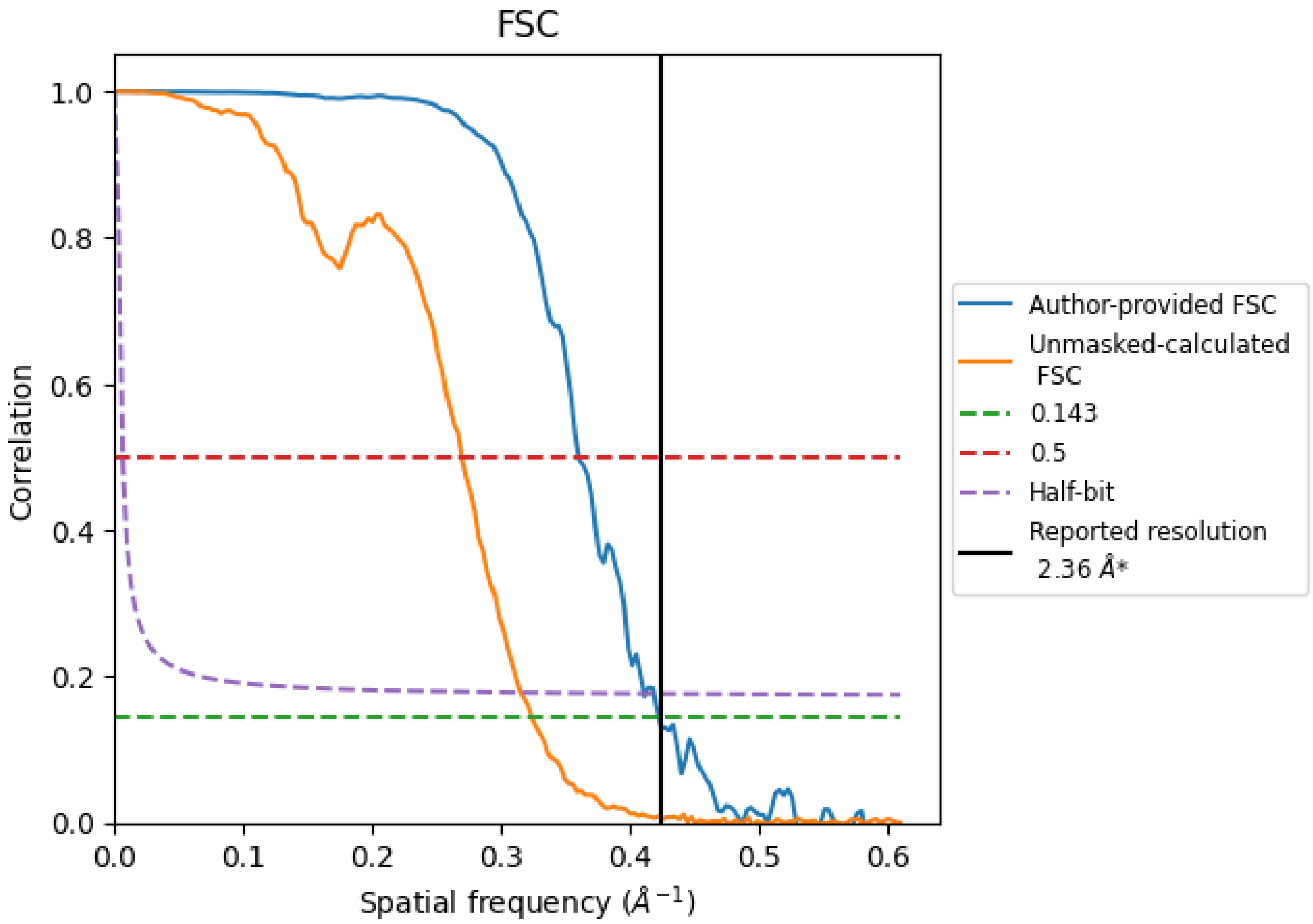

*Reported resolution corresponds to spatial frequency of 0.424 Å^{-1}

For Manuscript Review

8.2 Resolution estimates (i)

Resolution estimate (Å)	Estimation criterion (FSC cut-off)		
	0.143	0.5	Half-bit
Reported by author	2.36	-	-
Author-provided FSC curve	2.37	2.78	2.43
Unmasked-calculated*	3.09	3.71	3.17

*Resolution estimate based on FSC curve calculated by comparison of deposited half-maps. The value from deposited half-maps intersecting FSC 0.143 CUT-OFF 3.09 differs from the reported value 2.36 by more than 10 %

For Manuscript Review

9 Map-model fit (i)

This section contains information regarding the fit between EMDB map EMD-35612 and PDB model 8IO9. Per-residue inclusion information can be found in section 3 on page 8.

9.1 Map-model overlay (i)

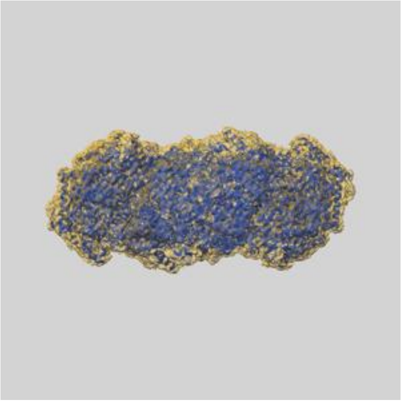

X

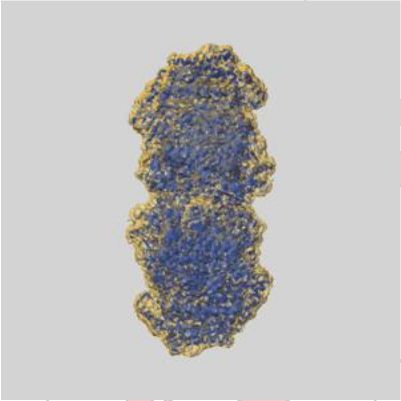

Y

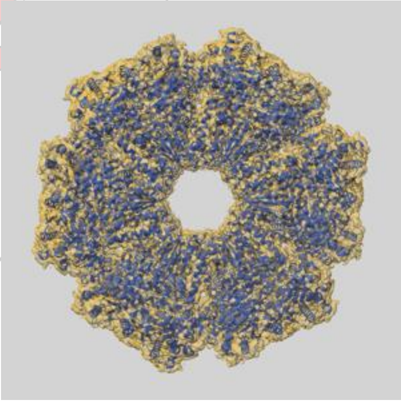

Z

The images above show the 3D surface view of the map at the recommended contour level 0.12 at 50% transparency in yellow overlaid with a ribbon representation of the model coloured in blue. These images allow for the visual assessment of the quality of fit between the atomic model and the map.

For Manuscript Review

9.2 Q-score mapped to coordinate model ⓘ

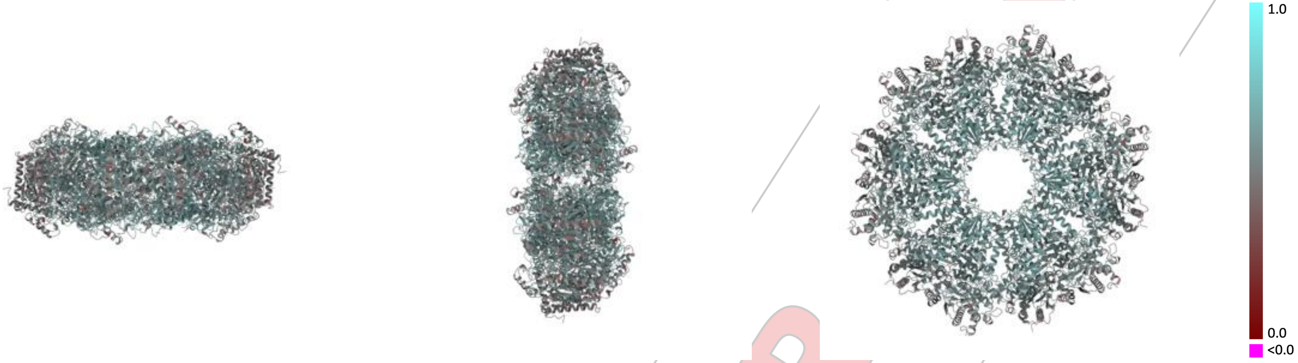

X Y Z

The images above show the model with each residue coloured according its Q-score. This shows their resolvability in the map with higher Q-score values reflecting better resolvability. Please note: Q-score is calculating the resolvability of atoms, and thus high values are only expected at resolutions at which atoms can be resolved. Low Q-score values may therefore be expected for many entries.

9.3 Atom inclusion mapped to coordinate model ⓘ

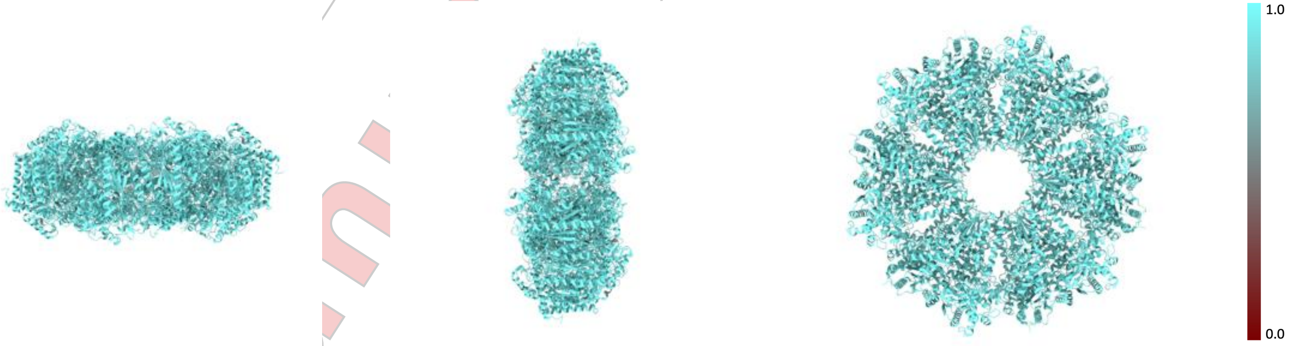

X Y Z

The images above show the model with each residue coloured according to its atom inclusion. This shows to what extent they are inside the map at the recommended contour level (0.12).

For Manuscript Review

9.4 Atom inclusion (i)

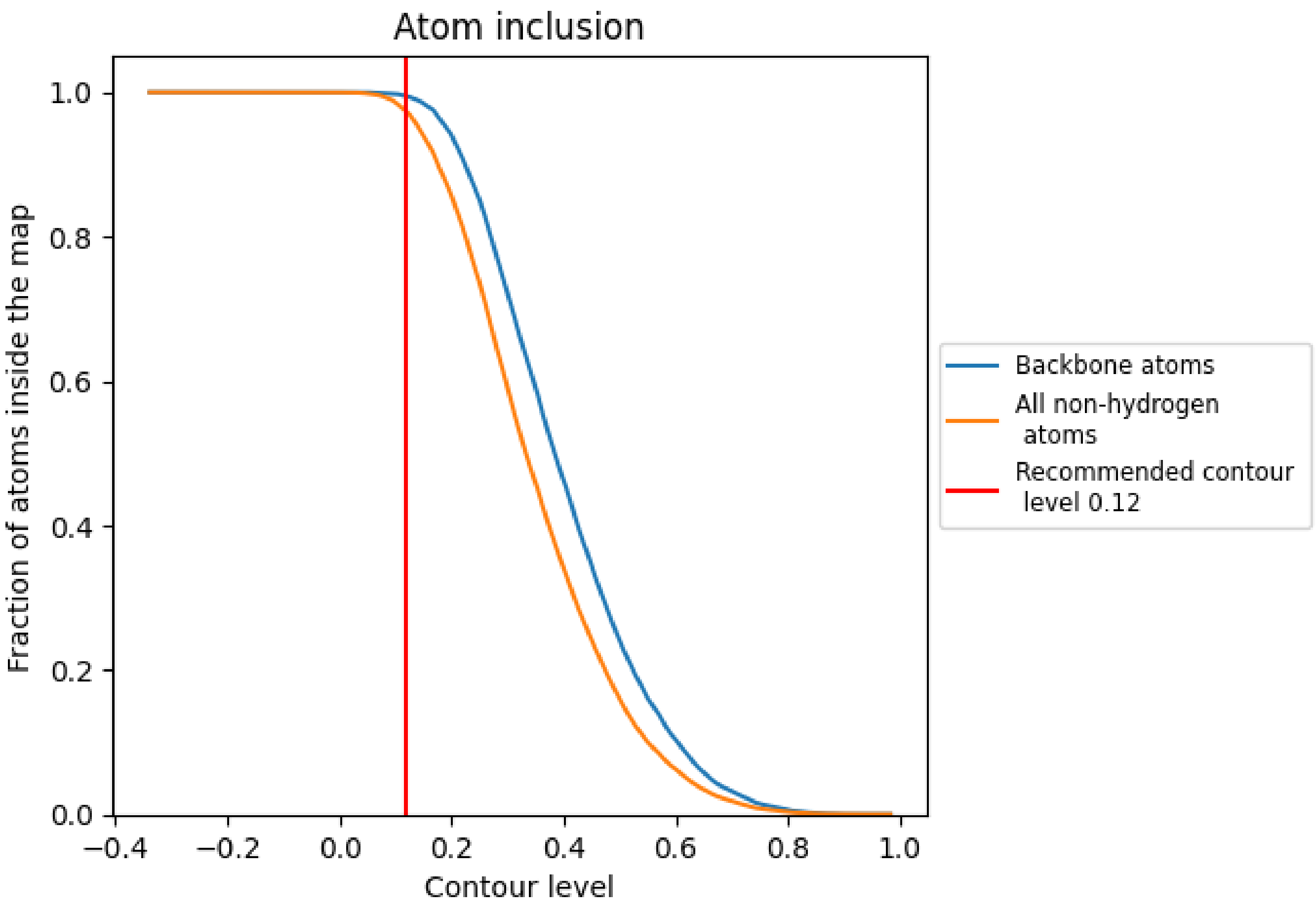

At the recommended contour level, 99% of all backbone atoms, 97% of all non-hydrogen atoms, are inside the map.

For Manuscript Review

9.5 Map-model fit summary (i)

The table lists the average atom inclusion at the recommended contour level (0.12) and Q-score for the entire model and for each chain.

Chain	Atom inclusion	Q-score
All	0.9730	0.5560
A	0.9742	0.5560
B	0.9727	0.5550
C	0.9716	0.5560
D	0.9708	0.5560
E	0.9741	0.5560
F	0.9729	0.5550
G	0.9742	0.5570
H	0.9731	0.5550
I	0.9729	0.5560
J	0.9716	0.5540
K	0.9742	0.5560
L	0.9737	0.5550

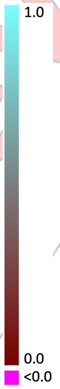

For Manuscript Review
