# Supplementary material for: An ATP-sensitive phosphoketolase regulates carbon fixation in cyanobacteria
Source: Nat Metab. 2023 Jun 22;5(7):1111–26. doi: 10.1038/s42255-023-00831-w (PMC10365998; doi:10.1038/s42255-023-00831-w)
Supplement: Supplementary file 6 — PDB validation reports for the cryo-EM structures of SeXPK and BlXPK. [file 42255_2023_831_MOESM6_ESM.zip › NATMETAB-A22127983A XPK_PDB-Validation report revision1/D_1300035944_val-report-full_8IO6.pdf]

Full wwPDB EM Validation Report (i)

Mar 21, 2023 – 08:42 PM JST

PDB ID	:	8IO6
EMDB ID	:	EMD-35609
Title	:	Cryo-EM structure of phosphoketolase from Bifidobacterium longum in octameric assembly
Deposited on	:	2023-03-10
Resolution	:	2.68 Å(reported)
Based on initial model	:	3AI7

This wwPDB validation report is for manuscript review

This is a Full wwPDB EM Validation Report.

This report is produced by the wwPDB biocuration pipeline after annotation of the structure.

We welcome your comments at *validation@mail.wwpdb.org*
A user guide is available at
https://www.wwpdb.org/validation/2017/EMValidationReportHelp
with specific help available everywhere you see the (i) symbol.

The types of validation reports are described at
http://www.wwpdb.org/validation/2017/FAQs#types.

The following versions of software and data (see references (i)) were used in the production of this report:

EMDB validation analysis	:	0.0.1.dev43
Mogul	:	1.8.5 (274361), CSD as541be (2020)
MolProbity	:	4.02b-467
buster-report	:	1.1.7 (2018)
Percentile statistics	:	20191225.v01 (using entries in the PDB archive December 25th 2019)
MapQ	:	1.9.9
Ideal geometry (proteins)	:	Engh & Huber (2001)

For Manuscript Review

1 Overall quality at a glance (i)

The following experimental techniques were used to determine the structure:
ELECTRON MICROSCOPY

The reported resolution of this entry is 2.68 Å.

Percentile scores (ranging between 0-100) for global validation metrics of the entry are shown in the following graphic. The table shows the number of entries on which the scores are based.

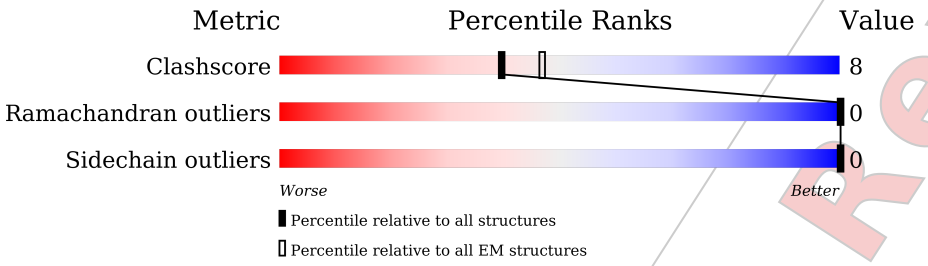

Metric	Whole archive (#Entries)	EM structures (#Entries)
Clashscore	158937	4297
Ramachandran outliers	154571	4023
Sidechain outliers	154315	3826

The table below summarises the geometric issues observed across the polymeric chains and their fit to the map. The red, orange, yellow and green segments of the bar indicate the fraction of residues that contain outliers for >=3, 2, 1 and 0 types of geometric quality criteria respectively. A grey segment represents the fraction of residues that are not modelled. The numeric value for each fraction is indicated below the corresponding segment, with a dot representing fractions <=5% The upper red bar (where present) indicates the fraction of residues that have poor fit to the EM map (all-atom inclusion < 40%). The numeric value is given above the bar.

Mol	Chain	Length	Quality of chain
1	A	825	79% 18% •
1	B	825	79% 18% •
1	C	825	79% 18% •
1	D	825	79% 19% •
1	E	825	79% 18% •
1	F	825	79% 19% •

Continued on next page...

Ideal geometry (DNA, RNA) : Parkinson et al. (1996)
Validation Pipeline (wwPDB-VP) : 2.32.1

For Manuscript Review

Continued from previous page...

Mol	Chain	Length	Quality of chain
1	G	825	79% 19% •
1	H	825	79% 18% •

For Manuscript Review

2 Entry composition (i)

There are 3 unique types of molecules in this entry. The entry contains 51504 atoms, of which 0 are hydrogens and 0 are deuteriums.

In the tables below, the AltConf column contains the number of residues with at least one atom in alternate conformation and the Trace column contains the number of residues modelled with at most 2 atoms.

- Molecule 1 is a protein called Xylulose5phosphatefructose6phosphate phosphoketolase.

Mol	Chain	Residues	Atoms					AltConf	Trace
1	A	806	Total 6411	C 4071	N 1093	O 1228	S 19	0	0
1	B	806	Total 6411	C 4071	N 1093	O 1228	S 19	0	0
1	C	806	Total 6411	C 4071	N 1093	O 1228	S 19	0	0
1	D	806	Total 6411	C 4071	N 1093	O 1228	S 19	0	0
1	E	806	Total 6411	C 4071	N 1093	O 1228	S 19	0	0
1	F	806	Total 6411	C 4071	N 1093	O 1228	S 19	0	0
1	G	806	Total 6411	C 4071	N 1093	O 1228	S 19	0	0
1	H	806	Total 6411	C 4071	N 1093	O 1228	S 19	0	0

- Molecule 2 is THIAMINE DIPHOSPHATE (three-letter code: TPP) (formula: $C_{12}H_{19}N_4O_7P_2S$) (labeled as "Ligand of Interest" by depositor).

For Manuscript Review

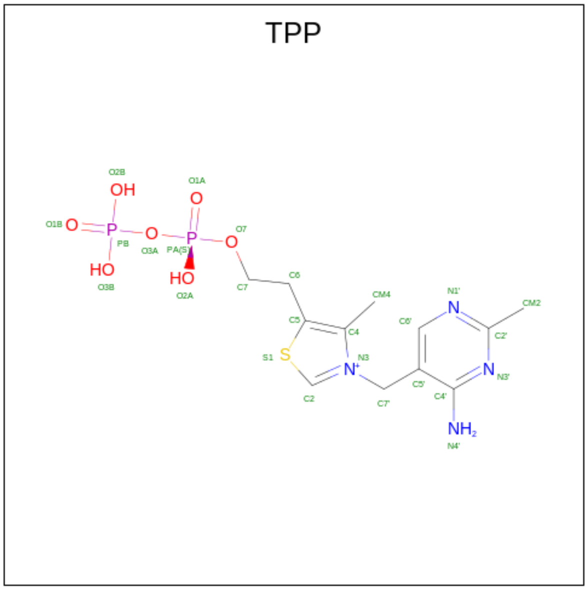

Mol	Chain	Residues	Atoms	AltConf
2	A	1	Total C N O P S<br>26 12 4 7 2 1	0
2	B	1	Total C N O P S<br>26 12 4 7 2 1	0
2	C	1	Total C N O P S<br>26 12 4 7 2 1	0
2	D	1	Total C N O P S<br>26 12 4 7 2 1	0
2	E	1	Total C N O P S<br>26 12 4 7 2 1	0
2	F	1	Total C N O P S<br>26 12 4 7 2 1	0
2	G	1	Total C N O P S<br>26 12 4 7 2 1	0
2	H	1	Total C N O P S<br>26 12 4 7 2 1	0

- Molecule 3 is MAGNESIUM ION (three-letter code: MG) (formula: Mg) (labeled as "Ligand of Interest" by depositor).

Mol	Chain	Residues	Atoms	AltConf
3	A	1	Total Mg<br>1 1	0
3	B	1	Total Mg<br>1 1	0
3	C	1	Total Mg<br>1 1	0

Continued on next page...

For Manuscript Review

Continued from previous page...

Mol	Chain	Residues	Atoms	AltConf
3	D	1	Total Mg<br>1 1	0
3	E	1	Total Mg<br>1 1	0
3	F	1	Total Mg<br>1 1	0
3	G	1	Total Mg<br>1 1	0
3	H	1	Total Mg<br>1 1	0

For Manuscript Review

3 Residue-property plots ⓘ

These plots are drawn for all protein, RNA, DNA and oligosaccharide chains in the entry. The first graphic for a chain summarises the proportions of the various outlier classes displayed in the second graphic. The second graphic shows the sequence view annotated by issues in geometry and atom inclusion in map density. Residues are color-coded according to the number of geometric quality criteria for which they contain at least one outlier: green = 0, yellow = 1, orange = 2 and red = 3 or more. A red diamond above a residue indicates a poor fit to the EM map for this residue (all-atom inclusion < 40%). Stretches of 2 or more consecutive residues without any outlier are shown as a green connector. Residues present in the sample, but not in the model, are shown in grey.

• Molecule 1: Xylulose5phosphatefructose6phosphate phosphoketolase

Chain A:

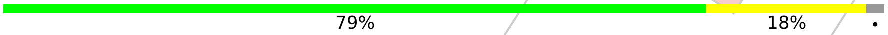

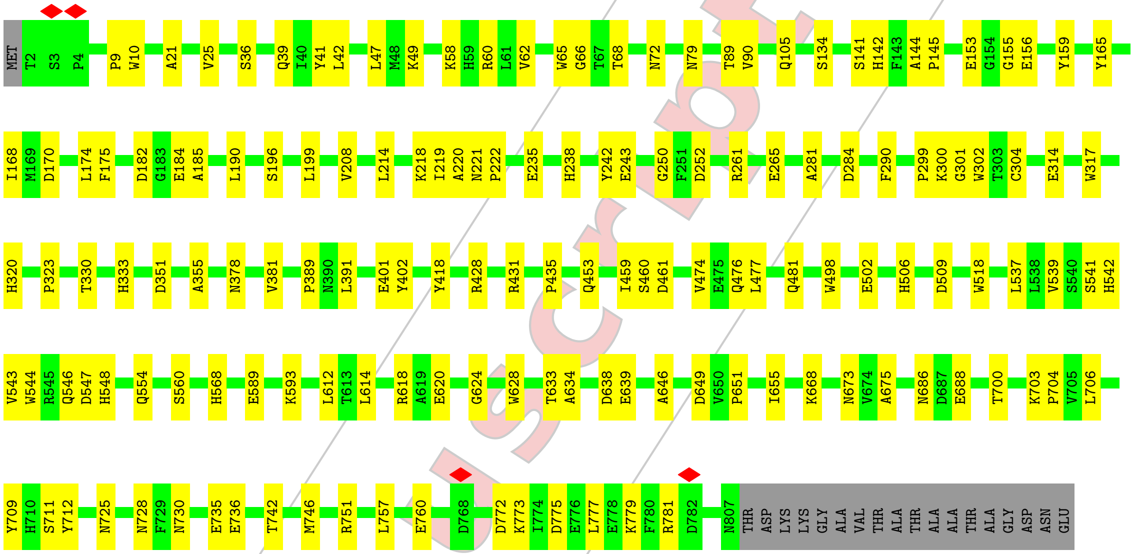

• Molecule 1: Xylulose5phosphatefructose6phosphate phosphoketolase

Chain B:

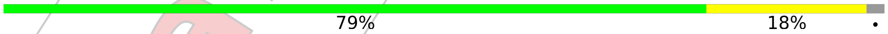

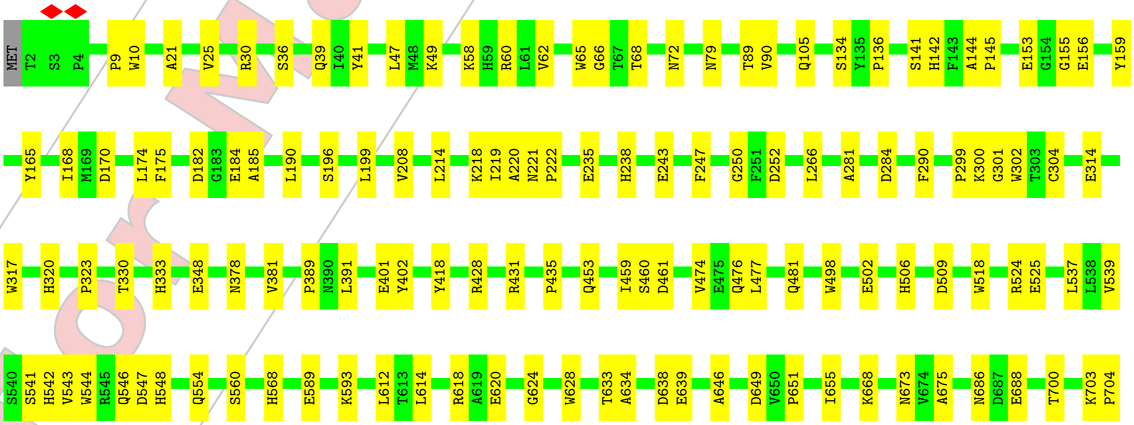

For Manuscript Review

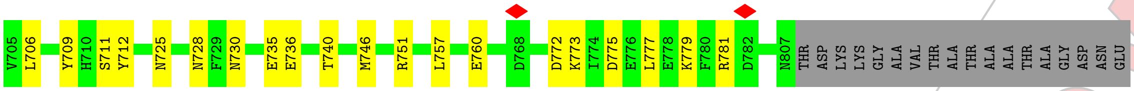

• Molecule 1: Xylulose5phosphatefructose6phosphate phosphoketolase

Chain C:

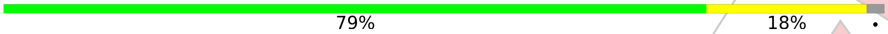

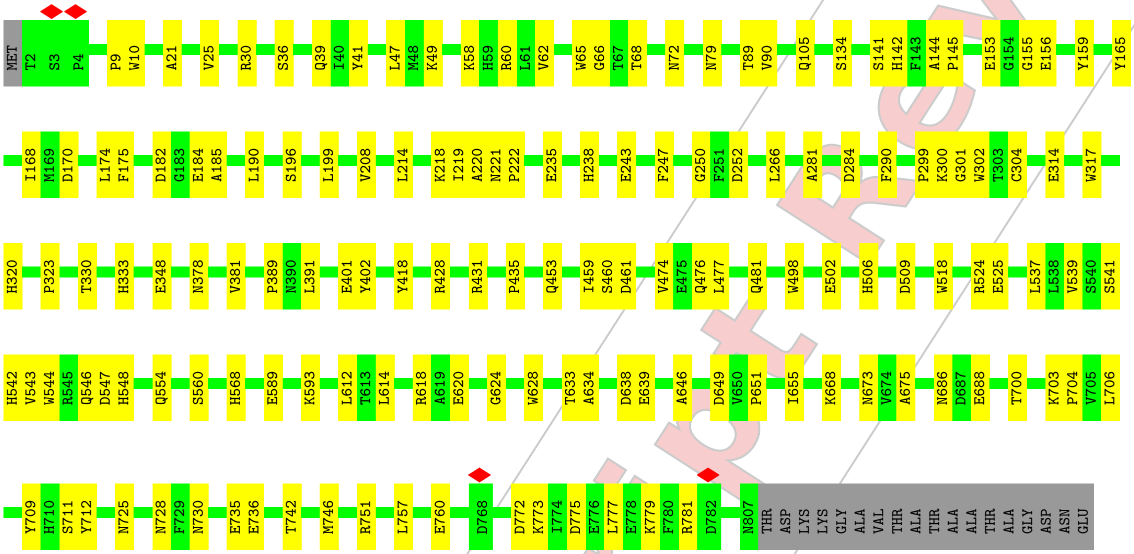

• Molecule 1: Xylulose5phosphatefructose6phosphate phosphoketolase

Chain D:

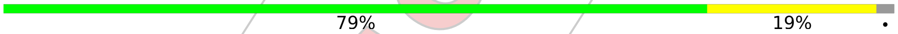

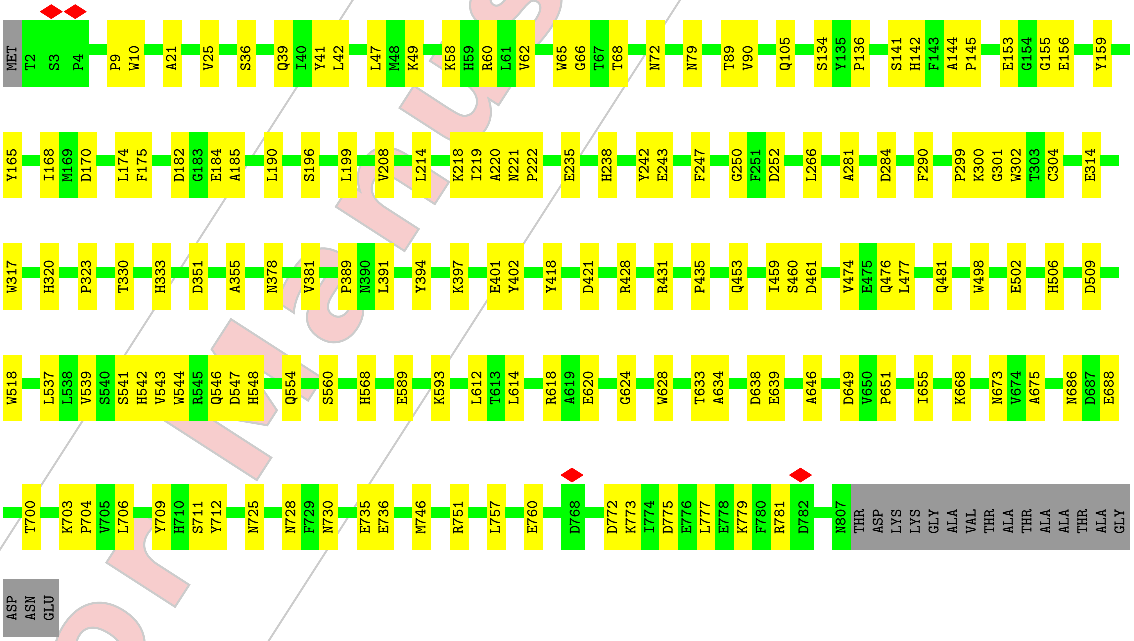

• Molecule 1: Xylulose5phosphatefructose6phosphate phosphoketolase

Chain E:

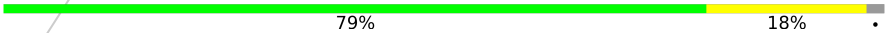

For Manuscript Review

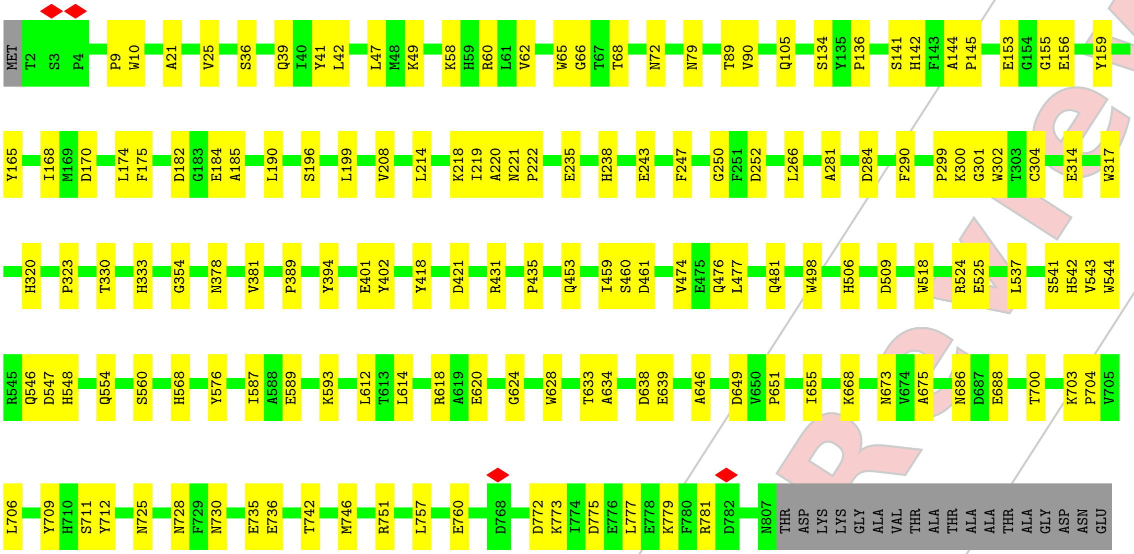

• Molecule 1: Xylulose5phosphatefructose6phosphate phosphoketolase

Chain F:

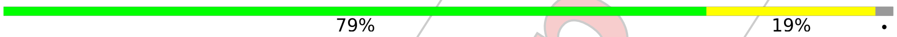

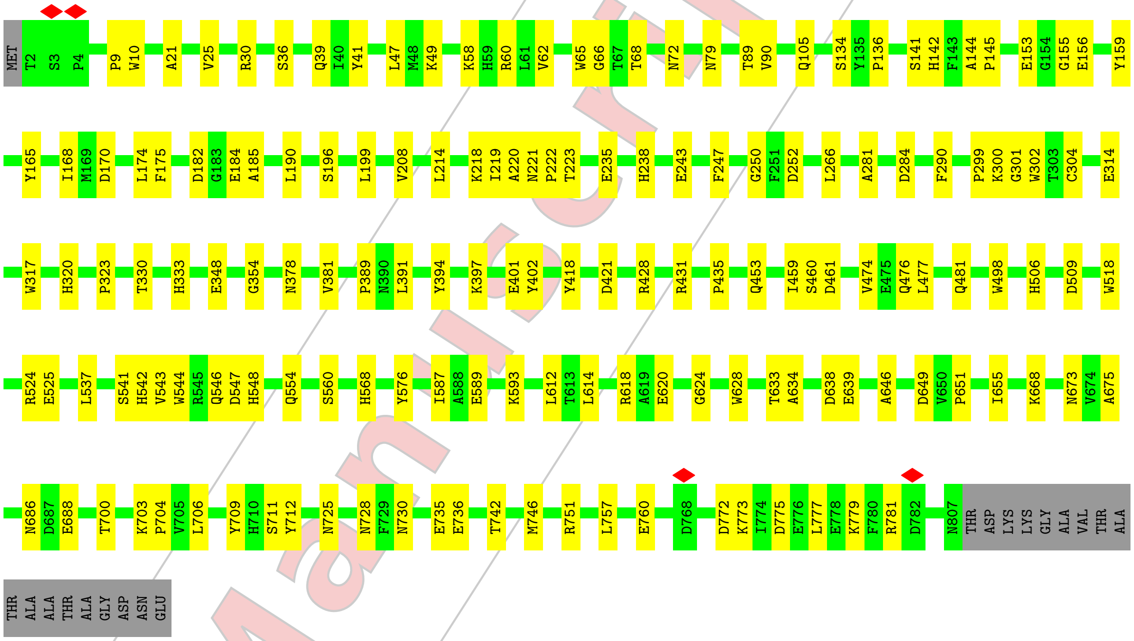

• Molecule 1: Xylulose5phosphatefructose6phosphate phosphoketolase

Chain G:

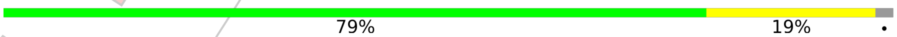

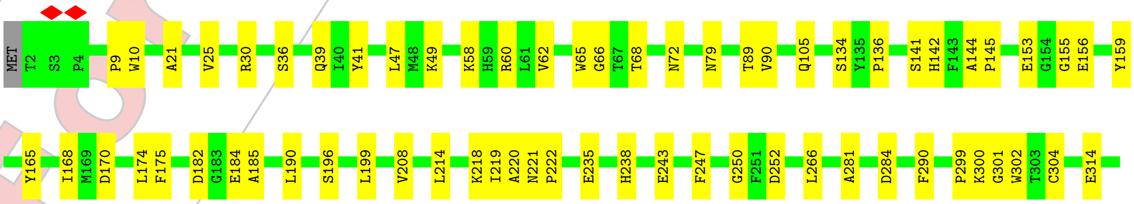

For Manuscript Review

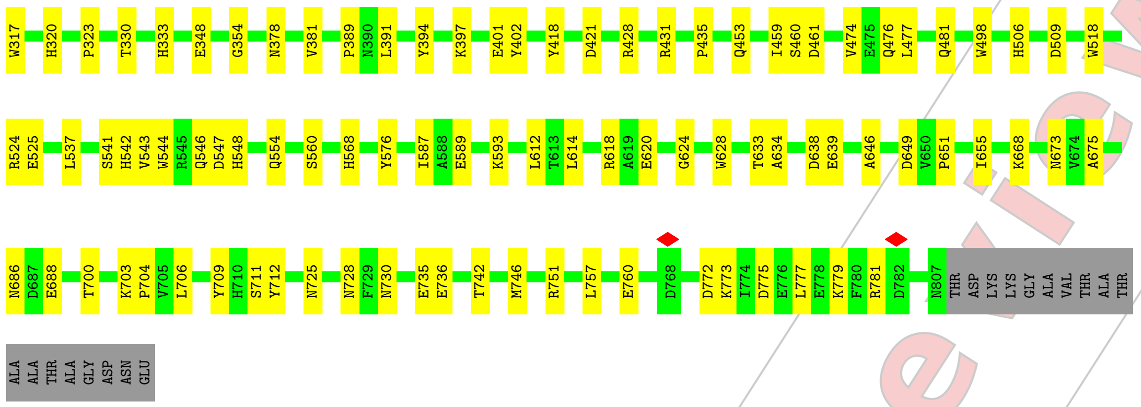

- Molecule 1: Xylulose5phosphatefructose6phosphate phosphoketolase

Chain H:

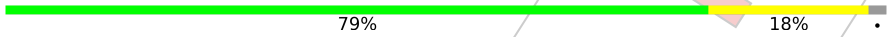

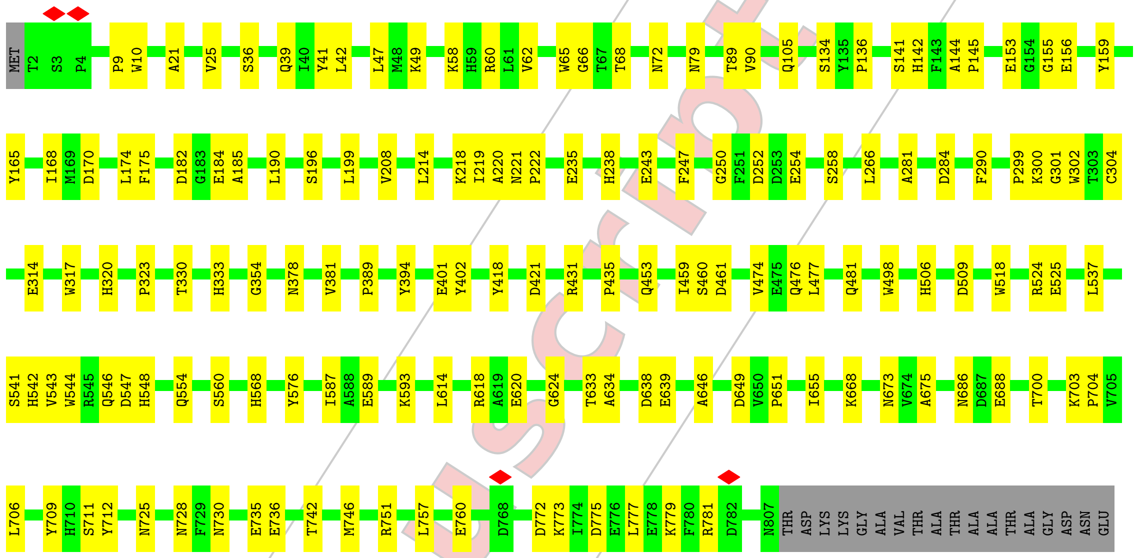

For Manuscript Review

4 Experimental information (i)

Property	Value	Source
EM reconstruction method	SINGLE PARTICLE	Depositor
Imposed symmetry	POINT, Not provided	
Number of particles used	590627	Depositor
Resolution determination method	FSC 0.143 CUT-OFF	Depositor
CTF correction method	PHASE FLIPPING AND AMPLITUDE CORRECTION	Depositor
Microscope	FEI TITAN KRIOS	Depositor
Voltage (kV)	300	Depositor
Electron dose ($e^-/\text{Å}^2$)	42	Depositor
Minimum defocus (nm)	500	Depositor
Maximum defocus (nm)	2500	Depositor
Magnification	105000	Depositor
Image detector	GATAN K3 BIOQUANTUM (6k x 4k)	Depositor
Maximum map value	1.321	Depositor
Minimum map value	-0.495	Depositor
Average map value	0.004	Depositor
Map value standard deviation	0.053	Depositor
Recommended contour level	0.238	Depositor
Map size (Å)	318.72, 318.72, 318.72	wwPDB
Map dimensions	384, 384, 384	wwPDB
Map angles (°)	90.0, 90.0, 90.0	wwPDB
Pixel spacing (Å)	0.83, 0.83, 0.83	Depositor

For Manuscript Review

5 Model quality (i)

5.1 Standard geometry (i)

Bond lengths and bond angles in the following residue types are not validated in this section: MG, TPP

The Z score for a bond length (or angle) is the number of standard deviations the observed value is removed from the expected value. A bond length (or angle) with $|Z| > 5$ is considered an outlier worth inspection. RMSZ is the root-mean-square of all Z scores of the bond lengths (or angles).

Mol	Chain	Bond lengths		Bond angles	
		RMSZ	#|Z| >5	RMSZ	#|Z| >5
1	A	0.39	0/6588	0.47	0/8960
1	B	0.39	0/6588	0.47	0/8960
1	C	0.39	0/6588	0.47	0/8960
1	D	0.39	0/6588	0.47	0/8960
1	E	0.39	0/6588	0.47	0/8960
1	F	0.39	0/6588	0.47	0/8960
1	G	0.39	0/6588	0.47	0/8960
1	H	0.39	0/6588	0.47	0/8960
All	All	0.39	0/52704	0.47	0/71680

There are no bond length outliers.

There are no bond angle outliers.

There are no chirality outliers.

There are no planarity outliers.

5.2 Too-close contacts (i)

In the following table, the Non-H and H(model) columns list the number of non-hydrogen atoms and hydrogen atoms in the chain respectively. The H(added) column lists the number of hydrogen atoms added and optimized by MolProbity. The Clashes column lists the number of clashes within the asymmetric unit, whereas Symm-Clashes lists symmetry-related clashes.

Mol	Chain	Non-H	H(model)	H(added)	Clashes	Symm-Clashes
1	A	6411	0	6101	104	0
1	B	6411	0	6101	102	0
1	C	6411	0	6101	102	0
1	D	6411	0	6101	105	0
1	E	6411	0	6101	103	0

Continued on next page...

For Manuscript Review

Continued from previous page...

Mol	Chain	Non-H	H(model)	H(added)	Clashes	Symm-Clashes
1	F	6411	0	6101	107	0
1	G	6411	0	6101	106	0
1	H	6411	0	6101	103	0
2	A	26	0	16	0	0
2	B	26	0	16	0	0
2	C	26	0	16	0	0
2	D	26	0	16	0	0
2	E	26	0	16	1	0
2	F	26	0	16	1	0
2	G	26	0	16	1	0
2	H	26	0	16	1	0
3	A	1	0	0	0	0
3	B	1	0	0	0	0
3	C	1	0	0	0	0
3	D	1	0	0	0	0
3	E	1	0	0	0	0
3	F	1	0	0	0	0
3	G	1	0	0	0	0
3	H	1	0	0	0	0
All	All	51504	0	48936	769	0

The all-atom clashscore is defined as the number of clashes found per 1000 atoms (including hydrogen atoms). The all-atom clashscore for this structure is 8.

All (769) close contacts within the same asymmetric unit are listed below, sorted by their clash magnitude.

Atom-1	Atom-2	Interatomic distance (Å)	Clash overlap (Å)
1:E:196:SER:OG	1:E:481:GLN:NE2	2.16	0.79
1:B:196:SER:OG	1:B:481:GLN:NE2	2.16	0.79
1:C:196:SER:OG	1:C:481:GLN:NE2	2.16	0.79
1:D:196:SER:OG	1:D:481:GLN:NE2	2.16	0.78
1:A:196:SER:OG	1:A:481:GLN:NE2	2.16	0.78
1:B:543:VAL:HG23	1:B:554:GLN:HB3	1.66	0.78
1:C:543:VAL:HG23	1:C:554:GLN:HB3	1.66	0.78
1:D:543:VAL:HG23	1:D:554:GLN:HB3	1.66	0.78
1:G:543:VAL:HG23	1:G:554:GLN:HB3	1.66	0.78
1:A:543:VAL:HG23	1:A:554:GLN:HB3	1.66	0.77
1:F:543:VAL:HG23	1:F:554:GLN:HB3	1.66	0.77
1:H:543:VAL:HG23	1:H:554:GLN:HB3	1.66	0.77
1:E:543:VAL:HG23	1:E:554:GLN:HB3	1.66	0.77
1:F:196:SER:OG	1:F:481:GLN:NE2	2.16	0.74

Continued on next page...

For Manuscript Review

Continued from previous page...

Atom-1	Atom-2	Interatomic distance (Å)	Clash overlap (Å)
1:G:196:SER:OG	1:G:481:GLN:NE2	2.16	0.74
1:H:196:SER:OG	1:H:481:GLN:NE2	2.16	0.72
1:E:41:TYR:OH	1:E:323:PRO:O	2.09	0.71
1:H:41:TYR:OH	1:H:323:PRO:O	2.09	0.71
1:B:41:TYR:OH	1:B:323:PRO:O	2.09	0.71
1:D:68:THR:O	1:D:72:ASN:ND2	2.24	0.71
1:A:68:THR:O	1:A:72:ASN:ND2	2.24	0.70
1:C:41:TYR:OH	1:C:323:PRO:O	2.09	0.70
1:D:41:TYR:OH	1:D:323:PRO:O	2.09	0.70
1:A:41:TYR:OH	1:A:323:PRO:O	2.09	0.70
1:B:541:SER:O	1:B:546:GLN:NE2	2.25	0.70
1:F:68:THR:O	1:F:72:ASN:ND2	2.24	0.70
1:C:541:SER:O	1:C:546:GLN:NE2	2.25	0.69
1:E:541:SER:O	1:E:546:GLN:NE2	2.25	0.69
1:F:41:TYR:OH	1:F:323:PRO:O	2.09	0.69
1:G:68:THR:O	1:G:72:ASN:ND2	2.24	0.69
1:G:41:TYR:OH	1:G:323:PRO:O	2.09	0.69
1:H:541:SER:O	1:H:546:GLN:NE2	2.25	0.69
1:B:68:THR:O	1:B:72:ASN:ND2	2.24	0.69
1:E:68:THR:O	1:E:72:ASN:ND2	2.24	0.69
1:C:68:THR:O	1:C:72:ASN:ND2	2.24	0.69
1:D:541:SER:O	1:D:546:GLN:NE2	2.25	0.69
1:F:541:SER:O	1:F:546:GLN:NE2	2.25	0.69
1:H:68:THR:O	1:H:72:ASN:ND2	2.24	0.69
1:G:541:SER:O	1:G:546:GLN:NE2	2.25	0.69
1:A:541:SER:O	1:A:546:GLN:NE2	2.25	0.69
1:B:712:TYR:OH	1:B:736:GLU:OE2	2.08	0.67
1:E:39:GLN:O	1:E:58:LYS:NZ	2.28	0.67
1:H:39:GLN:O	1:H:58:LYS:NZ	2.28	0.66
1:B:39:GLN:O	1:B:58:LYS:NZ	2.28	0.66
1:C:730:ASN:ND2	1:C:760:GLU:OE2	2.29	0.66
1:D:712:TYR:OH	1:D:736:GLU:OE2	2.08	0.66
1:A:712:TYR:OH	1:A:736:GLU:OE2	2.08	0.66
1:B:730:ASN:ND2	1:B:760:GLU:OE2	2.29	0.66
1:C:39:GLN:O	1:C:58:LYS:NZ	2.28	0.66
1:D:39:GLN:O	1:D:58:LYS:NZ	2.28	0.66
1:A:39:GLN:O	1:A:58:LYS:NZ	2.28	0.66
1:F:39:GLN:O	1:F:58:LYS:NZ	2.28	0.65
1:D:730:ASN:ND2	1:D:760:GLU:OE2	2.29	0.65
1:E:730:ASN:ND2	1:E:760:GLU:OE2	2.29	0.65
1:G:39:GLN:O	1:G:58:LYS:NZ	2.28	0.65

Continued on next page...

For Manuscript Review

Continued from previous page...

Atom-1	Atom-2	Interatomic distance (Å)	Clash overlap (Å)
1:A:730:ASN:ND2	1:A:760:GLU:OE2	2.29	0.65
1:G:730:ASN:ND2	1:G:760:GLU:OE2	2.29	0.65
1:H:730:ASN:ND2	1:H:760:GLU:OE2	2.29	0.65
1:F:730:ASN:ND2	1:F:760:GLU:OE2	2.29	0.65
1:C:712:TYR:OH	1:C:736:GLU:OE2	2.08	0.64
1:E:330:THR:OG1	1:E:333:HIS:ND1	2.28	0.64
1:H:330:THR:OG1	1:H:333:HIS:ND1	2.28	0.64
1:B:330:THR:OG1	1:B:333:HIS:ND1	2.29	0.64
1:G:330:THR:OG1	1:G:333:HIS:ND1	2.28	0.64
1:G:560:SER:OG	1:H:560:SER:OG	2.16	0.64
1:G:646:ALA:HB2	1:G:655:ILE:HG13	1.80	0.64
1:F:646:ALA:HB2	1:F:655:ILE:HG13	1.80	0.64
1:E:560:SER:OG	1:F:560:SER:OG	2.16	0.64
1:F:330:THR:OG1	1:F:333:HIS:ND1	2.28	0.64
1:A:646:ALA:HB2	1:A:655:ILE:HG13	1.80	0.64
1:D:646:ALA:HB2	1:D:655:ILE:HG13	1.80	0.64
1:A:330:THR:OG1	1:A:333:HIS:ND1	2.28	0.64
1:C:560:SER:OG	1:D:560:SER:OG	2.16	0.63
1:D:330:THR:OG1	1:D:333:HIS:ND1	2.28	0.63
1:H:712:TYR:OH	1:H:736:GLU:OE2	2.08	0.63
1:A:560:SER:OG	1:B:560:SER:OG	2.16	0.63
1:E:646:ALA:HB2	1:E:655:ILE:HG13	1.80	0.63
1:F:735:GLU:OE1	1:F:751:ARG:NH2	2.32	0.63
1:G:735:GLU:OE1	1:G:751:ARG:NH2	2.32	0.63
1:H:196:SER:HG	1:H:481:GLN:HE22	1.45	0.63
1:E:712:TYR:OH	1:E:736:GLU:OE2	2.08	0.62
1:H:646:ALA:HB2	1:H:655:ILE:HG13	1.80	0.62
1:D:735:GLU:OE1	1:D:751:ARG:NH2	2.32	0.62
1:E:735:GLU:OE1	1:E:751:ARG:NH2	2.32	0.62
1:A:735:GLU:OE1	1:A:751:ARG:NH2	2.32	0.62
1:H:221:ASN:OD1	1:H:222:PRO:HD2	1.99	0.62
1:H:735:GLU:OE1	1:H:751:ARG:NH2	2.32	0.62
1:E:221:ASN:OD1	1:E:222:PRO:HD2	1.99	0.62
1:C:221:ASN:OD1	1:C:222:PRO:HD2	1.99	0.62
1:C:646:ALA:HB2	1:C:655:ILE:HG13	1.80	0.62
1:E:196:SER:HG	1:E:481:GLN:HE22	1.46	0.62
1:A:221:ASN:OD1	1:A:222:PRO:HD2	1.99	0.62
1:B:221:ASN:OD1	1:B:222:PRO:HD2	1.99	0.62
1:B:646:ALA:HB2	1:B:655:ILE:HG13	1.80	0.62
1:D:221:ASN:OD1	1:D:222:PRO:HD2	1.99	0.62
1:F:221:ASN:OD1	1:F:222:PRO:HD2	1.99	0.62

Continued on next page...

For Manuscript Review

Continued from previous page...

Atom-1	Atom-2	Interatomic distance (Å)	Clash overlap (Å)
1:G:221:ASN:OD1	1:G:222:PRO:HD2	1.99	0.62
1:C:547:ASP:OD2	1:D:60:ARG:NH1	2.33	0.62
1:A:547:ASP:OD2	1:B:60:ARG:NH1	2.33	0.61
1:E:547:ASP:OD2	1:F:60:ARG:NH1	2.33	0.61
1:G:547:ASP:OD2	1:H:60:ARG:NH1	2.33	0.61
1:C:735:GLU:OE1	1:C:751:ARG:NH2	2.32	0.61
1:A:60:ARG:NH1	1:B:547:ASP:OD2	2.34	0.61
1:A:145:PRO:HG3	1:A:518:TRP:CE3	2.36	0.61
1:B:735:GLU:OE1	1:B:751:ARG:NH2	2.32	0.61
1:D:145:PRO:HG3	1:D:518:TRP:CE3	2.36	0.61
1:E:145:PRO:HG3	1:E:518:TRP:CE3	2.36	0.61
1:G:60:ARG:NH1	1:H:547:ASP:OD2	2.34	0.61
1:C:60:ARG:NH1	1:D:547:ASP:OD2	2.34	0.61
1:H:145:PRO:HG3	1:H:518:TRP:CE3	2.36	0.61
1:C:145:PRO:HG3	1:C:518:TRP:CE3	2.36	0.60
1:E:60:ARG:NH1	1:F:547:ASP:OD2	2.34	0.60
1:B:145:PRO:HG3	1:B:518:TRP:CE3	2.36	0.60
1:H:620:GLU:OE2	1:H:673:ASN:ND2	2.28	0.60
1:E:620:GLU:OE2	1:E:673:ASN:ND2	2.28	0.60
1:F:145:PRO:HG3	1:F:518:TRP:CE3	2.36	0.59
1:G:145:PRO:HG3	1:G:518:TRP:CE3	2.36	0.59
1:D:506:HIS:ND1	1:D:509:ASP:OD2	2.34	0.59
1:A:506:HIS:ND1	1:A:509:ASP:OD2	2.34	0.59
1:B:165:TYR:CD2	1:B:196:SER:HB2	2.38	0.59
1:C:165:TYR:CD2	1:C:196:SER:HB2	2.38	0.59
1:F:506:HIS:ND1	1:F:509:ASP:OD2	2.34	0.59
1:G:506:HIS:ND1	1:G:509:ASP:OD2	2.34	0.59
1:E:614:LEU:O	1:E:618:ARG:HG3	2.03	0.59
1:A:165:TYR:CD2	1:A:196:SER:HB2	2.38	0.59
1:B:506:HIS:ND1	1:B:509:ASP:OD2	2.34	0.59
1:E:547:ASP:CG	1:F:60:ARG:HH12	2.06	0.59
1:F:614:LEU:O	1:F:618:ARG:HG3	2.03	0.59
1:G:614:LEU:O	1:G:618:ARG:HG3	2.03	0.59
1:H:614:LEU:O	1:H:618:ARG:HG3	2.03	0.59
1:C:330:THR:OG1	1:C:333:HIS:ND1	2.28	0.59
1:C:506:HIS:ND1	1:C:509:ASP:OD2	2.34	0.59
1:C:547:ASP:CG	1:D:60:ARG:HH12	2.06	0.59
1:D:165:TYR:CD2	1:D:196:SER:HB2	2.38	0.59
1:F:165:TYR:CD2	1:F:196:SER:HB2	2.38	0.58
1:B:614:LEU:O	1:B:618:ARG:HG3	2.03	0.58
1:D:614:LEU:O	1:D:618:ARG:HG3	2.03	0.58

Continued on next page...

For Manuscript Review

Continued from previous page...

Atom-1	Atom-2	Interatomic distance (Å)	Clash overlap (Å)
1:G:60:ARG:HH12	1:H:547:ASP:CG	2.07	0.58
1:A:60:ARG:HH12	1:B:547:ASP:CG	2.07	0.58
1:A:614:LEU:O	1:A:618:ARG:HG3	2.03	0.58
1:G:165:TYR:CD2	1:G:196:SER:HB2	2.38	0.58
1:H:165:TYR:CD2	1:H:196:SER:HB2	2.38	0.58
1:A:547:ASP:CG	1:B:60:ARG:HH12	2.06	0.58
1:C:614:LEU:O	1:C:618:ARG:HG3	2.03	0.58
1:E:165:TYR:CD2	1:E:196:SER:HB2	2.38	0.58
1:C:60:ARG:HH12	1:D:547:ASP:CG	2.07	0.58
1:B:620:GLU:OE2	1:B:673:ASN:ND2	2.28	0.58
1:C:620:GLU:OE2	1:C:673:ASN:ND2	2.28	0.58
1:H:506:HIS:ND1	1:H:509:ASP:OD2	2.34	0.58
1:F:620:GLU:OE2	1:F:673:ASN:ND2	2.28	0.57
1:G:620:GLU:OE2	1:G:673:ASN:ND2	2.28	0.57
1:B:156:GLU:OE1	1:B:159:TYR:HE2	1.88	0.57
1:C:156:GLU:OE1	1:C:159:TYR:HE2	1.88	0.57
1:F:196:SER:HG	1:F:481:GLN:HE22	1.48	0.57
1:G:547:ASP:CG	1:H:60:ARG:HH12	2.06	0.57
1:E:506:HIS:ND1	1:E:509:ASP:OD2	2.34	0.57
1:G:196:SER:HG	1:G:481:GLN:HE22	1.48	0.57
1:E:60:ARG:HH12	1:F:547:ASP:CG	2.07	0.57
1:F:142:HIS:CD2	1:F:155:GLY:HA2	2.40	0.57
1:B:196:SER:HG	1:B:481:GLN:HE22	1.49	0.57
1:D:498:TRP:CE2	1:D:537:LEU:HD13	2.39	0.57
1:G:142:HIS:CD2	1:G:155:GLY:HA2	2.40	0.57
1:A:498:TRP:CE2	1:A:537:LEU:HD13	2.39	0.57
1:C:196:SER:HG	1:C:481:GLN:HE22	1.48	0.56
1:E:156:GLU:OE1	1:E:159:TYR:HE2	1.87	0.56
1:E:498:TRP:CE2	1:E:537:LEU:HD13	2.39	0.56
1:H:498:TRP:CE2	1:H:537:LEU:HD13	2.39	0.56
1:B:498:TRP:CE2	1:B:537:LEU:HD13	2.39	0.56
1:E:142:HIS:CD2	1:E:155:GLY:HA2	2.40	0.56
1:H:156:GLU:OE1	1:H:159:TYR:HE2	1.87	0.56
1:B:700:THR:O	1:B:725:ASN:ND2	2.38	0.56
1:C:170:ASP:CG	1:C:431:ARG:HH21	2.09	0.56
1:C:498:TRP:CE2	1:C:537:LEU:HD13	2.39	0.56
1:C:700:THR:O	1:C:725:ASN:ND2	2.38	0.56
1:E:700:THR:O	1:E:725:ASN:ND2	2.38	0.56
1:F:498:TRP:CE2	1:F:537:LEU:HD13	2.39	0.56
1:G:498:TRP:CE2	1:G:537:LEU:HD13	2.39	0.56
1:H:142:HIS:CD2	1:H:155:GLY:HA2	2.40	0.56

Continued on next page...

For Manuscript Review

Continued from previous page...

Atom-1	Atom-2	Interatomic distance (Å)	Clash overlap (Å)
1:A:170:ASP:CG	1:A:431:ARG:HH21	2.09	0.56
1:B:142:HIS:HD2	1:B:155:GLY:HA2	1.71	0.56
1:H:243:GLU:HG2	1:H:290:PHE:HB3	1.88	0.56
1:H:700:THR:O	1:H:725:ASN:ND2	2.38	0.56
1:A:156:GLU:OE1	1:A:159:TYR:HE2	1.88	0.56
1:B:170:ASP:CG	1:B:431:ARG:HH21	2.09	0.56
1:C:142:HIS:HD2	1:C:155:GLY:HA2	1.71	0.56
1:D:170:ASP:CG	1:D:431:ARG:HH21	2.09	0.56
1:D:700:THR:O	1:D:725:ASN:ND2	2.38	0.56
1:E:243:GLU:HG2	1:E:290:PHE:HB3	1.88	0.56
1:G:700:THR:O	1:G:725:ASN:ND2	2.38	0.56
1:A:700:THR:O	1:A:725:ASN:ND2	2.38	0.56
1:F:170:ASP:CG	1:F:431:ARG:HH21	2.09	0.56
1:F:700:THR:O	1:F:725:ASN:ND2	2.38	0.56
1:A:142:HIS:HD2	1:A:155:GLY:HA2	1.71	0.56
1:B:79:ASN:HD21	1:B:105:GLN:NE2	2.04	0.56
1:C:79:ASN:HD21	1:C:105:GLN:NE2	2.04	0.56
1:D:156:GLU:OE1	1:D:159:TYR:HE2	1.88	0.56
1:C:142:HIS:CD2	1:C:155:GLY:HA2	2.40	0.56
1:D:142:HIS:HD2	1:D:155:GLY:HA2	1.71	0.56
1:G:156:GLU:OE1	1:G:159:TYR:HE2	1.88	0.56
1:B:142:HIS:CD2	1:B:155:GLY:HA2	2.40	0.56
1:D:142:HIS:CD2	1:D:155:GLY:HA2	2.40	0.56
1:G:170:ASP:CG	1:G:431:ARG:HH21	2.09	0.56
1:A:142:HIS:CD2	1:A:155:GLY:HA2	2.40	0.56
1:E:79:ASN:HD21	1:E:105:GLN:NE2	2.04	0.56
1:F:156:GLU:OE1	1:F:159:TYR:HE2	1.88	0.56
1:F:243:GLU:HG2	1:F:290:PHE:HB3	1.88	0.56
1:G:243:GLU:HG2	1:G:290:PHE:HB3	1.88	0.56
1:H:79:ASN:HD21	1:H:105:GLN:NE2	2.04	0.56
1:A:66:GLY:O	1:A:300:LYS:HG3	2.06	0.55
1:D:243:GLU:HG2	1:D:290:PHE:HB3	1.88	0.55
1:A:243:GLU:HG2	1:A:290:PHE:HB3	1.88	0.55
1:D:66:GLY:O	1:D:300:LYS:HG3	2.06	0.55
1:E:66:GLY:O	1:E:300:LYS:HG3	2.06	0.55
1:F:66:GLY:O	1:F:300:LYS:HG3	2.06	0.55
1:H:66:GLY:O	1:H:300:LYS:HG3	2.06	0.55
1:G:66:GLY:O	1:G:300:LYS:HG3	2.06	0.55
1:A:196:SER:HG	1:A:481:GLN:HE22	1.49	0.55
1:A:79:ASN:HD21	1:A:105:GLN:NE2	2.04	0.55
1:D:79:ASN:HD21	1:D:105:GLN:NE2	2.04	0.55

Continued on next page...

For Manuscript Review

Continued from previous page...

Atom-1	Atom-2	Interatomic distance (Å)	Clash overlap (Å)
1:F:142:HIS:HD2	1:F:155:GLY:HA2	1.71	0.55
1:G:79:ASN:HD21	1:G:105:GLN:NE2	2.04	0.55
1:F:79:ASN:HD21	1:F:105:GLN:NE2	2.04	0.55
1:G:142:HIS:HD2	1:G:155:GLY:HA2	1.71	0.55
1:F:712:TYR:OH	1:F:736:GLU:OE2	2.08	0.55
1:G:704:PRO:HA	1:G:728:ASN:HB3	1.89	0.55
1:H:704:PRO:HA	1:H:728:ASN:HB3	1.89	0.55
1:B:66:GLY:O	1:B:300:LYS:HG3	2.06	0.55
1:B:243:GLU:HG2	1:B:290:PHE:HB3	1.88	0.55
1:C:243:GLU:HG2	1:C:290:PHE:HB3	1.88	0.55
1:E:704:PRO:HA	1:E:728:ASN:HB3	1.89	0.55
1:F:704:PRO:HA	1:F:728:ASN:HB3	1.89	0.55
1:C:66:GLY:O	1:C:300:LYS:HG3	2.06	0.54
1:E:775:ASP:O	1:E:779:LYS:HE2	2.07	0.54
1:H:775:ASP:O	1:H:779:LYS:HE2	2.07	0.54
1:A:704:PRO:HA	1:A:728:ASN:HB3	1.89	0.54
1:B:704:PRO:HA	1:B:728:ASN:HB3	1.89	0.54
1:C:735:GLU:O	1:D:568:HIS:NE2	2.36	0.54
1:D:704:PRO:HA	1:D:728:ASN:HB3	1.89	0.54
1:H:254:GLU:OE2	1:H:258:SER:OG	2.20	0.54
1:A:620:GLU:OE2	1:A:673:ASN:ND2	2.28	0.54
1:D:620:GLU:OE2	1:D:673:ASN:ND2	2.28	0.54
1:D:775:ASP:O	1:D:779:LYS:HE2	2.07	0.54
1:E:170:ASP:CG	1:E:431:ARG:HH21	2.09	0.54
1:G:712:TYR:OH	1:G:736:GLU:OE2	2.08	0.54
1:H:142:HIS:HD2	1:H:155:GLY:HA2	1.71	0.54
1:H:170:ASP:CG	1:H:431:ARG:HH21	2.09	0.54
1:A:775:ASP:O	1:A:779:LYS:HE2	2.07	0.54
1:C:704:PRO:HA	1:C:728:ASN:HB3	1.89	0.54
1:E:142:HIS:HD2	1:E:155:GLY:HA2	1.71	0.54
1:F:775:ASP:O	1:F:779:LYS:HE2	2.07	0.54
1:A:220:ALA:HA	1:A:317:TRP:CD2	2.43	0.54
1:D:220:ALA:HA	1:D:317:TRP:CD2	2.43	0.54
1:G:775:ASP:O	1:G:779:LYS:HE2	2.07	0.54
1:F:144:ALA:HA	1:F:153:GLU:OE1	2.08	0.54
1:F:220:ALA:HA	1:F:317:TRP:CD2	2.43	0.54
1:G:144:ALA:HA	1:G:153:GLU:OE1	2.08	0.54
1:G:220:ALA:HA	1:G:317:TRP:CD2	2.43	0.54
1:B:775:ASP:O	1:B:779:LYS:HE2	2.07	0.54
1:G:548:HIS:CE1	1:H:62:VAL:HA	2.43	0.54
1:E:548:HIS:CE1	1:F:62:VAL:HA	2.44	0.53

Continued on next page...

For Manuscript Review

Continued from previous page...

Atom-1	Atom-2	Interatomic distance (Å)	Clash overlap (Å)
1:B:220:ALA:HA	1:B:317:TRP:CD2	2.43	0.53
1:C:220:ALA:HA	1:C:317:TRP:CD2	2.43	0.53
1:C:775:ASP:O	1:C:779:LYS:HE2	2.07	0.53
1:D:196:SER:HG	1:D:481:GLN:HE22	1.49	0.53
1:D:219:ILE:HG12	1:D:320:HIS:CD2	2.44	0.53
1:A:219:ILE:HG12	1:A:320:HIS:CD2	2.44	0.53
1:D:144:ALA:HA	1:D:153:GLU:OE1	2.08	0.53
1:A:144:ALA:HA	1:A:153:GLU:OE1	2.08	0.53
1:A:548:HIS:CE1	1:B:62:VAL:HA	2.43	0.53
1:C:548:HIS:CE1	1:D:62:VAL:HA	2.43	0.53
1:H:220:ALA:HA	1:H:317:TRP:CD2	2.43	0.53
1:B:219:ILE:HG12	1:B:320:HIS:CD2	2.44	0.53
1:E:220:ALA:HA	1:E:317:TRP:CD2	2.43	0.53
1:C:219:ILE:HG12	1:C:320:HIS:CD2	2.44	0.53
1:H:144:ALA:HA	1:H:153:GLU:OE1	2.08	0.53
1:F:219:ILE:HG12	1:F:320:HIS:CD2	2.44	0.53
1:G:219:ILE:HG12	1:G:320:HIS:CD2	2.44	0.53
1:C:144:ALA:HA	1:C:153:GLU:OE1	2.08	0.53
1:E:144:ALA:HA	1:E:153:GLU:OE1	2.08	0.53
1:E:219:ILE:HG12	1:E:320:HIS:CD2	2.44	0.52
1:H:219:ILE:HG12	1:H:320:HIS:CD2	2.44	0.52
1:B:144:ALA:HA	1:B:153:GLU:OE1	2.08	0.52
1:A:299:PRO:HG2	1:A:302:TRP:CD1	2.45	0.52
1:F:299:PRO:HG2	1:F:302:TRP:CD1	2.45	0.52
1:G:299:PRO:HG2	1:G:302:TRP:CD1	2.45	0.52
1:H:299:PRO:HG2	1:H:302:TRP:CD1	2.45	0.52
1:D:299:PRO:HG2	1:D:302:TRP:CD1	2.45	0.52
1:E:299:PRO:HG2	1:E:302:TRP:CD1	2.45	0.52
1:G:62:VAL:HA	1:H:548:HIS:CE1	2.45	0.52
1:E:735:GLU:O	1:F:568:HIS:NE2	2.36	0.52
1:E:62:VAL:HA	1:F:548:HIS:CE1	2.45	0.52
1:A:62:VAL:HA	1:B:548:HIS:CE1	2.45	0.51
1:C:62:VAL:HA	1:D:548:HIS:CE1	2.45	0.51
1:G:568:HIS:NE2	1:H:735:GLU:O	2.38	0.51
1:B:299:PRO:HG2	1:B:302:TRP:CD1	2.45	0.51
1:B:460:SER:OG	1:B:461:ASP:N	2.44	0.51
1:C:460:SER:OG	1:C:461:ASP:N	2.44	0.51
1:C:299:PRO:HG2	1:C:302:TRP:CD1	2.45	0.51
1:C:184:GLU:HG2	1:C:190:LEU:HD13	1.93	0.51
1:B:184:GLU:HG2	1:B:190:LEU:HD13	1.93	0.51
1:D:655:ILE:HD13	1:D:757:LEU:HD13	1.93	0.51

Continued on next page...

For Manuscript Review

Continued from previous page...

Atom-1	Atom-2	Interatomic distance (Å)	Clash overlap (Å)
1:E:655:ILE:HD13	1:E:757:LEU:HD13	1.93	0.51
1:H:460:SER:OG	1:H:461:ASP:N	2.44	0.51
1:H:655:ILE:HD13	1:H:757:LEU:HD13	1.93	0.51
1:A:655:ILE:HD13	1:A:757:LEU:HD13	1.93	0.51
1:B:185:ALA:HA	1:B:190:LEU:HD22	1.93	0.51
1:C:185:ALA:HA	1:C:190:LEU:HD22	1.93	0.51
1:E:460:SER:OG	1:E:461:ASP:N	2.44	0.51
1:F:184:GLU:HG2	1:F:190:LEU:HD13	1.93	0.51
1:G:184:GLU:HG2	1:G:190:LEU:HD13	1.93	0.51
1:D:589:GLU:OE2	1:D:593:LYS:HD2	2.12	0.50
1:A:185:ALA:HA	1:A:190:LEU:HD22	1.93	0.50
1:A:589:GLU:OE2	1:A:593:LYS:HD2	2.12	0.50
1:D:185:ALA:HA	1:D:190:LEU:HD22	1.93	0.50
1:E:589:GLU:OE2	1:E:593:LYS:HD2	2.12	0.50
1:F:79:ASN:HD21	1:F:105:GLN:CD	2.15	0.50
1:G:79:ASN:HD21	1:G:105:GLN:CD	2.15	0.50
1:G:460:SER:OG	1:G:461:ASP:N	2.44	0.50
1:H:589:GLU:OE2	1:H:593:LYS:HD2	2.12	0.50
1:B:655:ILE:HD13	1:B:757:LEU:HD13	1.93	0.50
1:C:655:ILE:HD13	1:C:757:LEU:HD13	1.93	0.50
1:D:184:GLU:HG2	1:D:190:LEU:HD13	1.93	0.50
1:F:460:SER:OG	1:F:461:ASP:N	2.44	0.50
1:D:460:SER:OG	1:D:461:ASP:N	2.44	0.50
1:A:184:GLU:HG2	1:A:190:LEU:HD13	1.93	0.50
1:A:460:SER:OG	1:A:461:ASP:N	2.44	0.50
1:E:184:GLU:HG2	1:E:190:LEU:HD13	1.93	0.50
1:E:185:ALA:HA	1:E:190:LEU:HD22	1.93	0.50
1:F:199:LEU:O	1:F:453:GLN:NE2	2.41	0.50
1:A:79:ASN:HD21	1:A:105:GLN:CD	2.15	0.50
1:D:79:ASN:HD21	1:D:105:GLN:CD	2.15	0.50
1:F:185:ALA:HA	1:F:190:LEU:HD22	1.93	0.50
1:H:184:GLU:HG2	1:H:190:LEU:HD13	1.93	0.50
1:H:185:ALA:HA	1:H:190:LEU:HD22	1.93	0.50
1:G:185:ALA:HA	1:G:190:LEU:HD22	1.93	0.49
1:G:199:LEU:O	1:G:453:GLN:NE2	2.41	0.49
1:C:589:GLU:OE2	1:C:593:LYS:HD2	2.12	0.49
1:A:735:GLU:O	1:B:568:HIS:NE2	2.36	0.49
1:B:589:GLU:OE2	1:B:593:LYS:HD2	2.12	0.49
1:F:589:GLU:OE2	1:F:593:LYS:HD2	2.12	0.49
1:F:655:ILE:HD13	1:F:757:LEU:HD13	1.93	0.49
1:E:777:LEU:O	1:E:781:ARG:HG3	2.13	0.49

Continued on next page...

For Manuscript Review

Continued from previous page...

Atom-1	Atom-2	Interatomic distance (Å)	Clash overlap (Å)
1:H:777:LEU:O	1:H:781:ARG:HG3	2.13	0.49
1:G:589:GLU:OE2	1:G:593:LYS:HD2	2.12	0.49
1:G:655:ILE:HD13	1:G:757:LEU:HD13	1.93	0.49
1:B:777:LEU:O	1:B:781:ARG:HG3	2.13	0.49
1:C:777:LEU:O	1:C:781:ARG:HG3	2.13	0.49
1:C:568:HIS:NE2	1:D:735:GLU:O	2.38	0.49
1:E:79:ASN:HD21	1:E:105:GLN:CD	2.15	0.49
1:H:79:ASN:HD21	1:H:105:GLN:CD	2.15	0.49
1:B:79:ASN:HD21	1:B:105:GLN:CD	2.15	0.48
1:F:777:LEU:O	1:F:781:ARG:HG3	2.13	0.48
1:C:79:ASN:HD21	1:C:105:GLN:CD	2.15	0.48
1:G:777:LEU:O	1:G:781:ARG:HG3	2.13	0.48
1:A:568:HIS:NE2	1:B:735:GLU:O	2.38	0.48
1:C:330:THR:HG1	1:C:333:HIS:HD1	1.56	0.48
1:E:218:LYS:HD2	1:F:477:LEU:HB2	1.96	0.47
1:G:735:GLU:O	1:H:568:HIS:NE2	2.36	0.47
1:A:9:PRO:HB2	1:A:10:TRP:CD1	2.50	0.47
1:D:9:PRO:HB2	1:D:10:TRP:CD1	2.50	0.47
1:F:36:SER:HB3	1:F:68:THR:HG21	1.97	0.47
1:A:218:LYS:HD2	1:B:477:LEU:HB2	1.96	0.47
1:G:9:PRO:HB2	1:G:10:TRP:CD1	2.50	0.47
1:G:218:LYS:HD2	1:H:477:LEU:HB2	1.96	0.47
1:A:777:LEU:O	1:A:781:ARG:HG3	2.13	0.47
1:F:9:PRO:HB2	1:F:10:TRP:CD1	2.50	0.47
1:G:36:SER:HB3	1:G:68:THR:HG21	1.97	0.47
1:A:568:HIS:HE1	1:B:751:ARG:HH22	1.63	0.47
1:D:777:LEU:O	1:D:781:ARG:HG3	2.13	0.47
1:G:477:LEU:HB2	1:H:218:LYS:HD2	1.97	0.47
1:A:709:TYR:CE2	1:A:711:SER:HB3	2.50	0.47
1:C:9:PRO:HB2	1:C:10:TRP:CD1	2.50	0.47
1:E:9:PRO:HB2	1:E:10:TRP:CD1	2.50	0.47
1:E:568:HIS:HE1	1:F:751:ARG:HH22	1.63	0.47
1:E:709:TYR:CE2	1:E:711:SER:HB3	2.50	0.47
1:G:568:HIS:HE1	1:H:751:ARG:HH22	1.63	0.47
1:H:709:TYR:CE2	1:H:711:SER:HB3	2.50	0.47
1:D:709:TYR:CE2	1:D:711:SER:HB3	2.50	0.47
1:E:477:LEU:HB2	1:F:218:LYS:HD2	1.97	0.47
1:H:9:PRO:HB2	1:H:10:TRP:CD1	2.50	0.47
1:H:36:SER:HB3	1:H:68:THR:HG21	1.97	0.47
1:B:9:PRO:HB2	1:B:10:TRP:CD1	2.50	0.47
1:E:36:SER:HB3	1:E:68:THR:HG21	1.97	0.47

Continued on next page...

For Manuscript Review

Continued from previous page...

Atom-1	Atom-2	Interatomic distance (Å)	Clash overlap (Å)
1:B:134:SER:OG	1:B:141:SER:HB3	2.15	0.47
1:B:435:PRO:HA	1:B:476:GLN:O	2.15	0.47
1:C:134:SER:OG	1:C:141:SER:HB3	2.15	0.47
1:C:435:PRO:HA	1:C:476:GLN:O	2.15	0.47
1:A:134:SER:OG	1:A:141:SER:HB3	2.15	0.46
1:A:261:ARG:O	1:A:265:GLU:OE1	2.32	0.46
1:B:170:ASP:OD1	1:B:428:ARG:NH2	2.38	0.46
1:C:477:LEU:HB2	1:D:218:LYS:HD2	1.97	0.46
1:D:134:SER:OG	1:D:141:SER:HB3	2.15	0.46
1:E:394:TYR:OH	1:E:421:ASP:OD2	2.25	0.46
1:E:568:HIS:NE2	1:F:735:GLU:O	2.38	0.46
1:F:709:TYR:CE2	1:F:711:SER:HB3	2.50	0.46
1:G:709:TYR:CE2	1:G:711:SER:HB3	2.50	0.46
1:C:170:ASP:OD1	1:C:428:ARG:NH2	2.38	0.46
1:E:134:SER:OG	1:E:141:SER:HB3	2.15	0.46
1:H:435:PRO:HA	1:H:476:GLN:O	2.15	0.46
1:C:218:LYS:HD2	1:D:477:LEU:HB2	1.96	0.46
1:D:36:SER:HB3	1:D:68:THR:HG21	1.97	0.46
1:E:435:PRO:HA	1:E:476:GLN:O	2.15	0.46
1:H:134:SER:OG	1:H:141:SER:HB3	2.15	0.46
1:A:36:SER:HB3	1:A:68:THR:HG21	1.97	0.46
1:C:301:GLY:O	1:C:314:GLU:HG3	2.16	0.46
1:C:568:HIS:HE1	1:D:751:ARG:HH22	1.63	0.46
1:C:709:TYR:CE2	1:C:711:SER:HB3	2.50	0.46
1:H:394:TYR:OH	1:H:421:ASP:OD2	2.25	0.46
1:A:435:PRO:HA	1:A:476:GLN:O	2.15	0.46
1:B:301:GLY:O	1:B:314:GLU:HG3	2.16	0.46
1:B:709:TYR:CE2	1:B:711:SER:HB3	2.50	0.46
1:D:435:PRO:HA	1:D:476:GLN:O	2.15	0.46
1:E:751:ARG:HH22	1:F:568:HIS:HE1	1.64	0.46
1:A:238:HIS:HD1	1:B:235:GLU:CD	2.19	0.46
1:B:219:ILE:HG12	1:B:320:HIS:NE2	2.31	0.46
1:C:219:ILE:HG12	1:C:320:HIS:NE2	2.31	0.46
1:G:301:GLY:O	1:G:314:GLU:HG3	2.16	0.46
1:A:68:THR:HG22	1:A:72:ASN:HD21	1.81	0.46
1:A:301:GLY:O	1:A:314:GLU:HG3	2.16	0.46
1:B:542:HIS:CE1	1:B:544:TRP:HB2	2.51	0.46
1:C:235:GLU:CD	1:D:238:HIS:HD1	2.20	0.46
1:D:301:GLY:O	1:D:314:GLU:HG3	2.16	0.46
1:E:301:GLY:O	1:E:314:GLU:HG3	2.16	0.46
1:F:134:SER:OG	1:F:141:SER:HB3	2.15	0.46

Continued on next page...

For Manuscript Review

Continued from previous page...

Atom-1	Atom-2	Interatomic distance (Å)	Clash overlap (Å)
1:F:219:ILE:HG12	1:F:320:HIS:NE2	2.31	0.46
1:G:219:ILE:HG12	1:G:320:HIS:NE2	2.31	0.46
1:H:199:LEU:O	1:H:453:GLN:NE2	2.41	0.46
1:H:301:GLY:O	1:H:314:GLU:HG3	2.16	0.46
1:A:477:LEU:HB2	1:B:218:LYS:HD2	1.97	0.46
1:B:649:ASP:OD1	1:B:649:ASP:N	2.49	0.46
1:C:542:HIS:CE1	1:C:544:TRP:HB2	2.51	0.46
1:C:649:ASP:N	1:C:649:ASP:OD1	2.49	0.46
1:C:751:ARG:HH22	1:D:568:HIS:HE1	1.64	0.46
1:D:68:THR:HG22	1:D:72:ASN:HD21	1.81	0.46
1:F:301:GLY:O	1:F:314:GLU:HG3	2.16	0.46
1:G:134:SER:OG	1:G:141:SER:HB3	2.15	0.46
1:G:238:HIS:HD1	1:H:235:GLU:CD	2.19	0.46
1:A:686:ASN:OD1	1:A:688:GLU:HG3	2.17	0.45
1:B:36:SER:HB3	1:B:68:THR:HG21	1.97	0.45
1:B:65:TRP:HE1	1:B:304:CYS:HB3	1.81	0.45
1:C:36:SER:HB3	1:C:68:THR:HG21	1.97	0.45
1:D:686:ASN:OD1	1:D:688:GLU:HG3	2.17	0.45
1:E:199:LEU:O	1:E:453:GLN:NE2	2.41	0.45
1:G:751:ARG:HH22	1:H:568:HIS:HE1	1.64	0.45
1:H:65:TRP:HE1	1:H:304:CYS:HB3	1.81	0.45
1:A:542:HIS:CE1	1:A:544:TRP:HB2	2.51	0.45
1:C:65:TRP:HE1	1:C:304:CYS:HB3	1.81	0.45
1:E:65:TRP:HE1	1:E:304:CYS:HB3	1.81	0.45
1:E:238:HIS:HD1	1:F:235:GLU:CD	2.19	0.45
1:E:542:HIS:CE1	1:E:544:TRP:HB2	2.51	0.45
1:H:542:HIS:CE1	1:H:544:TRP:HB2	2.51	0.45
1:A:401:GLU:HG2	1:A:402:TYR:CD1	2.52	0.45
1:A:751:ARG:HH22	1:B:568:HIS:HE1	1.64	0.45
1:E:219:ILE:HG12	1:E:320:HIS:NE2	2.31	0.45
1:F:65:TRP:HE1	1:F:304:CYS:HB3	1.81	0.45
1:H:219:ILE:HG12	1:H:320:HIS:NE2	2.31	0.45
1:D:401:GLU:HG2	1:D:402:TYR:CD1	2.52	0.45
1:D:542:HIS:CE1	1:D:544:TRP:HB2	2.51	0.45
1:E:235:GLU:CD	1:F:238:HIS:HD1	2.20	0.45
1:F:68:THR:HG22	1:F:72:ASN:HD21	1.81	0.45
1:F:435:PRO:HA	1:F:476:GLN:O	2.15	0.45
1:G:68:THR:HG22	1:G:72:ASN:HD21	1.81	0.45
1:G:435:PRO:HA	1:G:476:GLN:O	2.15	0.45
1:D:65:TRP:HE1	1:D:304:CYS:HB3	1.81	0.45
1:D:170:ASP:OD1	1:D:428:ARG:NH2	2.38	0.45

Continued on next page...

For Manuscript Review

Continued from previous page...

Atom-1	Atom-2	Interatomic distance (Å)	Clash overlap (Å)
1:G:65:TRP:HE1	1:G:304:CYS:HB3	1.81	0.45
1:B:89:THR:HA	1:B:175:PHE:O	2.17	0.45
1:B:686:ASN:OD1	1:B:688:GLU:HG3	2.16	0.45
1:D:47:LEU:HD12	1:D:49:LYS:NZ	2.32	0.45
1:E:401:GLU:HG2	1:E:402:TYR:CD1	2.52	0.45
1:A:47:LEU:HD12	1:A:49:LYS:NZ	2.32	0.45
1:A:65:TRP:HE1	1:A:304:CYS:HB3	1.81	0.45
1:A:219:ILE:HG12	1:A:320:HIS:NE2	2.31	0.45
1:C:89:THR:HA	1:C:175:PHE:O	2.17	0.45
1:C:686:ASN:OD1	1:C:688:GLU:HG3	2.17	0.45
1:D:219:ILE:HG12	1:D:320:HIS:NE2	2.31	0.45
1:F:542:HIS:CE1	1:F:544:TRP:HB2	2.51	0.45
1:G:235:GLU:CD	1:H:238:HIS:HD1	2.20	0.45
1:H:401:GLU:HG2	1:H:402:TYR:CD1	2.52	0.45
1:A:235:GLU:CD	1:B:238:HIS:HD1	2.20	0.45
1:E:68:THR:HG22	1:E:72:ASN:HD21	1.81	0.45
1:H:68:THR:HG22	1:H:72:ASN:HD21	1.81	0.45
1:A:170:ASP:OD1	1:A:428:ARG:NH2	2.38	0.45
1:C:238:HIS:HD1	1:D:235:GLU:CD	2.19	0.45
1:E:182:ASP:HB3	1:E:214:LEU:HA	1.99	0.45
1:E:686:ASN:OD1	1:E:688:GLU:HG3	2.16	0.45
1:G:47:LEU:HD12	1:G:49:LYS:NZ	2.32	0.45
1:G:89:THR:HA	1:G:175:PHE:O	2.17	0.45
1:G:542:HIS:CE1	1:G:544:TRP:HB2	2.51	0.45
1:A:281:ALA:HA	1:A:284:ASP:O	2.17	0.45
1:B:199:LEU:O	1:B:453:GLN:NE2	2.41	0.45
1:E:47:LEU:HD12	1:E:49:LYS:NZ	2.32	0.45
1:F:47:LEU:HD12	1:F:49:LYS:NZ	2.32	0.45
1:F:89:THR:HA	1:F:175:PHE:O	2.17	0.45
1:G:686:ASN:OD1	1:G:688:GLU:HG3	2.17	0.45
1:H:47:LEU:HD12	1:H:49:LYS:NZ	2.32	0.45
1:H:182:ASP:HB3	1:H:214:LEU:HA	1.99	0.45
1:B:47:LEU:HD12	1:B:49:LYS:NZ	2.32	0.44
1:B:68:THR:HG22	1:B:72:ASN:HD21	1.81	0.44
1:B:182:ASP:HB3	1:B:214:LEU:HA	1.99	0.44
1:C:182:ASP:HB3	1:C:214:LEU:HA	1.99	0.44
1:D:281:ALA:HA	1:D:284:ASP:O	2.18	0.44
1:E:649:ASP:OD1	1:E:649:ASP:N	2.49	0.44
1:F:686:ASN:OD1	1:F:688:GLU:HG3	2.17	0.44
1:G:394:TYR:OH	1:G:421:ASP:OD2	2.25	0.44
1:H:686:ASN:OD1	1:H:688:GLU:HG3	2.17	0.44

Continued on next page...

For Manuscript Review

Continued from previous page...

Atom-1	Atom-2	Interatomic distance (Å)	Clash overlap (Å)
1:B:401:GLU:HG2	1:B:402:TYR:CD1	2.52	0.44
1:C:47:LEU:HD12	1:C:49:LYS:NZ	2.32	0.44
1:C:68:THR:HG22	1:C:72:ASN:HD21	1.81	0.44
1:H:649:ASP:N	1:H:649:ASP:OD1	2.49	0.44
1:C:401:GLU:HG2	1:C:402:TYR:CD1	2.52	0.44
1:F:170:ASP:OD1	1:F:428:ARG:NH2	2.38	0.44
1:F:182:ASP:HB3	1:F:214:LEU:HA	1.99	0.44
1:G:182:ASP:HB3	1:G:214:LEU:HA	1.99	0.44
1:C:199:LEU:O	1:C:453:GLN:NE2	2.41	0.44
1:F:401:GLU:HG2	1:F:402:TYR:CD1	2.52	0.44
1:G:401:GLU:HG2	1:G:402:TYR:CD1	2.52	0.44
1:D:378:ASN:O	1:D:381:VAL:HG22	2.17	0.44
1:F:281:ALA:HA	1:F:284:ASP:O	2.18	0.44
1:F:378:ASN:O	1:F:381:VAL:HG22	2.18	0.44
1:F:394:TYR:OH	1:F:421:ASP:OD2	2.25	0.44
1:G:378:ASN:O	1:G:381:VAL:HG22	2.18	0.44
1:A:89:THR:HA	1:A:175:PHE:O	2.17	0.44
1:A:378:ASN:O	1:A:381:VAL:HG22	2.18	0.44
1:B:281:ALA:HA	1:B:284:ASP:O	2.18	0.44
1:D:89:THR:HA	1:D:175:PHE:O	2.17	0.44
1:D:199:LEU:O	1:D:453:GLN:NE2	2.41	0.44
1:G:281:ALA:HA	1:G:284:ASP:O	2.18	0.44
1:H:378:ASN:O	1:H:381:VAL:HG22	2.17	0.44
1:B:378:ASN:O	1:B:381:VAL:HG22	2.17	0.44
1:C:252:ASP:OD1	1:C:252:ASP:N	2.50	0.44
1:C:281:ALA:HA	1:C:284:ASP:O	2.17	0.44
1:C:378:ASN:O	1:C:381:VAL:HG22	2.18	0.44
1:E:378:ASN:O	1:E:381:VAL:HG22	2.17	0.44
1:B:252:ASP:N	1:B:252:ASP:OD1	2.50	0.44
1:G:170:ASP:OD1	1:G:428:ARG:NH2	2.38	0.44
1:G:651:PRO:O	1:G:655:ILE:HG12	2.18	0.44
1:H:544:TRP:HE3	1:H:746:MET:HG3	1.83	0.44
1:A:182:ASP:HB3	1:A:214:LEU:HA	1.99	0.43
1:D:182:ASP:HB3	1:D:214:LEU:HA	1.99	0.43
1:D:252:ASP:N	1:D:252:ASP:OD1	2.50	0.43
1:D:624:GLY:O	1:D:675:ALA:N	2.51	0.43
1:E:544:TRP:HE3	1:E:746:MET:HG3	1.83	0.43
1:A:199:LEU:O	1:A:453:GLN:NE2	2.41	0.43
1:A:252:ASP:OD1	1:A:252:ASP:N	2.50	0.43
1:A:624:GLY:O	1:A:675:ALA:N	2.52	0.43
1:A:649:ASP:N	1:A:649:ASP:OD1	2.49	0.43

Continued on next page...

For Manuscript Review

Continued from previous page...

Atom-1	Atom-2	Interatomic distance (Å)	Clash overlap (Å)
1:B:544:TRP:HE3	1:B:746:MET:HG3	1.83	0.43
1:C:544:TRP:HE3	1:C:746:MET:HG3	1.83	0.43
1:D:706:LEU:HD21	1:D:757:LEU:HD22	2.01	0.43
1:F:651:PRO:O	1:F:655:ILE:HG12	2.18	0.43
1:A:706:LEU:HD21	1:A:757:LEU:HD22	2.01	0.43
1:D:165:TYR:OH	1:D:242:TYR:OH	2.29	0.43
1:D:649:ASP:OD1	1:D:649:ASP:N	2.49	0.43
1:F:544:TRP:HE3	1:F:746:MET:HG3	1.83	0.43
1:D:651:PRO:O	1:D:655:ILE:HG12	2.18	0.43
1:G:544:TRP:HE3	1:G:746:MET:HG3	1.83	0.43
1:A:638:ASP:O	1:A:703:LYS:NZ	2.52	0.43
1:A:651:PRO:O	1:A:655:ILE:HG12	2.18	0.43
1:B:30:ARG:NH2	1:B:348:GLU:OE1	2.37	0.43
1:D:638:ASP:O	1:D:703:LYS:NZ	2.52	0.43
1:F:649:ASP:N	1:F:649:ASP:OD1	2.49	0.43
1:G:649:ASP:OD1	1:G:649:ASP:N	2.49	0.43
1:E:89:THR:HA	1:E:175:PHE:O	2.17	0.43
1:F:624:GLY:O	1:F:675:ALA:N	2.52	0.43
1:F:706:LEU:HD21	1:F:757:LEU:HD22	2.01	0.43
1:H:21:ALA:O	1:H:25:VAL:HG23	2.19	0.43
1:H:89:THR:HA	1:H:175:PHE:O	2.17	0.43
1:H:168:ILE:HD13	1:H:208:VAL:HG23	2.01	0.43
1:A:165:TYR:OH	1:A:242:TYR:OH	2.29	0.43
1:D:544:TRP:HE3	1:D:746:MET:HG3	1.83	0.43
1:E:742:THR:HG23	1:F:136:PRO:HG3	2.01	0.43
1:G:624:GLY:O	1:G:675:ALA:N	2.51	0.43
1:G:706:LEU:HD21	1:G:757:LEU:HD22	2.01	0.43
1:G:742:THR:HG23	1:H:136:PRO:HG3	2.01	0.43
1:H:281:ALA:HA	1:H:284:ASP:O	2.18	0.43
1:B:21:ALA:O	1:B:25:VAL:HG23	2.19	0.43
1:C:459:ILE:HG13	1:C:460:SER:N	2.34	0.43
1:E:21:ALA:O	1:E:25:VAL:HG23	2.19	0.43
1:A:544:TRP:HE3	1:A:746:MET:HG3	1.83	0.43
1:B:459:ILE:HG13	1:B:460:SER:N	2.34	0.43
1:C:21:ALA:O	1:C:25:VAL:HG23	2.19	0.43
1:C:30:ARG:NH2	1:C:348:GLU:OE1	2.37	0.43
1:E:168:ILE:HD13	1:E:208:VAL:HG23	2.01	0.43
1:E:281:ALA:HA	1:E:284:ASP:O	2.18	0.43
1:H:252:ASP:OD1	1:H:252:ASP:N	2.50	0.43
1:H:706:LEU:HD21	1:H:757:LEU:HD22	2.01	0.43
1:E:706:LEU:HD21	1:E:757:LEU:HD22	2.01	0.43

Continued on next page...

For Manuscript Review

Continued from previous page...

Atom-1	Atom-2	Interatomic distance (Å)	Clash overlap (Å)
1:F:168:ILE:HD13	1:F:208:VAL:HG23	2.01	0.43
1:A:772:ASP:OD1	1:A:773:LYS:N	2.52	0.42
1:C:706:LEU:HD21	1:C:757:LEU:HD22	2.01	0.42
1:E:252:ASP:OD1	1:E:252:ASP:N	2.50	0.42
1:H:772:ASP:OD1	1:H:773:LYS:N	2.52	0.42
1:D:21:ALA:O	1:D:25:VAL:HG23	2.19	0.42
1:D:772:ASP:OD1	1:D:773:LYS:N	2.52	0.42
1:E:772:ASP:OD1	1:E:773:LYS:N	2.52	0.42
1:F:21:ALA:O	1:F:25:VAL:HG23	2.19	0.42
1:G:168:ILE:HD13	1:G:208:VAL:HG23	2.01	0.42
1:H:250:GLY:HA3	1:H:302:TRP:NE1	2.34	0.42
1:A:634:ALA:HB1	1:A:639:GLU:HB2	2.02	0.42
1:B:168:ILE:HD13	1:B:208:VAL:HG23	2.01	0.42
1:C:250:GLY:HA3	1:C:302:TRP:NE1	2.34	0.42
1:E:250:GLY:HA3	1:E:302:TRP:NE1	2.35	0.42
1:G:21:ALA:O	1:G:25:VAL:HG23	2.19	0.42
1:H:651:PRO:O	1:H:655:ILE:HG12	2.18	0.42
1:A:168:ILE:HD13	1:A:208:VAL:HG23	2.01	0.42
1:B:250:GLY:HA3	1:B:302:TRP:NE1	2.34	0.42
1:B:706:LEU:HD21	1:B:757:LEU:HD22	2.01	0.42
1:B:772:ASP:OD1	1:B:773:LYS:N	2.52	0.42
1:C:772:ASP:OD1	1:C:773:LYS:N	2.52	0.42
1:D:168:ILE:HD13	1:D:208:VAL:HG23	2.01	0.42
1:D:634:ALA:HB1	1:D:639:GLU:HB2	2.02	0.42
1:E:651:PRO:O	1:E:655:ILE:HG12	2.18	0.42
1:A:21:ALA:O	1:A:25:VAL:HG23	2.19	0.42
1:B:431:ARG:HH11	1:B:474:VAL:HG23	1.85	0.42
1:B:700:THR:HB	1:B:703:LYS:HB2	2.01	0.42
1:D:459:ILE:HG13	1:D:460:SER:N	2.34	0.42
1:G:30:ARG:NH2	1:G:348:GLU:OE1	2.37	0.42
1:G:638:ASP:O	1:G:703:LYS:NZ	2.52	0.42
1:H:247:PHE:HB2	1:H:266:LEU:CD2	2.50	0.42
1:A:742:THR:HG23	1:B:136:PRO:HG3	2.01	0.42
1:C:168:ILE:HD13	1:C:208:VAL:HG23	2.01	0.42
1:C:431:ARG:HH11	1:C:474:VAL:HG23	1.85	0.42
1:C:651:PRO:O	1:C:655:ILE:HG12	2.18	0.42
1:C:700:THR:HB	1:C:703:LYS:HB2	2.01	0.42
1:F:638:ASP:O	1:F:703:LYS:NZ	2.52	0.42
1:F:700:THR:HB	1:F:703:LYS:HB2	2.01	0.42
1:A:431:ARG:HH11	1:A:474:VAL:HG23	1.85	0.42
1:D:431:ARG:HH11	1:D:474:VAL:HG23	1.85	0.42

Continued on next page...

For Manuscript Review

Continued from previous page...

Atom-1	Atom-2	Interatomic distance (Å)	Clash overlap (Å)
1:G:700:THR:HB	1:G:703:LYS:HB2	2.01	0.42
1:H:459:ILE:HG13	1:H:460:SER:N	2.34	0.42
1:A:459:ILE:HG13	1:A:460:SER:N	2.34	0.42
1:A:706:LEU:HD13	1:A:760:GLU:HG2	2.02	0.42
1:B:651:PRO:O	1:B:655:ILE:HG12	2.18	0.42
1:C:742:THR:HG23	1:D:136:PRO:HG3	2.01	0.42
1:D:706:LEU:HD13	1:D:760:GLU:HG2	2.02	0.42
1:E:459:ILE:HG13	1:E:460:SER:N	2.34	0.42
1:F:252:ASP:OD1	1:F:252:ASP:N	2.50	0.42
1:F:459:ILE:HG13	1:F:460:SER:N	2.34	0.42
1:F:634:ALA:HB1	1:F:639:GLU:HB2	2.02	0.42
1:G:459:ILE:HG13	1:G:460:SER:N	2.34	0.42
1:F:247:PHE:HB2	1:F:266:LEU:CD2	2.50	0.42
1:F:250:GLY:HA3	1:F:302:TRP:NE1	2.34	0.42
1:G:250:GLY:HA3	1:G:302:TRP:NE1	2.35	0.42
1:G:634:ALA:HB1	1:G:639:GLU:HB2	2.02	0.42
1:D:250:GLY:HA3	1:D:302:TRP:NE1	2.34	0.42
1:E:90:VAL:HG23	1:E:174:LEU:HD11	2.02	0.42
1:G:90:VAL:HG23	1:G:174:LEU:HD11	2.02	0.42
1:H:634:ALA:HB1	1:H:639:GLU:HB2	2.02	0.42
1:A:90:VAL:HG23	1:A:174:LEU:HD11	2.02	0.41
1:A:250:GLY:HA3	1:A:302:TRP:NE1	2.34	0.41
1:D:90:VAL:HG23	1:D:174:LEU:HD11	2.02	0.41
1:D:394:TYR:OH	1:D:421:ASP:OD2	2.25	0.41
1:E:634:ALA:HB1	1:E:639:GLU:HB2	2.02	0.41
1:F:90:VAL:HG23	1:F:174:LEU:HD11	2.02	0.41
1:F:772:ASP:OD1	1:F:773:LYS:N	2.52	0.41
1:H:90:VAL:HG23	1:H:174:LEU:HD11	2.02	0.41
1:B:633:THR:OG1	1:B:668:LYS:HA	2.20	0.41
1:D:247:PHE:HB2	1:D:266:LEU:CD2	2.49	0.41
1:F:30:ARG:NH2	1:F:348:GLU:OE1	2.37	0.41
1:G:252:ASP:N	1:G:252:ASP:OD1	2.50	0.41
1:B:624:GLY:O	1:B:675:ALA:N	2.51	0.41
1:C:633:THR:OG1	1:C:668:LYS:HA	2.20	0.41
1:E:700:THR:HB	1:E:703:LYS:HB2	2.01	0.41
1:F:725:ASN:OD1	1:F:728:ASN:HB2	2.21	0.41
1:G:431:ARG:HH11	1:G:474:VAL:HG23	1.85	0.41
1:G:772:ASP:OD1	1:G:773:LYS:N	2.52	0.41
1:H:700:THR:HB	1:H:703:LYS:HB2	2.01	0.41
1:B:634:ALA:HB1	1:B:639:GLU:HB2	2.02	0.41
1:C:706:LEU:HD13	1:C:760:GLU:HG2	2.02	0.41

Continued on next page...

For Manuscript Review

Continued from previous page...

Atom-1	Atom-2	Interatomic distance (Å)	Clash overlap (Å)
1:D:725:ASN:OD1	1:D:728:ASN:HB2	2.21	0.41
1:E:638:ASP:O	1:E:703:LYS:NZ	2.52	0.41
1:E:725:ASN:OD1	1:E:728:ASN:HB2	2.21	0.41
1:A:633:THR:OG1	1:A:668:LYS:HA	2.20	0.41
1:B:706:LEU:HD13	1:B:760:GLU:HG2	2.02	0.41
1:C:634:ALA:HB1	1:C:639:GLU:HB2	2.02	0.41
1:D:633:THR:OG1	1:D:668:LYS:HA	2.20	0.41
1:E:431:ARG:HH11	1:E:474:VAL:HG23	1.85	0.41
1:E:524:ARG:HB2	1:E:525:GLU:OE1	2.21	0.41
1:F:431:ARG:HH11	1:F:474:VAL:HG23	1.85	0.41
1:F:576:TYR:HB3	1:F:587:ILE:HD13	2.02	0.41
1:G:725:ASN:OD1	1:G:728:ASN:HB2	2.21	0.41
1:A:542:HIS:HE1	1:A:544:TRP:HB2	1.86	0.41
1:A:612:LEU:HD11	1:A:628:TRP:CZ2	2.56	0.41
1:A:725:ASN:OD1	1:A:728:ASN:HB2	2.21	0.41
1:C:624:GLY:O	1:C:675:ALA:N	2.52	0.41
1:G:247:PHE:HB2	1:G:266:LEU:CD2	2.50	0.41
1:G:576:TYR:HB3	1:G:587:ILE:HD13	2.02	0.41
1:H:431:ARG:HH11	1:H:474:VAL:HG23	1.85	0.41
1:H:524:ARG:HB2	1:H:525:GLU:OE1	2.21	0.41
1:B:247:PHE:HB2	1:B:266:LEU:CD2	2.50	0.41
1:B:725:ASN:OD1	1:B:728:ASN:HB2	2.21	0.41
1:C:524:ARG:HB2	1:C:525:GLU:OE1	2.21	0.41
1:C:725:ASN:OD1	1:C:728:ASN:HB2	2.21	0.41
1:D:391:LEU:HD23	1:D:614:LEU:HD21	2.03	0.41
1:D:612:LEU:HD11	1:D:628:TRP:CZ2	2.56	0.41
1:E:247:PHE:HB2	1:E:266:LEU:CD2	2.51	0.41
1:E:542:HIS:HE1	1:E:544:TRP:HB2	1.86	0.41
1:G:136:PRO:HG3	1:H:742:THR:HG23	2.03	0.41
1:G:389:PRO:HG2	1:G:418:TYR:CE1	2.56	0.41
1:H:725:ASN:OD1	1:H:728:ASN:HB2	2.21	0.41
1:A:391:LEU:HD23	1:A:614:LEU:HD21	2.03	0.41
1:B:524:ARG:HB2	1:B:525:GLU:OE1	2.21	0.41
1:C:391:LEU:HD23	1:C:614:LEU:HD21	2.03	0.41
1:D:542:HIS:HE1	1:D:544:TRP:HB2	1.86	0.41
1:E:136:PRO:HG3	1:F:742:THR:HG23	2.03	0.41
1:E:389:PRO:HG2	1:E:418:TYR:CE1	2.56	0.41
1:F:389:PRO:HG2	1:F:418:TYR:CE1	2.56	0.41
1:G:706:LEU:HD13	1:G:760:GLU:HG2	2.02	0.41
1:H:389:PRO:HG2	1:H:418:TYR:CE1	2.56	0.41
1:H:706:LEU:HD13	1:H:760:GLU:HG2	2.02	0.41

Continued on next page...

For Manuscript Review

Continued from previous page...

Atom-1	Atom-2	Interatomic distance (Å)	Clash overlap (Å)
1:A:42:LEU:O	1:A:58:LYS:HE3	2.21	0.41
1:A:389:PRO:HG2	1:A:418:TYR:CE1	2.56	0.41
1:B:391:LEU:HD23	1:B:614:LEU:HD21	2.03	0.41
1:B:502:GLU:HG3	1:B:539:VAL:HG11	2.03	0.41
1:C:612:LEU:HD11	1:C:628:TRP:CZ2	2.56	0.41
1:D:502:GLU:HG3	1:D:539:VAL:HG11	2.03	0.41
1:E:633:THR:OG1	1:E:668:LYS:HA	2.20	0.41
1:E:706:LEU:HD13	1:E:760:GLU:HG2	2.02	0.41
1:F:524:ARG:HB2	1:F:525:GLU:OE1	2.21	0.41
1:F:542:HIS:HE1	1:F:544:TRP:HB2	1.86	0.41
1:F:706:LEU:HD13	1:F:760:GLU:HG2	2.02	0.41
1:G:524:ARG:HB2	1:G:525:GLU:OE1	2.21	0.41
1:G:542:HIS:HE1	1:G:544:TRP:HB2	1.86	0.41
1:H:542:HIS:HE1	1:H:544:TRP:HB2	1.86	0.41
1:A:502:GLU:HG3	1:A:539:VAL:HG11	2.03	0.41
1:B:612:LEU:HD11	1:B:628:TRP:CZ2	2.56	0.41
1:C:502:GLU:HG3	1:C:539:VAL:HG11	2.03	0.41
1:D:42:LEU:O	1:D:58:LYS:HE3	2.21	0.41
1:D:389:PRO:HG2	1:D:418:TYR:CE1	2.56	0.41
1:D:700:THR:HB	1:D:703:LYS:HB2	2.01	0.41
1:E:576:TYR:HB3	1:E:587:ILE:HD13	2.03	0.41
1:G:397:LYS:HE2	1:G:397:LYS:HB2	1.95	0.41
1:H:47:LEU:HD11	1:H:354:GLY:HA2	2.03	0.41
1:H:576:TYR:HB3	1:H:587:ILE:HD13	2.03	0.41
1:H:633:THR:OG1	1:H:668:LYS:HA	2.20	0.41
1:E:47:LEU:HD11	1:E:354:GLY:HA2	2.03	0.40
1:F:391:LEU:HD23	1:F:614:LEU:HD21	2.03	0.40
1:F:633:THR:OG1	1:F:668:LYS:HA	2.20	0.40
1:G:612:LEU:HD11	1:G:628:TRP:CZ2	2.56	0.40
1:H:624:GLY:O	1:H:675:ALA:N	2.52	0.40
1:H:638:ASP:O	1:H:703:LYS:NZ	2.52	0.40
1:A:134:SER:HA	1:B:740:THR:OG1	2.22	0.40
1:C:90:VAL:HG23	1:C:174:LEU:HD11	2.02	0.40
1:C:638:ASP:O	1:C:703:LYS:NZ	2.52	0.40
1:E:624:GLY:O	1:E:675:ALA:N	2.52	0.40
1:F:397:LYS:HE2	1:F:397:LYS:HB2	1.95	0.40
1:F:612:LEU:HD11	1:F:628:TRP:CZ2	2.56	0.40
1:G:391:LEU:HD23	1:G:614:LEU:HD21	2.03	0.40
1:G:633:THR:OG1	1:G:668:LYS:HA	2.20	0.40
2:G:900:TPP:HN42	2:G:900:TPP:C2	2.35	0.40
1:A:700:THR:HB	1:A:703:LYS:HB2	2.01	0.40

Continued on next page...

For Manuscript Review

Continued from previous page...

Atom-1	Atom-2	Interatomic distance (Å)	Clash overlap (Å)
1:B:90:VAL:HG23	1:B:174:LEU:HD11	2.02	0.40
1:E:42:LEU:O	1:E:58:LYS:HE3	2.21	0.40
2:E:900:TPP:HN42	2:E:900:TPP:C2	2.35	0.40
2:F:900:TPP:HN42	2:F:900:TPP:C2	2.35	0.40
1:H:42:LEU:O	1:H:58:LYS:HE3	2.21	0.40
1:B:389:PRO:HG2	1:B:418:TYR:CE1	2.56	0.40
1:C:247:PHE:HB2	1:C:266:LEU:CD2	2.51	0.40
1:C:389:PRO:HG2	1:C:418:TYR:CE1	2.56	0.40
1:G:47:LEU:HD11	1:G:354:GLY:HA2	2.03	0.40
2:H:900:TPP:HN42	2:H:900:TPP:C2	2.35	0.40
1:A:351:ASP:OD1	1:A:355:ALA:N	2.55	0.40
1:B:638:ASP:O	1:B:703:LYS:NZ	2.52	0.40
1:D:351:ASP:OD1	1:D:355:ALA:N	2.55	0.40
1:D:397:LYS:HE2	1:D:397:LYS:HB2	1.95	0.40
1:E:612:LEU:HD11	1:E:628:TRP:CZ2	2.56	0.40
1:F:47:LEU:HD11	1:F:354:GLY:HA2	2.03	0.40
1:F:218:LYS:HG3	1:F:223:THR:HG22	2.04	0.40

There are no symmetry-related clashes.

5.3 Torsion angles (i)

5.3.1 Protein backbone (i)

In the following table, the Percentiles column shows the percent Ramachandran outliers of the chain as a percentile score with respect to all PDB entries followed by that with respect to all EM entries.

The Analysed column shows the number of residues for which the backbone conformation was analysed, and the total number of residues.

Mol	Chain	Analysed	Favoured	Allowed	Outliers	Percentiles	
1	A	804/825 (98%)	778 (97%)	26 (3%)	0	100	100
1	B	804/825 (98%)	778 (97%)	26 (3%)	0	100	100
1	C	804/825 (98%)	778 (97%)	26 (3%)	0	100	100
1	D	804/825 (98%)	779 (97%)	25 (3%)	0	100	100
1	E	804/825 (98%)	778 (97%)	26 (3%)	0	100	100
1	F	804/825 (98%)	777 (97%)	27 (3%)	0	100	100
1	G	804/825 (98%)	778 (97%)	26 (3%)	0	100	100

Continued on next page...

For Manuscript Review

Continued from previous page...

Mol	Chain	Analysed	Favoured	Allowed	Outliers	Percentiles	
1	H	804/825 (98%)	779 (97%)	25 (3%)	0	100	100
All	All	6432/6600 (98%)	6225 (97%)	207 (3%)	0	100	100

There are no Ramachandran outliers to report.

5.3.2 Protein sidechains ⓘ

In the following table, the Percentiles column shows the percent sidechain outliers of the chain as a percentile score with respect to all PDB entries followed by that with respect to all EM entries.

The Analysed column shows the number of residues for which the sidechain conformation was analysed, and the total number of residues.

Mol	Chain	Analysed	Rotameric	Outliers	Percentiles	
1	A	675/687 (98%)	675 (100%)	0	100	100
1	B	675/687 (98%)	675 (100%)	0	100	100
1	C	675/687 (98%)	675 (100%)	0	100	100
1	D	675/687 (98%)	675 (100%)	0	100	100
1	E	675/687 (98%)	675 (100%)	0	100	100
1	F	675/687 (98%)	675 (100%)	0	100	100
1	G	675/687 (98%)	675 (100%)	0	100	100
1	H	675/687 (98%)	675 (100%)	0	100	100
All	All	5400/5496 (98%)	5400 (100%)	0	100	100

There are no protein residues with a non-rotameric sidechain to report.

Sometimes sidechains can be flipped to improve hydrogen bonding and reduce clashes. All (26) such sidechains are listed below:

Mol	Chain	Res	Type
1	A	79	ASN
1	A	256	HIS
1	A	710	HIS
1	B	79	ASN
1	B	710	HIS
1	C	79	ASN
1	C	256	HIS
1	C	710	HIS

Continued on next page...

For Manuscript Review

Continued from previous page...

Mol	Chain	Res	Type
1	D	79	ASN
1	D	256	HIS
1	D	710	HIS
1	E	79	ASN
1	E	256	HIS
1	E	476	GLN
1	E	481	GLN
1	E	710	HIS
1	F	79	ASN
1	F	256	HIS
1	F	710	HIS
1	G	79	ASN
1	G	256	HIS
1	G	710	HIS
1	H	79	ASN
1	H	476	GLN
1	H	481	GLN
1	H	710	HIS

5.3.3 RNA ⓘ

There are no RNA molecules in this entry.

5.4 Non-standard residues in protein, DNA, RNA chains ⓘ

There are no non-standard protein/DNA/RNA residues in this entry.

5.5 Carbohydrates ⓘ

There are no monosaccharides in this entry.

5.6 Ligand geometry ⓘ

Of 16 ligands modelled in this entry, 8 are monoatomic - leaving 8 for Mogul analysis.

In the following table, the Counts columns list the number of bonds (or angles) for which Mogul statistics could be retrieved, the number of bonds (or angles) that are observed in the model and the number of bonds (or angles) that are defined in the Chemical Component Dictionary. The Link column lists molecule types, if any, to which the group is linked. The Z score for a bond length (or angle) is the number of standard deviations the observed value is removed from the

For Manuscript Review

expected value. A bond length (or angle) with $|Z| > 2$ is considered an outlier worth inspection. RMSZ is the root-mean-square of all Z scores of the bond lengths (or angles).

Mol	Type	Chain	Res	Link	Bond lengths			Bond angles		
					Counts	RMSZ	$\#\|Z\| > 2$	Counts	RMSZ	$\#\|Z\| > 2$
2	TPP	E	900	3	22,27,27	0.69	0	29,40,40	0.87	1 (3%)
2	TPP	A	900	3	22,27,27	0.67	0	29,40,40	0.88	1 (3%)
2	TPP	D	900	3	22,27,27	0.70	0	29,40,40	0.74	1 (3%)
2	TPP	H	900	3	22,27,27	0.70	0	29,40,40	0.74	1 (3%)
2	TPP	B	900	3	22,27,27	0.70	0	29,40,40	0.74	1 (3%)
2	TPP	G	900	3	22,27,27	0.67	0	29,40,40	0.87	1 (3%)
2	TPP	C	900	3	22,27,27	0.68	0	29,40,40	0.87	1 (3%)
2	TPP	F	900	3	22,27,27	0.70	0	29,40,40	0.74	1 (3%)

In the following table, the Chirals column lists the number of chiral outliers, the number of chiral centers analysed, the number of these observed in the model and the number defined in the Chemical Component Dictionary. Similar counts are reported in the Torsion and Rings columns. '-' means no outliers of that kind were identified.

Mol	Type	Chain	Res	Link	Chirals	Torsions	Rings
2	TPP	E	900	3	-	10/16/17/17	0/2/2/2
2	TPP	A	900	3	-	10/16/17/17	0/2/2/2
2	TPP	D	900	3	-	10/16/17/17	0/2/2/2
2	TPP	H	900	3	-	10/16/17/17	0/2/2/2
2	TPP	B	900	3	-	10/16/17/17	0/2/2/2
2	TPP	G	900	3	-	10/16/17/17	0/2/2/2
2	TPP	C	900	3	-	10/16/17/17	0/2/2/2
2	TPP	F	900	3	-	10/16/17/17	0/2/2/2

There are no bond length outliers.

All (8) bond angle outliers are listed below:

Mol	Chain	Res	Type	Atoms	Z	Observed(°)	Ideal(°)
2	B	900	TPP	C5-C4-N3	2.22	112.01	107.57
2	C	900	TPP	C5-C4-N3	2.22	112.01	107.57
2	A	900	TPP	C5-C4-N3	2.21	112.00	107.57
2	D	900	TPP	C5-C4-N3	2.21	112.00	107.57
2	H	900	TPP	C5-C4-N3	2.21	112.00	107.57
2	F	900	TPP	C5-C4-N3	2.20	111.97	107.57
2	E	900	TPP	C5-C4-N3	2.19	111.96	107.57

Continued on next page...

For Manuscript Review

Continued from previous page...

Mol	Chain	Res	Type	Atoms	Z	Observed(°)	Ideal(°)
2	G	900	TPP	C5-C4-N3	2.18	111.94	107.57

There are no chirality outliers.

All (80) torsion outliers are listed below:

Mol	Chain	Res	Type	Atoms
2	A	900	TPP	C4'-C5'-C7'-N3
2	A	900	TPP	C4-C5-C6-C7
2	A	900	TPP	C5-C6-C7-O7
2	A	900	TPP	C7-O7-PA-O1A
2	A	900	TPP	C7-O7-PA-O3A
2	A	900	TPP	PA-O3A-PB-O2B
2	B	900	TPP	C4'-C5'-C7'-N3
2	B	900	TPP	C4-C5-C6-C7
2	B	900	TPP	C5-C6-C7-O7
2	B	900	TPP	C7-O7-PA-O3A
2	C	900	TPP	C4'-C5'-C7'-N3
2	C	900	TPP	C4-C5-C6-C7
2	C	900	TPP	C5-C6-C7-O7
2	C	900	TPP	C7-O7-PA-O1A
2	C	900	TPP	C7-O7-PA-O3A
2	C	900	TPP	PA-O3A-PB-O2B
2	D	900	TPP	C4'-C5'-C7'-N3
2	D	900	TPP	C4-C5-C6-C7
2	D	900	TPP	C5-C6-C7-O7
2	D	900	TPP	C7-O7-PA-O3A
2	E	900	TPP	C4'-C5'-C7'-N3
2	E	900	TPP	C4-C5-C6-C7
2	E	900	TPP	C5-C6-C7-O7
2	E	900	TPP	C7-O7-PA-O1A
2	E	900	TPP	C7-O7-PA-O3A
2	E	900	TPP	PA-O3A-PB-O2B
2	F	900	TPP	C4'-C5'-C7'-N3
2	F	900	TPP	C4-C5-C6-C7
2	F	900	TPP	C5-C6-C7-O7
2	F	900	TPP	C7-O7-PA-O3A
2	G	900	TPP	C4'-C5'-C7'-N3
2	G	900	TPP	C4-C5-C6-C7
2	G	900	TPP	C5-C6-C7-O7
2	G	900	TPP	C7-O7-PA-O1A
2	G	900	TPP	C7-O7-PA-O3A
2	G	900	TPP	PA-O3A-PB-O2B

Continued on next page...

For Manuscript Review

Continued from previous page...

Mol	Chain	Res	Type	Atoms
2	H	900	TPP	C4'-C5'-C7'-N3
2	H	900	TPP	C4-C5-C6-C7
2	H	900	TPP	C5-C6-C7-O7
2	H	900	TPP	C7-O7-PA-O3A
2	B	900	TPP	PA-O3A-PB-O1B
2	D	900	TPP	PA-O3A-PB-O1B
2	F	900	TPP	PA-O3A-PB-O1B
2	H	900	TPP	PA-O3A-PB-O1B
2	A	900	TPP	PB-O3A-PA-O2A
2	B	900	TPP	PB-O3A-PA-O1A
2	C	900	TPP	PB-O3A-PA-O2A
2	D	900	TPP	PB-O3A-PA-O1A
2	E	900	TPP	PB-O3A-PA-O2A
2	F	900	TPP	PB-O3A-PA-O1A
2	G	900	TPP	PB-O3A-PA-O2A
2	H	900	TPP	PB-O3A-PA-O1A
2	A	900	TPP	C7-O7-PA-O2A
2	B	900	TPP	C7-O7-PA-O1A
2	B	900	TPP	C7-O7-PA-O2A
2	C	900	TPP	C7-O7-PA-O2A
2	D	900	TPP	C7-O7-PA-O1A
2	D	900	TPP	C7-O7-PA-O2A
2	E	900	TPP	C7-O7-PA-O2A
2	F	900	TPP	C7-O7-PA-O1A
2	F	900	TPP	C7-O7-PA-O2A
2	G	900	TPP	C7-O7-PA-O2A
2	H	900	TPP	C7-O7-PA-O1A
2	H	900	TPP	C7-O7-PA-O2A
2	A	900	TPP	PA-O3A-PB-O3B
2	B	900	TPP	PA-O3A-PB-O2B
2	B	900	TPP	PA-O3A-PB-O3B
2	C	900	TPP	PA-O3A-PB-O3B
2	D	900	TPP	PA-O3A-PB-O2B
2	D	900	TPP	PA-O3A-PB-O3B
2	E	900	TPP	PA-O3A-PB-O3B
2	F	900	TPP	PA-O3A-PB-O2B
2	F	900	TPP	PA-O3A-PB-O3B
2	G	900	TPP	PA-O3A-PB-O3B
2	H	900	TPP	PA-O3A-PB-O2B
2	H	900	TPP	PA-O3A-PB-O3B
2	A	900	TPP	PB-O3A-PA-O1A
2	C	900	TPP	PB-O3A-PA-O1A

Continued on next page...

For Manuscript Review

Continued from previous page...

Mol	Chain	Res	Type	Atoms
2	E	900	TPP	PB-O3A-PA-O1A
2	G	900	TPP	PB-O3A-PA-O1A

There are no ring outliers.

4 monomers are involved in 4 short contacts:

Mol	Chain	Res	Type	Clashes	Symm-Clashes
2	E	900	TPP	1	0
2	H	900	TPP	1	0
2	G	900	TPP	1	0
2	F	900	TPP	1	0

The following is a two-dimensional graphical depiction of Mogul quality analysis of bond lengths, bond angles, torsion angles, and ring geometry for all instances of the Ligand of Interest. In addition, ligands with molecular weight > 250 and outliers as shown on the validation Tables will also be included. For torsion angles, if less then 5% of the Mogul distribution of torsion angles is within 10 degrees of the torsion angle in question, then that torsion angle is considered an outlier. Any bond that is central to one or more torsion angles identified as an outlier by Mogul will be highlighted in the graph. For rings, the root-mean-square deviation (RMSD) between the ring in question and similar rings identified by Mogul is calculated over all ring torsion angles. If the average RMSD is greater than 60 degrees and the minimal RMSD between the ring in question and any Mogul-identified rings is also greater than 60 degrees, then that ring is considered an outlier. The outliers are highlighted in purple. The color gray indicates Mogul did not find sufficient equivalents in the CSD to analyse the geometry.

Ligand TPP E 900

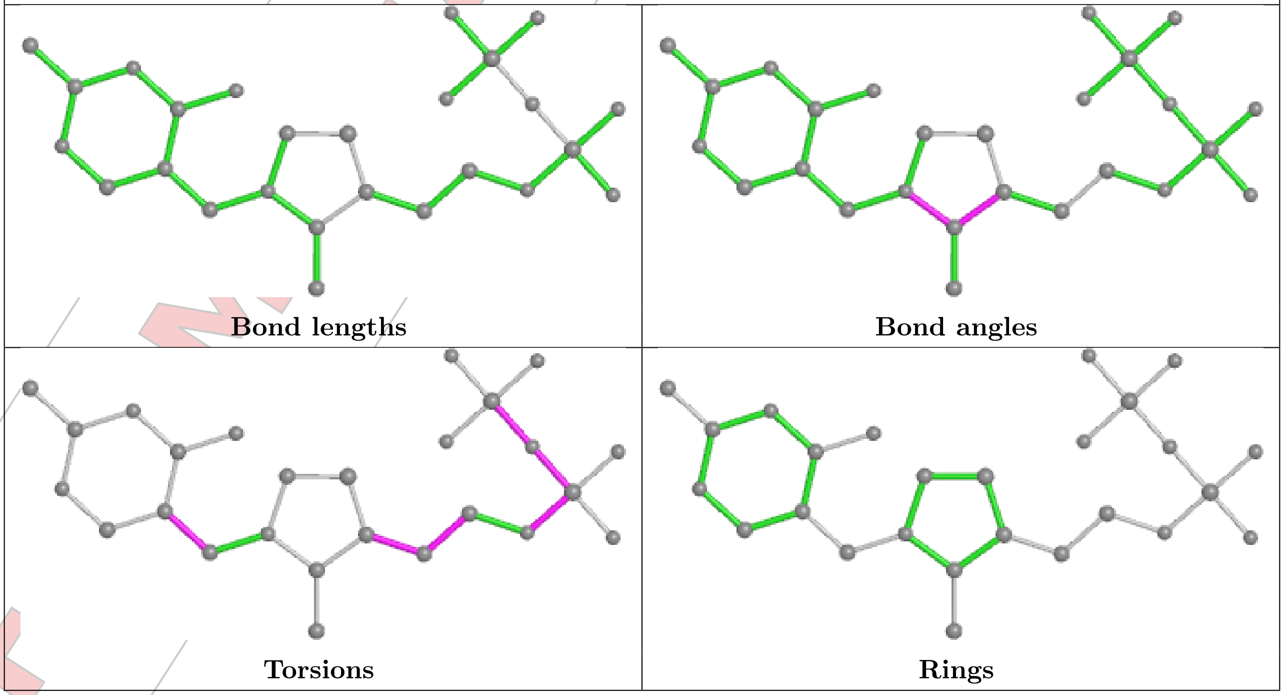

Bond lengths — Bond angles — Torsions — Rings

For Manuscript Review

Ligand TPP A 900

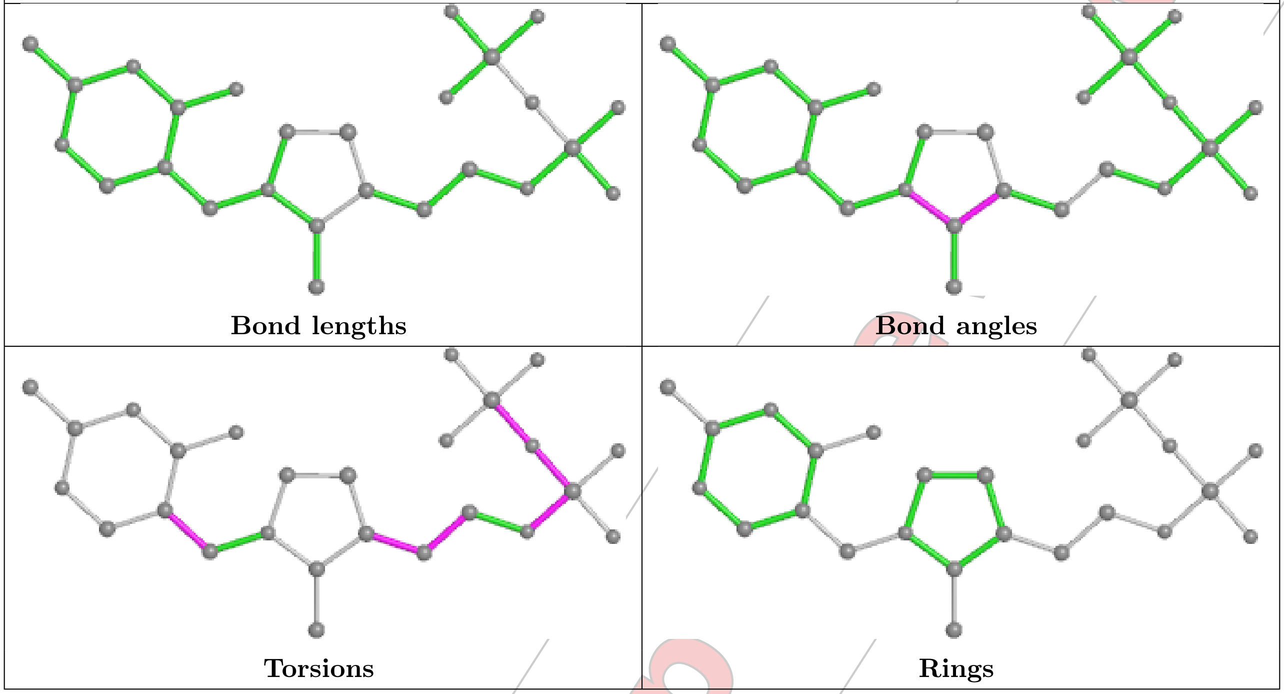

Ligand TPP D 900

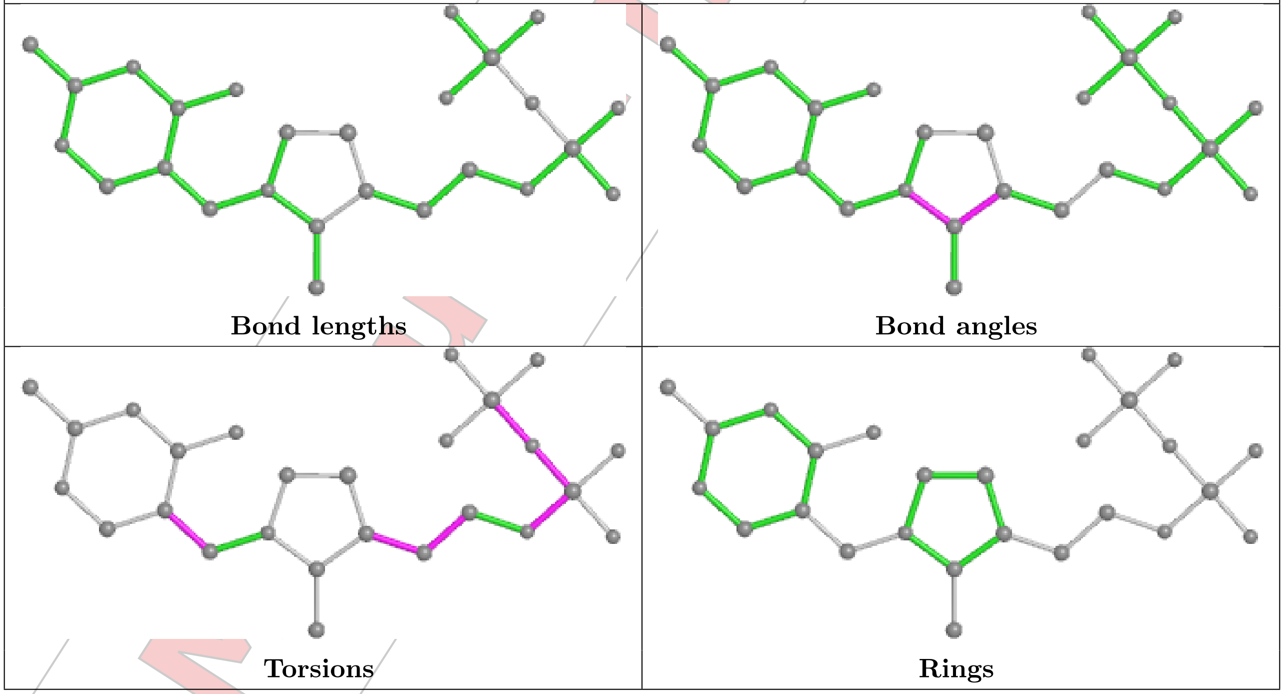

For Manuscript Review

Ligand TPP H 900

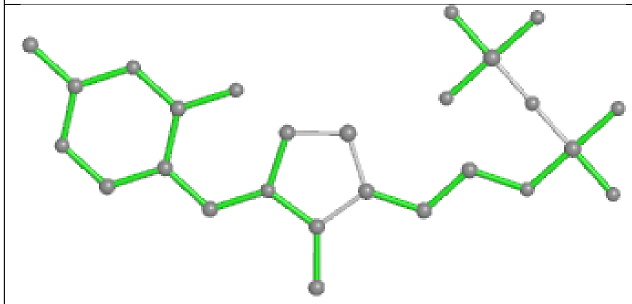

Bond lengths

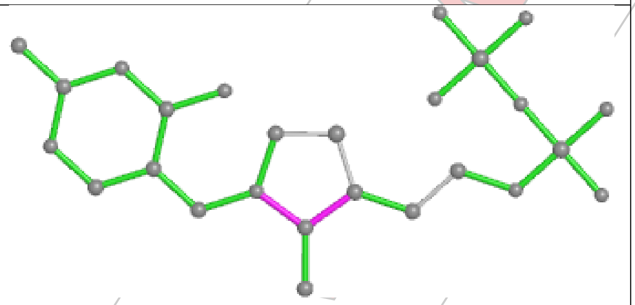

Bond angles

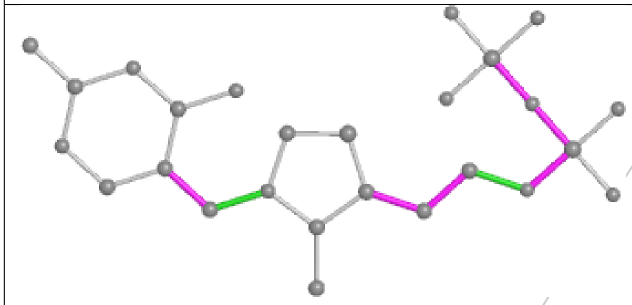

Torsions

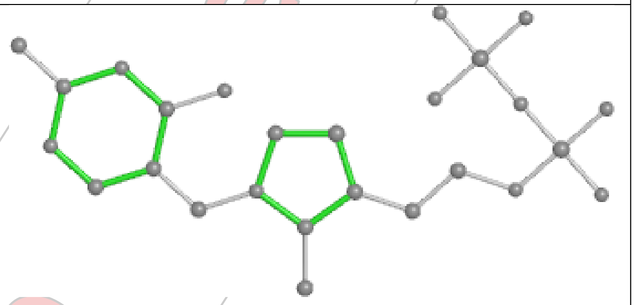

Rings

Ligand TPP B 900

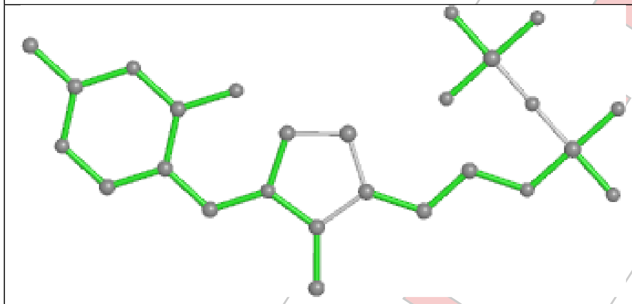

Bond lengths

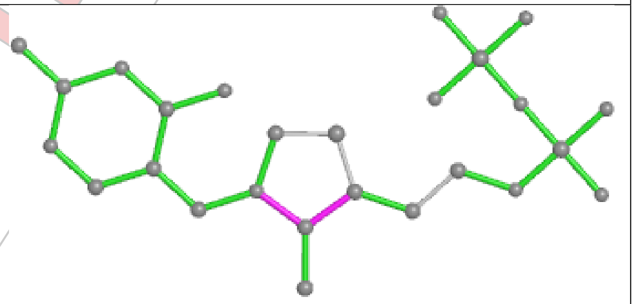

Bond angles

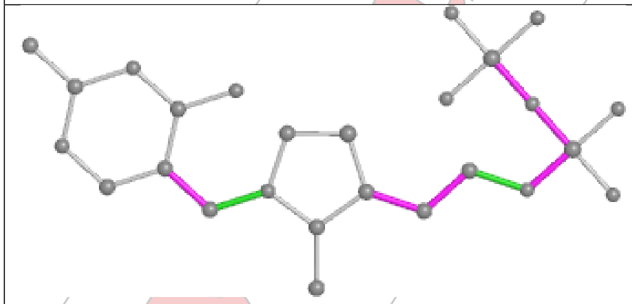

Torsions

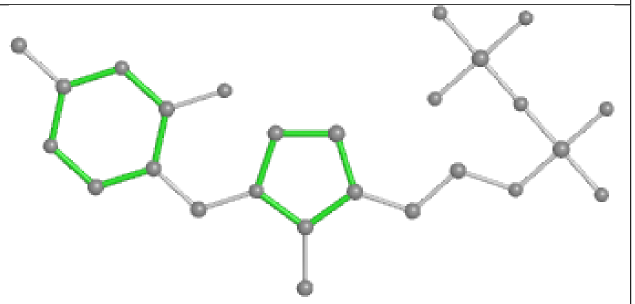

Rings

Ligand TPP G 900

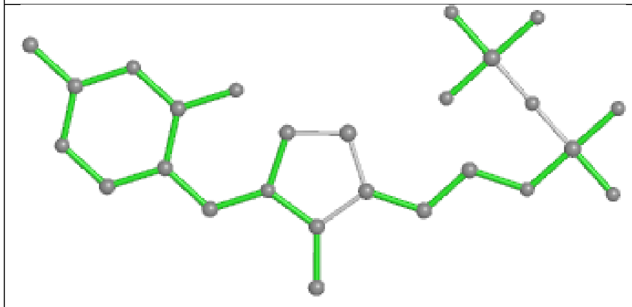
Bond lengths

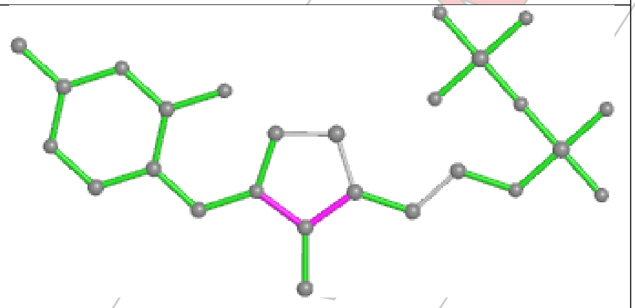
Bond angles

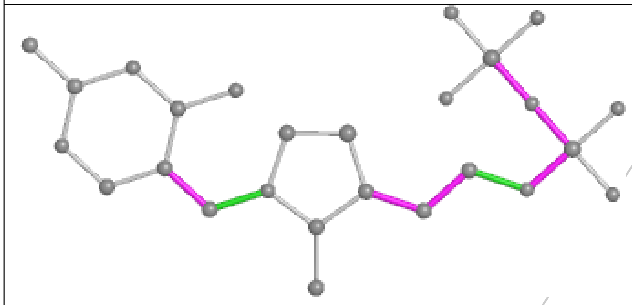
Torsions

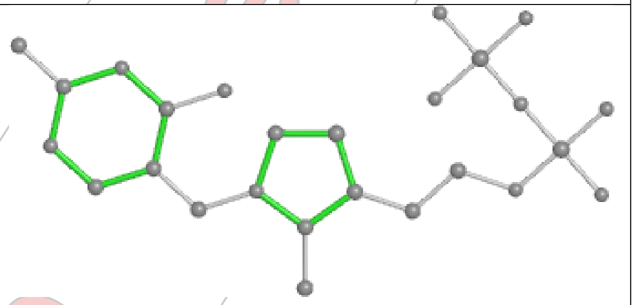
Rings

Ligand TPP C 900

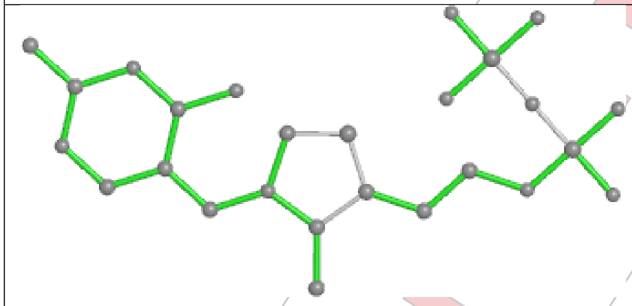
Bond lengths

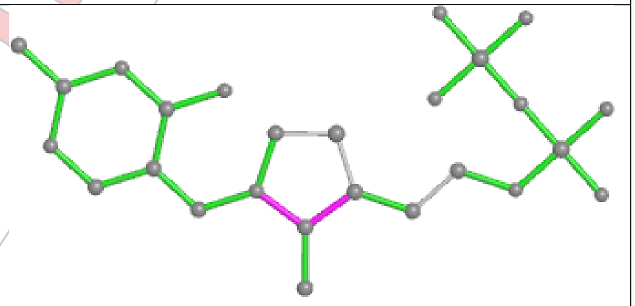
Bond angles

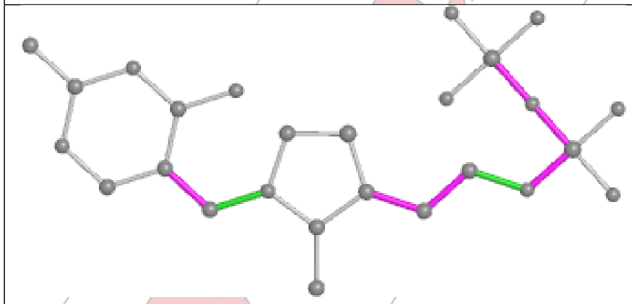
Torsions

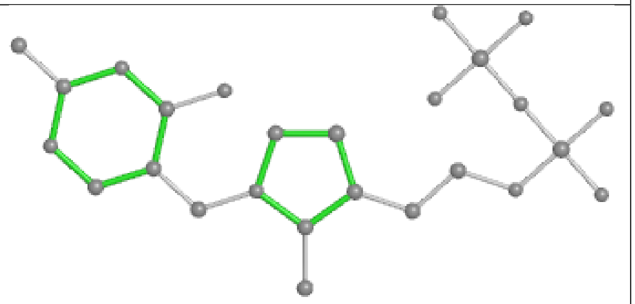
Rings

Ligand TPP F 900	
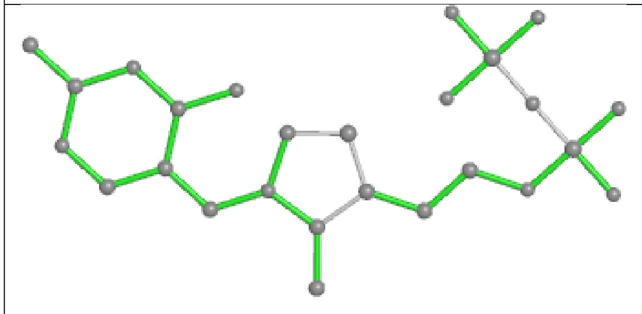 Bond lengths	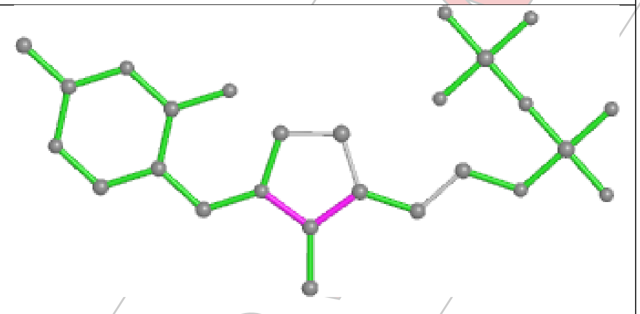 Bond angles
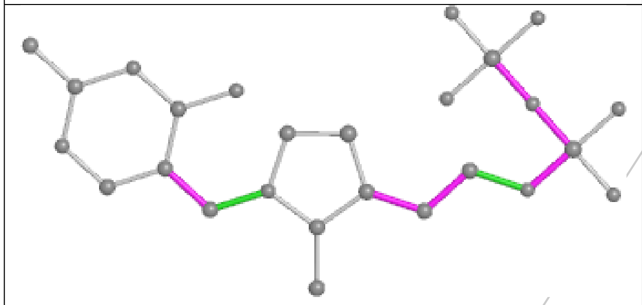 Torsions	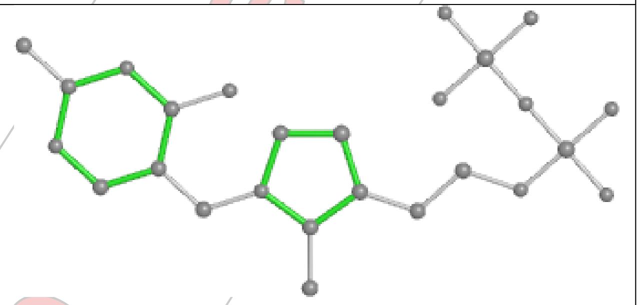 Rings

5.7 Other polymers (i)

There are no such residues in this entry.

5.8 Polymer linkage issues (i)

There are no chain breaks in this entry.

For Manuscript Review

6 Map visualisation (i)

This section contains visualisations of the EMDB entry EMD-35609. These allow visual inspection of the internal detail of the map and identification of artifacts.

Images derived from a raw map, generated by summing the deposited half-maps, are presented below the corresponding image components of the primary map to allow further visual inspection and comparison with those of the primary map.

6.1 Orthogonal projections (i)

6.1.1 Primary map

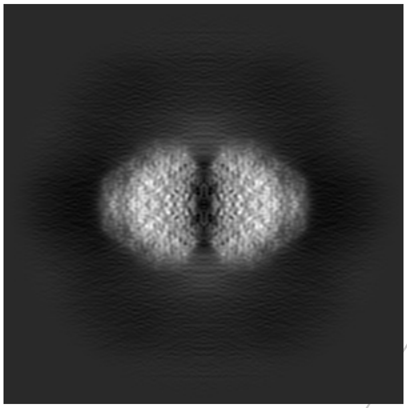

X

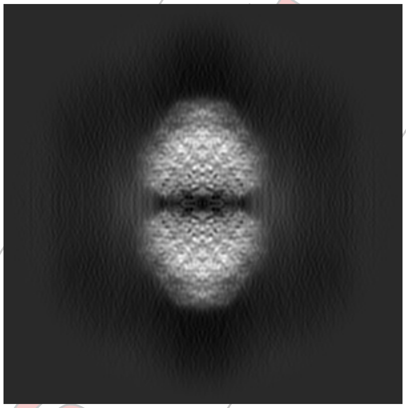

Y

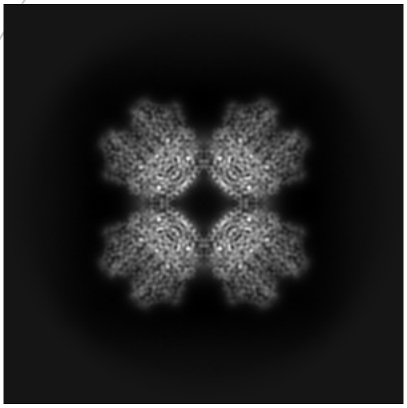

Z

6.1.2 Raw map

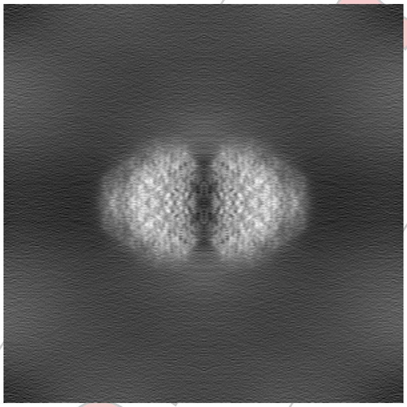

X

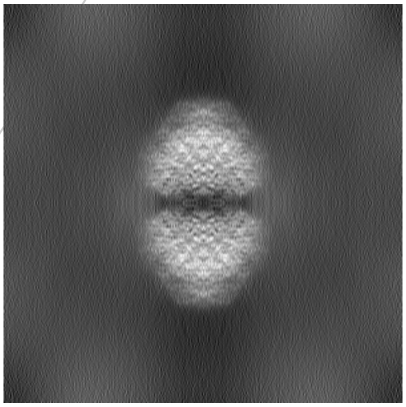

Y

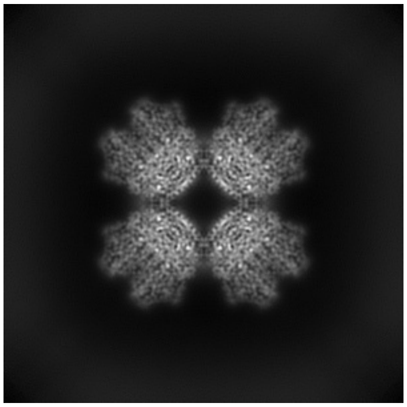

Z

The images above show the map projected in three orthogonal directions.

For Manuscript Review

6.2 Central slices (i)

6.2.1 Primary map

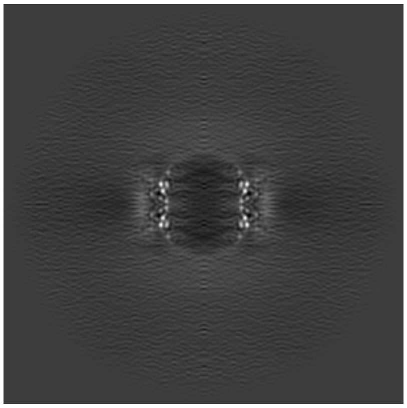

X Index: 192

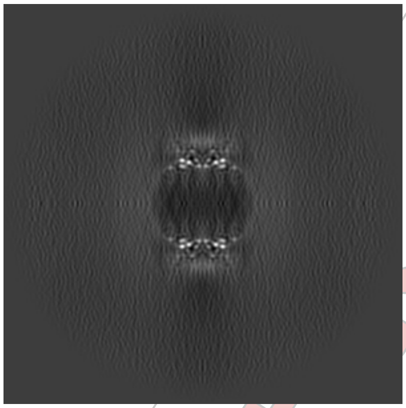

Y Index: 192

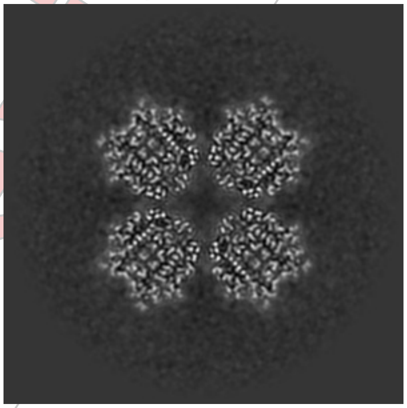

Z Index: 192

6.2.2 Raw map

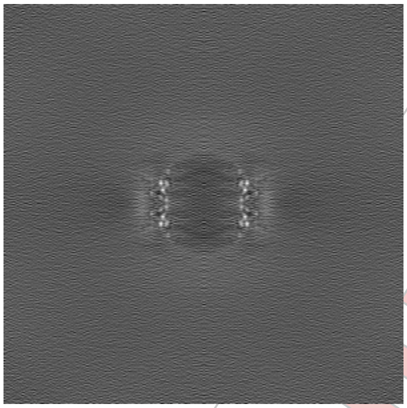

X Index: 192

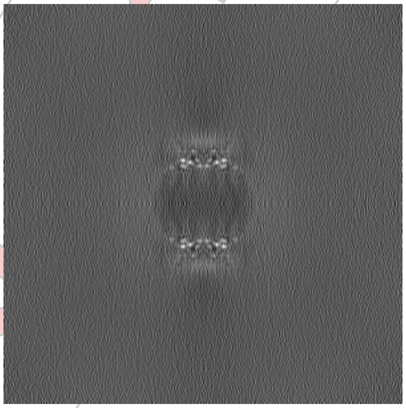

Y Index: 192

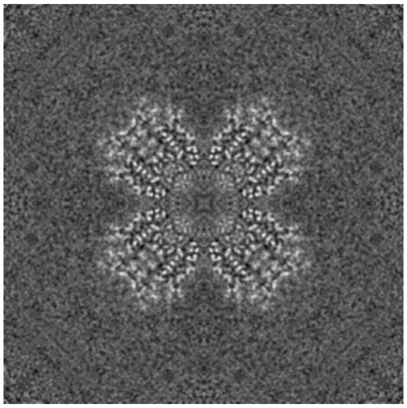

Z Index: 192

The images above show central slices of the map in three orthogonal directions.

For Manuscript Review

6.3 Largest variance slices (i)

6.3.1 Primary map

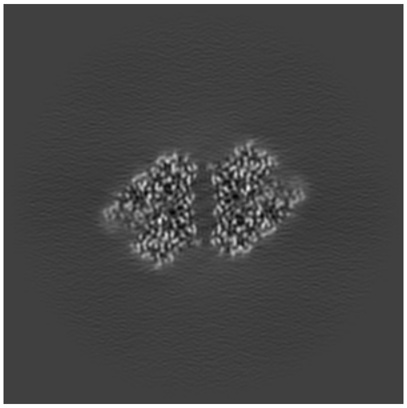
X Index: 217

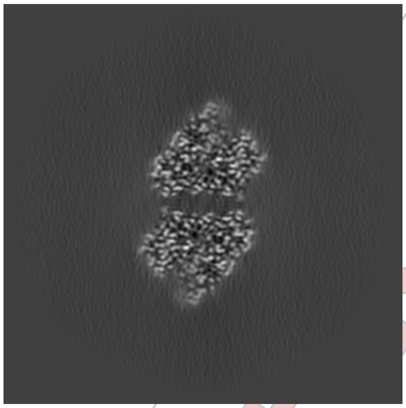
Y Index: 167

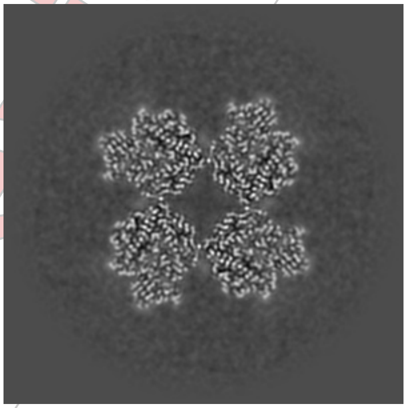
Z Index: 197

6.3.2 Raw map

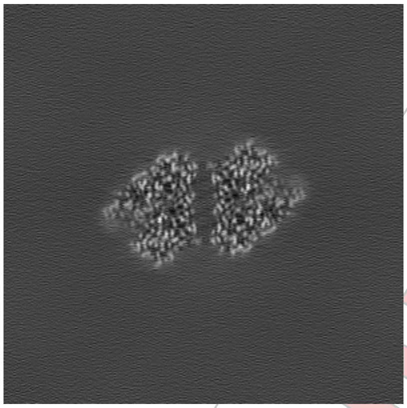
X Index: 167

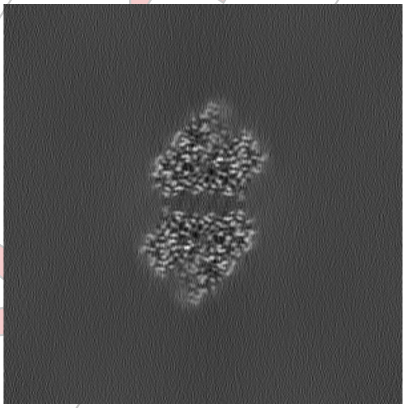
Y Index: 217

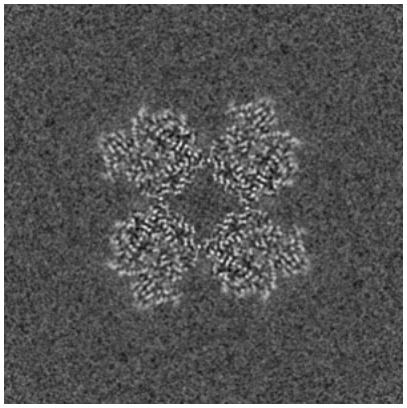
Z Index: 186

The images above show the largest variance slices of the map in three orthogonal directions.

For Manuscript Review

6.4 Orthogonal surface views (i)

6.4.1 Primary map

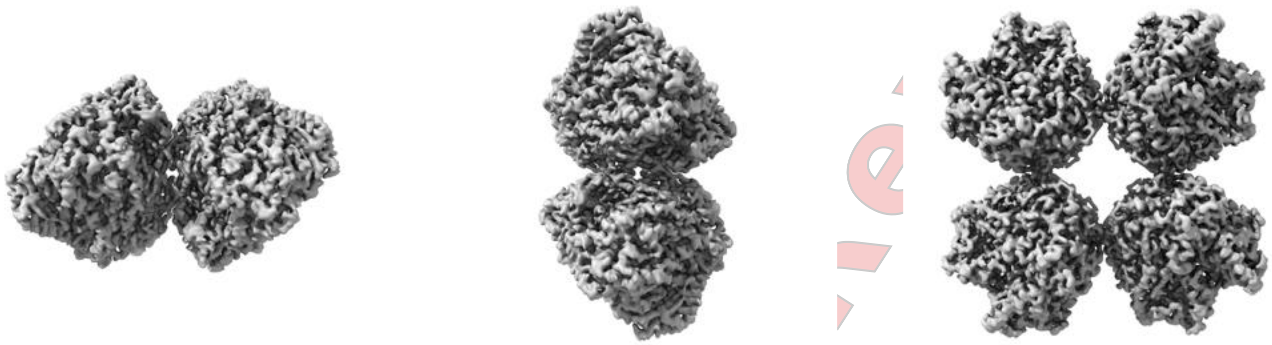

X Y Z

The images above show the 3D surface view of the map at the recommended contour level 0.238. These images, in conjunction with the slice images, may facilitate assessment of whether an appropriate contour level has been provided.

6.4.2 Raw map

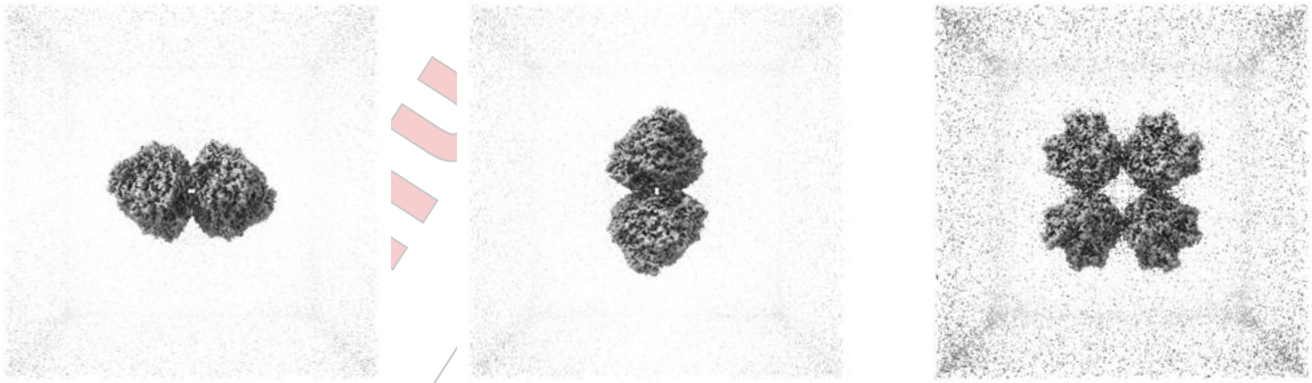

X Y Z

These images show the 3D surface of the raw map. The raw map's contour level was selected so that its surface encloses the same volume as the primary map does at its recommended contour level.

6.5 Mask visualisation (i)

This section was not generated. No masks/segmentation were deposited.

For Manuscript Review

7 Map analysis (i)

This section contains the results of statistical analysis of the map.

7.1 Map-value distribution (i)

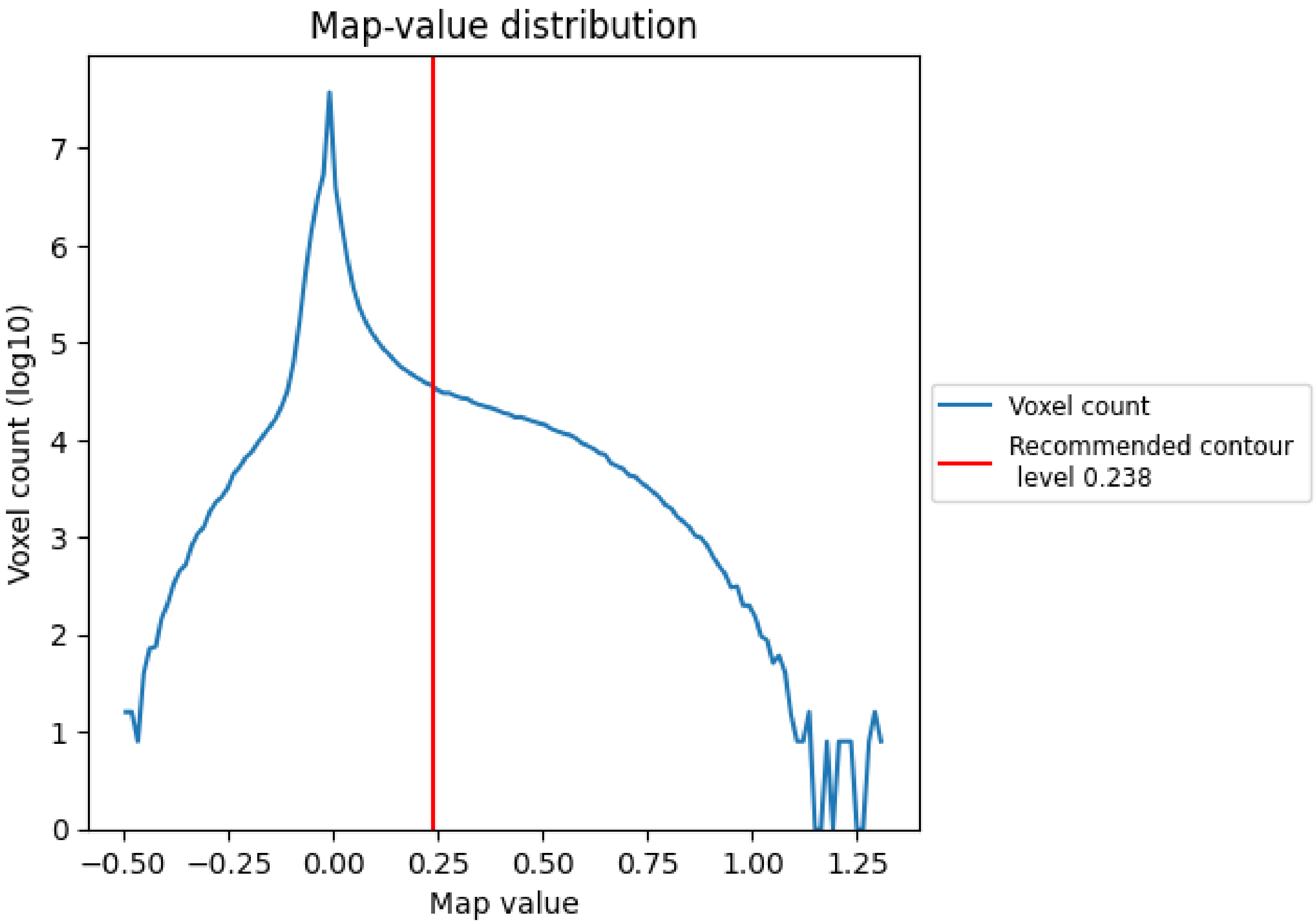

The map-value distribution is plotted in 128 intervals along the x-axis. The y-axis is logarithmic. A spike in this graph at zero usually indicates that the volume has been masked.

For Manuscript Review

7.2 Volume estimate ⓘ

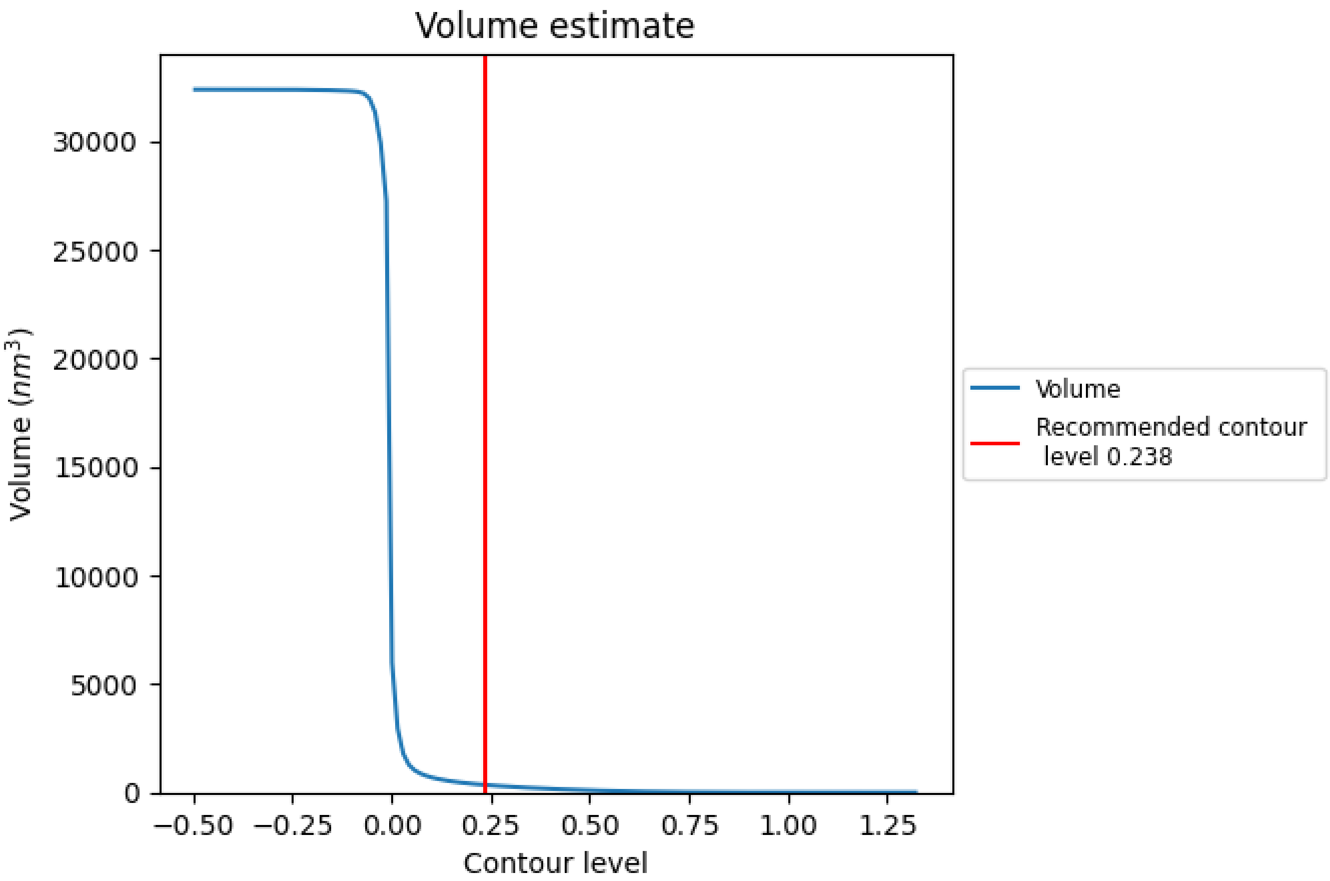

The volume at the recommended contour level is 342 nm^3; this corresponds to an approximate mass of 309 kDa.

The volume estimate graph shows how the enclosed volume varies with the contour level. The recommended contour level is shown as a vertical line and the intersection between the line and the curve gives the volume of the enclosed surface at the given level.

For Manuscript Review

7.3 Rotationally averaged power spectrum (i)

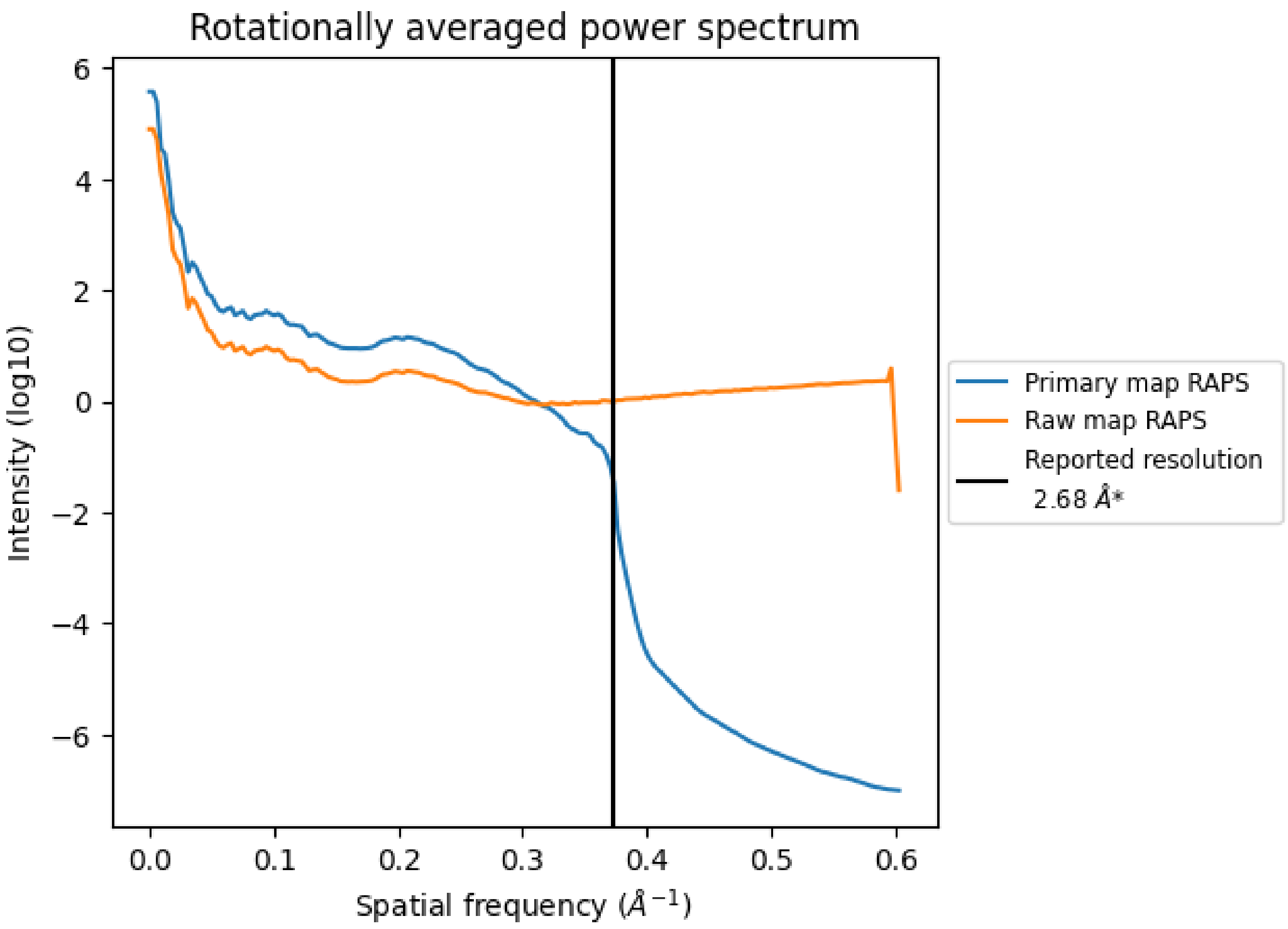

*Reported resolution corresponds to spatial frequency of 0.373 Å^{-1}

For Manuscript Review

8 Fourier-Shell correlation (i)

Fourier-Shell Correlation (FSC) is the most commonly used method to estimate the resolution of single-particle and subtomogram-averaged maps. The shape of the curve depends on the imposed symmetry, mask and whether or not the two 3D reconstructions used were processed from a common reference. The reported resolution is shown as a black line. A curve is displayed for the half-bit criterion in addition to lines showing the 0.143 gold standard cut-off and 0.5 cut-off.

8.1 FSC (i)

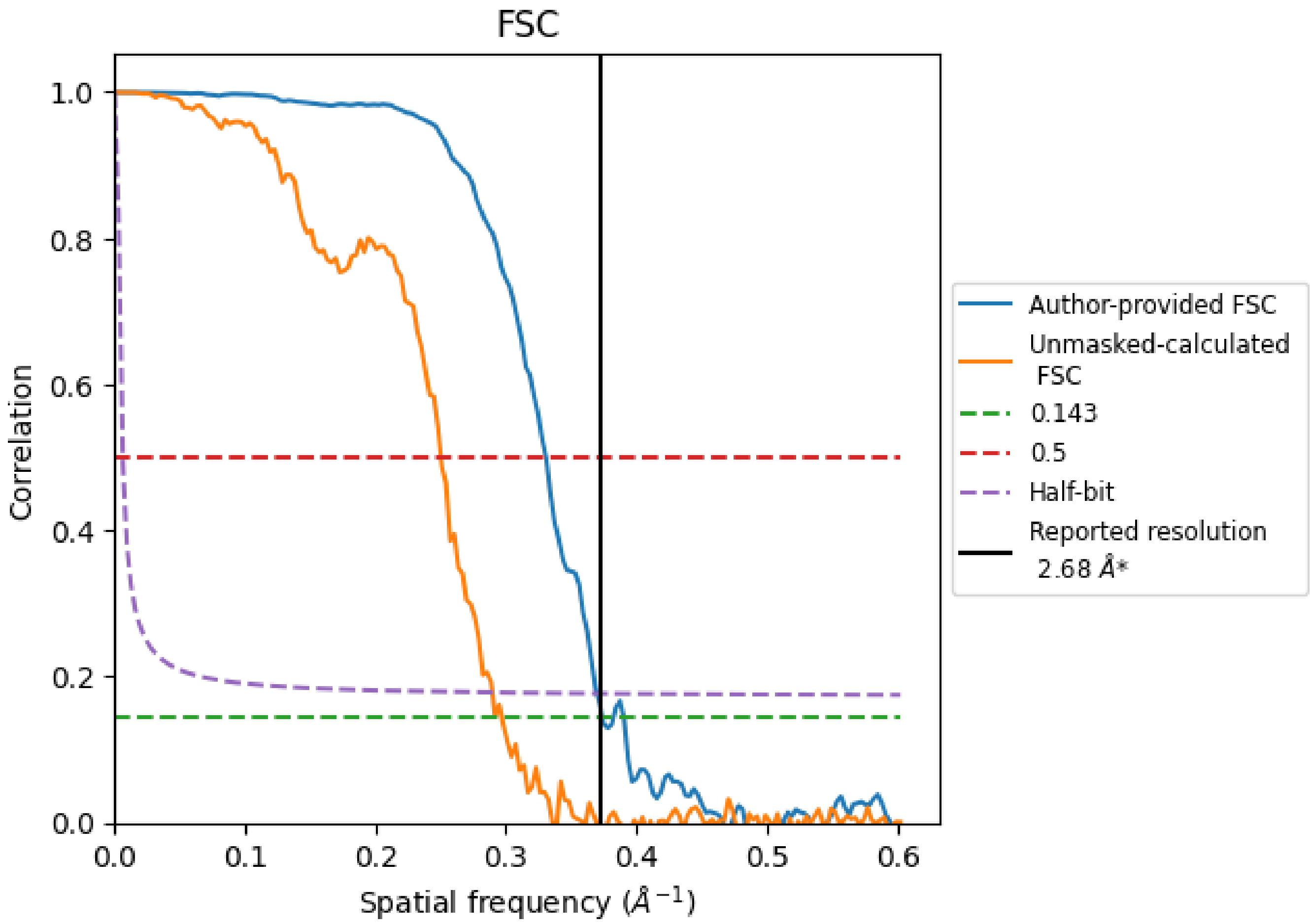

*Reported resolution corresponds to spatial frequency of 0.373 Å^{-1}

For Manuscript Review

8.2 Resolution estimates (i)

Resolution estimate (Å)	Estimation criterion (FSC cut-off)		
	0.143	0.5	Half-bit
Reported by author	2.68	-	-
Author-provided FSC curve	2.67	3.02	2.70
Unmasked-calculated*	3.37	4.00	3.45

*Resolution estimate based on FSC curve calculated by comparison of deposited half-maps. The value from deposited half-maps intersecting FSC 0.143 CUT-OFF 3.37 differs from the reported value 2.68 by more than 10 %

For Manuscript Review

9 Map-model fit (i)

This section contains information regarding the fit between EMDB map EMD-35609 and PDB model 8IO6. Per-residue inclusion information can be found in section 3 on page 7.

9.1 Map-model overlay (i)

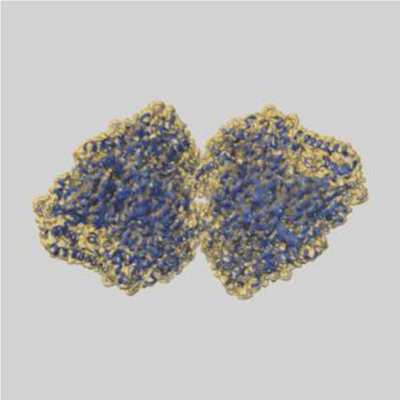

X

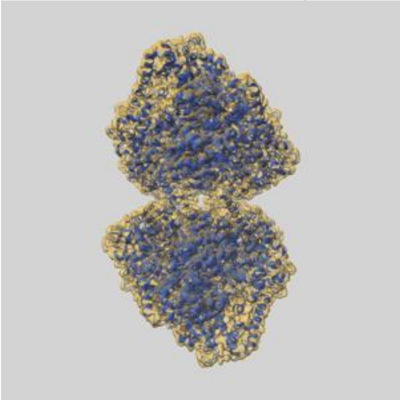

Y

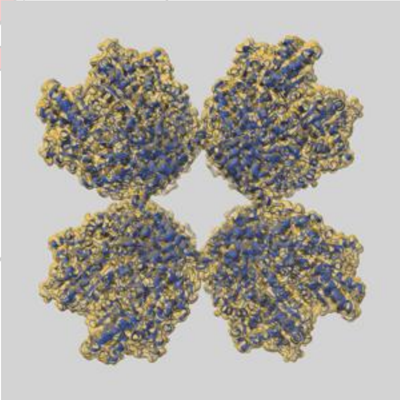

Z

The images above show the 3D surface view of the map at the recommended contour level 0.238 at 50% transparency in yellow overlaid with a ribbon representation of the model coloured in blue. These images allow for the visual assessment of the quality of fit between the atomic model and the map.

For Manuscript Review

9.2 Q-score mapped to coordinate model (i)

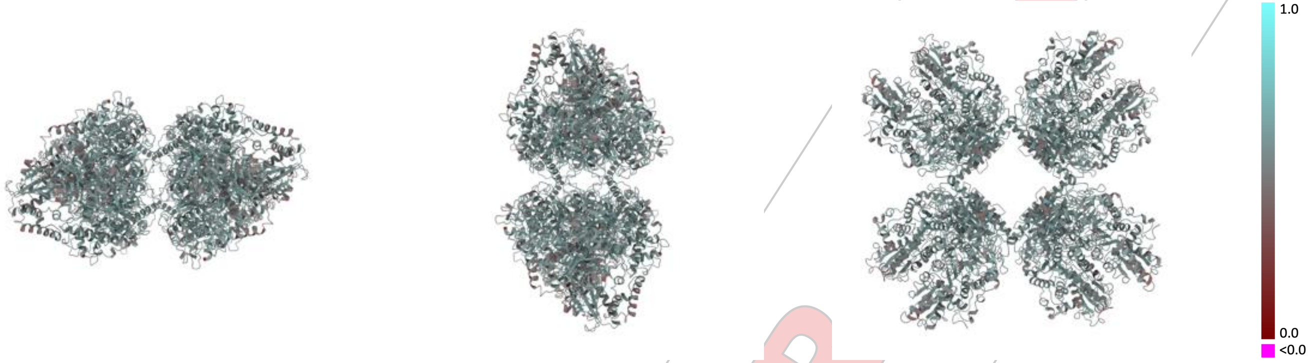

X Y Z

The images above show the model with each residue coloured according its Q-score. This shows their resolvability in the map with higher Q-score values reflecting better resolvability. Please note: Q-score is calculating the resolvability of atoms, and thus high values are only expected at resolutions at which atoms can be resolved. Low Q-score values may therefore be expected for many entries.

9.3 Atom inclusion mapped to coordinate model (i)

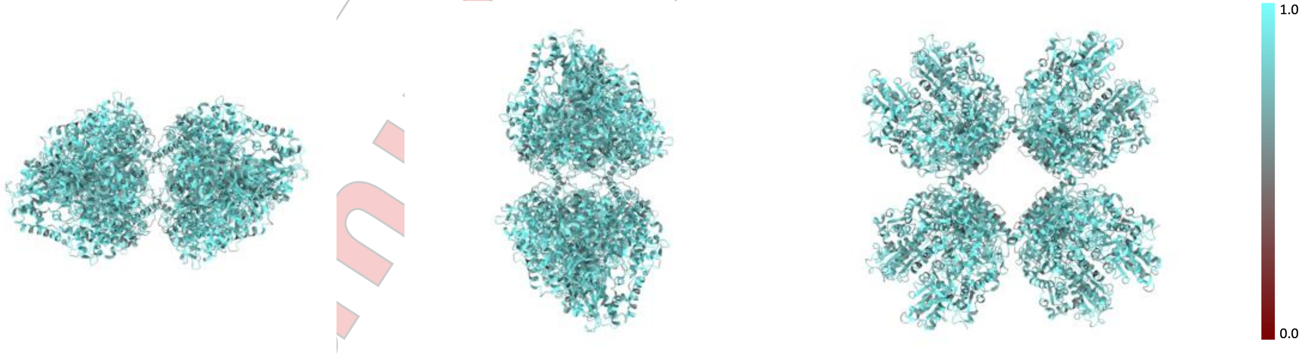

X Y Z

The images above show the model with each residue coloured according to its atom inclusion. This shows to what extent they are inside the map at the recommended contour level (0.238).

For Manuscript Review

9.4 Atom inclusion (i)

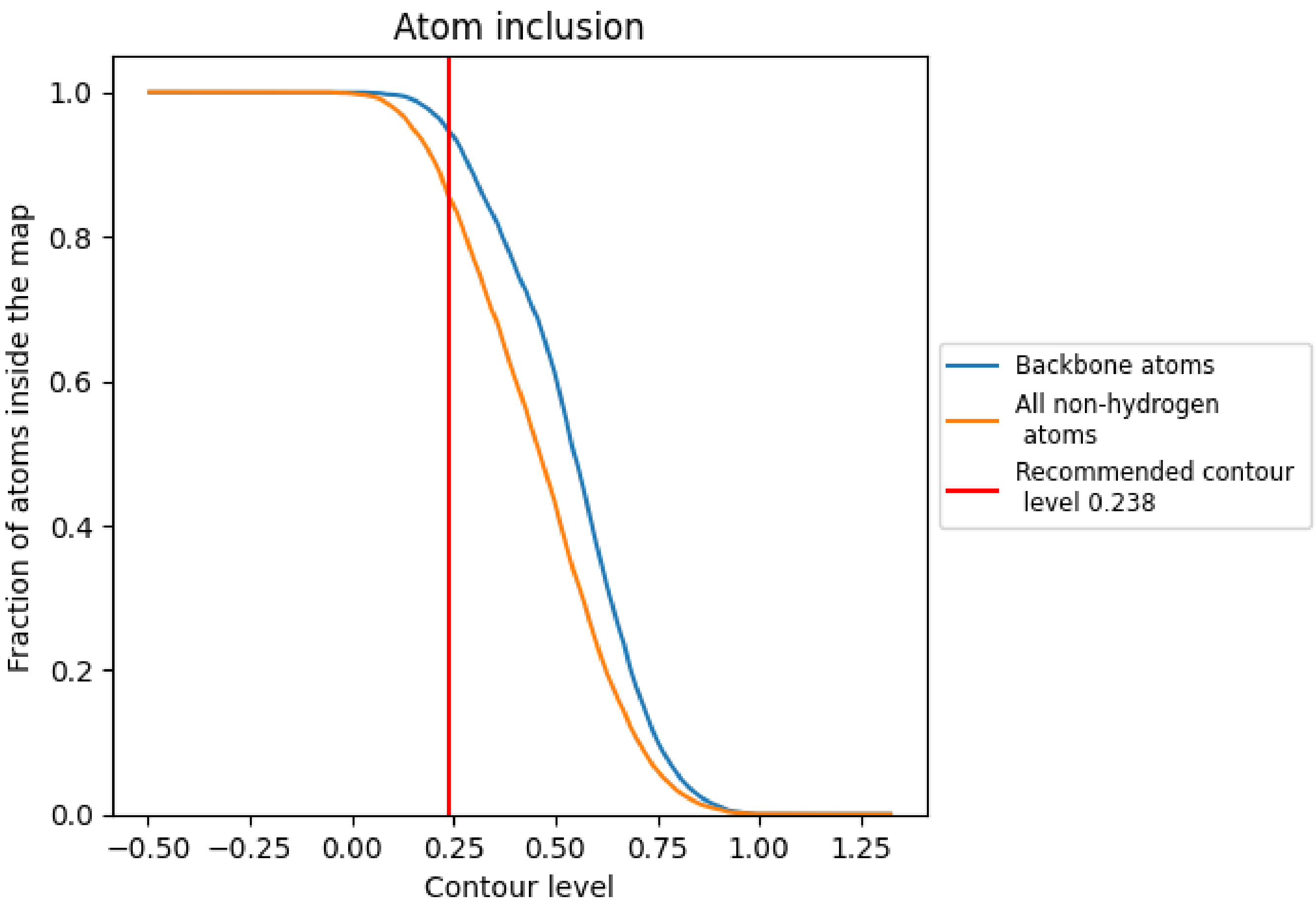

At the recommended contour level, 95% of all backbone atoms, 86% of all non-hydrogen atoms, are inside the map.

For Manuscript Review

9.5 Map-model fit summary (i)

The table lists the average atom inclusion at the recommended contour level (0.238) and Q-score for the entire model and for each chain.

Chain	Atom inclusion	Q-score
All	0.8550	0.5480
A	0.8551	0.5470
B	0.8549	0.5480
C	0.8552	0.5480
D	0.8549	0.5480
E	0.8555	0.5480
F	0.8546	0.5480
G	0.8547	0.5480
H	0.8554	0.5480

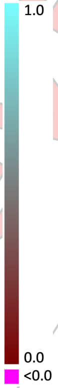

For Manuscript Review
